# Mapping Digital Public Health Interventions Among Existing Digital Technologies and Internet-Based Interventions to Maintain and Improve Population Health in Practice: Scoping Review

**DOI:** 10.2196/53927

**Published:** 2024-07-17

**Authors:** Laura Maaß, Konstantinos Angoumis, Merle Freye, Chen-Chia Pan

**Affiliations:** 1 University of Bremen, SOCIUM Research Center on Inequality and Social Policy Bremen Germany; 2 Leibniz ScienceCampus Digital Public Health Bremen Bremen Germany; 3 Digital Health Section European Public Health Association - EUPHA Utrecht Netherlands; 4 University of Bielefeld Bielefeld Germany; 5 Leibniz Institute for Prevention Research and Epidemiology - BIPS Bremen Germany; 6 University of Bremen, Institute for Information, Health and Medical Law - IGMR Bremen Germany; 7 University of Bremen, Institute for Public Health and Nursing Research – IPP Bremen Germany

**Keywords:** digital public health, digital health, public health, telemedicine, electronic health records, e-prescription, e-referral, e-consultation, e-surveillance, e-vaccination registries, scoping review

## Abstract

**Background:**

The rapid progression and integration of digital technologies into public health have reshaped the global landscape of health care delivery and disease prevention. In pursuit of better population health and health care accessibility, many countries have integrated digital interventions into their health care systems, such as web-based consultations, electronic health records, and telemedicine. Despite the increasing prevalence and relevance of digital technologies in public health and their varying definitions, there has been a shortage of studies examining whether these technologies align with the established definition and core characteristics of digital public health (DiPH) interventions. Hence, the imperative need for a scoping review emerges to explore the breadth of literature dedicated to this subject.

**Objective:**

This scoping review aims to outline DiPH interventions from different implementation stages for health promotion, primary to tertiary prevention, including health care and disease surveillance and monitoring. In addition, we aim to map the reported intervention characteristics, including their technical features and nontechnical elements.

**Methods:**

Original studies or reports of DiPH intervention focused on population health were eligible for this review. PubMed, Web of Science, CENTRAL, IEEE Xplore, and the ACM Full-Text Collection were searched for relevant literature (last updated on October 5, 2022). Intervention characteristics of each identified DiPH intervention, such as target groups, level of prevention or health care, digital health functions, intervention types, and public health functions, were extracted and used to map DiPH interventions. MAXQDA 2022.7 (VERBI GmbH) was used for qualitative data analysis of such interventions’ technical functions and nontechnical characteristics.

**Results:**

In total, we identified and screened 15,701 records, of which 1562 (9.94%) full texts were considered relevant and were assessed for eligibility. Finally, we included 185 (11.84%) publications, which reported 179 different DiPH interventions. Our analysis revealed a diverse landscape of interventions, with telemedical services, health apps, and electronic health records as dominant types. These interventions targeted a wide range of populations and settings, demonstrating their adaptability. The analysis highlighted the multifaceted nature of digital interventions, necessitating precise definitions and standardized terminologies for effective collaboration and evaluation.

**Conclusions:**

Although this scoping review was able to map characteristics and technical functions among 13 intervention types in DiPH, emerging technologies such as artificial intelligence might have been underrepresented in our study. This review underscores the diversity of DiPH interventions among and within intervention groups. Moreover, it highlights the importance of precise terminology for effective planning and evaluation. This review promotes cross-disciplinary collaboration by emphasizing the need for clear definitions, distinct technological functions, and well-defined use cases. It lays the foundation for international benchmarks and comparability within DiPH systems. Further research is needed to map intervention characteristics in this still-evolving field continuously.

**Trial Registration:**

PROSPERO CRD42021265562; https://tinyurl.com/43jksb3k

**International Registered Report Identifier (IRRID):**

RR2-10.2196/33404

## Introduction

### Background

Digital technologies have become ubiquitous in all facets of life and work [1]. Within health care, digital solutions offer a promising opportunity to make processes and operational methods more efficient and effective [2,3]. This advancement in health care and public health will empower individuals to engage in self-management and actively manage their overall well-being and health [4]. Similarly, digital technologies can facilitate the cost-efficient implementation of health promotion, prevention, monitoring, and surveillance measures, making them easy to access [5-11]. These technologies also foster novel care possibilities, enabling sustainable and equitable access to health services across entire populations [12-16]. Therefore, adopting digital health solutions presents a valuable opportunity to strengthen national health care systems. Eventually, these solutions will transform traditional clinical care and public health structures, fundamentally reshaping health systems on a global scale.

However, to our knowledge, no study has yet attempted to comprehensively map out the heterogeneous landscape of digital public health (DiPH) and digital public health interventions (DiPHIs). Despite existing reviews focusing on selected intervention categories [17], specific target groups such as adolescents [18,19] or patients who are chronically ill with specific medical conditions [20], a comprehensive mapping of interventions in digital health is still missing. In addition, in all existing reviews, the studies analyzed digital health interventions but not DiPHIs, thereby limiting these interventions to their medical and clinical usability. Therefore, this scoping review aims to fill the research gap and comprehensively map out the heterogeneous landscape of DiPHIs. The development, implementation, integration, and evaluation of evidence- and needs-based DiPH interventions require a clear and shared understanding of the specific characteristics of digital health technologies for public health purposes [21]. Conversely, the lack of a standardized lexicon creates communication challenges between the subdisciplines of DiPH [22,23], while concurrently hindering comparability between national health systems.

### Definition of DiPH

For this review, DiPH is defined as using information and communication technologies (ICTs) such as digital technologies, services, or tools to achieve traditional public health goals [24] with a population health impact [25]. According to Winslow [26], public health aims at “preventing disease, prolonging life, and promoting physical and mental health and efficiency through organized community efforts...” This definition is still used nowadays by the World Health Organization (WHO) to define essential public health functions. This places public health as the leading discipline in health governance, financing, health information systems, communication, coverage, care, education, and health regulations to protect and promote the health of populations [27].

Hence, we define DiPHI as interventions that address “at least one essential public health function through digital means.” [26]. To enhance acceptance among the population, DiPHI should consistently follow a needs- and evidence-based design with a participatory and human-centric approach [21,25,28,29]. The impetus behind their development should not stem solely from technological capabilities but from DiPHIs potential to mitigate disparities and improve access to health services among different groups of a population [22,25,27].

Unlike the domain of *digital health*, which involves the application of health ICT for personalized medicine and telemedicine [30] and its subfield *mobile health*, which primarily focuses on patient monitoring and the use of personal digital assistants in the clinical setting [31], DiPHIs encompass a broader spectrum of services and interventions. These include those in health promotion, such as wearables to promote physical activity; in prevention, such as web-based vaccination registries; in health care, such as telemedicine or electronic health records (EHRs); and in surveillance systems, such as dashboards or tracing systems for infectious disease monitoring on a national level. The goal of DiPHIs is to improve the health and well-being of groups considered vulnerable rather than individuals [25].

By combining the foundational goals of public health with the capabilities of ICT applications, DiPHIs have the potential to substantially strengthen population health, promote well-being, and advance public health objectives. This impact can be realized at the individual, community, and national levels [32].

### Study Aims and Objective

This scoping review primarily aims to outline a comprehensive range of proposed to implemented DiPHI, including different levels of prevention, health care, and public health research initiatives. Following the aforementioned definition of DiPHI, the second objective is to map the landscape of the existing DiPHI, shedding light on their self-reported digital health functions (based on the evidence standards framework [ESF] for digital health technologies from the National Institute for Health and Care Excellence [NICE] [33]) and the addressed essential public health functions as defined by the WHO [27]. Our scoping review will guide future research to address underexplored DiPHI types by identifying research gaps (eg, underrepresented intervention types). The multinational and interdisciplinary comparative analysis of interventions will generate a data set on various interventions, their characteristics, functions, and use cases, which can support researchers or policy makers to make informed decisions for future DiPHIs planning and upscaling.

It is vital to acknowledge that this scoping review, while intended to illustrate the distinctive characteristics of DiPHI, does not seek to provide exhaustive analyses on cost-effectiveness, efficacy, or barriers and facilitators of implementation. The inherent heterogeneity of DiPHI renders it impractical to address these multifaceted aspects within a single literature review [13,14].

## Methods

Following the PRISMA-ScR (Preferred Reporting Items for Systematic Reviews and Meta-Analyses extension for Scoping Reviews) guidelines, we registered our review protocol with the PROSPERO database (CRD42021265562) on August 2, 2021, and details and rationale of the protocol were published in 2022 [34].

### Eligibility Criteria

The standards for eligibility were constructed in the participants, intervention, and study design format. An overview of the inclusion and exclusion criteria is presented in Textbox 1. Accompanying these criteria, the general requirements of full-text accessibility and the publication language were also considered. The review expanded the publication language limitation to English, German, and Chinese based on the language skills of the reviewer team. Given the scope of our study, which focuses on mapping the characteristics of DiPHI rather than comparing the effectiveness or costs, the review process refrains from specifically incorporating reported outcome variables.

**Eligibility criteria for the scoping review.**

**Inclusion criteria**

- Population
  The study focuses on the community level or above (ie, regional or national) of the general population.
- Intervention
  The paper describes a concrete digital public health intervention (DiPHI) that addresses at least 1 essential public health function through digital means as defined by the World Health Organization (WHO).
  The DiPHI matches our definition of digital public health.
  The DiPHI addresses at least 1 essential public health function as defined by the WHO.
  The DiPHI is paid or reimbursed by the government or health insurance.
  The DiPHI uses the internet or Bluetooth to provide its core function or service.
  The DiPHI is the central research object of the paper.
- Study design
  All original peer-reviewed studies, reports, books, book chapters, or peer-reviewed conference papers that have a description of a DiPHI as their primary intervention component.
- Full text
  Full text was available.

**Exclusion criteria**

- Population
  The study population consists of veterans, armed forces, prisoners, inmates, refugees, or asylum seekers.
- Intervention
  The intervention needs to be privately bought by the user without reimbursement by the government or health insurance.
  The intervention focuses on background management processes.
- Study design
  Study protocols, editorials, commentaries, conference proceedings, or reviews (ie, narrative reviews, scoping reviews, systematic reviews, or meta-analyses).
- Full text
  Full text was not available.

### Literature Search

The initial search followed a comprehensive search strategy conducted on February 19, 2021, in CENTRAL, PubMed, and Web of Science. We ran an additional search following the same search strategy on December 1, 2021, for 2 additional databases: ACM Full-Text Collection and IEEE Xplore. The search results for all 5 databases were updated on October 5, 2022. We applied a consistent search string to all databases with adaption according to index and syntax specifications differences. Given the substantial volume of publications aligning with our inclusion criteria, supplementary manual searches were not undertaken. However, we conducted reference list searches for reviews and meta-analyses describing DiPHIs. No additional references were identified through this approach, as all references were already identified through our systematic search.

**Figure 1:**
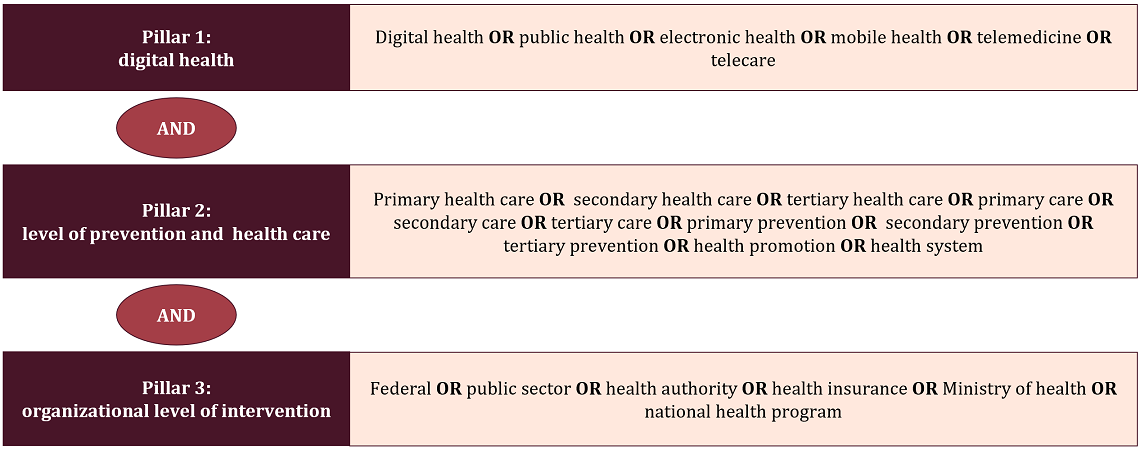
The fundamental concept of the search strategy.

The search strategy for all databases was structured around 3 themes connected through “AND.” Terms within the themes are combined through “OR” (refer to Figure 1 for the general overview and Multimedia Appendix 1 for the complete search strategy for all databases, including results per term of the initial search). We decided against searching for concrete interventions such as EHRs, specific health or medical apps, or other interventions to reduce the risk of confirmation bias.

We additionally used Medical Subject Headings wherever appropriate during database searches. All other terms were limited to title, abstract, or keywords. No search limits were applied.

### Screening Process

**Figure 2:**
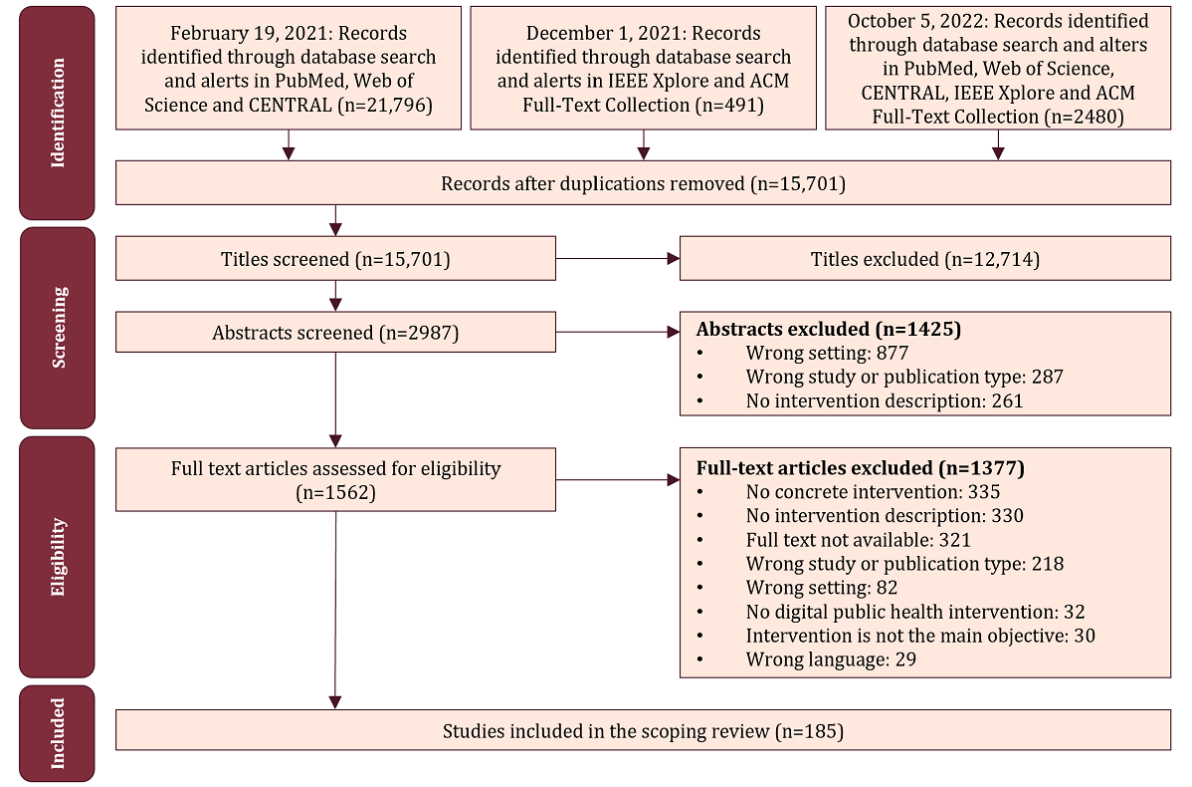
Flowchart of the search and screening process.

We applied a 3-stage screening strategy (ie, titles, abstracts, and full texts) to identify suitable publications (Figure 2). To reduce the list of reasons for full-text exclusion, the first 3 exclusion criteria mentioned in Textbox 1 were summarized under “wrong setting.” Three authors (LM, MF, and KA) were involved in the screening process for eligibility, and each stage had 2 authors partake in the screening independently. After each screening stage, another author (CCP) resolved any disagreements on study selection with independent decisions. The screening was conducted through the free web and mobile app Rayyan (Rayyan Systems Inc) [35]. References published in another language than English, Chinese, or German were excluded during full-text screening. Chinese references were screened only by CCP. Cohen κ for full-text screening was 0.77 (identifying a substantial agreement rate according to the study by Landis and Koch [36]). Full texts were included if the article was published open access, if it was published in a subscription journal licensed by our institution, or if the corresponding author provided a copy. An overview of all excluded full texts, along with the reasons for their exclusion, is included in Multimedia Appendix 2 [37-39].

### Data Extraction and Qualitative Content Analysis

Two authors (LM and KA) independently extracted data in an Excel 2019 spreadsheet (Microsoft Corp). The extraction sheet was piloted with the first 10 included publications. The extraction for all data except for the technical functions and nontechnical intervention characteristics (which were extracted as free texts) followed a predefined coding table, which was extended if necessary (Table 1). The extraction for all data was based on qualitative description directly provided by the included texts. Discrepancies in data extraction were resolved through discussion between the 2 authors. Missing data for the included publications or data where only a vaguely formulated description was given in the text were requested from authors via email. Where multiple interventions were described within 1 publication, data for each intervention was extracted in a new row. Target groups were defined and extracted based on the digital health application directory by Lantzsch et al [37] due to their precise differentiation between healthy users and patient users. Additional target groups (eg, policy makers, health professionals, or researchers) were added in an iterative process. The digital health functions and the intervention types followed the 2019 edition of the NICE ESF for digital health technologies [33]. The most updated ESF from 2022 reduced its number of individual technical functions and was changed toward a more clinical setting. However, the 2019 framework still included 10 different functions, for example, intervention types covering health promotion, health care, communication, education, and system services. Therefore, we deemed the 2019 version more suitable for our mapping approach. In addition, these functions were supported by the work of Lantzsch et al [37]. The public health functions were based on the 11 WHO functions [27]. Building on our previous research, ESF and ESPHF are merged to map DiPH for this study [29]. Extracting authors selected the option “other” where multiple target groups, functions, or prevention or health care levels appealed.

Target groups were defined and extracted based on the digital health application directory by Lantzsch et al [37] due to their precise differentiation between healthy users and patient users. Extracting authors selected the option “other” where multiple functions or target groups appealed. This decision displays the only deviation from the registered study protocol for this review [34] where we did not define different categories within target groups (the study by Lantzsch et al [37] was published after our study protocol, which underlines the rapidly evolving nature of DiPH). The definitions for prevention and health care (ie, last column in Table 2) were based on the WHO definitions (Table 2).

In addition to the previously mentioned topics, we also extracted information on the study design, publication year and type, intervention name, technical features, and nontechnical characteristics. We did not extract data on outcome measures or type of data collection in the included publications because this review aims to conduct an intervention mapping focusing on their characteristics and not on their effectiveness or costs.

**Table 1:** Coding table for data extraction.

Study type	Country	Type of intervention	Implementation status	Primary target group	Digital health function	Essential public health function	Level of prevention, health care, and research
Case report	Afghanistan	Health or medical app	Proposed intervention	Health insurances	Active monitoring	Governance	Primary prevention
Case-control study	Albania	Disease surveillance system	Planned intervention	Health professionals	Simple monitoring	Financing	Secondary prevention
Longitudinal study	Algeria	Early warning software	Pilot study	Researchers	Calculate	Human resources	Tertiary prevention
Cross-sectional study	Andorra	Electronic health record	Locally implemented	Policy makers	Communicate	Health information system	Primary health care
Economical study	Angola	Electronic consultation	Regionally implemented	Healthy without known risk factors	Diagnose	Research	Secondary health care
Mixed methods study	Antigua and Barbuda	Electronic medication plan	Nationally implemented	Healthy with known risk factors	Inform	Social participation and health communication	Tertiary health care
Trial or Experimental study	Argentina	Electronic prescribing	Internationally implemented	Acute ill not life threatening	Preventative behavior change	Health promotion	Research
Qualitative study	Armenia	Electronic referral	Website	Chronically ill stable	Self-manage	Health protection	—
—^a^	Austria	Electronic disease registry	—	Highly vulnerable or unstable health status	System service	Disease prevention	—
—	Azerbaijan	Electronic vaccination registry	—	Disaster management professionals	Treatment	Preparedness for public health emergencies	—
—	Bahamas	Health information system	—	—	—	health care	—
—	Bahrain	Implants	—	—	—	—	—
—	Bangladesh	Information website	—	—	—	—	—
—	Barbados	Patient portal	—	—	—	—	—
—	Belarus	Patient to provider communication portal	—	—	—	—	—
—	Belgium	Provider to provider communication portal	—	—	—	—	—
—	Belize	Telemedicine	—	—	—	—	—
—	Benin	Wearable	—	—	—	—	—

^a^Not applicable.

**Table 2:** Applied definitions for prevention and health care.

Level of prevention or health care	Definition	Source
Primary prevention	Avoiding new diseases, disabilities, or injuries	WHO^a^ [40] and Baumann and Ylinen [41]
Secondary prevention	Early detection of diseases, disabilities, or injuries	WHO [40,42] and Baumann and Ylinen [41]
Tertiary prevention	Reducing complication risk of existing diseases, disabilities, or injuries	Baumann and Ylinen [41]
Primary health care	Care for acute mild illness, injuries, or medical problems at a primary care provider with a whole-of-society approach (eg, through a general practitioner)	WHO [40,42], Hopayian [43], and Bodenheimer and Grumbach [44]
Secondary health care	Specialist or emergency medical care through specialized physicians	Hopayian [43] and Bodenheimer and Grumbach [44]
Tertiary health care	Highly specialized medical health care over an extended period in stationary settings, usually in referral hospitals	Hopayian [43] and Bodenheimer and Grumbach [44]

^a^WHO: World Health Organization.

For the geographic analysis of interventions, we counted every intervention multiple times if it was implemented internationally. For instance, Adler et al [45] described an intervention that was implemented in 7 countries. Weighting these interventions per country by the number of countries they were implemented in (here 1/7) would have weakened internationally planned or implemented interventions compared to interventions only targeting 1 country. Therefore, we decided to give full weight to each intervention and count the intervention as 1 for every targeted country. While this approach resulted in an artificially higher number of included interventions, it also ensured that international interventions were not discriminated against based on the geographic analysis.

For the qualitative analysis, we grouped the identified references by intervention types based on our study protocol. We then summarized the extracted data for each intervention type, as illustrated in Table 2. Additional intervention types were added in an inductive procedure where needed. In addition, we developed an inductive category system based on 5 randomly selected publications per intervention type to describe the technical functions and nontechnical characteristics of different intervention types. The qualitative content analysis through iterative coding was then conducted for all references of 1 intervention type in MAXQDA 2022.7 (VERBI GmbH) by LM. The procedure followed the inductive content analysis procedure described by Vears and Gillam [46].

## Results

### Overview

In total, we identified 15,701 different publications published until October 5, 2022 (24,767 before deduplication), through our systematic search in 5 scientific databases. Of these, 2987 (19.02%) were assessed for abstract screening and 1609 (10.25%) for full-text screening. The process excluded 1414 records, primarily due to unavailable full-text content and a lack of specificity in describing interventions. Of the screened full texts, the remaining 11.5% (185/1609) were considered eligible for this review. Some of these publications described multiple interventions, whereas, in other cases, the same intervention was presented by >1 publication. Of the 185 publications, 96 (51.9%) were case reports that focused on a detailed intervention design or implementation description, while the aim of the remaining 89 (48.1%) publications lay on the intervention evaluation, most often evaluated through cross-sectional studies (n=37, 20%), trials (n=19, 10.3%), or longitudinal studies (n=18, 9.7%). The characteristics and extracted information of all included 185 references are displayed in Multimedia Appendix 3 [38,45,47-229] instead of the main manuscript. As sometimes multiple references are reported on the same intervention, this scoping review mapped the characteristics of 179 different DiPHI for the included 185 publications.

### Distribution of Interventions Globally

**Figure 3:**
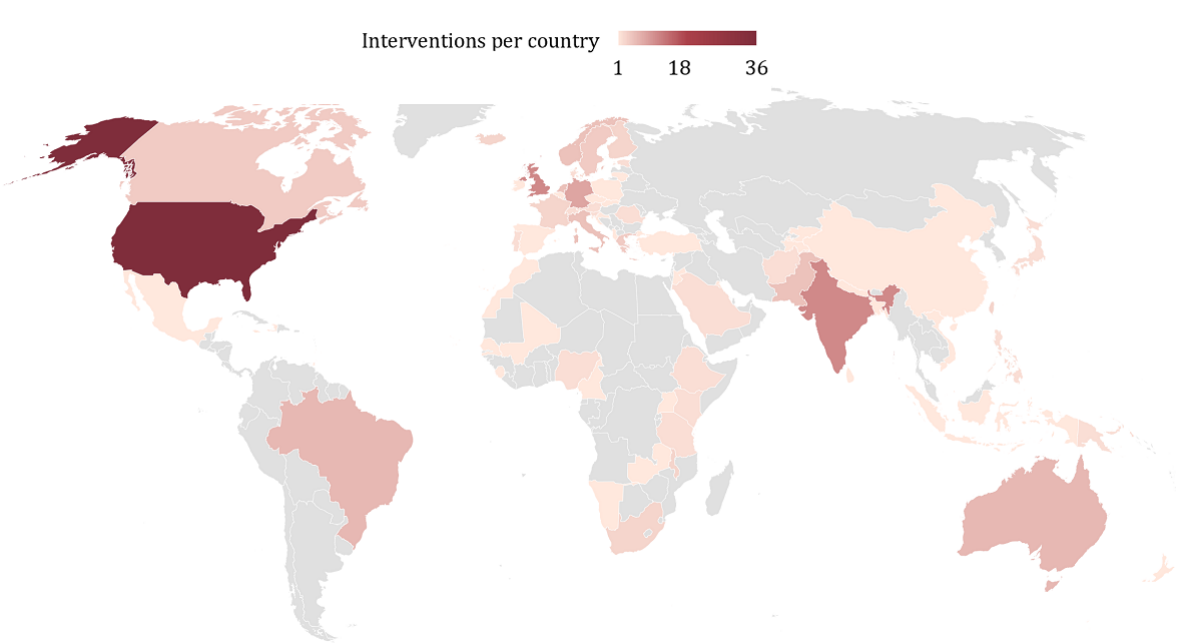
World map for the number of included interventions by country, continent, and high-income to low-income countries.

Figure 3 displays the distribution of the included interventions by country. For this analysis, all interventions were weighted equally regardless of the number of countries they were implemented in. As several interventions were implemented in >1 country, this analysis artificially increased the number of observed interventions to 199. Although most interventions (36/199, 18.1%) were from the United States, continent -wise, Europe took the lead with 38.2% (76/199) reported interventions. Ranked third was Asia with 17.6% (35/199), followed by Africa (26/199, 13.1%), Oceania and Australia (9/199, 4.5%), and South America (6/199, 3%). From a gross domestic product point of view (ie, grouped in high, middle, or low-income countries as classified by the World Bank [230]), most interventions were planned for or implemented in high-income countries (129/199, 64.8%) and were set-up in middle-income countries (58/199, 29.1%). Of the 10 interventions in low-income countries, 3 (30%) targeted Malawi [73-76,82,218], and 2 (20%) each were applied in Afghanistan and Ethiopia [73-76,91,175,222]. Furthermore, of the 10 interventions, 1 (10%) each was planned or implemented in Mali, Sierra Leone, and Uganda [56,111,190].

### Intervention Types and Implementation Status at the Time of Publication

**Figure 4:**
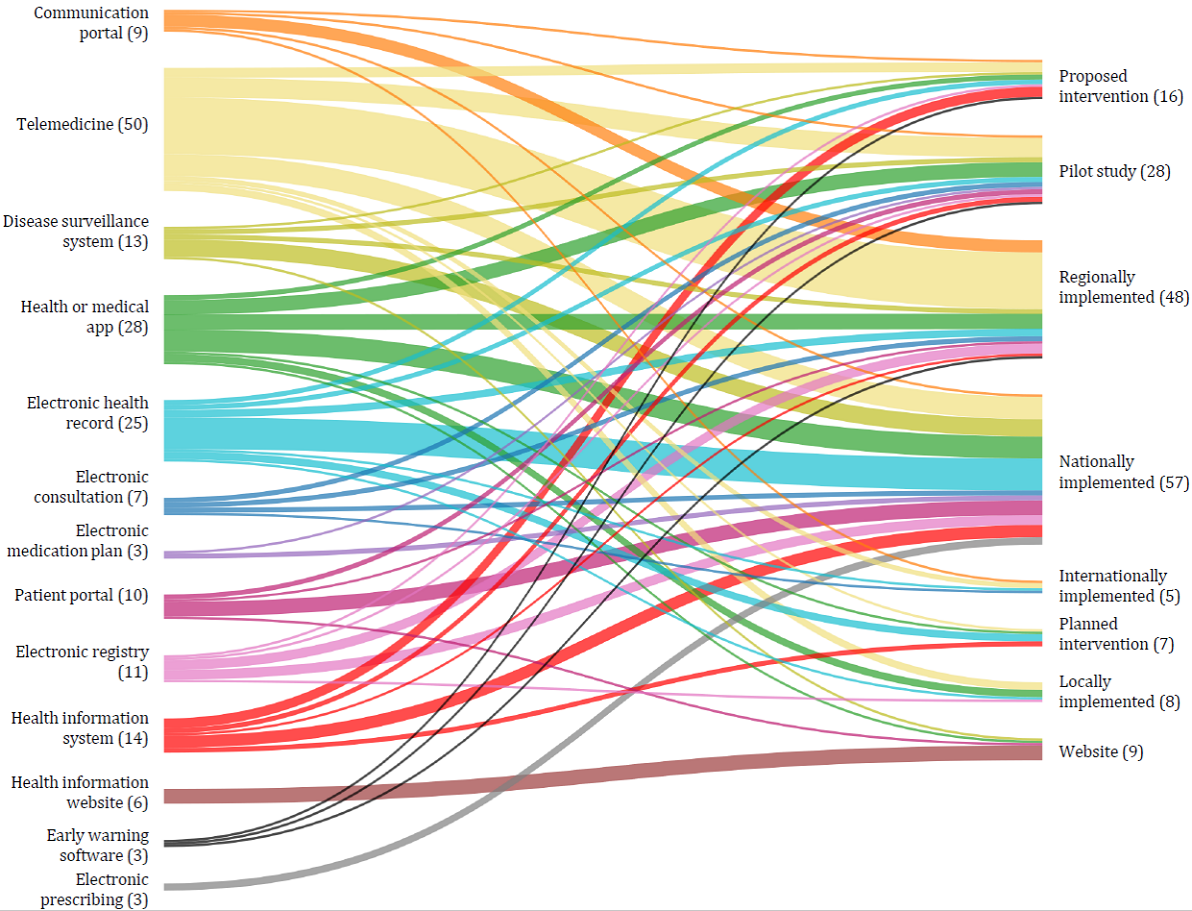
Overview of intervention types and implementation status. Some interventions were described during earlier phases and then again in later periods. Therefore, they are listed once for every reported intervention life cycle in this figure.

Under the classification of intervention by NICE ESF, most interventions (49/179, 27.4%) were centered on telemedical services, including telestroke, telecare, and telemonitoring. According to Timpel et al [231], these interventions are characterized using ICT to cover a geographic distance in health care delivery from a health professional to a patient or group of patients. The second most frequently counted interventions were health apps and medical apps (28/179, 15.6%), followed by EHR (23/179, 12.8%). The complete overview of intervention types is displayed in Figure 4, together with their implementation status at the reported time from the included references. As 4 publications reported on the same intervention but at different time phases, all publications were included for this display (eg, Austria’s national EHR *ELGA* was only planned when Ströher and Honekamp [199] reported about it, but was already nationally implemented when Herbek et al [113] and Schaller et al [177] described the platform). This results in a total of 181 displayed interventions. Interventions in >1 country were categorized as internationally implemented.

### Target Population and Intervention Setting

Our analysis showed that DiPHIs tend to focus on an average of 3 distinct groups within the population. These include health professionals, policy makers, public insurance executives, researchers, disaster management professionals, patients, and healthy individuals. Unsurprisingly, health professionals were the most often targeted group, with 140 (78%) of 179 interventions. They were followed by patients who were chronically ill but stable (101/179, 56.4%). Of all interventions, 51.9% (93/179) were aimed at healthy people with or without risk factors for certain health conditions.

**Figure 5:**
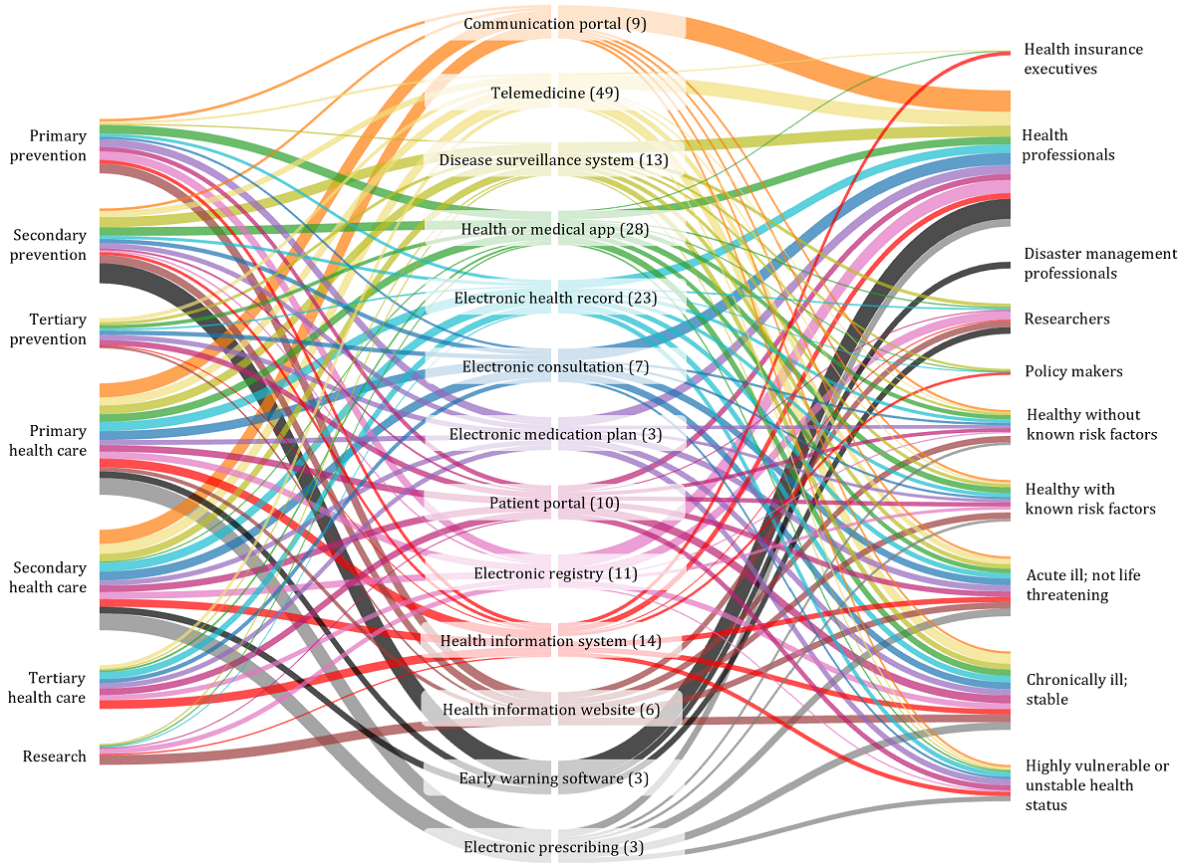
Addressed target groups and level of prevention, health care, or research in relative distribution per intervention type.

Parallel to the observation of the target population, each intervention affected, on average, 2 different settings, that is, level of prevention, health care, or research as defined previously. Most interventions were health care applications (ie, 100/179, 55.9% for primary, 89/179, 49.7% for secondary, and 55/179, 30.7% for tertiary health care). Out of the 179 interventions, while 16 (8.9%) interventions included a research component [57,84,93,104,128,139,154,163,180,220,221,225], only 3 (1.7%) focused exclusively on research: the Lone Star Stroke Consortium Telestroke Registry, the SAI Databank for electronic health research and evaluation, and the German COVID-19 data donation app (ie, Corona Datenspende) [57,93,128]. For prevention, of the 179 interventions, 13 (7%) exclusively targeted primary prevention (ie, through vaccination registries, behavior change programs, or health education in healthy individuals) [55,62,96,98,101,106,126,140,143,188, 208,223,226]. Of the 179 interventions, 8 (4%) focused only on secondary prevention, as digital screening and surveillance programs [48,61,153,161,171,194,201,219], and 3 (2%) improved the health of patients who were chronically ill on a tertiary prevention level [103,147,202]. In contrast, of all included 179 interventions, 10 (6%) were established for primary health care purposes only [49,67,79,109,124,172,178, 186,210,212,228,229], 17 (9%) were designed for secondary health care through specialists or emergency use cases [52,54,60,65,78,83,88,95,134,135,173-175,179,189,193,216], and 5 (3%) aimed at tertiary health care in hospital settings [70,109,132,157,200]. Figure 5 gives an overview of the relative size of each target group and the addressed level of prevention, health care, or research per intervention setting.

### The Intersection of Digital Health Technologies and Essential Public Health Functions in DiPHIs

**Figure 6:**
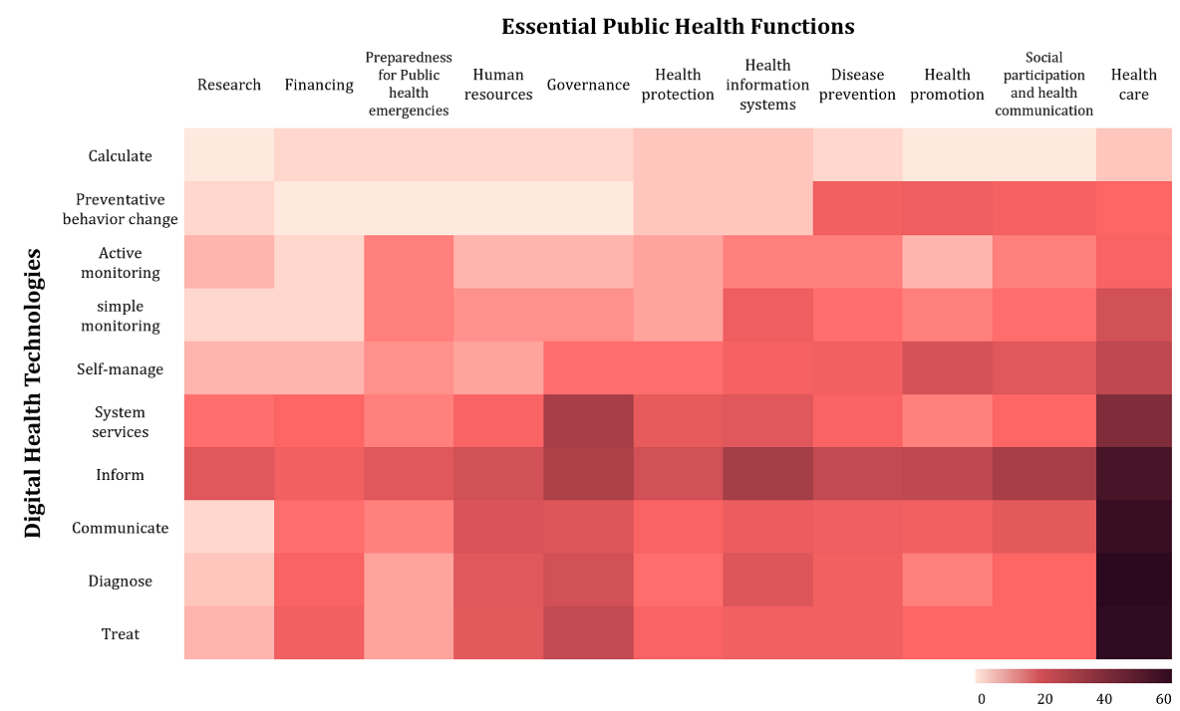
Heat map of the essential public health functions and digital health technologies to map digital public health interventions.

The analysis of the distribution of EPHFs and digital health technology functions matches the aforementioned focus on health care in DiPHIs. The heat map, displayed in Figure 6, highlights that most interventions were found in the cross-section of health care and diagnostic (64/179, 35.8%), treatment (63/179, 35.2%), communication (61/179, 34.1%), and information (57/179, 31.8%) or as system services in health care (42/179, 23.5%). Relatively few interventions, in contrast, were used for calculation (11/179, 6.1%), preventive behavior change (40/179, 22.3%), or public health research (48/179, 26.8%). A subgroup analysis for all intervention types with at least 20 included references is included in Multimedia Appendix 4. This analysis covers telemedicine, EHR, and health or medical apps, offering an overview of the diversity of topics addressed even within the same intervention category.

### Technological Functions and Nontechnical Intervention Characteristics

Given the sample size, we only provide details on the technical functions and nontechnical characteristics of telemedical interventions and EHRs. We chose not to include health apps or medical apps in this analysis due to the considerable heterogeneity in the wide range of purposes and functions of these apps, as discussed in a previous article [232].

#### Telemedicine

According to a review by Sood et al [233], which included 104 publications, telemedicine “...uses communications networks for delivery of health care services and medical education from one geographical location to another, primarily to address challenges like uneven distribution and shortage of infrastructural and human resources.” The category system includes 5 mayor categories and subcategories for which we assessed all 49 telemedical interventions included in our review [45,47,50,51,60,61,63,65,66,71,85,92,100,101,109,110,115,116,118, 119,124,129,134,141,142,145,146,149,151,155,156,159,164,165,174, 176,178,179,184,189,193,200,204,206,209-212,227]. The results are displayed in Table 3. Each intervention may exhibit a combination of these features, showcasing how telemedicine is applied in different settings. The most dominant characteristic for included interventions was that the software program was supported by hardware to facilitate clinical care. Besides computers and webcams, this included high-quality cameras, sensors (eg, for blood pressure or blood sugar), ultrasounds, or digital stethoscopes. Nearly half of the interventions (25/49, 51%) described how they used the internet or satellite connections to process and store data. The share of interventions that included a provider-to-provider communication option was slightly lower (17/49, 34%) than those that allowed patient-to-provider communication (23/49, 47%). Nevertheless, 20 (41%) of 49 interventions were characterized by their focus on individual patients, sometimes even through personalized intervention interfaces. However, telemedical interventions may not only focus on clinical care. As stated in the definition, they are also applied to the education context (ie, for patients and health care workers), health promotion, and improving self-efficacy in patients who were chronically ill.

In summary, telemedical interventions exhibit core characteristics that include the use of supporting hardware during consultations, data storage through databases, internet connectivity, bidirectional communication between users, educational modules, user interfaces, and the ability to plan and adjust clinical care plans when needed.

**Table 3:** Technical functions and nontechnical characteristics of the telemedical interventions (n=49).

Category		Interventions, n (%)	References
**Technical infrastructure**			
	Software supported through hardware tools	41 (84)	[45,47,50,60,61,63,65,66,71,85,92,100,101,109,115,116,118,124,129,134,141, 145,146,151,155,156,159,164,174,176,178,184,189,193, 200,204,209-212,227]^a-c^
	Data acquisition, transmission, and storage in a database	33 (67)	[45,50,51,60,61,63,65,71,85,109,110,116,118,119,124,134,135,145,146,151,155,156,164, 174,176, 179,184,189,193,200,206,210]^a^
	Internet network and connectivity	25 (51)	[47,50,60,61,71,85,100,101,115,118,124,146,151,159,174,178,179,184,189,193,200,206, 209,210,212]
	Web-based user interfaces	22 (45)	[47,50,51,60,65,85,92,101,110,119,145,149,151,156,159,174,178,184,200,206,210,212]
	Data security procedures	18 (37)	[45,47,60,61,66,85,100,101,149,155,159,174,179,189,193,204,206,211,212,227]^a^
	Centralized server application	16 (33)	[47,50,51,61,85,92,100,109,110,116,119,142,155,156,176,210]^b^
	Multiaccess, multiprofile web application	16 (33)	[60,66,85,92,100,101,110,118,135,151,156,174,178,184,204,211,212,227]^a^
	Clinician modules and units	14 (29)	[47,50,51,60,66,92,100,116,124,145,176,189,193,209,211,227]^a^
	Patient modules and units	6 (12)	[85,92,109,179,209,210]^b^
**Patient-provider interaction**			
	Bidirectional communication (patient-provider)	23 (47)	[60,63,92,100,109,116,119,124,135,142,145,146,149,151,156,174,176,178,184,189, 200,204,212]^c^
	Clinical care treatment planning and adjustments	22 (45)	[51,60,61,63,66,85,92,100,109,110,116,118,142,146,149,151,176,189,193,200,204,211,227]^a-c^
	Bidirectional communication (provider-provider)	17 (35)	[50,71,85,92,116,118,119,124,129,134,145,149,156,176,189,193,210]
	Remote monitoring of vital signs and clinical status through monitoring kits and sensors	17 (35)	[50,71,85,92,116,118,119,124,129,134,145,149,156,176,189,193,210]
	Compliance and adherence monitoring	11 (22)	[92,100,116,118,124,145,146,149,178,189,204]
	Custom and predefined questionnaires	6 (12)	[85,116,145,156,176,204]
**User-centered features**			
	Patient-centered interactions possible	20 (41)	[45,51,60,85,92,109,110,142,145,149,151,155,156,159,164,165,174,204,212]^b,c^
	Self-monitoring and tracking	10 (20)	[92,101,116,119,124,145,149,156,176,210]
	Personalization (eg, avatars, screen names, communication preferences, reminders, and alerts)	8 (16)	[65,85,92,101,149,151,204,210]
	Alarm detection and notification	6 (12)	[85,92,116,119,184,206]
**Use of patient data**			
	Data interpretation and analysis	12 (24)	[50,51,60,61,63,65,85,92,100,145,151,156]
	Data visualization tool	13 (27)	[45,50,51,66,85,92,101,110,151,176,204,209-211,227]^a^
	Data- and guideline-driven decision support	11 (22)	[50,66,71,85,92,145,149,151,156,176,193,211,227]^a^
**Support and engagement**			
	Education and counseling modules	22 (45)	[45,51,71,92,101,109,116,118,134,135,145,146,149,156,159,164,165,178,189, 200,204,206]^c^
	Counseling and motivation module	10 (20)	[45,92,116,118,141,146,149,155,189,204]
	Patient empowerment and self-efficacy	5 (10)	[85,141,145,146,189]

^a^3 studies [66,211,227] described the same intervention.

^b^Intervention 1 by Hartvigsen et al [109].

^c^Intervention 2 by Hartvigsen et al [109].

#### EHRs

Following the broadly accepted definition by the International Organization for Standardization (2019), an EHR is “a data repository regarding the health and health care of a subject of care where all information is stored on electronic media” [234]. According to this terminology, we developed a category system with 4 major topics and subcategories, which was applied to the 26 publications that described 23 different EHR systems [67,69,81,83,87,89-91,104,105,107,113,114,123,125,162,166,177, 181,182,185,195,199,205,215,221,224]. The results are displayed in Table 4. Nearly all interventions (18/23, 78%) allowed health care professionals access to the EHR after the patient consented or an active relationship (eg, due to treatment of chronic diseases) existed between the 2 parties. Although not as often described, most EHR systems (14/23, 61%) stated that patients were able to access their data. Data sharing was possible in 61% (14/23) of the interventions, and another 61% (14/23) described data protective procedures for accessing and sharing data. Although fewer times, interoperability with other health care systems was described (11/23, 48%). We observed differences regarding the stored and accessible data. Unsurprisingly, nearly all interventions (21/23, 91%) reported on storing health and clinical data. However, only 30% (7/23) of the interventions included personal data on the record holder or their immunization records. Only 17% (4/23) of the interventions allowed clinicians to upload their notes to the system.

**Table 4:** Technical functions and nontechnical characteristics of the electronic health records (EHRs; n=23).

Category		Interventions, n (%)	References
**Technical infrastructure**			
	Integration and interoperability with external health care systems	11 (48)	[69,87,91,104,107,113,123,162,177,181,195,199,205,215,221]^a,b^
	Mobile platform	7 (30)	[87,90,113,166,177,185,199,205,221]^b,c^
	Centralized server	6 (26)	[81,104,113,114,162,177,182,195,199,215]^a,b^
	Communication protocols	6 (26)	[69,81,113,177,181,182,199,221]^b,c^
	Cloud-based system	4 (17)	[87,90,185,205,221]^c^
	Data synchronization	3 (13)	[81,185,205,221]
**Data transfer and privacy**			
	Data sharing possible	14 (61)	[67,69,81,87,91,104,105,107,113,123,162,177,181,182,199,215,221]^a-c^
	Data security protocols embedded	14 (61)	[69,87,89,90,104,107,113,123,125,162,166,177,181,195,199,205,215,221]^a-c^
	Opt-out approach	4 (17)	[67,104,113,162,177,199,215,221]^a-c^
	Opt-in approach	4 (17)	[69,83,182,205,221]^c^
**Stored data**			
	Medical history (eg, diagnoses, medical procedures, laboratory results, and treatments)	21 (91)	[69,81,83,87,89,91,104,105,107,113,114,123,125,162,166,177,181,182,185,195,199, 205,215,221,224]^a-c^
	Medication plan	14 (61)	[69,83,89,91,105,107,113,114,123,166,177,181,182,199,205,221]^b,c^
	Patient demographic data	7 (30)	[69,89,91,113,125,166,177,195,199]^b^
	Immunization records	7 (30)	[69,91,105,125,185,195,205,221]^c^
	Financial and administrative data related to health care services	5 (22)	[91,114,205,221,224]^c^
	Clinician notes	4 (17)	[105,107,224]
**EHR system features and modules**			
	Health professional access to patient data	18 (78)	[67,69,87,89,104,105,107,113,123,125,162,166,177,181,182,185,195,199,205,215,221,224]^a-c^
	Patient access to data	14 (61)	[69,87,89-91,104,105,113,162,166,177,181,182,199,205,215,221,224]^a-c^
	User interfaces	11 (48)	[69,87,89,90,113,123,166,177,181,185,195,199,205,221]^b,c^
	Clinical case–finding features	8 (35)	[67,104,107,113,114,123,162,177,181,195,199,215]^a,b^
	Patient management modules	3 (13)	[114,166,195]
	Data used for real-time surveillance and monitoring	3 (13)	[81,87,195]

^a^3 studies [104,162,215] described the same intervention.

^b^3 studies [113,177,199] described the same intervention.

^c^2 studies [205,221] described the same intervention.

## Discussion

### Principal Findings

Our scoping review has identified a substantial number of publications, underscoring the substantial interest and activity in DiPH. The wealth of publications illustrates the broad spectrum of interventions being explored and implemented worldwide. This diversity reflects the evolving landscape of health care delivery and disease prevention in the digital age.

The distribution of DiPHIs across countries and continents showcases the worldwide interest in leveraging technology to enhance health care but also points out significant differences in implementation adaptation between high-income and low-income countries. A reason for this imbalance negatively impacting the global digital divide might lie in the inequality regarding the digital advance among developed countries compared to developing countries [235,236]. The prevalence of interventions in high-income countries may partly be attributed to more significant resources and infrastructure [237]. This divide is highlighted by a lack of funding, inadequate computer availability, and limited internet skills that limit the expansion and applicability of ICT and digital (public) health [238]. Still, interventions in low-income countries signal the potential for digital technologies to bridge health care gaps in resource-constrained settings and aging societies [237]. Indeed, the Global Digital Health Monitor covers our findings by displaying overall scores for countries regarding their digital health maturity. Developing countries (especially in Africa) rank lower than their developed counterparts in Europe or North America [239]. More rigorous public health research and practice efforts are needed to empower developing countries to apply digital tools for health-related purposes. The WHO’s Digital Health Atlas can serve as such an effort where project leads can voluntarily register their digital health intervention free of charge [240]. Maps such as these will support public health policy, research, and practice in better resource allocation (eg, the map can display already existing subnational initiatives that could be scaled up to the national or international level without creating double-infrastructures), benchmarking between interventions and further developing intervention types. However, the explicit distinction between medically focused digital health interventions and population-centric DiPHIs will be needed for future versions of the atlas.

Our analysis shows that DiPHIs often target multiple population groups and settings. Health professionals are a primary target, reflecting technology integration into health care practice and education. The emphasis on patients who are chronically ill but stable underscores the potential of digital interventions to improve care for this population. Moreover, interventions in diverse prevention, health care, and research settings illustrate their versatility across the health care continuum. This observation was supported by the heat- map representation of EPHFs and ESFs, highlighting a significant concentration of digital interventions in health care–related functions such as diagnostics, treatment, communication, and information. This reflects the predominant role of digital interventions in enhancing clinical care and health care management. However, there is room for further exploration of interventions addressing prevention and public health research functions.

Health apps, telemedical services, and EHRs emerged as the dominant intervention types in our review. Health apps are well known for their heterogeneity [232], which provides the potential to address all areas of public health, including health promotion, clinical care, and rehabilitation [241]. Telemedical services stood out as a substantial focus of our included references, aligning with their capacity to extend universal health coverage reach across geographic boundaries and increase health care delivery speed while lowering treatment costs and response times to control disease outbreaks [242]. EHRs, in contrast, can be essential tools for public health surveillance in providing data on the population’s health status and the health care system’s performance [39,114,243]. These findings highlight the multifaceted nature of DiPHIs and their adaptability to various health care needs and contexts in public health.

Overall, the analysis underscores the heterogeneity within DiPHIs, even within the same intervention category, as we have displayed through a qualitative analysis of included publications for telemedical interventions and EHRs. This diversity necessitates precise definitions and standardized terminologies to facilitate effective communication, collaboration, and evaluation. Clear definitions can also aid in benchmarking and comparing interventions across different health care systems. The detailed analysis of technological functions and nontechnical characteristics of both intervention types revealed the complexity and variation within each intervention type. Identifying core characteristics and added features can guide intervention development and enhance our understanding of their capabilities. In 2019, WHO published their recommendations on digital interventions for the clinical setting, which provides a definition for each intervention (eg, client-to-provider telemedicine), synonyms for these tools, and recommendations regarding their use cases [244]. While this overview serves as a good starting point, it requires extensions for nonclinical settings and an overview of core functions and technical features to allow benchmarking. The heterogeneity in DiPHI forces us to rethink how national health system assessments should be conducted to allow for comparable results. Instead of asking, *Does your country have an EHR system?”* it is critical to ask for the availability of an intervention with specific characteristics: “*Does your country have an intervention with the following characteristics, targeting these population groups, and being applied for at least one of the following settings?”*

As the field continues to evolve, it is essential to prioritize rigorous research and evaluation to assess these interventions’ effectiveness, safety, and impact on population health and health care accessibility. This review serves as a valuable resource for policy makers, researchers, and health care practitioners seeking to navigate the dynamic and diverse field of DiPHIs.

### Strengths and Limitations

Originally conceived as a systematic review, our research protocol was registered with PROSPERO to conduct a comprehensive synthesis of the existing literature on DiPHIs. The systematic approach is typically characterized by a rigorous process involving reviewing evidence to answer narrowly defined research questions. However, as our study progressed, we recognized that our research question and the evolving DiPH landscape were better suited for a scoping review methodology. While systematic reviews focus on specific research questions and outcomes, the strengths of scoping reviews for effectively mapping and characterizing interdisciplinary and comprehensive topics (such as the diverse spectrum of DiPHIs in our case, including their characteristics, settings, and target populations) [245] were more applicable for our study aim. This change allowed us to adapt to the dynamic nature of the field. It provided a broader perspective essential for understanding the multifaceted nature of digital interventions. This transition underscores the importance of selecting the most suitable methodology to achieve comprehensive insights into the subject matter. The selection of our information sources demonstrated the interdisciplinarity of DiPH by combining literature databases from public health and computer science. This rigorous strategy mitigates the risk of missing essential publications. The broader inclusion of publications from 3 languages (ie, English, German, and Chinese) is another strength of our study, given the rising number of publications on digital health and DiPH from German-speaking and Chinese-speaking countries. Throughout the process, a minimum of 2 researchers independently screened all references, adhering to the 4-eye principle. Any disagreements in decisions regarding inclusion or exclusion were resolved by another author (CCP) to ensure the reliability of the results. This study followed the PRISMA-ScR [245] to provide high-quality, transparent research results. The filled checklist is provided in Multimedia Appendix 5.

Nevertheless, while this scoping review exhibits numerous strengths, it also presents certain limitations. We did not create publication alerts for the selected databases, limiting our results to references published before October 6, 2022. However, we conducted manual reference list checks for reviews and meta-analyses to identify additional publications that were potentially missed in our search. As no further relevant findings were added from the manual search, we are confident that our scoping review draws a holistic picture of the landscape of DiPHIs globally. Furthermore, we are convinced that the included references are sufficient for an initial intervention mapping of DiPHIs. Nonetheless, it is worth noting that certain intervention types might be underrepresented, such as early warning software, electronic medication plans, electronic prescribing, or emerging intervention types that were not yet reported during our search (eg, artificial intelligence tools). Here, we invite other researchers to build upon our first results and continue the mapping process. Following the PRISMA-ScR [245], assessing the quality of the included publications was not applicable, which could lead to questions regarding the reporting accuracy of the included publications. Nevertheless, as we focused solely on the functions and use cases of the interventions and not on their outcomes, this limitation remains minor.

Furthermore, the challenge of intervention specificity emerged as a notable consideration. The varying levels of detail in the descriptions of digital interventions in the literature posed difficulties in discerning their precise nature. This lack of specificity hindered the ability to categorize and analyze interventions effectively, as it often left critical questions unanswered. For instance, understanding the core technological functions, nontechnical attributes, determined use cases, and defined user groups of a given digital intervention is essential for its accurate classification and evaluation. Although in our case, data extraction was conducted by 2 authors (LM and KA) independently and authors were contacted in case of uncertainty, the absence of such information generally can impede efforts to compare interventions, assess their impact, and make informed decisions regarding their implementation or scalability. This is why we advocate for clearer reporting standards for digital health and DiPH interventions. Finally, we must point out that although the volume of included publications was substantial, a distinct portion could not be included in our review due to factors such as unavailable full-text content and a lack of specificity in intervention descriptions. This highlights challenges in accessing relevant literature and emphasizes the need for more detailed reporting in published studies, particularly regarding the specifics of digital interventions. In an era where timely access to information is critical, the inaccessibility of full-text articles poses an obstacle to researchers and stakeholders seeking to explore and build upon existing knowledge in DiPHIs.

### Further Research

Due to the heterogeneity of DiPHIs, it was not feasible for this scoping review to report detailed information for every intervention type. Therefore, we encourage other researchers to uptake our methodology, conduct reviews on specific intervention types, and refine our initial intervention mapping results. More critically, a shared and multidisciplinary understanding of key terminology in digital health and DiPH is needed. Consequently, consensus finding methods (eg, Delphi approaches) need to be applied to internationally create a shared understanding of DiPHI.

In addition, assessments of the sustainability of DiPHIs are crucial to map the implementation status. As Iwelunmor et al [246] and Kaboré et al [247] already highlighted, the sustainability of DiPHIs is an essential component of the whole life cycle of implemented interventions. Therefore, it is crucial to consider whether an intervention has been sustainably implemented or stopped after the initial pilot funding phase has ended. Further research should be conducted to analyze, through a longitudinal design or pre-post comparison, how sustainably DiPHIs are implemented and whether differences between intervention types exist.

### Conclusions

This scoping review has shown that DiPHIs are distinctly diverse in their use cases between individual intervention groups and within such groups. Therefore, using precise terminology when planning, implementing, or evaluating DiPHIs is crucial. For instance, instead of phrasing an intervention as an EHR, one should ask for specific technological functions and nontechnical characteristics, determine use cases (from a digital health and public health perspective), and define user groups. This approach facilitates cross-disciplinary collaboration in intervention development and research and fosters international benchmarks and comparability across global DiPH systems. These insights offer valuable guidance for policy makers, researchers, and health care practitioners interested in using the potential of digital interventions to improve population health and health care accessibility.

## Appendix

Multimedia Appendix 1 Complete search strategies for the PubMed, Web of Science, CENTRAL, IEEE Xplore, and ACM Full-Text Collection databases.

Multimedia Appendix 2 Publications excluded during full-text screening.

Multimedia Appendix 3 Overview of the included interventions.

Multimedia Appendix 4 Heat maps of the selected intervention types.

Multimedia Appendix 5 PRISMA-ScR (Preferred Reporting Items for Systematic Reviews and Meta-Analyses extension for Scoping Reviews) checklist for this manuscript.

## Abbreviations

DiPH: digital public health
DiPHI: digital public health intervention
EHR: electronic health record
ESF: evidence standards framework
ICT: information and communication technology
NICE: National Institute for Health and Care Excellence
PRISMA-ScR: Preferred Reporting Items for Systematic Reviews and Meta-Analyses extension for Scoping Reviews
WHO: World Health Organization

## Data Availability

The data sets generated and analyzed during this study are available from the corresponding author on reasonable request.
