# Supplementary material for: Mapping Digital Public Health Interventions Among Existing Digital Technologies and Internet-Based Interventions to Maintain and Improve Population Health in Practice: Scoping Review
Source: J Med Internet Res. 2024 Jul 17;26:e53927. doi: 10.2196/53927 (PMC11292160; doi:10.2196/53927)
Supplement: Multimedia Appendix 1 [file jmir_v26i1e53927_app1.pdf]

Maaß L, Angoumis K, Freye M, Pan CC. Mapping Digital Public Health Interventions Among Existing Digital Technologies and Internet-Based Interventions to Maintain and Improve Population Health in Practice: Scoping Review. Journal of Medical Internet Research 2024; 26: e53927. DOI: 10.2196/53927.

Multimedia Appendix 1: Complete Search Strategies PubMed, Web of Science, CENTRAL, Ieee Xplore, and ACM Full-Text Collection

No	Medline (PubMed) (19th Feb 2021 12:40 PM CET)		Web of Science (19th Feb 2021 12:40 PM CET)		CENTRAL (19th Feb 2021 12:40 PM CET)		Ieee Xplore (1st Dec 2021 2:00 PM CET)		ACM Full-Text Collection (1st Dec 2021 2:00 PM CET)	
	**Search Term**	**Results**	**Search Term**	**Results**	**Search Term**	**Results**	**Search Term**	**Results**	**Search Term**	**Results**
#1	“digital health” [Title/Abstract]	2.683	TS=("digital health")	2.999	(“digital health”):ti,ab	260	"Document Title":"digital health" OR "Abstract":"digital health"	207	Title:("digital health") OR Abstract:("digital health")	251
#2	“digital public health” [Title/Abstract]	20	TS=("digital public health")	29	(“digital public health”):ti,ab	0	"Document Title":"digital public health" OR "Abstract":"digital public health"	1	Title:("digital public health") OR Abstract:("digital public health")	43
#3	“health 2.0“ [Title/Abstract]	69	TS=("health 2.0")	129	(“health 2.0”):ti,ab	3	"Document Title":"health 2.0" OR "Abstract":"health 2.0"	13	Title:("health 2.0") OR Abstract:("health 2.0")	10
#4	Mhealth [Title/Abstract]	5.895	TS=(mhealth)	6.732	(mhealth):ti,ab	1.266	"Document Title":mhealth OR "Abstract": mhealth	571	Title:(mhealth) OR Abstract:(mhealth)	359
#5	m-health [Title/Abstract]	641	TS=(m-health)	1.285	(m-health):ti,ab	1.255	"Document Title":"m health" OR "Abstract":"m health"	330	Title:("m health") OR Abstract:("m health")	35
#6	“m health” [Title/Abstract]	641	TS=("m health")	1.285	(“m health”):ti,ab	1.718	"Document Title":"mobile health" OR "Abstract":"mobile health"	815	Title:("mobile health") OR Abstract:("mobile health")	250
#7	“mobile health”[Title/Abstract]	5.197	TS=("mobile health")	6.655	(“mobile health”):ti,ab	1.177	"Document Title":ehealth OR "Abstract": ehealth	760	Title:(ehealth) OR Abstract:(ehealth)	122
#8	mobile-health [Title/Abstract]	5.197	TS=(ehealth)	6.583	(mobile-health):ti,ab	1.177	"Document Title":"e health" OR "Abstract":"e health"	1.830	Title:("e health") OR Abstract:("e health")	218
#9	Ehealth[Title/Abstract]	6.560	TS=(e-health)	7.517	(ehealth):ti,ab	955	"Document Title":"electronic health" OR "Abstract": "electronic health"	1.867	Title:("electronic health") OR Abstract:("electronic health")	463

No	Medline (PubMed) (19th Feb 2021 12:40 PM CET)		Web of Science (19th Feb 2021 12:40 PM CET)		CENTRAL (19th Feb 2021 12:40 PM CET)		Ieee Xplore (1st Dec 2021 2:00 PM CET)		ACM Full-Text Collection (1st Dec 2021 2:00 PM CET)	
	**Search Term**	**Results**	**Search Term**	**Results**	**Search Term**	**Results**	**Search Term**	**Results**	**Search Term**	**Results**
#10	e-health [Title/Abstract]	3.306	TS=("e health")	7.517	(e-health):ti,ab	967	"Document Title":uhealth OR "Abstract": uhealth	3	Title:(uhealth) OR Abstract:(uhealth)	0
#11	"e health" [Title/Abstract]	3.306	TS=("electronic health")	22.517	("e health"):ti,ab	1.105	"Document Title":"u health" OR "Abstract":"u health"	67	Title:("u health") OR Abstract:("u health")	6
#12	"electronic health" [Title/Abstract]	20.372	TS=(uhealth)	23	("electronic health"):ti,ab	1.911	"Document Title":"ubiquitous health" OR "Abstract":"ubiquitous health"	113	Title:("ubiquitous health") OR Abstract:("ubiquitous health")	20
#13	uhealth[Title/Abstract]	34	TS=(u-health)	170	(uhealth):ti,ab	7	"Document Title":Telemedicine OR "Abstract": Telemedicine	2.728	Title:(Telemedic*) OR Abstract:(Telemedic*)	152
#14	u-health [Title/Abstract]	32	TS=("ubiquitous health")	253	(u-health):ti,ab	9	"Document Title":Telecare OR "Abstract": Telecare	251	Title:(Telecar*) OR Abstract:(Telecar*)	38
#15	"u health" [Title/Abstract]	32	TS=(telemedicine)	23.838	("u health"):ti,ab	9	"Document Title":telehealth OR "Abstract": telehealth	603	Title:(telehealth) OR Abstract:(telehealth)	52
#16	"ubiquitous health" [Title/Abstract]	80	TS=(telecare)	1.745	("ubiquitous health"):ti,ab	2	"Document Title":telemonitoring OR "Abstract": telemonitoring	432	Title:(telemonitor*) OR Abstract:(telemonitor*)	21
#17	Telemedicine [Title/Abstract]	15.682	TS=(telehealth)	8.869	(Telemedicine):ti,ab	3.858	"Document Title":"Remote Consultation" OR "Abstract":"Remote Consultation"	34	Title:("Remote Consultation") OR Abstract:("Remote Consultation")	5
#18	Telecare [Title/Abstract]	817	TS=(telemonitoring)	2.570	(Telecare):ti,ab	182	"Document Title":"Distance Counseling" OR "Abstract":"Distance Counseling"	1	Title:("Distance Counseling") OR Abstract:("Distance Counseling")	0
#19	Telehealth [Title/Abstract]	6.827	TS=(telecommunication*)	58.182	(telehealth):ti,ab	1.865	**{OR #1-18}**	**9.245**	**{OR #1-#18}**	**1.659**

No	Medline (PubMed) (19th Feb 2021 12:40 PM CET)		Web of Science (19th Feb 2021 12:40 PM CET)		CENTRAL (19th Feb 2021 12:40 PM CET)		Ieee Xplore (1st Dec 2021 2:00 PM CET)		ACM Full-Text Collection (1st Dec 2021 2:00 PM CET)	
	**Search Term**	**Results**	**Search Term**	**Results**	**Search Term**	**Results**	**Search Term**	**Results**	**Search Term**	**Results**
#20	Telemonitoring [Title/Abstract]	1.812	TS=("remote consultation")	510	(telemonitoring):ti,ab	1.016	"Document Title":"health care" OR "Abstract":"health care"	8.578	Title:("health care") OR Abstract:("health care")	1.602
#21	telecommunication* [Title/Abstract]	4.580	TS=("distance counseling")	29	(telecommunication*):ti,ab	534	"Document Title":healthcare OR "Abstract":healthcare	18.968	Title:("healthcare") OR Abstract:("healthcare")	4.409
#22	"telemedicine"[Mesh]	32.661	**{OR #1-#21}**	**131.880**	MeSH descriptor: [Telemedicine] explode all trees	2.649	"Document Title":"health system" OR "Abstract":"health system"	711	Title:("health system") OR Abstract:("health system")	130
#23	"Remote Consultation"[Mesh]	5.102	TS=("health care")	406.041	MeSH descriptor: [Remote Consultation] explode all trees	391	"Document Title":"health systems" OR "Abstract":"health systems"	633	Title:("health systems") OR Abstract:("health systems")	166
#24	"Distance Counseling"[Mesh]	66	TS=(healthcare)	257.720	MeSH descriptor: [Distance Counseling] explode all trees	20	"Document Title":"primary care" OR "Abstract":"primary care"	335	Title:("primary care") OR Abstract:("primary care")	70
#25	**{OR #1-#24}**	**74.185**	TS=(health-care)	406.162	**{OR #1-#24}**	**11.317**	"Document Title":"secondary care" OR "Abstract":"secondary care"	13	Title:("secondary care") OR Abstract:("secondary care")	6
#26	"health care" [Title/Abstract]	391.538	TS=("health system")	41.073	("health care"):ti,ab	58.949	"Document Title":"tertiary care" OR "Abstract":"tertiary care"	53	Title:("tertiary care") OR Abstract:("tertiary care")	6
#27	"healthcare" [Title/Abstract]	263.891	TS=("primary care")	147.519	("healthcare"):ti,ab	21.011	"Document Title":prevent OR "Abstract":prevent	62.222	Title:(prevent*) OR Abstract:(prevent*)	11.634
#28	"health-care" [Title/Abstract]	391.538	TS=("secondary care")	7.055	("health-care"):ti,ab	58.949	"Document Title":"Health Promotion" OR "Abstract":"Health Promotion"	173	Title:("health promotion") OR Abstract:("health promotion")	68

No	Medline (PubMed) (19th Feb 2021 12:40 PM CET)		Web of Science (19th Feb 2021 12:40 PM CET)		CENTRAL (19th Feb 2021 12:40 PM CET)		Ieee Xplore (1st Dec 2021 2:00 PM CET)		ACM Full-Text Collection (1st Dec 2021 2:00 PM CET)	
	**Search Term**	**Results**	**Search Term**	**Results**	**Search Term**	**Results**	**Search Term**	**Results**	**Search Term**	**Results**
#29	“health system“ [Title/Abstract]	41.299	TS=("tertiary care")	54.229	(“health system”):ti,ab	2.65	"Document Title":"Health Information System" OR "Abstract":"Health Information System"	178	Title:("Health Information System") OR Abstract:("Health Information System")	31
#30	“health systems“ [Title/Abstract]	21.008	TS=(prevent*)	1.879.428	(“health systems”):ti,ab	922	"Document Title":"Health Information Systems" OR "Abstract":"Health Information Systems"	226	Title:("Health Information Systems") OR Abstract:("Health Information Systems")	73
#31	“primary care“ [Title/Abstract]	122.013	TS=("health promotion")	42.355	(“primary care”):ti,ab	18.563	**{OR #20-30}**	**88.754**	**{OR #20-#30}**	**17.309**
#32	“secondary care“ [Title/Abstract]	7.031	TS=("health information system*")	4.692	(“secondary care”):ti,ab	1.3	"Document Title": government OR "Abstract":government	27.706	Title:(government*) OR Abstract:(government*)	7.893
#33	“tertiary care“ [Title/Abstract]	51.085	**{OR #23-#33}**	**2.601.278**	(“tertiary care”):ti,ab	6.329	"Document Title":federal OR "Abstract":federal	6.055	Title:(federal*) OR Abstract:(federal*)	1.305
#34	prevent* [Title/Abstract]	1.503.970	TS=(government*)	500.697	(prevent*):ti,ab	231.425	"Document Title": national OR "Abstract": national	45.812	Title:(national*) OR Abstract:(national*)	10.207
#35	“health promotion“ [Title/Abstract]	34.692	TS=(federal*)	161.659	(“health promotion”):ti,ab	9.876	"Document Title":"ministry of health" OR "Abstract":"ministry of health"	221	Title:("ministry of health") OR Abstract:("ministry of health")	26
#36	“Delivery of Health Care“[Mesh]	1.110.486	TS=(national*)	1.209.471	MeSH descriptor: [Delivery of Health Care] explode all trees	46.211	"Document Title":"health ministry" OR "Abstract":"health ministry"	26	Title:("health ministry") OR Abstract:("health ministry")	5.474
#37	“Health Information Systems“[Mesh]	1.370	TS=(ministr*)	55.649	MeSH descriptor: [Health Information Systems] explode all trees	12	"Document Title":"institute of health" OR "Abstract":"institute of health"	48	Title:("institute of health") OR Abstract:("institute of health")	6
#38	“Primary Health Care“[Mesh]	165.523	TS=(institute*)	550.479	MeSH descriptor: [Primary Health Care] explode all trees	7.352	"Document Title":"health institute" OR "Abstract":"health institute"	11	Title:("health institute") OR Abstract:("health institute")	3

Maaß L, Angoumis K, Freye M, Pan CC. Mapping Digital Public Health Interventions Among Existing Digital Technologies and Internet-Based Interventions to Maintain and Improve Population Health in Practice: Scoping Review. Journal of Medical Internet Research 2024; 26: e53927. DOI: 10.2196/53927.

No	Medline (PubMed) (19th Feb 2021 12:40 PM CET)		Web of Science (19th Feb 2021 12:40 PM CET)		CENTRAL (19th Feb 2021 12:40 PM CET)		Ieee Xplore (1st Dec 2021 2:00 PM CET)		ACM Full-Text Collection (1st Dec 2021 2:00 PM CET)	
	**Search Term**	**Results**	**Search Term**	**Results**	**Search Term**	**Results**	**Search Term**	**Results**	**Search Term**	**Results**
#39	“Secondary Care“[Mesh]	689	TS=(department*)	405.918	MeSH descriptor: [Secondary Care] explode all trees	39	"Document Title":"health institutes" OR "Abstract":"health institutes"	11	Title:("health institutes") OR Abstract:("health institutes")	0
#40	“Tertiary Healthcare“[Mesh]	1.184	TS=("health agenc*")	5.229	MeSH descriptor: [Tertiary Healthcare] explode all trees	20	"Document Title":"department of health" OR "Abstract":"department of health"	140	Title:("department of health") OR Abstract:("department of health")	29
#41	“Primary Prevention“[Mesh]	155.766	TS=("health autorit*")	6	MeSH descriptor: [Primary Prevention] explode all trees	4.185	"Document Title":"health department" OR "Abstract":"health department"	46	Title:("health department") OR Abstract:("health department")	14
#42	“Secondary Prevention“[Mesh]	21.006	TS=("public sector*")	35.583	MeSH descriptor: [Secondary Prevention] explode all trees	3.179	"Document Title":"health departments" OR "Abstract":"health departments"	32	Title:("health departments") OR Abstract:("health departments")	8
#43	“Tertiary Prevention“[Mesh]	167	TS=("health insurance*")	45.429	MeSH descriptor: [Tertiary Prevention] explode all trees	4	"Document Title":"health agency" OR "Abstract":"health agency"	13	Title:("health agency") OR Abstract:("health agency")	4
#44	“Health Promotion“[Mesh]	78.808	TS=("ministr* of health")	15.080	MeSH descriptor: [Health Promotion] explode all trees	6.617	"Document Title":"health agencies" OR "Abstract":"health agencies"	38	Title:("health agencies") OR Abstract:("health agencies")	22
#45	**{OR #26-#44}**	**3.181.642**	TS=("health ministr*")	1.107	**{OR #26-#44}**	**329.463**	"Document Title":"health authority" OR "Abstract":"health authority"	31	Title:("health authority") OR Abstract:("health authority")	6
#46	government* [Title/Abstract]	114.494	TS=("institute* of health")	20.482	(government*):ti,ab	4.113	"Document Title":"health authorities" OR "Abstract":"health authorities"	113	Title:("health authorities") OR Abstract:("health authorities")	23

Maaß L, Angoumis K, Freye M, Pan CC. Mapping Digital Public Health Interventions Among Existing Digital Technologies and Internet-Based Interventions to Maintain and Improve Population Health in Practice: Scoping Review. Journal of Medical Internet Research 2024; 26: e53927. DOI: 10.2196/53927.

No	Medline (PubMed) (19th Feb 2021 12:40 PM CET)		Web of Science (19th Feb 2021 12:40 PM CET)		CENTRAL (19th Feb 2021 12:40 PM CET)		Ieee Xplore (1st Dec 2021 2:00 PM CET)		ACM Full-Text Collection (1st Dec 2021 2:00 PM CET)	
	**Search Term**	**Results**	**Search Term**	**Results**	**Search Term**	**Results**	**Search Term**	**Results**	**Search Term**	**Results**
#47	federal*[Title/Abstract]	54.103	TS=("health institute*")	1.090	(federal*):ti,ab	2.100	"Document Title":"health insurances" OR "Abstract":"health insurances"	8	Title:("health insurances") OR Abstract:("health insurances")	0
#48	national* [Title/Abstract]	563.338	TS=("department* of health")	9.083	(national*):ti,ab	36.376	"Document Title":"health insurance" OR "Abstract":"health insurance"	364	Title:("health insurance") OR Abstract:("health insurance")	96
#49	ministr* [Title/Abstract]	27.106	TS=("health department*")	7.787	(ministr*):ti,ab	2.201	"Document Title":"public sectors" OR "Abstract":"public sectors"	164	Title:("public sector") OR Abstract:("public sector")	426
#50	institute* [Title/Abstract]	181.003	TS=("national health program*")	541	(institute*):ti,ab	20.196	"Document Title":"public sector" OR "Abstract":"public sector"	966	Title:("public sectors") OR Abstract:("public sectors")	45
#51	department* [Title/Abstract]	334.254	TS=("health plan*")	12.753	(department*):ti,ab	34.228				
#52	"health agency" [Title/Abstract]	2.085	TS=(insurance*)	133.747	("health agency"):ti,ab	84				
#53	"health agencies" [Title/Abstract]	4.947	TS=(reimbursement)	22.550	("health agencies"):ti,ab	141				
#54	"health authority" [Title/Abstract]	3.766			("health authority"):ti,ab	269				
#55	"health authorities" [Title/Abstract]	9.730			("health authorities"):ti,ab	370				
#56	"public sector" [Title/Abstract]	7.328			("public sector"):ti,ab	371				
#57	"public sectors" [Title/Abstract]	521			("public sectors"):ti,ab	9				
#58	"health insurance" [Title/Abstract]	44.314			("health insurance"):ti,ab	2.311				
#59	"health insurances" [Title/Abstract]	455			("health insurances"):ti,ab	47				
#60	"ministry of health" [Title/Abstract]	16.651	**{OR #34-#52}**	**2.697.509**	("ministry of health"):ti,ab	1553	**{OR #32-#50}**	**76.007**	**{OR #32-50}**	**17.520**

No	Medline (PubMed) (19th Feb 2021 12:40 PM CET)		Web of Science (19th Feb 2021 12:40 PM CET)		CENTRAL (19th Feb 2021 12:40 PM CET)		Ieee Xplore (1st Dec 2021 2:00 PM CET)		ACM Full-Text Collection (1st Dec 2021 2:00 PM CET)	
	Search Term	Results	Search Term	Results	Search Term	Results	Search Term	Results	Search Term	Results
#61	"health ministry" [Title/Abstract]	1.029			("health ministry"):ti,ab	98				
#62	"institute of health"[Title/Abstract]	4.778			("institute of health"):ti,ab	1.040				
#63	"health institute" [Title/Abstract]	939			("health institute"):ti,ab	61				
#64	"department of health" [Title/Abstract]	13.171			("department of health"):ti,ab	673				
#65	"health department" [Title/Abstract]	5.963			("health department*"):ti,ab	298				
#66	"Government"[Mesh]	150.148			MeSH descriptor: [Federal Government] explode all trees	919				
#67	"Federal Government" [Mesh]	115.676			MeSH descriptor: [Government] explode all trees	983				
#68	"Government Agencies"[Mesh]	128.758			MeSH descriptor: [Government Agencies] explode all trees	955				
#69	"State Government" [Mesh]	11.937			MeSH descriptor: [State Government] explode all trees	6				
#70	"Local Government" [Mesh]	3.408			MeSH descriptor: [Local Government] explode all trees	14				
#71	"Public Sector"[Mesh]	6.629			MeSH descriptor: [Public Sector] explode all trees	58				
#72	"National Health Programs"[Mesh]	92.339			MeSH descriptor: [National Health Programs] explode all trees	571				
#73	"Regional Health Planning"[Mesh]	40.074			MeSH descriptor: [Regional Health Planning] explode all trees	275				
#74	"Insurance, Health"[Mesh]	148.570			MeSH descriptor: [Insurance, Health] explode all trees	1.123				

Maaß L, Angoumis K, Freye M, Pan CC. Mapping Digital Public Health Interventions Among Existing Digital Technologies and Internet-Based Interventions to Maintain and Improve Population Health in Practice: Scoping Review. Journal of Medical Internet Research 2024; 26: e53927. DOI: 10.2196/53927.

No	Medline (PubMed) (19th Feb 2021 12:40 PM CET)		Web of Science (19th Feb 2021 12:40 PM CET)		CENTRAL (19th Feb 2021 12:40 PM CET)		Ieee Xplore (1st Dec 2021 2:00 PM CET)		ACM Full-Text Collection (1st Dec 2021 2:00 PM CET)	
	**Search Term**	**Results**	**Search Term**	**Results**	**Search Term**	**Results**	**Search Term**	**Results**	**Search Term**	**Results**
#75	"Insurance, Health, Reimbursement"[Mesh]	46.123			MeSH descriptor: [Insurance, Health, Reimbursement] explode all trees	290				
#76	**{OR #46-#75}**	**1.454.483**			**{OR #46-#71}**	**86.069**				
#77	**#25 AND #45 AND #76**	**12.086**	**#22 AND #33 AND #54**	**8.227**	**#25 AND #45 AND #72**	**1.483**	**#19 AND #31 AND #51**	**418**	**#19 AND #31 AND #51**	**73**
