# Supplementary material for: Mapping Digital Public Health Interventions Among Existing Digital Technologies and Internet-Based Interventions to Maintain and Improve Population Health in Practice: Scoping Review
Source: J Med Internet Res. 2024 Jul 17;26:e53927. doi: 10.2196/53927 (PMC11292160; doi:10.2196/53927)
Supplement: Multimedia Appendix 2 [file jmir_v26i1e53927_app2.pdf]

Multimedia Appendix 2: Publications excluded during full-text screening

Publications without full text

1. Aarnio E, Raitoharju R. Patient's Medication Information and e-Health Development in Finland: A Case Study of a Finnish Primary Care Organization. Remenyi D, editor. Nr Reading: Acad Conferences Ltd; 2008. 1-7 p. ISBN: 978-1-906638-20-7.
2. Adetiba E, Eleanya M, Fatumo SA, Matthews VO. National Hospital Management Portal (NHMP): a framework for e-health implementation. Int J Electron Healthc. 2009;5(3):273-83. PMID: 20643641. doi: 10.1504/ijeh.2010.034176.
3. Adewale OS. An internet-based telemedicine system in Nigeria. International Journal of Information Management. 2004 Jun;24(3):221-34. PMID: WOS:000221484800003. doi: 10.1016/j.ijinfomgt.2003.12.014.
4. Adler K, Harper R, Hoyt R. Bridging the gap. Electronic health information exchanges could eliminate the silos of information and improve care. Med Econ. 2010 Dec 17;87(24):52-6. PMID: 21341614.
5. Adler-Milstein J, Furukawa MF, King J, Jha AK. Early results from the hospital Electronic Health Record Incentive Programs. Am J Manag Care. 2013 Jul 1;19(7):e273-84. PMID: 23919447.
6. Adubato S. The Changing World of Healthcare. MD Advis. 2019 Fall;12(4):31-2. PMID: 32501662.
7. Afridi MI. Tele-mental and behavioural health: implications in glocal context - a hope to meet the needs of the underserved. J Pak Med Assoc. 2013 Jun;63(6):668-9. PMID: 23901661.
8. Ahwidy M, Pemberton L. What Changes Need to be Made within the LNHS for Ehealth Systems to be Successfully Implemented? Rocker C, Ziefle M, Odonoghue J, Maciaszek L, Molloy W, editors. Setubal: Scitepress; 2016. 71-9 p. ISBN: 978-989-758-180-9.
9. Al-Jafar E. Exploring patient satisfaction before and after electronic health record (EHR) implementation: the Kuwait experience. Perspect Health Inf Manag. 2013;10(Spring):1c. PMID: 23805063.
10. Al-Qirim N, Insticc. Telemedicine adoption and diffusion: The case of the United Arab emirates. Setubal: Insticc-Inst Syst Technologies Information Control & Communication; 2006. 191-9 p. ISBN: 972-8865-62-7.
11. Al-Rawajfah O, Tubaishat A. Barriers and facilitators to using electronic healthcare records in Jordanian hospitals from the nurses' perspective: A national survey. Inform Health Soc Care. 2019 Jan;44(1):1-11. PMID: 28829654. doi: 10.1080/17538157.2017.1353998.
12. Alasheev AM, Hubert GJ, Santo GC, Vanhooren GT, Zvan B, Campos ST, et al. [Recommendations on telestroke in Europe]. Zh Nevrol Psikhiatr Im S S Korsakova. 2020;120(3. Vyp. 2):33-41. PMID: 32307428. doi: 10.17116/jnevro202012003233.

13. Albarrak AI, Mohammed R, Almarshoud N, Almujalli L, Aljaeed R, Altuwaijiri S, et al. Assessment of physician's knowledge, perception and willingness of telemedicine in Riyadh region, Saudi Arabia. J Infect Public Health. 2021 Jan;14(1):97-102. PMID: 31060975. doi: 10.1016/j.jiph.2019.04.006.

14. Alfaro M, Bonis J, Bravo R, Fluiters E, Minué S. [New technologies in primary care: people, machines, records, and networks. SESPAS Report 2012]. Gac Sanit. 2012 Mar;26 Suppl 1:107-12. PMID: 22336322. doi: 10.1016/j.gaceta.2011.12.005.

15. Alharbi A, Ramirez R, Assoc Informat S. Factors Affecting Electronic Health Record Adoption in Developing Countries: A Case of Saudi Arabia. Atlanta: Assoc Information Systems; 2020. ISBN: 978-1-7336325-4-6.

16. Ali A, Ehsan N, Mirza E. CONVENTIONAL MEDICAL CARE AND E-HOSPITAL APPLICATIONS IN HEALTHCARE SECTOR OF PAKISTAN. Chova LG, Belenguer DM, Torres IC, editors. Valenica: Iated-Int Assoc Technology Education & Development; 2010. 1513-8 p. ISBN: 978-84-613-5538-9.

17. Almaiman A, Bahkali S, Alfrih S, Househ M, El Metwally A. The Use of Health Information Technology in Saudi Primary Healthcare Centers. In: Mantas J, Househ MS, Hasman A, editors. Integrating Information Technology and Management for Quality of Care. Amsterdam: Ios Press; 2014. p. 209-12.

18. Almond H, Cummings E, Turner P. Australia's personally controlled electronic health record and primary healthcare: generating a framework for implementation and evaluation. Stud Health Technol Inform. 2013;188:1-6. PMID: 23823280.

19. Alsadi M, Saleh A, Khalil M, Oweidat I. Readiness-Based Implementation of Electronic Health Records: A Survey of Jordanian Nurses. Creat Nurs. 2022;28(1):42-7. PMID: WOS:000759636800007. doi: 10.1891/cn-2021-0024.

20. Alzoubaidi AR. Cloud Computing National e-health services: Data Center Solution Architecture. Int J Comput Sci Netw Secur. 2016 Sep;16(9):1-6. PMID: WOS:000389062700001.

21. Amato S, Di Giovanni C, Politi M, De Salazar V. [The telemedicine as tool of management on chronic disease: the experience of ASL ROMA 3 (Italian Local Authority). Impact, outcomes and results]. Ig Sanita Pubbl. 2019 May-Jun;75(3):231-44. PMID: 31645064.

22. Ambroise B, Benateau H, Garmi R, Hauchard K, Prevost R, Veyssiere A. The role of telemedicine in the management of maxillofacial trauma in emergency departments - preliminary results. Journal of Stomatology Oral and Maxillofacial Surgery. 2019 Apr;120(2):95-8. PMID: WOS:000462648400003. doi: 10.1016/j.jormas.2018.11.015.

23. Ammenwerth E, Duftschmid G, Gall W, Hackl WO, Hoerbst A, Janzek-Hawlat S, et al. A nationwide computerized patient medication history: Evaluation of the Austrian pilot project "e-Medikation". International Journal of Medical Informatics. 2014 Sep;83(9):655-69. PMID: WOS:000340262600005. doi: 10.1016/j.ijmedinf.2014.06.004.

24. Amuasi J, Agbogbatey MK, Sarfo FS, Beyuo A, Duah K, Agasiya P, et al. Feasibility, acceptability, and appropriateness of a mobile health stroke intervention among Ghanaian health workers. J Neurol Sci. 2022 Aug;439:5. PMID: WOS:000816993200008. doi: 10.1016/j.jns.2022.120304.

25. Amudha R, Motha LCS, Alamelu R, Nalini R, Srinivasan A. Telemedicine- A catalyst to health promotion. Research Journal of Pharmaceutical Biological and Chemical Sciences. 2017 Jan-Feb;8(1):323-8. PMID: WOS:000410595600042.

26. Anderson JG. Security of the distributed electronic patient record: a case-based approach to identifying policy issues. International Journal of Medical Informatics. 2000 Nov;60(2):111-8. PMID: WOS:000169986000006. doi: 10.1016/s1386-5056(00)00110-6.

27. Andrews L, Gajanayake R, Sahama T. The Australian general public's perceptions of having a personally controlled electronic health record (PCEHR). Int J Med Inform. 2014 Dec;83(12):889-900. PMID: 25200198. doi: 10.1016/j.ijmedinf.2014.08.002.

28. Angood PB. TELEHEALTH - HAS ITS TIME ARRIVED? Physician Leadersh J. 2015 May-Jun;2(3):6-7. PMID: 26214942.

29. Ardielli E. eHealth in the Czech Republic and Poland. Ardielli E, editor. Ostrava: Vsb-Tech Univ Ostrava; 2016. 9-16 p. ISBN: 978-80-248-3991-2.

30. Armani R, Mitchell LE, Allen-Graham J, Heriot NR, Kotsimbos T, Wilson JW. Current patient and healthcare worker attitudes to eHealth and the personally controlled electronic health record in major hospitals. Intern Med J. 2016 Jun;46(6):717-22. PMID: WOS:000380041600010. doi: 10.1111/imj.13086.

31. Arpad R. Information culture in healthcare, especially in the field of m-Health. Informacios Tarsadalom. 2012;12(1):108-+. PMID: WOS:000319234800007.

32. Artinian NT. Telehealth as a tool for enhancing care for patients with cardiovascular disease. J Cardiovasc Nurs. 2007 Jan-Feb;22(1):25-31. PMID: WOS:000243289500013. doi: 10.1097/00005082-200701000-00004.

33. Asbach P, Nerlich M. A telemedicine guideline for the practice of teleconsultation. In: Nerlich M, Schaechinger U, editors. Integration of Health Telematics into Medical Practice. Amsterdam: I O S Press; 2003. p. 1-14.

34. Asch DA. The hidden economics of telemedicine. Ann Intern Med. 2015 Nov 17;163(10):801-2. PMID: 26343261. doi: 10.7326/m15-1416.

35. Aston G. TELEHEALTH Reshaping Your World and Your Patients'. Hosp Health Netw. 2015 Mar;89(3):22-6. PMID: 30277330.

36. Auefuea S, Sillabutra J, Satitvipawee P, Nartthanarung A, Soontornpipit P. Development of electronic home health care record system on web applications. In: Siritaratiwat A, editor. 2016 International Electrical Engineering Congress, Ieecon2016. Amsterdam: Elsevier Science Bv; 2016. p. 204-7.

37. Bagayoko CO, Bediang G, Anne A, Niang M, Traore AK, Geissbuhler A. Digital health and the need to develop centers of expertise in sub-Saharan Africa : two examples in Mali and Cameroon. Medecine Et Sante Tropicales. 2017 Oct-Dec;27(4):348-53. PMID: WOS:000425236300004. doi: 10.1684/mst.2017.0726.

38. Bahkali S, Almaiman A, Almadani W, Househ M, El Metwally A. The State Public Health Informatics in Saudi Arabia. In: Mantas J, Househ MS, Hasman A, editors. Integrating Information Technology and Management for Quality of Care. Amsterdam: Ios Press; 2014. p. 257-60.

39. Baillie J. Distance no object for teleconsultations. Health Estate. 2008 Sep;62(8):67-9. PMID: 18822823.

40. Barlow J. Technology. Will the NHS show some love for remote care? Health Serv J. 2013 Jan 24;123(6336):23-5. PMID: 23488409.

41. Barr P. mEvidence, please. Mobile health tech is the rage, but does it work? Hosp Health Netw. 2013 Sep;87(9):22, 4. PMID: 24260962.

42. Barthold D, Chiguluri V, Gumpina R, Sweet CC, Pieratt J, Cordier T, et al. Health Care Utilization and Medical Cost Outcomes from a Digital Diabetes Prevention Program in a Medicare Advantage Population. Popul Health Manag. 2020 Dec;23(6):414-21. PMID: WOS:000586297400005. doi: 10.1089/pop.2019.0184.

43. Bashford C. Virtual care. How telemedicine is transforming the delivery of emergency medical services in the field. EMS World. 2011 Oct;40(10):31, 3-5. PMID: 22046896.

44. Bashshur R. Telemedicine and state-based licensure in the United States, revisited. Telemed J E Health. 2008 May;14(4):310-1. PMID: 18570558. doi: 10.1089/tmj.2008.9977.

45. Beatty Y. CURRENT TELEMEDICINE LANDSCAPE IN TENNESSEE. Tenn Med. 2015 Summer;108(4):23, 7. PMID: 26402936.

46. Beckley ET. Telecommunications. Rural areas poised to bolster telehealth. Hosp Health Netw. 2008 Feb;82(2):12, 4. PMID: 18330359.

47. Bennett K, Reynolds J, Christensen H, Griffiths KM. e-hub: an online self-help mental health service in the community. Med J Aust. 2010 Jun 7;192(S11):S48-52. PMID: 20528710. doi: 10.5694/j.1326-5377.2010.tb03694.x.

48. Beratarrechea A, Diez-Canseco F, Irazola V, Miranda J, Ramirez-Zea M, Rubinstein A. Use of m-Health Technology for Preventive Interventions to Tackle Cardiometabolic Conditions and Other Non-Communicable Diseases in Latin America- Challenges and Opportunities. Prog Cardiovasc Dis. 2016 May-Jun;58(6):661-73. PMID: WOS:000376552200011. doi: 10.1016/j.pcad.2016.03.003.

49. Bercovitz AR, [1-3]-Lee E, Jamoom E. Adoption and use of electronic health records and mobile technology by home health and hospice care agencies. Natl Health Stat Report. 2013 May 20(66):1-11. PMID: 24988819.

50. Berler A, Apostolakis I, Informat Resources Management A. Normalizing Cross-Border Healthcare in Europe via New E-Prescription Paradigms. Hersey: Igi Global; 2017. 31-72 p. ISBN: 978-1-5225-1763-4; 978-1-5225-1762-7.

51. Berlin J. The Tele-Future is Now: Will Telemedicine's Footprint Be Permanent Post-COVID-19? Tex Med. 2020 Jul 1;116(7):14-9. PMID: 32872705.

52. Bhavaraju SR. From subconscious to conscious to artificial intelligence: A focus on electronic health records. Neurol India. 2018 Sep-Oct;66(5):1270-5. PMID: WOS:000447605100005. doi: 10.4103/0028-3886.241377.

53. Bhuyan SS, Zhu H, Chandak A, Kim J, Stimpson JP. Do service innovations influence the adoption of electronic health records in long-term care organizations? Results from the US National Survey of Residential Care Facilities. International Journal of Medical Informatics. 2014 Dec;83(12):975-82. PMID: WOS:000346052600010. doi: 10.1016/j.ijmedinf.2014.09.007.

54. Bilimoria NM. Telemedicine 2.0: it's all about access and wellness. J Med Pract Manage. 2013 Mar-Apr;28(5):314-6. PMID: 23767129.

55. Bill G, Crisci CD, Canet T. [The Telehealth Network of the Americas and its role in primary health care]. Rev Panam Salud Publica. 2014 May-Jun;35(5-6):442-5. PMID: 25211574.

56. Blackledge C. Telehealth: Remote chance for virtual care. Health Serv J. 2011 Dec 1;121(6285):27-8. PMID: 22533021.

57. Blackman K. Telehealth and Licensing Interstate Providers. NCSL Legisbrief. 2016 Jul;24(25):1-2. PMID: 27825194.

58. Blackman K. Covering and Reimbursing Telehealth Services. NCSL Legisbrief. 2016 Jan;24(4):1-2. PMID: 27032126.

59. Bobbs M, Bayer M, Frazer T, Humphrey S, Wilson B, Olasz E, et al. Building a global teledermatology collaboration. Int J Dermatol. 2016 Apr;55(4):446-9. PMID: 26873427. doi: 10.1111/ijd.13223.

60. Bracale M, Cesarelli M, Bifulco P. Health technology assessment of telemedicine service in 'Islands Project'. Magjarevic R, Tonkovic S, Bilas V, Lackovic I, editors. Zagreb: Univ Zagreb; 2001. 90-3 p. ISBN: 953-184-023-7.

61. Brewer R, Goble G, Guy P. A peach of a telehealth program: Georgia connects rural communities to better healthcare. Perspect Health Inf Manag. 2011 Jan 1;8(Winter):1c. PMID: 21307986.

62. Brinn JE. Networking technology and health care in Eastern North Carolina. Conf Proc IEEE Eng Med Biol Soc. 2004;2004:3088. PMID: 17270931. doi: 10.1109/iembs.2004.1403872.

63. Bryce J. Telehealth connecting care. Aust Nurs J. 2011 Jun;18(11):23. PMID: 21739777.

64. Bursell SE, Zang S, Keech AC, Jenkins AJ. Evolving telehealth reimbursement in Australia. Intern Med J. 2016 Aug;46(8):977-81. PMID: 27553999. doi: 10.1111/imj.13150.

65. Butcher L. Technology. Telehealth programs spreading like 'wildfire'. Hosp Health Netw. 2011 Jun;85(6):17. PMID: 21790090.

66. Campion FX, Richter JM. High-level adoption of electronic health records. J Med Pract Manage. 2011 Jul-Aug;27(1):50-6. PMID: 21936433.

67. Cerda-Calafat I, Continente-Gonzalo M, Garcia-Lopez C, Guanyabens-Calvet J. Personal Health Folder. Med Clin (Barc). 2010 Feb;134:63-6. PMID: WOS:000277354300012. doi: 10.1016/s0025-7753(10)70012-9.

68. Cerdan J, Catalan-Matamoros D, Berg SW. Online communication in a rehabilitation setting: Experiences of patients with chronic conditions using a web portal in Denmark. Patient Educ Couns. 2017 Dec;100(12):2283-9. PMID: 28698033. doi: 10.1016/j.pec.2017.06.023.

69. Chalhoub-Jonsson C, Urosevic V, Kostic P, Devedzic V, Ciglenecki L, Slavec S. Introducing EHR in Serbia. Reichert A, Mihalas G, StoicusTivadar L, Schulz S, Engelbrecht R, editors. Berlin: Akademische Verlagsgesellsch Aka Gmbh; 2006. 398-404 p. ISBN: 978-3-89838-072-0.

70. Chang HL, Shaw MJ, Lai FP, Ko WJ, Ho YL, Chen HS, et al. U-Health: an example of a high-quality individualized healthcare service. Per Med. 2010 Nov;7(6):677-87. PMID: WOS:000285701800012. doi: 10.2217/pme.10.64.

71. Chong J, Singh M, Teoh SY. An Exploration of e-Health in the Public Sector: The Australian Perspective. Remenyi D, editor. Nr Reading: Acad Conferences Ltd; 2008. 99-107 p. ISBN: 978-1-906638-20-7.

72. Chronaki CE, Voss H, Santana S, Prokosch HU. eHealth Consumer Attitude on Online Access to Personal Health Records. In: Cunningham P, Cunningham M, editors. Expanding the Knowledge Economy: Issues, Applications, Case Studies, Pts 1 and 2. Amsterdam: Ios Press; 2007. p. 672-8.

73. Chuchvara N, Patel R, Srivastava R, Reilly C, Rao BK. The growth of teledermatology: Expanding to reach the underserved. J Am Acad Dermatol. 2020 Apr;82(4):1025-33. PMID: 31811880. doi: 10.1016/j.jaad.2019.11.055.

74. Cohen LT. Will the Federation of State Medical Boards compact ease the path to medical licensure and telemedicine? J Am Coll Radiol. 2015 Feb;12(2):132-3. PMID: 25652299. doi: 10.1016/j.jacr.2014.10.017.

75. Conn J. More telehealth ... beefed-up data security ... revised meaningful-use rules. Mod Healthc. 2016 Jan 4;46(1):22. PMID: 27086383.

76. Consorti F. Health record in the Age of e-Health. Clin Ter. 2011 Nov-Dec;162(6):585-9. PMID: WOS:000299587600014.

77. Cooney MA, Iademarco MF, Huang M, MacKenzie WR, Davidson AJ. The Public Health Community Platform, Electronic Case Reporting, and the Digital Bridge. J Public Health Manag Pract. 2018 Mar-Apr;24(2):185-9. PMID: WOS:000426552500018. doi: 10.1097/phh.0000000000000775.

78. Coulter KJ, Hintzsche MF. Establishing a Telehealth Program in Primary Care for the Treatment of Opioid Use Disorder. J Dr Nurs Pract. 2020 Nov 1;13(3):207-15. PMID: 33334926. doi: 10.1891/jdnp-d-19-00068.

79. Crane M. Exploring telehealth models. Med Econ. 2014 Jul 25;91(14):17-20. PMID: 25233550.

80. Crompton P, Motley R, Morris A. Teledermatology - the Cardiff experience. J Vis Commun Med. 2010 Dec;33(4):153-8. PMID: 21087184. doi: 10.3109/17453054.2010.525447.

81. Cross M. Reaching out to rural residents. Health Data Manag. 2007 Jun;15(6):62-3. PMID: 17580793.

82. Currie WL, Guah MW. IT-enabled healthcare delivery: The UK National Health Service. Information Systems Management. 2006 Spr;23(2):7-22. PMID: WOS:000236033900004. doi: 10.1201/1078.10580530/45925.23.2.20060301/92670.3.

83. Dacey B, Bholat MA. Health information technology: medical record documentation issues in the electronic era. Prim Care. 2012 Dec;39(4):633-42. PMID: 23148956. doi: 10.1016/j.pop.2012.08.001.

84. Dafoulas GE. Deploying Integrated Homecare Telemedicine Services in Central Greece. In: Stone CS, editor. Benchmarking Telemedicine: Improving Health Security in the Balkans. Amsterdam: Ios Press; 2017. p. 65-71.

85. Daley S. Riding the technological wave. Health Manag Technol. 2007 Nov;28(11):18, 20. PMID: 18019862.

86. Dandachi D, Lee C, Morgan RO, Tavakoli-Tabasi S, Giordano TP, Rodriguez-Barradas MC. Integration of telehealth services in the healthcare system: with emphasis on the experience of patients living with HIV. J Investig Med. 2019 Jun;67(5):815-20. PMID: WOS:000471864900123. doi: 10.1136/jim-2018-000872.

87. Daniel H, Sulmasy LS, American Coll P. Policy Recommendations to Guide the Use of Telemedicine in Primary Care Settings: An American College of Physicians Position Paper. Ann Intern Med. 2015 Nov;163(10):787-+. PMID: WOS:000365746400021. doi: 10.7326/m15-0498.

88. Danovaro-Holliday MC, Ortiz C, Cochi S, Ruiz-Matus C. Electronic immunization registries in Latin America: progress and lessons learned. Rev Panam Salud Publica. 2014 May-Jun;35(5-6):453-7. PMID: 25211576.

89. De Bustos EM, Ohannessian R, Bouamra B, Moulin T. Telemedicine and stroke: Telestroke. "The role of telemedicine in stroke". Bulletin De L Academie Nationale De Medecine. 2020 Oct;204(8):826-38. PMID: WOS:000602390500025. doi: 10.1016/j.banm.2020.07.038.

90. de Oliveira LR, Rolim RD, Tavares DR, Ferreira MMV, Coutinho AJF, Dantas GS, et al. DEVELOPMENT OF FORMATIVE SECOND OPINION: EXPERIENCES FROM THE BRAZILIAN NATIONAL TELEHEALTH NETWORK PROGRAM IN CEARA. In: Chova LG, Martinez AL, Torres IC, editors. 11th International Conference of Education, Research and Innovation. Valenica: Iated-Int Assoc Technology Education & Development; 2018. p. 3795-800.

91. Dean E. Use of new technology to help patients receive care at home. Nurs Older People. 2016 Mar;28(2):8-9. PMID: 26917174. doi: 10.7748/nop.28.2.8.s8.

92. DeAngles M. National Electronic Health Record Network Regulation and Synchronization of National and State Privacy Laws Needed to Increase Efficiency and Reduce Costs in Healthcare. J Leg Med. 2015 Jul-Dec;36(3-4):413-9. PMID: 28256941. doi: 10.1080/01947648.2015.1262194.

93. DeBor G, Diamond C, Grodecki D, Halamka J, Overhage JM, Shirky C. A tale of three cities--where RHIOS meet the NHIN. J Healthc Inf Manag. 2006 Summer;20(3):63-70. PMID: 16903663.

94. Decman M. Electronic Health Records Management and Preservation: The Case of Slovenia. Klun M, Decman M, Jukic T, editors. Nr Reading: Acad Conferences Ltd; 2011. 193-202 p. ISBN: 978-1-908272-00-3.

95. Dellavalle E, Perdoni V, Gadda L. A Semantic-based Healthcare Information Infrastructure to Integrate Medical Information and eHealth Services. In: Cunningham P, Cunningham M, editors. Innovation and the Knowledge Economy: Issues, Applications, Case Studies, Pts 1 & 2. Amsterdam: Ios Press; 2005. p. 888-95.

96. Dent L, Peters A, Kerr PL, Mochari-Greenberger H, Pande RL. Using Telehealth to Implement Cognitive-Behavioral Therapy. Psychiatr Serv. 2018 Apr 1;69(4):370-3. PMID: 29446336. doi: 10.1176/appi.ps.201700477.

97. Dias A, Boas MV, Polonia D, Queiros A, Rocha NP. Home Monitoring in Portugal An Overview on Current Experiences. VanDenBroek EL, Fred A, Gamboa H, Vaz M, editors. Setubal: Scitepress; 2017. 377-82 p. ISBN: 978-989-758-213-4.

98. Ding H, Ireland D, Jayasena R, Curmi J, Karunanithi M. Integrating a mobile health setup in a chronic disease management network. Stud Health Technol Inform. 2013;188:20-5. PMID: 23823283.

99. Dixon AK, FitzGerald R. Outsourcing and teleradiology: potential benefits, risks and solutions from a UK/European perspective. J Am Coll Radiol. 2008 Jan;5(1):12-8. PMID: 18180002. doi: 10.1016/j.jacr.2007.09.010.

100. Doarn CR. The last challenges and barriers to the development of telemedicine programs. Stud Health Technol Inform. 2008;131:45-54. PMID: 18305322.

101. Doarn CR, Merrell RC. A national strategy for telemedicine and e-health. Telemed J E Health. 2007 Jun;13(3):243-4. PMID: 17603825. doi: 10.1089/tmj.2007.9982.

102. Dogac A, Yuksel M, Avci A, Ceyhan B, Hülür U, Eryilmaz Z, et al. Electronic health record interoperability as realized in the Turkish health information system. Methods Inf Med. 2011;50(2):140-9. PMID: 21132219. doi: 10.3414/me10-01-0022.

103. Donahue M. Québec moves the telehealth yardstick forward. Health Law Can. 2006 Nov;27(2):29-33. PMID: 17370725.

104. Dorda W, Duftschmid G, Gerhold L, Gall W, Gambal J. Austria's path toward nationwide electronic health records. Methods Inf Med. 2008;47(2):117-23. PMID: 18338082. doi: 10.3414/me0401.

105. dos Santos Ade F, D'Agostino M, Bouskela MS, Fernandéz A, Messina LA, Alves HJ. [An overview of telehealth initiatives in Latin America]. Rev Panam Salud Publica. 2014 May-Jun;35(5-6):465-70. PMID: 25211578.

106. Dramos LMM, dos Santos AD, Sena MIB, Freitas MAC, Silva EMD, de Mello MDB, et al. Quality of Telehealth Services in Primary Care in the State of Minas Gerais. Telemed e-Health. 2021:8. PMID: WOS:000613491200001. doi: 10.1089/tmj.2020.0262.

107. Dramos LMM, Santos AFD, Sena MIB, Freitas MAC, Silva EMS, Mello M, et al. Quality of Telehealth Services in Primary Care in the State of Minas Gerais. Telemed J E Health. 2021 Sep;27(9):1054-61. PMID: 33493024. doi: 10.1089/tmj.2020.0262.

108. Drayton K. How mobile technology can improve healthcare. Nurs Times. 2013 Mar 19-25;109(11):16, 8. PMID: 23596769.

109. Drop SLS, Mure PY, Wood D, El-Ghoneimi A, Ahmed SF. E-consultation for DSD: A global platform for access to expert advice. J Pediatr Urol. 2012 Dec;8(6):629-32. PMID: WOS:000311962400011. doi: 10.1016/j.jpurol.2012.10.009.

110. Duplaga M. E-health development policies in new member states in Central Europe. World Hosp Health Serv. 2007;43(2):34-8. PMID: 17894192.

111. Eason K, Waterson P. The implications of e-health system delivery strategies for integrated healthcare: Lessons from England. International Journal of Medical Informatics. 2013 May;82(5):E96-E106. PMID: WOS:000318998000003. doi: 10.1016/j.ijmedinf.2012.11.004.

112. Easton J. Let's get serious about telehealth. Health Serv J. 2012 Jun 28;122(6311):18. PMID: 22950194.

113. Eber EL, Janda M, Arzberger E, Hofmann-Wellenhof R. Survey on the status of teledermatology in Austria. Journal Der Deutschen Dermatologischen Gesellschaft. 2019 Jan;17(1):25-31. PMID: WOS:000455009200004. doi: 10.1111/ddg.13729_g.

114. Ekman B, Thulesius H, Wilkens J, Lindgren A, Cronberg O, Arvidsson E. Utilization of digital primary care in Sweden: Descriptive analysis of claims data on demographics, socioeconomics, and diagnoses. Int J Med Inform. 2019 Jul;127:134-40. PMID: 31128825. doi: 10.1016/j.ijmedinf.2019.04.016.

115. El Sanadi N. Telehealth expansion requires advances in payment and coverage policies. Mod Healthc. 2015 Sep 14;45(37):25. PMID: 26619694.

116. Elawady A, Khalil A, Assaf O, Toure S, Cassidy C. Telemedicine during COVID-19: a survey of Health Care Professionals' perceptions. Monaldi Arch Chest Dis. 2020 Sep 22;90(4). PMID: 32959627. doi: 10.4081/monaldi.2020.1528.

117. Elliott T, Yopes MC. Direct-to-Consumer Telemedicine. Journal of Allergy and Clinical Immunology-in Practice. 2019 Nov-Dec;7(8):2546-52. PMID: WOS:000495746100004. doi: 10.1016/j.jaip.2019.06.027.

118. Evans S, Stemple C. Electronic health records and the value of health IT. J Manag Care Pharm. 2008 Aug;14(6):S16-S8. PMID: WOS:000259093900005.

119. Exum E, Hull BL, Lee ACW, Gumieny A, Villarreal C, Longnecker D. Applying Telehealth Technologies and Strategies to Provide Acute Care Consultation and Treatment of Patients With Confirmed or Possible COVID-19. Journal of Acute Care Physical Therapy. 2020 Jul;11(3):103-12. PMID: WOS:000552428300002. doi: 10.1097/jat.0000000000000143.

120. Farzandipour M, Meidani Z, Riazi H, Jabali MS. Task-specific usability requirements of electronic medical records systems: Lessons learned from a national survey of end-users. Inform Health Soc Care. 2018;43(3):280-99. PMID: WOS:000431555200005. doi: 10.1080/17538157.2017.1290639.

121. Fazzi R, Ashe T, Doak L. Telehealth and technology in home care: part I. Insights from the Philips National Study on the Future of Technology and Telehealth in Home Care. Caring. 2007 Oct;26(10):40-2, 4-7. PMID: 17988003.

122. Finch TL, Mair FS, May CR. Teledermatology in the UK: lessons in service innovation. Br J Dermatol. 2007 Mar;156(3):521-7. PMID: 17300243. doi: 10.1111/j.1365-2133.2006.07608.x.

123. Fleming JA, Brude S. Healthcare Information Technology. Issue Brief Health Policy Track Serv. 2018 Dec 24;2018:1-88. PMID: 30681314.

124. Fontenot SF. The Affordable Care Act and electronic health care records: can technology help reduce the cost of health care? Physician Exec. 2014 Jan-Feb;40(1):68-72. PMID: 24575706.

125. Freymann Fontenot S. The Affordable Care Act and electronic health care records. Does today's technology support the vision of a paperless health care system? Physician Exec. 2013 Nov-Dec;39(6):72-4, 6. PMID: 24354149.

126. Frisch LE, Borycki EM, Capron A, Mawudeku A, St John R. Public Health Informatics in Canada. In: Magnuson JA, Fu PC, editors. Public Health Informatics and Information Systems, 2nd Edition. New York: Springer; 2014. p. 603-18.

127. Gabriel ME, Furukawa MF, Vaidya V. Emerging and encouraging trends in e-prescribing adoption among providers and pharmacies. Am J Manag Care. 2013 Sep;19(9):760-4. PMID: 24304258.

128. Gall W, Aly AF, Sojer R, Spahni S, Ammenwerth E. The national e-medication approaches in Germany, Switzerland and Austria: A structured comparison. Int J Med Inform. 2016 Sep;93:14-25. PMID: 27435943. doi: 10.1016/j.ijmedinf.2016.05.009.

129. Ganapathy K, Das S, Reddy S, Thaploo V, Nazneen A, Kosuru A, et al. Digital Health Care in Public Private Partnership Mode. Telemed e-Health. 2021 Dec;27(12):1363-71. PMID: WOS:000637382100001. doi: 10.1089/tmj.2020.0499.

130. Geissbuhler A. Lessons learned implementing a regional health information exchange in Geneva as a pilot for the Swiss national eHealth strategy. International Journal of Medical Informatics. 2013 May;82(5):E118-E24. PMID: WOS:000318998000005. doi: 10.1016/j.ijmedinf.2012.11.002.

131. Gellis ZD, Kenaley BL, Ten Have T. Integrated Telehealth Care for Chronic Illness and Depression in Geriatric Home Care Patients: The Integrated Telehealth Education and Activation of Mood (I-TEAM) Study. J Am Geriatr Soc. 2014 May;62(5):889-95. PMID: WOS:000336385300013. doi: 10.1111/jgs.12776.

132. Gerkin DG. Telemedicine and e-health. Tenn Med. 2008 Jul;101(7):6-7, 10. PMID: 18649619.

133. Giansanti D, Castrichella L, Giovagnoli MR. Telepathology: Success or Failure? In: Jarm T, Kramar P, Zupanic A, editors. 11th Mediterranean Conference on Medical and Biological Engineering and Computing 2007, Vols 1 and 2. Berlin: Springer-Verlag Berlin; 2007. p. 745-+.

134. Giordanengo A, Bradway M, Grottland A, Hartvigsen G, Arsand E. A FHIR-based data flow enabling patients with diabetes to share self-collected data with the Norwegian national healthcare systems and electronic health record systems. Diabetes Technol Ther. 2018 Feb;20:A109-A. PMID: WOS:000425015800255.

135. Glaser J. The electronic health record: a digital divide? Healthc Financ Manage. 2007 Oct;61(10):38-40, 2. PMID: 17953180.

136. Goddard A, Sullivan E, Fields P, Mackey S. The Future of Telehealth in School-Based Health Centers: Lessons from COVID-19. J Pediatr Health Care. 2021 Jan 29. PMID: 33518441. doi: 10.1016/j.pedhc.2020.11.008.

137. Goldstein MM, Thorpe Jane H. The First Anniversary of the Health Information Technology for Economic and Clinical Health (HITECH) Act: the regulatory outlook for implementation. Perspect Health Inf Manag. 2010 Sep 1;7(Summer). PMID: 20808607.

138. Gomel N, Azem N, Baruch T, Hollander N, Rachmiel R, Kurtz S, et al. Teleophthalmology Screening for Early Detection of Ocular Diseases in Underserved Populations in Israel. Telemed J E Health. 2022 Feb;28(2):233-9. PMID: 33999746. doi: 10.1089/tmj.2021.0098.

139. Goozner M. National action needed to advance telemedicine. Mod Healthc. 2015 Jan 19;45(3):26. PMID: 25671904.

140. Gordon CR. The mobile technology era: HITECH and expectations for the future. Plast Reconstr Surg. 2013 Aug;132(2):319e. PMID: 23897367. doi: 10.1097/PRS.0b013e3182958eaf.

141. Gorton M. Welcome to the world of telehealth: physicians reaping significant benefits. J Med Pract Manage. 2008 Nov-Dec;24(3):147-50. PMID: 19146083.

142. Gregory S, Llewellyn C. Store and forward Teledermatology - the Newport way. J Vis Commun Med. 2018 Apr;41(2):45-51. PMID: 29355433. doi: 10.1080/17453054.2018.1425609.

143. Griffin J. Transforming health care through technology. NCSL Legisbrief. 2011 Aug-Sep;19(35):1-2. PMID: 22022749.

144. Gundlapalli AV, Reid JH, Root J, Xu W, Shukla A, Tiwari R. Regional and Community Health Information Exchange in the United States. Hersey: Igi Global; 2011. 198-218 p. ISBN: 978-1-61692-005-0; 978-1-61692-004-3.

145. Haddad P, Muhammad I, Wickramasinghe N, Assoc Informat S. Assessing the Business Value of Australia's National e-health Solution. Atlanta: Assoc Information Systems; 2016. ISBN: 978-0-9966831-2-8.

146. Haddad PJ. Telemedicine in Michigan: What Physicians Need to Know. Mich Med. 2016 Mar-Apr;115(2):10-3. PMID: 27149753.

147. Halamka J. The perfect storm for electronic health records. J Healthc Inf Manag. 2006 Summer;20(3):25-7. PMID: 16903658.

148. Halpern NA. From telemedicine to a critical care database: a new resource for national benchmarking. Chest. 2011 Nov;140(5):1111-3. PMID: 22045874. doi: 10.1378/chest.11-2004.

149. Hannan TJ. Solving a Health Information Management Problem. An international success story. World Hosp Health Serv. 2015;51(2):32-5. PMID: 26521384.

150. Harnett B. Creating telehealth networks from existing infrastructures. Stud Health Technol Inform. 2008;131:55-65. PMID: 18305323.

151. Heisey-Grove D, Danehy LN, Consolazio M, Lynch K, Mostashari F. A National Study of Challenges to Electronic Health Record Adoption and Meaningful Use. Med Care. 2014 Feb;52(2):144-8. PMID: WOS:000331087100010. doi: 10.1097/mlr.0000000000000038.

152. Henry TA. Telemedicine: Transforming Georgia's landscape. J Med Assoc Ga. 2015;104(3):8-10. PMID: 26665999.

153. Herendeen NE, Schaefer GB. Practical applications of telemedicine for pediatricians. Pediatr Ann. 2009 Oct;38(10):567-9. PMID: 19968195. doi: 10.3928/00904481-20090918-04.

154. Hertz B. Navigate 2016's Reimbursement Challenges. Get paid what you're owed. Med Econ. 2016 Jan 10;93(1):26-30. PMID: 27078908.

155. Ho TW, Lai TY. [The functional structure of Taiwan's Pilot Telecare Project]. Hu Li Za Zhi. 2008 Aug;55(4):17-23. PMID: 18668478.

156. Hoerbst A, Kohl CD, Knaup P, Ammenwerth E. Attitudes and behaviors related to the introduction of electronic health records among Austrian and German citizens. International Journal of Medical Informatics. 2010 Feb;79(2):81-9. PMID: WOS:000274568900002. doi: 10.1016/j.ijmedinf.2009.11.002.

157. Hofert J, Bossen R, Schramm L, Dowell M. Healthcare risk management challenges created by federal regulation of electronic medical records and care management. J Healthc Risk Manag. 2013;33(1):45-51. PMID: 23861123. doi: 10.1002/jhrm.21117.

158. Hofmeyer J, Leider JP, Satorius J, Tanenbaum E, Basel D, Knudson A. Implementation of Telemedicine Consultation to Assess Unplanned Transfers in Rural Long-Term Care Facilities, 2012-2015: A Pilot Study. J Am Med Dir Assoc. 2016 Nov 1;17(11):1006-10. PMID: 27477614. doi: 10.1016/j.jamda.2016.06.014.

159. Holmgren AJ, Adler-Milstein J. Health Information Exchange in US Hospitals: The Current Landscape and a Path to Improved Information Sharing. J Hosp Med. 2017 Mar;12(3):193-8. PMID: WOS:000399577700012. doi: 10.12788/jhm.2704.

160. Holt F. Making a difference with a telehealth service. Br J Nurs. 2007 Oct 11-24;16(18):1108-9. PMID: 18073679. doi: 10.12968/bjon.2007.16.18.27500.

161. Horner K, Wagner E, Tufano J. Electronic consultations between primary and specialty care clinicians: early insights. Issue Brief (Commonw Fund). 2011 Oct;23:1-14. PMID: 22059281.

162. Horwood T, Aylott M, Loyola M, Henderson J, Frank G, Fyfe ML. Telecardiology on Vancouver Island: imagination to implementation. Stud Health Technol Inform. 2013;183:63-7. PMID: 23388256.

163. Hostiuc F, Buciu A. Integration of Technology in the MnTS. In: Doarn CR, Latifi R, Hostiuc F, Arafat R, Zoicas C, editors. Multinational Telemedicine Systems for Disaster Response: Opportunities and Challenges. Amsterdam: Ios Press; 2017. p. 46-58.

164. Hovenga EJS. National Standards in Health Informatics. In: Hovenga EJS, Kidd MR, Garde S, Cossio CHL, editors. Health Informatics: An Overview. Amsterdam: Ios Press; 2010. p. 133-55.

165. Hovenga EJS, Grain H. Health Information Systems. In: Hovenga EJS, Grain H, editors. Health Information Governance in a Digital Environment. Amsterdam: Ios Press; 2013. p. 120-40.

166. Huffman JR. Mobile health technology and the law. Health Care Law Mon. 2012 Jun;2012(6):2-5. PMID: 22779143.

167. Ilioudi S, Lazakidou A. Principles and effects of electronic communication systems between healthcare providers and managed-care organisations. Int J Electron Healthc. 2007;3(4):468-78. PMID: 18048278. doi: 10.1504/ijeh.2007.015324.

168. Jackson CL. Our first National Primary Health Care Strategy: 3 years on, what change for general practice? Med J Aust. 2013 Jun 17;198(11):581-2. PMID: 23919691. doi: 10.5694/mja13.10298.

169. Jamoom EW, Yang N. State Variation in Electronic Sharing of Information in Physician Offices: United States, 2015. NCHS Data Brief. 2016 Oct(261):1-8. PMID: 27805548.

170. Johnson A. Digital doctors: telemedicine improves medical care in remote areas, but faces some policy obstacles. State Legis. 2006 Jun;32(6):30-2. PMID: 16791976.

171. Joseph RC, Lim CT, Huang H, Lacasse JJ. Expanding Access to Psychiatric Care: Implementation of Psychiatric e-Consultation and Tele-consultation for Primary Care in a Safety-net Health System. J Health Care Poor Underserved. 2022;33(2):767-78. PMID: 35574875. doi: 10.1353/hpu.2022.0062.

172. Juhra C, Ansorg J, Back DA, John D, Kuckuck-Winkelmann A, Raschke M, et al. Online Patient Consultation. Z Orthop Unfall. 2020 Aug;158(4):345-50. PMID: 32819007. doi: 10.1055/a-1192-7800.

173. Jung SY, Lee K, Lee HY, Hwang H. Barriers and facilitators to implementation of nationwide electronic health records in the Russian Far East: A qualitative analysis. International Journal of Medical Informatics. 2020 Nov;143:7. PMID: WOS:000600412800016. doi: 10.1016/j.ijmedinf.2020.104244.

174. Kahn C. Telehealth is already showing value, but the feds could open the door to even more gains. Mod Healthc. 2017 Feb;47(8):25. PMID: 30605581.

175. Kaipio J, Laaveri T, Hypponen H, Vainiomaki S, Reponen J, Kushniruk A, et al. Usability problems do not heal by themselves: National survey on physicians' experiences with EHRs in Finland. International Journal of Medical Informatics. 2017 Jan;97:266-81. PMID: WOS:000390485500026. doi: 10.1016/j.ijmedinf.2016.10.010.

176. Kaldy J. Digital Therapeutics: Health Care Wired for the Future. Sr Care Pharm. 2020 Aug 1;35(8):338-44. PMID: 32718389. doi: 10.4140/TCP.n.2020.338.

177. Kasitipradith N. The Ministry of Public Health Telemedicine Network of Thailand. International Journal of Medical Informatics. 2001 May;61(2-3):113-6. PMID: WOS:000169550300005. doi: 10.1016/s1386-5056(01)00133-2.

178. Kern J, Erceg M, Poljicanin T. [Efficacy of public health surveillance systems]. Acta Med Croatica. 2010 Dec;64(5):415-23. PMID: 21692266.

179. Kertesz L. Legislation supports progress in health information technology. AHIP Cover. 2009 Mar-Apr;50(2):10, 51, 3-4. PMID: 19385312.

180. Ketchersid T. Health information technology: driving adoption. Nephrol News Issues. 2011 Sep;25(10):30-1. PMID: 21998979.

181. Kiefer B. [Health politics, 2012]. Rev Med Suisse. 2012 Jan 18;8(324):144. PMID: 23185829.

182. King I, Heidler P. Teleconsultation Services in Pediatric Primary Care in Austria: Upgrading Clinical Decision Support in Primary Health Care Settings. Telemed J E Health. 2022 Mar 29. PMID: 35357947. doi: 10.1089/tmj.2021.0452.

183. Kinoshita S, Kishimoto T. Current Status and Challenges of the Dissemination of Telemedicine in Japan After the Start of the COVID-19 Pandemic. Telemed J E Health. 2022 Aug;28(8):1220-4. PMID: 34918952. doi: 10.1089/tmj.2021.0336.

184. Kirkner RM. Texas's Corralling of Telemedicine Won't Start a National Stampede. Manag Care. 2015 Jun;24(6):12-3. PMID: 26182715.

185. Kohler M. Telemedicine seeks to empower patients to manage their care. Br J Community Nurs. 2008 Mar;13(3):135-7. PMID: 18557576. doi: 10.12968/bjcn.2008.13.3.28679.

186. Konduri N, Bastos LGV, Sawyer K, Reciolino LFA. User experience analysis of an eHealth system for tuberculosis in resource-constrained settings: A nine-country comparison. International Journal of Medical Informatics. 2017 Jun;102:118-29. PMID: WOS:000405361900014. doi: 10.1016/j.ijmedinf.2017.03.017.

187. Kralj D, Koncar M, Tonkovic S. A Methodology to Assess Experiences in Implementing e-Health Solutions in Croatian Family Medicine. In: StoicuTivader L, Blobel B, Marcun T, Orel A, editors. E-Health across Borders without Boundaries: E-Salus Trans Confinia Sine Finibus. Amsterdam: Ios Press; 2011. p. 129-34.

188. Krania E, Avgerinou E, Limaki E, Bartzis G, Mantzana V. E-prescribing in Greece: Myth or reality. In: Mantas J, Hasman A, editors. Informatics, Management and Technology in Healthcare. Amsterdam: Ios Press; 2013. p. 194-7.

189. Kuhne M, Breitschwerdt R. Adoption and diffusion of web 2.0 applications and social networks in the German health insurance landscape. Schreier G, Hayn D, Horbst A, Ammenwerth E, editors. Wien: Osterreichische Computer Gesellschaft-Ocg; 2012. 165-72 p. ISBN: 978-3-85403-289-2.

190. Kwak YS. Electronic Health Record: Definition, Categories and Standards. Healthc Inform Res. 2005 Mar;11(1):1-15. PMID: WOS:000219319300001.

191. Ladika S. Tuning in to Telemedicine. Manag Care. 2015 Jul;24(7):14-8. PMID: 26281465.

192. Ladika S. Telehealth Dials Up Discussion About Payment to Providers. Manag Care. 2016 Jun;25(6):15-9. PMID: 27464368.

193. Ladika S. Telehealth Overview: The Reality Check, Please. Manag Care. 2017 Apr;26(4):16-8. PMID: 28511757.

194. Lamb N. The NHS can learn from US success stories. Health Serv J. 2013 Jun 21;123(6356):16-7. PMID: 23946993.

195. Latifi R. "Initiate-Build-Operate-Transfer" - A Strategy for Establishing Sustainable Telemedicine Programs Not Only in the Developing Countries. In: StoicuTivader L, Blobel B, Marcun T, Orel A, editors. E-Health across Borders without Boundaries: E-Salus Trans Confinia Sine Finibus. Amsterdam: Ios Press; 2011. p. 3-10.

196. Latifi R, Boci A. Implementation of Telemedicine in Albania The First Eight Years: Making a Case to Continue. In: Stone CS, editor. Benchmarking Telemedicine: Improving Health Security in the Balkans. Amsterdam: Ios Press; 2017. p. 95-103.

197. Lawrence D. Telemedicine: with limited specialists, more area to cover, and better reimbursement models, many hospitals are embracing telemedicine. Healthc Inform. 2009 Feb;25(14):42-4. PMID: 19266854.

198. Lear SA, Bates J, Lavoie JG, Johnston S, Scott RE. The British Columbia Alliance on Telehealth Research and Policy. Healthc Q. 2008;11(4):52-6, 2. PMID: 18818530. doi: 10.12927/hcq.2008.20092.

199. Liang J, Zheng X, Chen ZY, Dai SQ, Xu J, Ye H, et al. The experience and challenges of healthcare-reform-driven medical consortia and Regional Health Information Technologies in China: A longitudinal study. International Journal of Medical Informatics. 2019 Nov;131:8. PMID: WOS:000488525700004. doi: 10.1016/j.ijmedinf.2019.103954.

200. Lieber HS. Health IT provides the tools to drive change, implement strategies that improve care. Mod Healthc. 2016 Feb 29;46(9):38. PMID: 27079025.

201. Lieu G, Cho V. Perceptions, Expectations and Support for a Community-Wide eHR System Among Hong Kong Residents. Asia Pacific Journal of Health Management. 2011;6(1):30-42. PMID: WOS:000214925700009.

202. Linder M. Mobile health technology: touching lives across the globe. Health Manag Technol. 2012 Jun;33(6):6-7. PMID: 22787942.

203. Linkous JD. Telemedicine's promise: reaching new heights. Provider. 2006 Mar;32(3):42-4. PMID: 16910185.

204. Lopez DM. A reference Model for the Specification and Analysis of Information Systems for Public Health Surveillance. Sistemas & Telematica. 2007;5(10):29-46. PMID: WOS:000215564200002.

205. Luk CY. The Impact of Digital Health on Traditional Healthcare Systems and Doctor-Patient Relationships: The Case Study of Singapore. Innovative Perspectives on Public Administration in the Digital Age. Hersey: Igi Global; 2018. p. 143-67.

206. Lukosevicius A. Building and Implementing an eHealth Strategy: is there a Good Recipe for Baltic Countries? In: Katashev A, Dekhtyar Y, Spigulis J, editors. 14th Nordic-Baltic Conference on Biomedical Engineering and Medical Physics. New York: Springer; 2008. p. 1-4.

207. Mac McCullough J, Goodin K. Clinical Data Systems to Support Public Health Practice: A National Survey of Software and Storage Systems Among Local Health Departments. J Public Health Manag Pract. 2016 Nov-Dec;22:S18-S26. PMID: WOS:000385458200006. doi: 10.1097/phh.0000000000000443.

208. MacGinnis G. The National Health Service in England - Moving to Mainstream Use of Ambient Assisted Living Technology. In: Augusto JC, Huch M, Kameas A, Maitland J, McCullagh P, Roberts J, et al., editors. Handbook of Ambient Assisted Living: Technology for Healthcare, Rehabilitation and Well-Being. Amsterdam: Ios Press; 2012. p. 37-66.

209. Mack D, Brantley KM, Bell KG. Mitigating the health effects of disasters for medically underserved populations: electronic health records, telemedicine, research, screening, and surveillance. J Health Care Poor Underserved. 2007 May;18(2):432-42. PMID: 17483570. doi: 10.1353/hpu.2007.0040.

210. Malcolm EJ, Brandon Z, Wilson LE, Shoup JP, King HA, Lewinski A, et al. eConsults' Impact on Care Access and Wait Times in Rheumatology. JCR-J Clin Rheumatol. 2022 Apr;28(3):147-54. PMID: WOS:000772599500007. doi: 10.1097/rhu.0000000000001825.

211. Mansilla E, Mazzon E, Carcamo D, Jurado F, Lara L, Arevalo M, et al. Telestroke in Chile: 1 year experience at 7 hospitals. Revista Medica De Chile. 2019 Sep;147(9):1109-15. PMID: WOS:000504650600003.

212. Mantero A, Posteraro A, Giordano G, Tonti G, Pinciroli F. The problem of the correct implementation of Information and Communication Technology in medicine in our country: document of the "e-cardio" Area of the National Association of Cardiologists and Hospital Doctors (ANMCO). G Ital Cardiol. 2013 Dec;14(12):867-74. PMID: WOS:000444319600009. doi: 10.1714/1371.15243.

213. Marcin JP, Trujano J, Sadorra C, Dharmar M. Telemedicine in Rural Pediatric Care: The Fundamentals. Pediatr Ann. 2009 Apr;38(4):224-6. PMID: WOS:000267004300009. doi: 10.3928/00904481-20090401-10.

214. Marco Cuenca G, Salvador Olivan JA. Knowledge representation in electronic interoperable health records: the case of the Digital Health Record of the Spanish National Health System. Scire-Representacion Y Organizacion Del Conocimiento. 2017 Jan-Jun;23(1):25-38. PMID: WOS:000406218700002.

215. Marimon-Sunol S, Rovira-Barbera M, Acedo-Anta M, Nozal-Baldajos MA, Guanyabens-Calvet J. Shared Electronic Health Record in Catalonia, Spain. Med Clin (Barc). 2010 Feb;134:45-8. PMID: WOS:000277354300009. doi: 10.1016/s0025-7753(10)70009-9.

216. Marti M, Balladelli PP, Gherardi A. [Implementation of the eHealth Strategy and Plan of Action in Argentina, 2011-2013]. Rev Panam Salud Publica. 2014 May-Jun;35(5-6):432-6. PMID: 25211572.

217. Maxson ER, Buntin MJ, Mostashari F. Using electronic prescribing transaction data to estimate electronic health record adoption. Am J Manag Care. 2010 Dec;16(12 Suppl HIT):e320-6. PMID: 21322303.

218. Mazzolini C. Telemedicine's next big leap. Med Econ. 2013 Oct 25;90(20):64-6. PMID: 25233739.

219. McCarthy M. Telehealth or Tele-education? Providing intensive, ongoing therapy to remote communities. Stud Health Technol Inform. 2010;161:104-11. PMID: 21191163.

220. McDaniel AM, Schutte DL, Keller LO. Consumer health informatics: from genomics to population health. Nurs Outlook. 2008 Sep-Oct;56(5):216-23.e3. PMID: 18922271. doi: 10.1016/j.outlook.2008.06.006.

221. McInnes DK, Saltman DC, Kidd MR. General practitioners' use of computers for prescribing and electronic health records: results from a national survey. Med J Aust. 2006 Jul;185(2):88-91. PMID: WOS:000239731000011. doi: 10.5694/j.1326-5377.2006.tb00479.x.

222. McKoy J, Fitzner K, Margetts M, Heckinger E, Specker J, Roth L, et al. Are Telehealth Technologies for Hypertension Care and Self-Management Effective or Simply Risky and Costly? Popul Health Manag. 2015 Jun;18(3):192-202. PMID: WOS:000363965700007. doi: 10.1089/pop.2014.0073.

223. McNeill KM, Weinstein RS, Ovitt TW. Project nightingale: A geographically distributed, multi-organizational integrated telemedicine network infrastructure. In: Lemke HU, Inamura K, Vannier MW, Farman AG, editors. Cars '99: Computer Assisted Radiology and Surgery. Amsterdam: Elsevier Science Bv; 1999. p. 550-3.

224. Meijer WJ. The Patient's Perspective in the Dutch National Technical Agreement on Telemedicine. In: Bos L, Blobel B, Marsh A, Carroll D, editors. Medical and Care Compunetics 5. Amsterdam: I O S Press; 2008. p. 26-33.

225. Menon S, Singh H, Meyer AN, Belmont E, Sittig DF. Electronic health record-related safety concerns: a cross-sectional survey. J Healthc Risk Manag. 2014;34(1):14-26. PMID: 25070253. doi: 10.1002/jhrm.21146.

226. Merlière Y. [The shared medical record, a digital health record for all]. Soins. 2020 Jan-Feb;65(842):29-32. PMID: 32245555. doi: 10.1016/j.soin.2020.01.005.

227. Merrell RC, Doarn CR. Tales of telemedicine: I met my doctor on television. Telemed J E Health. 2013 Aug;19(8):571-2. PMID: 23806083. doi: 10.1089/tmj.2013.9990.

228. Merrell RC, Doarn CR. Tales of telemedicine--telepsychiatry at work. Telemed J E Health. 2013 Apr;19(4):233-4. PMID: 23540275. doi: 10.1089/tmj.2013.9995.

229. Mettner J. Virtual visits. Minn Med. 2008 Oct;91(10):15-7. PMID: 18991007.

230. Mielonen ML, Ohinmaa A, Moring J, Isohanni M. The use of videoconferencing for telepsychiatry in Finland. J Telemed Telecare. 1998;4(3):125-31. PMID: WOS:000075907400001. doi: 10.1258/1357633981932073.

231. Mizushima H, Uchiyama E, Nagata H, Matsuno Y, Sekiguchi R, Ohmatsu H, et al. Japanese experience of telemedicine in oncology. International Journal of Medical Informatics. 2001 May;61(2-3):207-15. PMID: WOS:000169550300014. doi: 10.1016/s1386-5056(01)00142-3.

232. Mohan J, Yaacob RRR. The Malaysian Telehealth Flagship Application: a national approach to health data protection and utilisation and consumer rights. International Journal of Medical Informatics. 2004 Mar;73(3):217-27. PMID: WOS:000221013900002. doi: 10.1016/j.ijmedinf.2003.11.023.

233. Moisil I, Jitaru E. E-health progresses in Romania. International Journal of Medical Informatics. 2006 Mar-Apr;75(3-4):315-21. PMID: WOS:000236360500017. doi: 10.1016/j.ijmedinf.2005.08.013.

234. Monique F. Health Care Engineering, Part I: Clinical Engineering and Technology Management: Morgan & Claypool; 2013. 1 p. ISBN: 9781608453672.

235. Moore A. Telehealth. Home comfort. Health Serv J. 2011 Apr 28;121(6254):17-8. PMID: 21604655.

236. Moore A. Telehealth. Remote control of care. Monitoring patients with chronic conditions in. Health Serv J. 2014 Jun 6;124(6401):24-5. PMID: 25046905.

237. Morland LA, Poizner JM, Williams KE, Masino TT, Thorp SR. Home-based clinical video teleconferencing care: Clinical considerations and future directions. Int Rev Psychiatry. 2015 Nov;27(6):504-12. PMID: WOS:000366215300006. doi: 10.3109/09540261.2015.1082986.

238. Morosini P, Campanella N, Lucci G, Giorgetti A, Lorenzetti V, Manfrini E, et al. [The televideoconsultation in support of home management of the patient with CoViD-19 by general practitioner: the results in some municipalities of the Marche Region.]. Recenti Prog Med. 2020 Jul-Aug;111(7):454-60. PMID: 32658884. doi: 10.1701/3407.33927.

239. Morozova LF. [Use of geographic information systems in public health]. Med Parazitol (Mosk). 2014 Jul-Sep(3):46-51. PMID: 25286553.

240. Morrissey J. Telemedicine:'If you aren't doing anything now, you're way behind'. Hosp Health Netw. 2013 Oct;87(10):22-3, 2. PMID: 24303630.

241. Morrissey J. Regulatory clouds part for mHealth apps, but barriers for full integration remain. Hosp Health Netw. 2014 Feb;88(2):22-3. PMID: 24693731.

242. Muir J, Lucas L. Tele-dermatology in Australia. Stud Health Technol Inform. 2008;131:245-53. PMID: 18305334.

243. Najjar J, Bourquard JA. High-tech health care: lawmakers are building up telehealth by breaking down barriers. State Legis. 2012 Dec;38(10):26-8. PMID: 23547325.

244. Naka F, Lu J, Porto A, Villagra J, Wu ZH, Anderson D. Impact of dermatology eConsults on access to care and skin cancer screening in underserved populations: A model for teledermatology services in community health centers. J Am Acad Dermatol. 2018 Feb;78(2):293-302. PMID: 29061478. doi: 10.1016/j.jaad.2017.09.017.

245. Nalin M, Baroni I, Faiella G, Romano M, Matrisciano F, Gelenbe E, et al. The European cross-border health data exchange roadmap: Case study in the Italian setting. Journal of Biomedical Informatics. 2019 Jun;94:11. PMID: WOS:000525692600029. doi: 10.1016/j.jbi.2019.103183.

246. Nall J. CDC 2.0: using social media to improve public health. Proceedings of the 11th Annual International Digital Government Research Conference on Public Administration Online: Challenges and Opportunities; Puebla, Mexico: Digital Government Society of North America; 2010. p. 1.

247. Neokleous KC, Schiza EC, Pattichis CS, Schizas CN. A Patient Centered Electronic Health System: An Example for Cyprus. In: Mantas J, Househ MS, Hasman A, editors. Integrating Information Technology and Management for Quality of Care. Amsterdam: Ios Press; 2014. p. 111-4.

248. Nevet A, Bitton Y, Wolf L, Waisman Y. [Telemedicine: a novel service in pediatric emergency care]. Harefuah. 2016 Jul;155(7):410-3. PMID: 28514123.

249. Nguyen QT, Naguib RNG, Tawfik H, Phuong NH. Strategic challenges facing user-and patient-centred e-health in Vietnam. International Journal of Healthcare Technology and Management. 2012;13(5-6):378-94. PMID: WOS:000216232900008. doi: 10.1504/ijhtm.2012.052551.

250. Nusbaum NJ, Peratrovich J. Some Thoughts on Implementing Remote Telehealth. South Med J. 2018 Sep;111(9):542-3. PMID: 30180251. doi: 10.14423/smj.0000000000000860.

251. O'Sullivan UM, Somers J. Southern Health Board - Advanced Telematic/Telemedicine in healthcare services in the South West of Ireland. In: Nerlich M, Kretschmer R, editors. Impact of Telemedicine on Health Care Management. Amsterdam: I O S Press; 1999. p. 230-40.

252. Obi T, Ishmatova D, Iwasaki N. Promoting ICT innovations for the ageing population in Japan. Int J Med Inform. 2013 Apr;82(4):e47-62. PMID: 22727612. doi: 10.1016/j.ijmedinf.2012.05.004.

253. Ogle SM, Cooke CE, Brandt NJ. Medication Management and e-Care Planning: What are the Opportunities for the Future? J Gerontol Nurs. 2015 Oct;41(10):13-7. PMID: 26488251. doi: 10.3928/00989134-20150915-02.

254. Ohannessian R, Dhote-Burger P, Chauvin F, Colin C, Nighoghossian N, Moulin T, et al. Health policy for telestroke in France: A retrospective description from 2003 to 2016. Rev Neurol (Paris). 2019 Jun;175(6):390-5. PMID: 30736986. doi: 10.1016/j.neurol.2018.10.002.

255. Oliveira TC, Branquinho MJ, Gonçalves L. State of the art in telemedicine - concepts, management, monitoring and evaluation of the telemedicine programme in Alentejo (Portugal). Stud Health Technol Inform. 2012;179:29-37. PMID: 22925783.

256. Olsen J, Peterson S, Stevens A. Implementing electronic health record-based National Diabetes Prevention Program referrals in a rural county. Public Health Nurs. 2020 Dec 26. PMID: 33368605. doi: 10.1111/phn.12860.

257. Ortolon K. Going online. Tex Med. 2007 Mar;103(3):35-9. PMID: 17542469.

258. Pangalos G, Asimakopoulos D, Pagkalos I. The new Greek national e-prescription system: an effective tool for improving quality of care and containing medication costs. In: Mantas J, Hasman A, editors. Informatics, Management and Technology in Healthcare. Amsterdam: Ios Press; 2013. p. 13-7.

259. Pangalos G, Sfyroeras V, Pagkalos I. E-prescription as a tool for improving services and the financial viability of healthcare systems: the case of the Greek national e-prescription system. Int J Electron Healthc. 2014;7(4):301-14. PMID: 25161106. doi: 10.1504/ijeh.2014.064331.

260. Parker SB, deShazo RD, Adcock M, Rodenmeyer K. Telemedicine in Mississippi: Can It Improve Our Last Place in Health? J Miss State Med Assoc. 2016 Nov;57(11):342-8. PMID: 30281233.

261. Parks R, Wigand RT, Othmani MB, Serhier Z, Bouhaddou O. Electronic health records implementation in Morocco: Challenges of silo efforts and recommendations for improvements. International Journal of Medical Informatics. 2019 Sep;129:430-7. PMID: WOS:000483422400055. doi: 10.1016/j.ijmedinf.2019.05.026.

262. Parv L, Kruus P, Motte K, Ross P. An evaluation of e-prescribing at a national level. Inform Health Soc Care. 2016 Jan;41(1):78-95. PMID: WOS:000372152500005. doi: 10.3109/17538157.2014.948170.

263. Pearce CM, de Lusignan S, Phillips C, Hall S, Travaglia J. The Computerized Medical Record as a Tool for Clinical Governance in Australian Primary Care. Interactive Journal of Medical Research. 2013 Jul-Dec;2(2):21-33. PMID: WOS:000218915500003. doi: 10.2196/ijmr.2700.

264. Pearce FW. The Alaska telemedicine testbed project (1996-2001): digital healthcare in a narrow bandwidth environment. Proceedings of the 2002 annual national conference on Digital government research; Los Angeles, California, USA: Digital Government Society of North America; 2002. p. 1–51.

265. Perna G. The promise of mobile: connecting to underserved populations. Healthc Inform. 2013 Jul-Aug;30(5):12, 4, 6. PMID: 24027812.

266. Perrier-Bonnet S. [A telemedicine consultation in the framework of a wound and wound healing network]. Rev Infirm. 2016 Aug-Sep;223:35-7. PMID: 27633698. doi: 10.1016/j.revinf.2016.06.010.

267. Peskin SR. Is'mobile health' revolution made for managed care? Manag Care. 2010 Dec;19(12):20-3. PMID: 21291127.

268. Pflugeisen BM, McCarren C, Poore S, Carlile M, Schroeder R. Virtual Visits: Managing prenatal care with modern technology. Mcn-the American Journal of Maternal-Child Nursing. 2016 Jan-Feb;41(1):24-30. PMID: WOS:000367838300004. doi: 10.1097/nmc.0000000000000199.

269. Phillips J. Reducing admissions for long-term conditions: is telehealth the answer? Br J Community Nurs. 2012 Feb;17(2):76. PMID: 22306599. doi: 10.12968/bjcn.2012.17.2.76.

270. Plantier M, Havet N, Durand T, Caquot N, Amaz C, Biron P, et al. Does adoption of electronic health records improve the quality of care management in France? Results from the French e-SI (PREPS-SIPS) study. International Journal of Medical Informatics. 2017 Jun;102:156-65. PMID: WOS:000405361900018. doi: 10.1016/j.ijmedinf.2017.04.002.

271. Popescu AI. An assessment of e-Health services implementation in Romania. Metalurgia International. 2010 Mar;15(3):62-6. PMID: WOS:000274149300012.

272. Prinz L, Cramer M, Englund A. Telehealth: A policy analysis for quality, impact on patient outcomes, and political feasibility. Nurs Outlook. 2008 Jul-Aug;56(4):152-8. PMID: WOS:000258393000006. doi: 10.1016/j.outlook.2008.02.005.

273. Protti D, Johansen I, Perez-Torres F. Comparing the application of Health Information Technology in primary care in Denmark and Andalucia, Spain. International Journal of Medical Informatics. 2009 Apr;78(4):270-83. PMID: WOS:000264669200006. doi: 10.1016/j.ijmedinf.2008.08.002.

274. Pruthi S, Stange KJ, Malagrino GD, Chawla KS, LaRusso NF, Kaur JS. Successful Implementation of a Telemedicine-Based Counseling Program for High-Risk Patients With Breast Cancer. Mayo Clin Proc. 2013 Jan;88(1):68-73. PMID: WOS:000312807000014. doi: 10.1016/j.mayocp.2012.10.015.

275. Ramalanjaona G, Brogan GX. EMS in Mauritius. Resuscitation. 2009 Feb;80(2):163-4. PMID: 19081170. doi: 10.1016/j.resuscitation.2008.10.006.

276. Raths D. Trend: telehealth. Is telehealth going mainstream? Healthc Inform. 2013 Mar;30(2):30-1. PMID: 23581008.

277. Raths D. Telehealth makes gains out west. Healthc Inform. 2014 Jul-Aug;31(5):20-1. PMID: 25230444.

278. Ray S, Biswas GP. Design of RSA-CA Based E-Health System for Supporting HIPAA Privacy-Security Regulations. In: Jena SK, Majhi B, editors. 2nd International Conference on Communication, Computing & Security [Icccs-2012]. Amsterdam: Elsevier Science Bv; 2012. p. 954-61.

279. Reddy KV. Using teledentistry for providing the specialist access to rural Indians. Indian J Dent Res. 2011 Mar-Apr;22(2):189. PMID: 21891882. doi: 10.4103/0970-9290.84275.

280. Reidt S, Holtan H, Stender J, Salvatore T, Thompson B. Integrating home-based medication therapy management (MTM) services in a health system. J Am Pharm Assoc (2003). 2016 Mar-Apr;56(2):178-83. PMID: 27000169. doi: 10.1016/j.japh.2016.01.003.

281. Reponen J, Winblad I, Hamalainen P. Current Status of National eHealth and Telemedicine Development in Finland. In: Blobel B, Pharow P, Nerlich M, editors. Ehealth: Combining Health Telematics, Telemedicine, Biomedical Engineering and Bioinformatics to the Edge: Global Experts Summit Textbook. Amsterdam: Ios Press; 2008. p. 199-+.

282. Roberts LJ, LaMont EG, Lim I, Sabesan S, Barrett C. Telerheumatology: an idea whose time has come. Intern Med J. 2012 Oct;42(10):1072-8. PMID: WOS:000309752100002. doi: 10.1111/j.1445-5994.2012.02931.x.

283. Rose JL. Improved and expanded pharmacy care in rural Alaska through telepharmacy and alternative methods demonstration project. Int J Circumpolar Health. 2007;66 Suppl 1:14-22. PMID: 18154228.

284. Rosser JC, Bell RL, Harnett B, Rodas E, Murayama M, Merrell R. Use of mobile low-bandwith telemedical techniques for extreme telemedicine applications. J Am Coll Surg. 1999 Oct;189(4):397-404. PMID: WOS:000082832600009. doi: 10.1016/s1072-7515(99)00185-4.

285. Rowley R. An effective approach for choosing an electronic health record. Radiol Manage. 2009 May-Jun;31(3):20-5; quiz 6-7. PMID: 21591489.

286. Rubin AD, McFerran VA. The Road to Electronic Health Records Is Paved With Operations. Am J Manag Care. 2010 Dec;16:E289-E92. PMID: WOS:000286881800001.

287. Rudin RS, Auerbach D, Zaydman M, Mehrotra A. Paying for telemedicine. Am J Manag Care. 2014;20(12):983-5. PMID: 25526386.

288. Ruotsalainen P. A cross-platform model for secure electronic health record communication. International Journal of Medical Informatics. 2004 Mar;73(3):291-5. PMID: WOS:000221013900013. doi: 10.1016/j.ijmedinf.2003.12.012.

289. Ruotsalainen P, Iivari AK, Doupi P. Finland's Strategy and Implementation of Citizens' Access to Health Information. In: Bos L, Blobel B, Marsh A, Carroll D, editors. Medical and Care Compunetics 5. Amsterdam: I O S Press; 2008. p. 379-85.

290. Ruotsalainen P, Manning B. A notary archive model for secure preservation and distribution of electrically signed patient documents. International Journal of Medical Informatics. 2007 May-Jun;76(5-6):449-53. PMID: WOS:000246320500020. doi: 10.1016/j.ijmedinf.2006.09.011.

291. Sabesan S, Allen DT, Caldwell P, Loh PK, Mozer R, Komesaroff PA, et al. Practical aspects of telehealth: establishing telehealth in an institution. Intern Med J. 2014 Feb;44(2):202-5. PMID: 24528818. doi: 10.1111/imj.12339.

292. Samantaray R, Njoku VO, Brunner JWM, Raghavan V, Kendall ML, Shih SC. Promoting Electronic Health Record Adoption Among Small Independent Primary Care Practices. Am J Manag Care. 2011 May;17(5):353-8. PMID: WOS:000291217500013.

293. Saranummi N, Ensio A, Laine M, Nykänen P, Itkonen P. National health IT services in Finland. Methods Inf Med. 2007;46(4):463-9. PMID: 17694242. doi: 10.1160/me9054.

294. Sarhan F. Get yourself connected. Nurs Stand. 2011 May 11-17;25(36):20-1. PMID: 21702350. doi: 10.7748/ns.25.36.20.s27.

295. Sato APS, Ferreira VLD, Tauil MD, Rodrigues LC, Barros MB, Martineli E, et al. Use of electronic immunization registry in the surveillance of adverse events following immunization. Rev Saude Publica. 2018;52:10. PMID: WOS:000424275900001. doi: 10.11606/s1518-8787.2018052000295.

296. Saurman E, Perkins D, Roberts R, Roberts A, Patfield M, Lyle D. Responding to mental health emergencies: Implementation of an innovative telehealth service in rural and remote New South Wales, Australia. J Emerg Nurs. 2011 Sep;37(5):453-9. PMID: WOS:000295007200011. doi: 10.1016/j.jen.2010.11.005.

297. Scott DM, Friesner DL, Undem T, Anderson G, Sem K, Peterson CD. Perceived sustainability of community telepharmacy in North Dakota. J Am Pharm Assoc (2003). 2017 May-Jun;57(3):362-8.e5. PMID: 28400253. doi: 10.1016/j.japh.2017.02.005.

298. Scott RE, Jennett P, Yeo M. Access and authorisation in a Glocal e-Health Policy context. International Journal of Medical Informatics. 2004 Mar;73(3):259-66. PMID: WOS:000221013900008. doi: 10.1016/j.ijmedinf.2003.11.020.

299. Séror AC. A Case Analysis of INFOMED: The Cuban National Health Care Telecommunications Network and Portal (Reprinted). MEDICC Rev. 2008 Win;10(1):37-42. PMID: WOS:000260567400010.

300. Serra HO, Maia AB, de Andrade PJA, Amaral JS, Maia LB, Silva RD, et al. Deployment of Brazil telehealth networks program in the state of Maranhao (Brazil). In: Chova LG, Martinez AL, Torres IC, editors. Edulearn14: 6th International Conference on Education and New Learning Technologies. Valenica: Iated-Int Assoc Technology Education a& Development; 2014. p. 7576-8.

301. Shah SGS, Fitton R, Hannan A, Fisher B, Young T, Barnett J. Accessing personal medical records online: A means to what ends? International Journal of Medical Informatics. 2015 Feb;84(2):111-8. PMID: WOS:000346422000003. doi: 10.1016/j.ijmedinf.2014.10.005.

302. Shane-McWhorter L, McAdam-Marx C, Lenert L, Petersen M, Woolsey S, Coursey JM, et al. Pharmacist-provided diabetes management and education via a telemonitoring program. J Am Pharm Assoc (2003). 2015 Sep-Oct;55(5):516-26. PMID: 26359961. doi: 10.1331/JAPhA.2015.14285.

303. Shao SC, Chan YY, Yang YHK, Lin SJ, Hung MJ, Chien RN, et al. The Chang Gung Research Database-A multi-institutional electronic medical records database for real-world epidemiological studies in Taiwan. Pharmacoepidemiology and Drug Safety. 2019 May;28(5):593-600. PMID: WOS:000472004500006. doi: 10.1002/pds.4713.

304. Shea CM, Tabriz AA, Turner K, North S, Reiter KL. Telestroke Adoption Among Community Hospitals in North Carolina: A Cross-Sectional Study. J Stroke Cerebrovasc Dis. 2018 Sep;27(9):2411-7. PMID: WOS:000441086000025. doi: 10.1016/j.jstrokecerebrovasdis.2018.04.032.

305. Sherer SA, Meyerhoefer CD, Peng LZ. Applying institutional theory to the adoption of electronic health records in the US. Information & Management. 2016 Jul;53(5):570-80. PMID: WOS:000379636300003. doi: 10.1016/j.im.2016.01.002.

306. Shin DY, Menachemi N, Diana M, Kazley AS, Ford EW. Payer mix and EHR adoption in hospitals. J Healthc Manag. 2012 Nov-Dec;57(6):435-48; discussion 49-50. PMID: 23297609.

307. Shu T, Liu HY, Goss FR, Yang W, Zhou L, Bates DW, et al. EHR adoption across China's tertiary hospitals: A cross-sectional observational study. International Journal of Medical Informatics. 2014 Feb;83(2):113-21. PMID: WOS:000329458600004. doi: 10.1016/j.ijmedinf.2013.08.008.

308. Sidek YH, Martins JT. Perceived critical success factors of electronic health record system implementation in a dental clinic context: An organisational management perspective. International Journal of Medical Informatics. 2017 Nov;107:88-100. PMID: WOS:000412684900011. doi: 10.1016/j.ijmedinf.2017.08.007.

309. Sifferlin A. Health care that reaches everyone. Time. 2016 Jul 11-18;188(2-3):47. PMID: 27526479.

310. Silva CM, Oliveira RD, Queiroz NR, dos Santos AD, Picthon A, de Souza C. Teleconsultation in angiology: Strategies to reduce the suppressed demand in the health system in Belo Horizonte City, Brazil. Macedo M, editor. Lisboa: Iadis-Int Assoc Development Information Society; 2013. 158-62 p. ISBN: 978-972-8939-87-8.

311. Sinha K. E-way to health: government bets big on telemedicine (India). Leadersh Health Serv. 2012;25(3):3. PMID: WOS:000218412300003. doi: 10.1108/lhs.2012.21125caa.003.

312. Sjogren LH, Tornqvist H, Schwieler A, Karlsson L. The potential of telemedicine: barriers, incentives and possibilities in the implementation, phase. J Telemed Telecare. 2001;7:S12-S3. PMID: WOS:000171462300005.

313. Skelton-Macedo MC, Haddad AE, Antoniazzi JH. Teledentistry in Brazil. In: Kumar S, editor. Teledentistry. New York: Springer; 2015. p. 87-100.

314. Slaper MR, Conkol K. mHealth Tools for the Pediatric Patient-Centered Medical Home. Pediatr Ann. 2014 Feb;43(2):E39-E43. PMID: WOS:000331875100003. doi: 10.3928/00904481-20140127-09.

315. Smeets O, Martin Abello K, Zijlstra-Vlasveld M, Boon B. [E-health within the Dutch mental health services: what is the current situation?]. Ned Tijdschr Geneeskd. 2014;158:A8589. PMID: 25515395.

316. Snyder W, Hewitt D. Ministry Embraces Telehealth Care. Health Prog. 2015 Jan-Feb;96(1):46-50. PMID: 26571875.

317. Soderberg K, Laventure M. Minnesota clinics' adoption, use and exchange of electronic health information. Minn Med. 2013 Sep;96(9):45-8. PMID: 24494362.

318. Spivak S, Spivak A, Cullen B, Meuchel J, Johnston D, Chernow R, et al. Telepsychiatry Use in U.S. Mental Health Facilities, 2010-2017. Psychiatr Serv. 2020 Feb 1;71(2):121-7. PMID: 31615370. doi: 10.1176/appi.ps.201900261.

319. Stanciu A. A blockchain based application scenario for active ageing. Boja C, Doinea M, Pocatilu P, Ciurea C, Batagan L, Velicanu A, et al., editors. Bucharest: Bucharest Univ Economic Studies-Ase; 2017. 206-11 p.

320. Stanimirovic D, Vintar M. Evaluating the Development of e-Health Project: The Case of Slovenia. Castelnovo W, Ferrari E, editors. Nr Reading: Acad Conferences Ltd; 2013. 491-8 p. ISBN: 978-1-909507-24-1.

321. Stanimirovic D, Vintar M. Development of eHealth at a national level - comparative aspects and mapping of general success factors. Inform Health Soc Care. 2014 Mar;39(2):140-60. PMID: WOS:000332815000005. doi: 10.3109/17538157.2013.872108.

322. Tahir D. Kaiser tests video visits to cut waits. Mod Healthc. 2015 Feb 23;45(8):29. PMID: 25823210.

323. Teixeira JG, de Pinho NF, Patricio L. Bringing service design to the development of health information systems: The case of the Portuguese national electronic health record. International Journal of Medical Informatics. 2019 Dec;132:8. PMID: WOS:000492149900011. doi: 10.1016/j.ijmedinf.2019.08.002.

324. Tierney WM, Were MC, Siika AM, Wools-Kaloustian K, Nyandiko WM, Sidle JE, et al. National health care financing strategies and implementing electronic health records in 3 East African countries. J Gen Intern Med. 2009 Apr;24:126-. PMID: WOS:000265382000336.

325. Tiwari A, Bhanu Y, Prasad BRR. The telemedicine experience of Care Hospitals. International Journal of Services Technology and Management. 2005;6(3-5):467-82. PMID: WOS:000216489200013. doi: 10.1504/ijstm.2005.007421.

326. Tschirch P, Walker G, Calvacca LT. Nursing in tele-mental health. J Psychosoc Nurs Ment Health Serv. 2006 May;44(5):20-7. PMID: WOS:000204017700012.

327. Tubaishat A. The Adoption of Electronic Health Records in Primary Healthcare Settings. Comput Inform Nurs. 2021 Jun 8;39(12):883-9. PMID: 34101659. doi: 10.1097/cin.0000000000000755.

328. Unita L, Giurgiu A. The Development of the e-Health System in Romania. Gasco M, editor. Nr Reading: Acad Conferences Ltd; 2012. 734-44 p. ISBN: 978-1-908272-42-3.

329. van der Heijden J. Teledermatology integrated in the Dutch national healthcare system. J Eur Acad Dermatol Venereol. 2010 May;24(5):615-6. PMID: WOS:000276491100022. doi: 10.1111/j.1468-3083.2010.03595.x.

330. Volk M, Sterle J, Sedlar U. Safety and Privacy Considerations for Mobile Application Design in Digital Healthcare. International Journal of Distributed Sensor Networks. 2015:12. PMID: WOS:000364074100001. doi: 10.1155/2015/549420.

331. Vujosevic S, Pucci P, Casciano M, Daniele A, Bini S, Berton M, et al. A decade-long telemedicine screening program for diabetic retinopathy in the north-east of Italy. J Diabetes Complications. 2017 Aug;31(8):1348-53. PMID: 28551296. doi: 10.1016/j.jdiacomp.2017.04.010.

332. Wade AJ, McCormack A, Roder C, McDonald K, Davies M, Scott N, et al. Aiming for elimination: Outcomes of a consultation pathway supporting regional general practitioners to prescribe direct-acting antiviral therapy for hepatitis C. Journal of Viral Hepatitis. 2018 Sep;25(9):1089-98. PMID: WOS:000442991000010. doi: 10.1111/jvh.12910.

333. Wan TTH. Impacts of health information technology adoption on patient and population health: Designing a health-finder system for elder care. Chova LG, Torres IC, Martinez AL, editors. Valenica: Iated-Int Assoc Technology Education & Development; 2011. 5601-11 p. ISBN: 978-84-614-7423-3.

334. Welch SS, McCullough A. Important changes in the MPFS. J Med Assoc Ga. 2016;105(1):32, 4. PMID: 27209680.

335. Westra B, Murphy J. ANA pledges to help patients improve care through use of electronic health information: association joins national Consumer eHealth Program launch. Comput Inform Nurs. 2011 Nov;29(11):620. PMID: 22076111. doi: 10.1097/01.Ncn.0000407668.25110.48.

336. Wetzel TG. Mobile health is in the regulatory crosshairs. Health Data Manag. 2012 Feb;20(2):44, 6, 8 passim. PMID: 22384739.

337. Whetstone M, Randeree E. Personal health records: addressing consumer needs for access. International Journal of Healthcare Technology and Management. 2008;9(3):258-74. PMID: WOS:000216221100005. doi: 10.1504/ijhtm.2008.018217.

338. Whetten J, van der Goes DN, Tran H, Moffett M, Semper C, Yonas H. Cost-effectiveness of Access to Critical Cerebral Emergency Support Services (ACCESS): a neuro-emergent telemedicine consultation program. J Med Econ. 2018;21(4):398-405. PMID: WOS:000427945100011. doi: 10.1080/13696998.2018.1426591.

339. Whitfield L. On telecare. Health Serv J. 2006 May 18;116(6006):suppl 2. PMID: 16749392.

340. Wilson JF. Making electronic health records meaningful. Ann Intern Med. 2009 Aug 18;151(4):293-6. PMID: 19687504. doi: 10.7326/0003-4819-151-4-200908180-00026.

341. Witt JD. Telemedicine extends healing ministry and offers innovative "patient safety net". Health Prog. 2007 May-Jun;88(3):64-5. PMID: 17533800.

342. Wong TK. Feeling the digital pulse: Consumer-centered approach to individual health profiling. Stud Health Technol Inform. 2006;122:9-17. PMID: 17102209.

343. Wood EW, Strauss RA, Janus C, Carrico CK. Telemedicine Consultations in Oral and Maxillofacial Surgery: A Follow-Up Study. J Oral Maxillofac Surg. 2016 Feb;74(2):262-8. PMID: 26501427. doi: 10.1016/j.joms.2015.09.026.

344. Wood PW, Boulanger P, Padwal RS. Home Blood Pressure Telemonitoring: Rationale for Use, Required Elements, and Barriers to Implementation in Canada. Can J Cardiol. 2017 May;33(5):619-25. PMID: WOS:000402460500009. doi: 10.1016/j.cjca.2016.12.018.

345. Wright D. Telemedicine and developing countries - A report of Study Group 2 of the ITU Development Sector. J Telemed Telecare. 1998;4:2-85. PMID: WOS:000074524700002.

346. Yacobazzo JEG, Rodriguez MJV. Electronic health record: confidentiality and privacy of clinical data. Revista Medica Del Uruguay. 2018 Dec;34(4):228-33. PMID: WOS:000451876400007. doi: 10.29193/tmu.34.4.6.

347. Yang NH, Dharmar M, Kuppermann N, Romano PS, Nesbitt TS, Hojman NM, et al. Appropriateness of disposition following telemedicine consultations in rural emergency departments. Pediatr Crit Care Med. 2015 Mar;16(3):e59-64. PMID: 25607743. doi: 10.1097/pcc.0000000000000337.

348. Yang XS, Barbieri JS, Kovarik CL. Cost analysis of a store-and-forward teledermatology consult system in Philadelphia. J Am Acad Dermatol. 2019 Sep;81(3):758-64. PMID: WOS:000480621300027. doi: 10.1016/j.jaad.2018.09.036.

349. Yoon D, Chang BC, Kang SW, Bae H, Park RW. Adoption of electronic health records in Korean tertiary teaching and general hospitals. International Journal of Medical Informatics. 2012 Mar;81(3):196-203. PMID: WOS:000300613600007. doi: 10.1016/j.ijmedinf.2011.12.002.

350. Yuan B, Li J, Wu P. The effectiveness of electronic health record promotion for healthcare providers in the United States since the Health Information Technology for Economic and Clinical Health Act: An empirical investigation. Int J Health Plann Manage. 2020 Oct 6. PMID: 33025639. doi: 10.1002/hpm.3085.

351. Yun EK, Park HA. Factors affecting the implementation of telenursing in Korea. Stud Health Technol Inform. 2006;122:657-9. PMID: 17102344.

352. Zamora Navas P, Montañez Heredia E, Nieto Orellana J, Gónzalez García C, Cano Obando L, Cárdenas Rebollo L, et al. Result of the implementation of telematic consultations in orthopaedic surgery and traumatology during COVID-19 laparoscopic surgery. Rev Esp Cir Ortop Traumatol. 2021 Jan-Feb;65(1):54-62. PMID: 33277229. doi: 10.1016/j.recot.2020.06.012.

353. Zigmond J. FCC has IT dreams in rural setting. Commissioner talks $400 million for telehealth. Mod Healthc. 2007 Nov 19;37(46):8-9. PMID: 18077851.

354. Zigmond J. Innovation won't wait. Despite law's uncertainty, CMS awards grants. Mod Healthc. 2012 May 14;42(20):12. PMID: 22670273.

355. Zimmermann GW. [In telephone consultation Nr. 3 GOÄ is also possible]. MMW Fortschr Med. 2015 Oct 8;157(17):32. PMID: 26759869.

356. Zolfo M, Arnould L, Huyst V, Lynen L. Telemedicine for HIV/AIDS care in low resource settings. In: Bos L, Marsh A, editors. Medical and Care Compunetics 2. Amsterdam: I O S Press; 2005. p. 18-22.

357. Zolfo M, Lynen L, Dierckx J, Colebunders R. Remote consultations and HIV/AIDS continuing education in low-resource settings. International Journal of Medical Informatics. 2006 Sep;75(9):633-7. PMID: WOS:000240045800002. doi: 10.1016/j.ijmedinf.2006.03.002.

358. Zorica Z. Telemedicine in Croatia. In: Stone CS, editor. Benchmarking Telemedicine: Improving Health Security in the Balkans. Amsterdam: Ios Press; 2017. p. 104-10.

359. Zwicker M, Seitz J, Wickramasinghe N. Adaptations for e-Kiosk Systems in Germany to Develop Barrier-Free Terminals for Handicapped Persons. Wickramasinghe N, Bali RK, Suomi R, Kirn S, editors. New York: Springer; 2012. 99-112 p. ISBN: 978-1-4614-1535-0.

Publications without concrete intervention

1. Balogun ZA, Reynolds TL, editors. Patient Portal Adoption, Use, and Satisfaction among U.S. Adults in Late-stage COVID-19 Pandemic. 2022 IEEE 10th International Conference on Healthcare Informatics (ICHI); 2022 11-14 June 2022.

2. Altpeter B. E-health as a component of holistic therapy optimization. Diabetologe. 2017 Jan;13(1):29-37. PMID: WOS:000393038200006. doi: 10.1007/s11428-016-0172-8.

3. McBride S, Delaney JM, Tietze M. Health Information Technology and Nursing. Am J Nurs. 2012 Aug;112(8):36-42. PMID: WOS:000306849700018. doi: 10.1097/01.NAJ.0000418095.31317.1b.

4. Chaudhari K, Karule PT. WiMAX network based E Health service And telemedicine applications for rural and remote populations in India. New York: Ieee; 2015. 398-406 p. ISBN: 978-1-4799-5097-3.

5. Sammani A, Jansen M, Linschoten M, Bagheri A, de Jonge N, Kirkels H, et al. UNRAVEL: big data analytics research data platform to improve care of patients with cardiomyopathies using routine electronic health records and standardised biobanking. Neth Heart J. 2019 Sep;27(9):426-34. PMID: WOS:000483711800005. doi: 10.1007/s12471-019-1288-4.

6. Allan R. Medical electronics: Coming: The era of telemedicine: Interactive audio/video telediagnosis is proven viable in both Government- and privately-funded experiments. IEEE Spectrum. 1976;13(12):31-6. doi: 10.1109/MSPEC.1976.6330440.

7. Marcun T. On-Line Data Exchange in Slovenian Healthcare and Health Insurance. In: Adlassnig KP, Blobel B, Mantas J, Masic I, editors. Medical Informatics in a United and Healthy Europe. Amsterdam: Ios Press; 2009. p. 48-52.

8. dos Santos AD, da Mata-Machado ATG, de Melo MDB, Sobrinho DF, Araujo LL, Silva EA, et al. Implementation of Telehealth Resources in Primary Care in Brazil and Its Association with Quality of Care. Telemed e-Health. 2019 Oct;25(10):996-1004. PMID: WOS:000489260800016. doi: 10.1089/tmj.2018.0166.

9. Posircaru DR, Serbanati LD. Integrating legacy medical applications in a standardized Electronic Health Record platform. 2015 E-Health and Bioengineering Conference. New York: Ieee; 2015.

10. Islam SMS, Tabassum R. Implementation of information and communication technologies for health in Bangladesh. Bulletin of the World Health Organization. 2015 Nov;93(11):806-9. PMID: WOS:000365244500018. doi: 10.2471/blt.15.153684.

11. Dullabh P, Hovey L. Large Scale Health Information Exchange: Implementation Experiences from Five States. In: Lehmann CU, Ammenwerth E, Nohr C, editors. Medinfo 2013: Proceedings of the 14th World Congress on Medical and Health Informatics, Pts 1 and 2. Amsterdam: Ios Press; 2013. p. 613-7.

12. Ford D, Harvey JB, McElligott J, King K, Simpson KN, Valenta S, et al. Leveraging health system telehealth and informatics infrastructure to create a continuum of services for COVID-19 screening, testing, and treatment. J Am Med Inform Assoc. 2020 Dec 9;27(12):1871-7. PMID: 32602884. doi: 10.1093/jamia/ocaa157.

13. Samofalov DA, Izhytska NV, Dragomyretska NM, Lyashenko AV. Information and communication technologies in public management of the healthcare institutions network during Covid-19 pandemics. Wiad Lek. 2020;73(11):2535-42. PMID: 33454697.

14. Bitton A, Poku M, Bates DW. Policy context and considerations for patient engagement with health information technology. Grando MA, Rozenblum R, Bates DW, editors. Berlin: Walter De Gruyter Gmbh; 2015. 75-90 p. ISBN: 978-1-61451-434-3; 978-1-61451-592-0.

15. Philip NY, Rodrigues J, Wang HG, Fong SJ, Chen J. Internet of Things for In-Home Health Monitoring Systems: Current Advances, Challenges and Future Directions. Ieee Journal on Selected Areas in Communications. 2021 Feb;39(2):300-10. PMID: WOS:000608975200002. doi: 10.1109/jsac.2020.3042421.

16. Anderson JG, Balas EA. Computerization of Primary Care in the United States. Int J Healthc Inf Syst Inf. 2006 Jul-Sep;1(3):1-23. PMID: WOS:000214478900001. doi: 10.4018/jhisi.2006070101.

17. Gusew N, Gerlach A, Bartkiewicz T, Goldapp M, Haux R, Heller U, et al. eHealth vision towards cooperative patient care--domain fields and architectural challenges of regional health care networks. Stud Health Technol Inform. 2010;160(Pt 1):386-90. PMID: 20841714.

18. Levy S. Diffusion of innovation: Telehealth for care at home. Stud Health Technol Inform. 2015;216:963. PMID: 26262265.

19. Bauerly BC, McCord RF, Hulkower R, Pepin D. Broadband Access as a Public Health Issue: The Role of Law in Expanding Broadband Access and Connecting Underserved Communities for Better Health Outcomes. J Law Med Ethics. 2019 Jun;47(2_suppl):39-42. PMID: 31298126. doi: 10.1177/1073110519857314.

20. Larsen SB, Sørensen NS, Petersen MG, Kjeldsen GF. Towards a shared service centre for telemedicine: Telemedicine in Denmark, and a possible way forward. Health Informatics J. 2016 Dec;22(4):815-27. PMID: 26261216. doi: 10.1177/1460458215592042.

21. Ibrahim FA, Pahuja E, Dinakaran D, Manjunatha N, Kumar CN, Math SB. The Future of Telepsychiatry in India. Indian J Psychol Med. 2020 Oct;42(5_SUPPL):112S-7S. PMID: WOS:000613802000021. doi: 10.1177/0253717620959255.

22. von Harbou K, Kordy H. [Psychiatric consultation via internet]. Psychiatr Prax. 2009 Oct;36(7):308-10. PMID: 19787564. doi: 10.1055/s-0029-1220458.

23. King J, Furukawa MF, Buntin MB. Geographic Variation in Ambulatory Electronic Health Record Adoption: Implications for Underserved Communities. Health Services Research. 2013 Dec;48(6):2037-59. PMID: WOS:000327392300014. doi: 10.1111/1475-6773.12078.

24. Minoi JL, Suhaili MR, Yeo AW. A Holistic Ecosystem for Rural mHealth Applications and Lesson Learnt. New York: Ieee; 2014. 1003-8 p. ISBN: 978-1-4799-4084-4.

25. Piliouras T, Yu PLR, Tian X, Zuo B, Yu S, Paulino J, et al., editors. Electronic health record systems: A current and future-oriented view. 2013 IEEE Long Island Systems, Applications and Technology Conference (LISAT); 2013 3-3 May 2013.

26. Alaboudi A, Atkins A, Sharpe B, Alzahrani M, Balkhair A, Sunbul T. Perceptions and Attitudes of Clinical Staff Towards Telemedicine Acceptance in Saudi Arabia. 2016 Ieee/Acs 13th International Conference of Computer Systems and Applications. New York: Ieee; 2016.

27. Hunter DJ. Trying to "Protect the NHS" in the United Kingdom. N Engl J Med. 2020 Dec 17;383(25):e136. PMID: 33176081. doi: 10.1056/NEJMp2032508.

28. Lantzsch H, Panteli D, Martino F, Stephani V, Seißler D, Püschel C, et al. Benefit Assessment and Reimbursement of Digital Health Applications: Concepts for Setting Up a New System for Public Coverage. Front Public Health. 2022;10:832870. PMID: 35530738. doi: 10.3389/fpubh.2022.832870.

29. Bell K. Public Policy and Health Informatics. Semin Oncol Nurs. 2018 May;34(2):184-7. PMID: WOS:000432470000010. doi: 10.1016/j.soncn.2018.03.010.

30. Picot J. Telemedicine and telehealth in Canada: Forty years of change in the use of information and communications technologies in a publicly administered health care system. Telemed J. 1998 Fal;4(3):199-205. PMID: WOS:000077259100002. doi: 10.1089/tmj.1.1998.4.199.

31. Sarwar MA, Bashir T, Shahzad O, Abbas A. Cloud Based Architecture to Implement Electronic Health Record (EHR) System in Pakistan. It Professional. 2019 May-Jun;21(3):49-54. PMID: WOS:000469338200008. doi: 10.1109/mitp.2018.2882437.

32. Luchenski SA, Reed JE, Marston C, Papoutsi C, Majeed A, Bell D. Patient and Public Views on Electronic Health Records and Their Uses in the United Kingdom: Cross-Sectional Survey. J Med Internet Res. 2013 Aug;15(8):13. PMID: WOS:000324620700020. doi: 10.2196/jmir.2701.

33. Ludewig G, Klose C, Hunze L, Matenaar S. [Digital health applications: statutory introduction of patient-centred digital innovations into healthcare]. Bundesgesundheitsblatt Gesundheitsforschung Gesundheitsschutz. 2021 Oct;64(10):1198-206. PMID: 34529096. doi: 10.1007/s00103-021-03407-9.

34. Elankavi R, Krishnamoorthy P, Jose JJ, Surekha R, editors. Smart IoT based Human Well-being Monitoring in Health Care System. 2022 3rd International Conference on Electronics and Sustainable Communication Systems (ICESC); 2022 17-19 Aug. 2022.

35. Dornan L, Pinyopornpanish K, Jiraporncharoen W, Hashmi A, Dejkriengkraikul N, Angkurawaranon C. Utilisation of Electronic Health Records for Public Health in Asia: A Review of Success Factors and Potential Challenges. Biomed Res Int. 2019;2019:7341841. PMID: 31360723. doi: 10.1155/2019/7341841.

36. Shaw J. Remote doctoring is only part of online health. J R Soc Med. 2012 Jan;105(1):41-2. PMID: 22275497. doi: 10.1258/jrsm.2011.110318.

37. Ohlund SE, Astrand B, Petersson G. Improving Interoperability in ePrescribing. Interact J Med Res. 2012 Nov 22;1(2):e17. PMID: 23612314. doi: 10.2196/ijmr.2089.

38. Yun Y, Huang W, Seitz J, Wickramasinghe N. Evaluation of e-Health in China. In: Zimmermann HD, Wickramasinghe N, Pucihar A, Gricar J, Babnik M, editors. 23rd Bled Econference Etrust: Implications for the Individual, Enterprises and Society; Kranj: Univ Maribor; 2010. p. 524-+.

39. Xu WC, Pan ZJ, Lu S, Zhang L. Regional Heterogeneity of Application and Effect of Telemedicine in the Primary Care Centres in Rural China. International Journal of Environmental Research and Public Health. 2020 Jun;17(12):15. PMID: WOS:000553566000001. doi: 10.3390/ijerph17124531.

40. Vosshoff A, Raum B, Ernestus W. Telematics in the public health sector. Where is the protection of health data? Bundesgesundheitsblatt-Gesund. 2015 Oct;58(10):1094-100. PMID: WOS:000361988400008. doi: 10.1007/s00103-015-2222-6.

41. Penjor K, Tshering G. Bhutan health telematics project: Overcoming the barriers. Kurokawa K, Nakajima I, Ishibashi Y, editors. New York: Ieee; 2004. 48-53 p. ISBN: 0-7803-8453-9.

42. Saksena N, Matthan R, Bhan A, Balsari S. Rebooting consent in the digital age: a governance framework for health data exchange. BMJ Glob Health. 2021 Jul;6(Suppl 5). PMID: 34301754. doi: 10.1136/bmjgh-2021-005057.

43. AlBar AM, Hoque MR. Patient Acceptance of e-Health Services in Saudi Arabia: An Integrative Perspective. Telemed J E Health. 2019 Sep;25(9):847-52. PMID: 30452333. doi: 10.1089/tmj.2018.0107.

44. Harno K, Nykanen P, Ohtonen J, Seppala A, Kopra K. Healthcare Information Exchange in Regional eHealth Networks Implications for Initiatives in Advancing Shared Care. Conley EC, Doarn C, HajjamElHassani A, editors. Los Alamitos: Ieee Computer Soc; 2009. 42-+ p. ISBN: 978-1-4244-3360-5.

45. Rajkovic P, Jankovic D, Tosic V. A Software Solution for Ambulatory Healthcare Facilities in the Republic of Serbia. New York: Ieee; 2009. 161-+ p. ISBN: 978-1-4244-5013-8.

46. Katuu S. Health Information Systems, eHealth Strategy, and the Management of Health Records: The Quest to Transform South Africa's Public Health Sector. Health Information Systems and the Advancement of Medical Practice in Developing Countries. Hersey: Igi Global; 2017. p. 237-61.

47. Becevic M, Sheets LR, Wallach E, McEowen A, Bass A, Mutrux ER, et al. Telehealth and Telemedicine in Missouri. Mo Med. 2020 May-Jun;117(3):228-34. PMID: 32636555.

48. Zachrison KS, Boggs KM, E MH, Espinola JA, Camargo CA. A national survey of telemedicine use by US emergency departments. J Telemed Telecare. 2020 Jun;26(5):278-84. PMID: 30558518. doi: 10.1177/1357633x18816112.

49. Mehrotra A, Bhatia RS, Snoswell CL. Paying for Telemedicine After the Pandemic. JAMA. 2021 Feb 2;325(5):431-2. PMID: 33528545. doi: 10.1001/jama.2020.25706.

50. Mahajan V, Singh T, Azad C. Using Telemedicine During the COVID-19 Pandemic. Indian Pediatr. 2020 Jul;57(7):658-61. PMID: WOS:000553026200011. doi: 10.1007/s13312-020-1895-6.

51. Moore C, Werner L, BenDor AP, Bailey M, Khan N. Accelerating Harmonization in Digital Health. World Health Popul. 2017;17(3):43-54. PMID: 29400273. doi: 10.12927/whp.2017.25306.

52. LeRouge CM, Gupta M, Corpart G, Arrieta A. Health System Approaches Are Needed To Expand Telemedicine Use Across Nine Latin American Nations. Health Affairs. 2019 Feb;38(2):212-21. PMID: WOS:000464094500008. doi: 10.1377/hlthaff.2018.05274.

53. Ghai B, Malhotra N, Bajwa SJS. Telemedicine for chronic pain management during COVID-19 pandemic. Indian J Anaesth. 2020 Jun;64(6):456-62. PMID: WOS:000538153100002. doi: 10.4103/ija.IJA_652_20.

54. Rea S, Pathak J, Savova G, Oniki TA, Westberg L, Beebe CE, et al. Building a robust, scalable and standards-driven infrastructure for secondary use of EHR data: The SHARPn project. Journal of Biomedical Informatics. 2012 Aug;45(4):763-71. PMID: WOS:000308258200019. doi: 10.1016/j.jbi.2012.01.009.

55. Lei J, Wen D, Zhang X, Li J, Lan H, Meng Q, et al. Enabling Health Reform through Regional Health Information Exchange: A Model Study from China. J Healthc Eng. 2017;2017:1053403. PMID: 29065565. doi: 10.1155/2017/1053403.

56. Piao C, Terrault NA, Sarkar S. Telemedicine: An Evolving Field in Hepatology. Hepatology Communications. 2019 May;3(5):716-21. PMID: WOS:000466288700011. doi: 10.1002/hep4.1340.

57. Wharton GA, Sood HS, Sissons A, Mossialos E. Virtual primary care: fragmentation or integration? Lancet Digit Health. 2019 Nov;1(7):e330-e1. PMID: 33323207. doi: 10.1016/s2589-7500(19)30152-9.

58. Makhni S, Atreja A, Sheon A, Van Winkle B, Sharp J, Carpenter N. The Broken Health Information Technology Innovation Pipeline: A Perspective from the NODE Health Consortium. Digit Biomark. 2017 Sep-Dec;1(1):64-72. PMID: 32095746. doi: 10.1159/000479017.

59. Narattharaksa K, Speece M, Newton C, Bulyalert D. Key success factors behind electronic medical record adoption in Thailand. Journal of Health Organization and Management. 2016;30(6):985-1008. PMID: WOS:000387175600012. doi: 10.1108/jhom-10-2014-0180.

60. Yellowlees P, Burke MM, Marks SL, Hilty DM, Shore JH. Emergency telepsychiatry. J Telemed Telecare. 2008;14(6):277-81. PMID: 18776070. doi: 10.1258/jtt.2008.080419.

61. Krausz M, Westenberg JN, Vigo D, Spence RT, Ramsey D. Emergency Response to COVID-19 in Canada: Platform Development and Implementation for eHealth in Crisis Management. JMIR Public Health Surveill. 2020 May 15;6(2):e18995. PMID: 32401218. doi: 10.2196/18995.

62. Friedman RH. Medicaid information technology architecture: an overview. Health Care Financ Rev. 2006 Winter;28(2):1-9. PMID: 17427840.

63. Geisthoff UW, Federspil PA, Sittel C, Plinkert PK. Telemedicine: Interactions between in- and outpatient services. HNO. 2002 Sep;50(9):812-21. PMID: WOS:000178233300005. doi: 10.1007/s00106-002-0684-y.

64. Dallest K, Strachan H, Flett G. The online Managed Knowledge Network that shares knowledge for eHealth in NHS Scotland. Stud Health Technol Inform. 2009;146:49-53. PMID: 19592807.

65. Popovich ML, Watkins T. Applied medical & care compunetics to public health disease surveillance and management: Leveraging external data sources--a key to public health preparedness. Stud Health Technol Inform. 2006;121:151-61. PMID: 17095812.

66. Zundel KM. Telemedicine: History, applications, and impact on librarianship. Bull Med Libr Assoc. 1996 Jan;84(1):71-9. PMID: WOS:A1996TV82500009.

67. Schiza EC, Kyprianou TC, Petkov N, Schizas CN. Proposal for an eHealth Based Ecosystem Serving National Healthcare. Ieee Journal of Biomedical and Health Informatics. 2019 May;23(3):1346-57. PMID: WOS:000467060400045. doi: 10.1109/jbhi.2018.2834230.

68. Demaerschalk BM. Remote Evaluation of the Patient With Acute Stroke. Continuum (Minneap Minn). 2017 Feb;23(1, Cerebrovascular Disease):259-67. PMID: 28157754. doi: 10.1212/con.0000000000000433.

69. Hughes MS, Apostolou A, Reilley B, Leston J, McCollum J, Iralu J. Electronic Health Record Reminders for Chlamydia Screening in an American Indian Population. Public Health Rep. 2021 May;136(3):320-6. PMID: 33301693. doi: 10.1177/0033354920970947.

70. Dwyer P, Hagerman V, Ingram CA, MacFarlane R, McCourt S. Atlantic Telehealth Knowledge Exchange. Telemed J E Health. 2004 Spr;10(1):93-101. PMID: WOS:000220909700013. doi: 10.1089/153056204773644634.

71. Gurung MS, Dorji G, Khetrapal S, Ra S, Babu GR, Krishnamurthy RS. Transforming health care through Bhutan's digital health strategy: progress to date. WHO South East Asia J Public Health. 2019 Sep;8(2):77-82. PMID: 31441441. doi: 10.4103/2224-3151.264850.

72. Barkman C, Weinehall L. Policymakers and mHealth: roles and expectations, with observations from Ethiopia, Ghana and Sweden. Global Health Action. 2017;10:7. PMID: WOS:000417200700006. doi: 10.1080/16549716.2017.1337356.

73. Lee YT, Park YT, Park JS, Yi BK. Association between Electronic Medical Record System Adoption and Healthcare Information Technology Infrastructure. Healthc Inform Res. 2018 Oct;24(4):327-34. PMID: WOS:000449771200010. doi: 10.4258/hir.2018.24.4.327.

74. Kheir D, Alshamsi RA, Alalwi ST, Alshammari RZ. "Webside" healthcare from medical interns' perspective: Telemedicine implementation and need for training. J Family Community Med. 2022 May-Aug;29(2):145-54. PMID: 35754750. doi: 10.4103/jfcm.jfcm_105_22.

75. Grain H, Robinson K, Torney B, Fraunholz B, Unnithan C. Healthcare in continuum for an ageing population - National self monitoring or remote offshore monitoring for Australia? Azevedo L, Londral AR, editors. Setubal: Insticc-Inst Syst Technologies Information Control & Communication; 2008. 266-+ p. ISBN: 978-989-8111-16-6.

76. Al Nuaimi N, AlShamsi A, Mohamed N, Al-Jaroodi J. e-Health Cloud Implementation Issues and Efforts. New York: Ieee; 2015. ISBN: 978-1-4799-6065-1.

77. Collen MF, Greenes RA. Medical Informatics: Past and Future. In: Collen MF, Ball MJ, editors. History of Medical Informatics in the United States. New York: Springer; 2015. p. 725-48.

78. Davis DC, Thakkar M. Perceived Level of Benefits and Risks of Core Functionalities of an EHR System. Int J Healthc Inf Syst Inf. 2006 Oct-Dec;1(4):55-67. PMID: WOS:000214480300005. doi: 10.4018/jhisi.2006100105.

79. Gensorowsky D, Witte J, Batram M, Greiner W. Market access and value-based pricing of digital health applications in Germany. Cost Eff Resour Alloc. 2022 Jun 13;20(1):25. PMID: 35698135. doi: 10.1186/s12962-022-00359-y.

80. Peine A, Paffenholz P, Martin L, Dohmen S, Marx G, Loosen SH. Telemedicine in Germany During the COVID-19 Pandemic: Multi-Professional National Survey. J Med Internet Res. 2020 Aug;22(8):11. PMID: WOS:000575058000001. doi: 10.2196/19745.

81. Elliott AF, Davidson A, Lum F, Chiang MF, Saaddine JB, Zhang X, et al. Use of electronic health records and administrative data for public health surveillance of eye health and vision-related conditions in the United States. Am J Ophthalmol. 2012 Dec;154(6 Suppl):S63-70. PMID: 23158225. doi: 10.1016/j.ajo.2011.10.002.

82. Junping Z, Zhenjiang Z, Huayuang G, Yi L, Wanguo X, Yunqi C, editors. E-health in China, our practice and exploration. 2009 Annual International Conference of the IEEE Engineering in Medicine and Biology Society; 2009 3-6 Sept. 2009.

83. Lantzsch H, Eckhardt H, Campione A, Busse R, Henschke C. Digital health applications and the fast-track pathway to public health coverage in Germany: challenges and opportunities based on first results. BMC Health Serv Res. 2022 Sep 21;22(1):1182. PMID: 36131288. doi: 10.1186/s12913-022-08500-6.

84. Nagoshi K, Watari T, Matsumura Y. Prospects of Hospital Information Systems and Patient Safety in Japan. Healthc Inform Res. 2022 Apr;28(2):105-11. PMID: WOS:000798662800002. doi: 10.4258/hir.2022.28.2.105.

85. Kasemsap K. Telemedicine and Electronic Health: Issues and Implications in Developing Countries. Health Information Systems and the Advancement of Medical Practice in Developing Countries. Hersey: Igi Global; 2017. p. 149-67.

86. Messer-Misak K, Reiter C. eHealth Networking Information Systems - The New Quality of Information Exchange. Stud Health Technol Inform. 2017;236:254-8. PMID: 28508804.

87. Marano A, Di Nicolantonio M. Ergonomic design in eHealthcare: a study case of eHealth technology system. In: Ahram T, Karwowski W, Schmorrow D, editors. 6th International Conference on Applied Human Factors and Ergonomics. Amsterdam: Elsevier Science Bv; 2015. p. 272-9.

88. Roberts J. Personal electronic health records: from biomedical research to people's health. Inform Prim Care. 2009;17(4):255-60. PMID: 20359404. doi: 10.14236/jhi.v17i4.745.

89. Everson J, Cross DA. Mind the gap: the potential of alternative health information exchange. Am J Manag Care. 2019 Jan;25(1):32-8. PMID: 30667609.

90. Fuad A, Putri SSM, Sitaresmi MN, Puspandari DA, editors. Financial Sources Options for Telemedicine Program within Universal Health Coverage (UHC) Era in Indonesia. 2018 1st International Conference on Bioinformatics, Biotechnology, and Biomedical Engineering - Bioinformatics and Biomedical Engineering; 2018 19-20 Oct. 2018.

91. Rahman M, Das S, Tazim MZ, Rana M, Tuhin RA, Das AK, editors. State of the Art of ICT based Telemedicine and E-health Services in Bangladesh. 2021 6th International Conference on Inventive Computation Technologies (ICICT); 2021 20-22 Jan. 2021.

92. Maher L, Craig A, Menezes G. A national survey of telemedicine in the Republic of Ireland. J Telemed Telecare. 2007;13(7):348-51. PMID: WOS:000250439500006. doi: 10.1258/135763307782215361.

93. Yesenofski L, Kromer S, Hitchings K. Nurses Leading the Transformation of Patient Care Through Telehealth. J Nurs Adm. 2015 Dec;45(12):650-6. PMID: 26565644. doi: 10.1097/nna.0000000000000279.

94. Gerke S, Stern AD, Minssen T. Germany's digital health reforms in the COVID-19 era: lessons and opportunities for other countries. npj Digit Med. 2020 Jul;3(1):6. PMID: WOS:000546929700001. doi: 10.1038/s41746-020-0306-7.

95. Zhao JP, Zhang ZJ, Guo HY, Li Y, Xue WG, Ren LZ, et al. E-health in China: Challenges, Initial Directions, and Experience. Telemed J E Health. 2010 Apr;16(3):344-9. PMID: WOS:000276909000011. doi: 10.1089/tmj.2009.0076.

96. Rizzato Lede DA, Pedernera FA, López E, Speranza CD, Guevel C, Maid JJ, et al. Argentinian Digital Health Strategy. Stud Health Technol Inform. 2020 Jun 16;270:818-22. PMID: 32570496. doi: 10.3233/shti200275.

97. Gholson J, Tennyson H. One System of Care, One Electronic Chart. Sarnikar S, Bennett D, Gaynor M, editors. Hersey: Igi Global; 2013. 55-69 p. ISBN: 978-1-4666-2702-4; 978-1-4666-2671-3.

98. Hochmuth A, Exner AK, Dockweiler C. [Implementation and participatory design of digital health interventions]. Bundesgesundheitsblatt Gesundheitsforschung Gesundheitsschutz. 2020 Feb;63(2):145-52. PMID: 31938837. doi: 10.1007/s00103-019-03079-6.

99. Gerli P, Arakpogun EO, Elsahn Z, Olan F, Prime KS. Beyond contact-tracing: The public value of eHealth application in a pandemic. Gov Inf Q. 2021 Jul;38(3):9. PMID: WOS:000668202500002. doi: 10.1016/j.giq.2021.101581.

100. Jian WS, Hsuc CY, Hao TH, Wen HC, Hsu MH, Lee YL, et al. Building a portable data and information interoperability infrastructure - framework for a standard Taiwan electronic medical record template. Computer Methods and Programs in Biomedicine. 2007 Nov;88(2):102-11. PMID: WOS:000250840300002. doi: 10.1016/j.cmpb.2007.07.014.

101. Hoffman DA. Increasing access to care: telehealth during COVID-19. J Law Biosci. 2020 Jan-Jun;7(1):lsaa043. PMID: 32843985. doi: 10.1093/jlb/lsaa043.

102. Barlow J, Bayer S, Curry R. Implementing complex innovations in fluid multi-stakeholder environments: Experiences of 'telecare'. Technovation. 2006 Mar;26(3):396-406. PMID: WOS:000236083700012. doi: 10.1016/j.technovation.2005.06.010.

103. Vimalachandran P, Wang H, Zhang Y, Zhuo G. The Australian PCEHR System: Ensuring Privacy and Security through an Improved Access Control Mechanism. Eai Endorsed Transactions on Scalable Information Systems. 2016;3(8):8. PMID: WOS:000407179000003. doi: 10.4108/eai.9-8-2016.151633.

104. Whitten P, Buis L, Love B. Physician-patient e-visit programs - Implementation and appropriateness. Disease Management & Health Outcomes. 2007;15(4):207-14. PMID: WOS:000249603200002. doi: 10.2165/00115677-200715040-00002.

105. Mirza H, El-Masri S. National Electronic Medical Records integration on Cloud Computing System. In: Lehmann CU, Ammenwerth E, Nohr C, editors. Medinfo 2013: Proceedings of the 14th World Congress on Medical and Health Informatics, Pts 1 and 2. Amsterdam: Ios Press; 2013. p. 1219-.

106. Perlman SE, McVeigh KH, Thorpe LE, Jacobson L, Greene CM, Gwynn RC. Innovations in Population Health Surveillance: Using Electronic Health Records for Chronic Disease Surveillance. Am J Public Health. 2017 Jun;107(6):853-7. PMID: 28426302. doi: 10.2105/ajph.2017.303813.

107. Grechenig T, Tappeiner B, Wujciow A. Challenging interoperability and bandwidth issues in national e-Health strategies by a bottom-up approach: Establishing a performant IT infrastructure network in a Middle East State. New York: Ieee; 2008. 148-+ p. ISBN: 978-1-4244-2280-7.

108. Brönneke JB, Debatin JF. [Digitalization of healthcare and its effects on quality of care]. Bundesgesundheitsblatt Gesundheitsforschung Gesundheitsschutz. 2022 Mar;65(3):342-7. PMID: 35181795. doi: 10.1007/s00103-022-03493-3.

109. Schiza EC, Neokleous KC, Petkov N, Schizas CN. A patient centered electronic health: eHealth system development. Technology and Health Care. 2015;23(4):509-22. PMID: WOS:000358797600012. doi: 10.3233/thc-150919.

110. Coats B, Acharya S. The forecast for electronic health record access: partly cloudy. Proceedings of the 2013 IEEE/ACM International Conference on Advances in Social Networks Analysis and Mining; Niagara, Ontario, Canada: Association for Computing Machinery; 2013. p. 937–42.

111. AlZghoul MM, Al-Taee MA, Al-Taee AM. Towards Nationwide Electronic Health Record System in Jordan. 2016 13th International Multi-Conference on Systems, Signals & Devices. New York: Ieee; 2016. p. 650-5.

112. Alanezi F. Factors affecting the adoption of e-health system in the Kingdom of Saudi Arabia. Int Health. 2020 Nov 10. PMID: 33170217. doi: 10.1093/inthealth/ihaa091.

113. Harno K, Ruotsalainen P, Nykanen P, Kopra K. Migration from Regional to a National eHealth Network. Berntzen L, Smedberg A, editors. Los Alamitos: Ieee Computer Soc; 2008. 107-+ p. ISBN: 978-1-4244-4229-4.

114. Davis D, Qu XL, Yuan XH. Evaluation of WorldVistA and OpenEMR for PCMH Care. Southeastcon 2017. New York: Ieee; 2017.

115. Littman-Quinn R, Mibenge C, Antwi C, Chandra A, Kovarik CL. Implementation of m-health applications in Botswana: telemedicine and education on mobile devices in a low resource setting. J Telemed Telecare. 2013 Feb;19(2):120-5. PMID: WOS:000321901300011. doi: 10.1177/1357633x12474746.

116. Ackerman M, Locatis C. Advanced networks and computing in healthcare. Journal of the American Medical Informatics Association. 2011 Jul;18(4):523-8. PMID: WOS:000292061700027. doi: 10.1136/amiajnl-2010-000054.

117. Riazi H, Jafarpour M, Bitaraf E. Towards National eHealth Implementation - A Comparative Study on WHO/ITU National eHealth Strategy Toolkit In Iran. In: Lovis C, Seroussi B, Hasman A, PapeHaugaard L, Saka O, Andersen SK, editors. E-Health - for Continuity of Care. Amsterdam: Ios Press; 2014. p. 246-50.

118. Abaza H, Marschollek M. mHealth Application Areas and Technology Combinations. Methods of Information in Medicine. 2017;56:E105-E22. PMID: WOS:000413013300006. doi: 10.3414/me17-05-0003.

119. Bukowski M, Farkas R, Beyan O, Moll L, Hahn H, Kiessling F, et al. Implementation of eHealth and AI integrated diagnostics with multidisciplinary digitized data: are we ready from an international perspective? Eur Radiol. 2020 Oct;30(10):5510-24. PMID: WOS:000530780700002. doi: 10.1007/s00330-020-06874-x.

120. Curtis LH, Brown J, Platt R. Four health data networks illustrate the potential for a shared national multipurpose big-data network. Health Aff (Millwood). 2014 Jul;33(7):1178-86. PMID: 25006144. doi: 10.1377/hlthaff.2014.0121.

121. Garai A, Pentek I, Adamko A. Revolutionizing Healthcare with IoT and Cognitive, Cloud-based Telemedicine. Acta Polytechnica Hungarica. 2019;16(2):163-81. PMID: WOS:000466165100010.

122. Dobbs D, Trebatoski M, Revere D. The Northwest Public Health Information Exchange's Accomplishments in Connecting a Health Information Exchange with Public Health. Online J Public Health Inform. 2010;2(2). PMID: 23569585. doi: 10.5210/ojphi.v2i2.3210.

123. Bali S, Informat Resources Management A. Enhancing the Reach of Health Care Through Telemedicine: Status and New Possibilities in Developing Countries. Hersey: Igi Global; 2018. 1382-97 p. ISBN: 978-1-5225-3927-8; 978-1-5225-3926-1.

124. Pedrosa T, Lopes RP, Santos JC, Costa C, Oliveira JL. Hybrid electronic health records. Traver V, Fred A, Filipe J, Gamboa H, editors. Setubal: Scitepress; 2011. 571-+ p. ISBN: 978-989-8425-34-8.

125. Kwankam SY. Successful partnerships for international collaboration in e-health: the need for organized national infrastructures. Bull World Health Organ. 2012 May 1;90(5):395-7. PMID: 22589576. doi: 10.2471/blt.12.103770.

126. Lee M, Heo E, Lim H, Lee JY, Weon S, Chae H, et al. Developing a Common Health Information Exchange Platform to Implement a Nationwide Health Information Network in South Korea. Healthc Inform Res. 2015 Jan;21(1):21-9. PMID: WOS:000219435300004. doi: 10.4258/hir.2015.21.1.21.

127. Zailani S, Gilani MS, Nikbin D, Iranmanesh M. Determinants of telemedicine acceptance in selected public hospitals in Malaysia: clinical perspective. J Med Syst. 2014 Sep;38(9):111. PMID: 25038891. doi: 10.1007/s10916-014-0111-4.

128. Suselj M, Zuffada R. Netc@rds for e-EHIC - a Step Towards the Introduction of the European Health Insurance Card. In: Cunningham P, Cunningham M, editors. Innovation and the Knowledge Economy: Issues, Applications, Case Studies, Pts 1 & 2. Amsterdam: Ios Press; 2005. p. 445-51.

129. Busey JC, Michael P. Telehealth--opportunities and pitfalls. J Am Diet Assoc. 2008 Aug;108(8):1296-301. PMID: 18656568. doi: 10.1016/j.jada.2008.05.002.

130. Williams R, Sheikh A, Franklin BD, Krasuska M, Nguyen HT, Hinder S, et al. Using Blueprints to promote interorganizational knowledge transfer in digital health initiatives-a qualitative exploration of a national change program in English hospitals. J Am Med Inform Assoc. 2021 Jul 14;28(7):1431-9. PMID: 33706378. doi: 10.1093/jamia/ocab020.

131. Hansen DP, Gurney P, Morgan G, Barraclough B. The Australian e-Health Research Centre: enabling the health care information and communication technology revolution. Med J Aust. 2011 Feb;194(4):S5-S7. PMID: WOS:000288046000001.

132. Chen NY. Stakeholder Power Analysis of the Facilitators and Barriers for Telehealth Solution Implementation in China: A Qualitative Study of Individual Users in Beijing and Interviews With Institutional Stakeholders. JMIR Form Res. 2022 Jan;6(1):17. PMID: WOS:000854067700025. doi: 10.2196/19448.

133. Peebles J. Hawaii's telehealth reimbursement policy: Our successful collaborative community approach. Nelson R, Gelish A, Mun SK, editors. Los Alamitos: Ieee Computer Soc; 1998. 192-6 p. ISBN: 0-8186-8667-7.

134. El Khaddar MA, Harroud H, Boulmalf M, Elkoutbi M, Habbani A. Emerging Wireless Technologies In E-Health. In: Essaaidi M, Zaz Y, editors. 2012 International Conference on Multimedia Computing and Systems. New York: Ieee; 2012. p. 440-5.

135. Lopez E, Berlin M, Stein R, Cozzi E, Bermudez A, Mandirola Brieux H, et al. Results of the Use of the Teleconsultation Platform After 2 Months of Implementation. Stud Health Technol Inform. 2020 Jun 16;270:1377-8. PMID: 32570667. doi: 10.3233/shti200450.

136. Meyers L, Gibbs D, Thacker M, LaFile L. Building a Telehealth Network Through Collaboration The Story of the Nebraska Statewide Telehealth Network. Crit Care Nurs Q. 2012 Oct-Dec;35(4):346-52. PMID: WOS:000217400500007. doi: 10.1097/CNQ.0b013e318266bed1.

137. Ahmed M, Ahamad M, Jaiswal T. Augmenting security and accountability within the eHealth Exchange. Ibm Journal of Research and Development. 2014 Jan-Feb;58(1):11. PMID: WOS:000339882000009. doi: 10.1147/jrd.2013.2288068.

138. Baldoni S, Pallotta G, Traini E, Sagaro GG, Nittari G, Amenta F. A survey on feasibility of telehealth services among young Italian pharmacists. Pharmacy Practice-Granada. 2020 Jul-Sep;18(3):11. PMID: WOS:000581814200002. doi: 10.18549/PharmPract.2020.3.1926.

139. Stroetmann KA, Novell TG, Gutierrez C. eSurveillance for Public Health - An Implementation Approach for sub-Saharan Africa. New York: Ieee; 2017.

140. Dansky KH. Marketing telehealth to align with strategy. J Healthc Manag. 2005 Jan-Feb;50(1):19-30. PMID: WOS:000226458100006.

141. McSpadden C, Spiro S. Health information technology: how we got where we are. Consult Pharm. 2011 Mar;26(3):156-64, 67-9. PMID: 21402516. doi: 10.4140/TCP.n.2011.156.

142. Scandurra I, Liljequist D. Ten Demands of Improved Usability in eHealth and Some Progress - Co-creation by Health and Social Care Professionals. In: Sermeus W, Procter PM, Weber P, editors. Nursing Informatics 2016: Ehealth for All: Every Level Collaboration - from Project to Realization. Amsterdam: Ios Press; 2016. p. 227-31.

143. Currie WL, Finnegan DJ. The policy-practice nexus of electronic health records adoption in the UK NHS An institutional analysis. Journal of Enterprise Information Management. 2011;24(2):146-+. PMID: WOS:000212894300005. doi: 10.1108/17410391111106284.

144. Kang GX, Zhang L, Li SH, Zhang P, Boussakta S. Case study of applying wireless technologies into healthcare industry in China and UK. Stephanidis C, editor. Berlin: Springer-Verlag Berlin; 2007. 874-+ p. ISBN: 978-3-540-73282-2.

145. Balog J. A Three-Step Approach for Creating Successful Electronic Immunization Record Exchanges between Clinical Practice and Public Health. Online J Public Health Inform. 2012;4(3). PMID: 23569651. doi: 10.5210/ojphi.v4i3.4290.

146. Hansen D, Golbeck AL, Noblitt V, Pinsonneault J, Christner J. Cost factors in implementing telemonitoring programs in rural home health agencies. Home Healthc Nurse. 2011 Jun;29(6):375-82. PMID: 21633229. doi: 10.1097/NHH.0b013e31821b736f.

147. Wolf DM, Kapadia A, Kintzel J, Anton BB. Nurses Using Futuristic Technology in Today's Healthcare Setting. In: Saranto K, Brennan PF, Park HA, Tallberg M, Ensio A, editors. Connecting Health and Humans. Amsterdam: I O S Press; 2009. p. 59-63.

148. Nøhr C, Villumsen S, Bernth Ahrenkiel S, Hulbæk L. Monitoring Telemedicine Implementation in Denmark. Stud Health Technol Inform. 2015;216:497-500. PMID: 26262100.

149. Gregor-Haack J. Reimbursement of health apps by the German statutory health insurance. Bundesgesundheitsblatt-Gesund. 2018 Mar;61(3):328-33. PMID: WOS:000426098000012. doi: 10.1007/s00103-018-2689-z.

150. Gul O, Al-Qutayri M, Yeun CY, Vu QH. Framework of a National Level Electronic Health Record System. New York: Ieee; 2012. 60-5 p. ISBN: 978-1-4673-4415-9.

151. Trief PM, Izquierdo R, Eimicke JP, Teresi JA, Goland R, Palmas W, et al. Adherence to diabetes self care for white, African-American and Hispanic American telemedicine participants: 5 year results from the IDEATel project. Ethn Health. 2013;18(1):83-96. PMID: 22762449. doi: 10.1080/13557858.2012.700915.

152. Pai RR, Alathur S. Mobile Health System Framework in India. Proceedings of the 20th Annual International Conference on Digital Government Research; Dubai, United Arab Emirates: Association for Computing Machinery; 2019. p. 186–95.

153. Walden A, Kemp AS, Larson-Prior LJ, Kim T, Gan J, McCoy H, et al. Establishing a digital health platform in an academic medical center supporting rural communities. J Clin Transl Sci. 2020 Apr 28;4(5):384-8. PMID: 33244426. doi: 10.1017/cts.2020.11.

154. Yun Sik K, editor. International standards for building Electronic Health Record (EHR). Proceedings of 7th International Workshop on Enterprise networking and Computing in Healthcare Industry, 2005 HEALTHCOM 2005; 2005 23-25 June 2005.

155. Khanna N, Klyushnenkova E, Rao V, Siegel N, Wolfe S. Electronic referrals to the tobacco Quitline: implementation strategies in a large health system to optimize delivery of tobacco cessation to patients. Transl Behav Med. 2021 May 25;11(5):1107-14. PMID: 33410484. doi: 10.1093/tbm/ibaa094.

156. Larbi D, Anthun KS, Asah FN, Debrah O, Antypas K, editors. Assessing Strategic Priority Factors in eHealth Policies of Four African Countries. 2022 IST-Africa Conference (IST-Africa); 2022 16-20 May 2022.

157. Al-Qirim NAY. Critical success factors for strategic telemedicine planning in New Zealand. Telemed J E Health. 2005 Oct;11(5):600-7. PMID: WOS:000233271300028. doi: 10.1089/tmj.2005.11.600.

158. Mwogi T, Were MC. Setting the Agenda for Personal Health Records in Low- and Middle-Income Countries. Stud Health Technol Inform. 2017;245:1234. PMID: 29295321.

159. Seerainer C, Sabutsch SW. eHealth Terminology Management in Austria. Stud Health Technol Inform. 2016;228:426-30. PMID: 27577418.

160. Ivanovic A, Rakovic P. E-health Card Information System: Case Study Health Insurance Fund of Montenegro. In: Jozwiak L, Stojanovic R, Lutovac B, Jurisic D, editors. 2019 8th Mediterranean Conference on Embedded Computing. New York: Ieee; 2019. p. 695-9.

161. Delgado A, Gorry C. Cuba's National eHealth Strategy. MEDICC Rev. 2008 Jan;10(1):6-8. PMID: 21483348.

162. Androuchko L, Nakajima I, editors. Developing countries and e-health services. Proceedings 6th International Workshop on Enterprise Networking and Computing in Healthcare Industry - Healthcom 2004 (IEEE Cat No04EX842); 2004 29-29 June 2004.

163. Lynch K, Kendall M, Shanks K, Haque A, Jones E, Wanis MG, et al. The Health IT Regional Extension Center Program: evolution and lessons for health care transformation. Health Serv Res. 2014 Feb;49(1 Pt 2):421-37. PMID: 24359032. doi: 10.1111/1475-6773.12140.

164. Marzuki N, Ismail S, Al-Sadat N, Ehsan FZ, Chan CK, Ng CW. Integrating Information and Communication Technology for Health Information System Strengthening: A Policy Analysis. Asia Pac J Public Health. 2015 Nov;27(8):86S-93S. PMID: WOS:000363204800012. doi: 10.1177/1010539515590180.

165. Knight AW, Szucs C, Dhillon M, Lembke T, Mitchell C. The eCollaborative: using a quality improvement collaborative to implement the National eHealth Record System in Australian primary care practices. Int J Qual Health Care. 2014 Aug;26(4):411-7. PMID: WOS:000342220800010. doi: 10.1093/intqhc/mzu059.

166. Barnett ML, Ray KN, Souza J, Mehrotra A. Trends in Telemedicine Use in a Large Commercially Insured Population, 2005-2017. JAMA. 2018 Nov 27;320(20):2147-9. PMID: 30480716. doi: 10.1001/jama.2018.12354.

167. Park AJ, Weintraub GS, Asgari MM. Leveraging the electronic health record to improve dermatologic care delivery: The importance of finding structure in data. J Am Acad Dermatol. 2020 Mar;82(3):773-5. PMID: WOS:000513949800066. doi: 10.1016/j.jaad.2019.10.064.

168. Ge X, Paige RF, McDermid JA. Domain analysis on an electronic health records system. Proceedings of the First International Workshop on Feature-Oriented Software Development; Denver, Colorado, USA: Association for Computing Machinery; 2009. p. 49–54.

169. Avanesova AA, Shamliyan TA. Worldwide implementation of telemedicine programs in association with research performance and health policy. Health Policy Technol. 2019 Jun;8(2):179-91. PMID: WOS:000472696800011. doi: 10.1016/j.hlpt.2019.04.001.

170. Madden J, Khan A, editors. Adaption and Implication of Telemedicine in Rural Healthcare Delivery throughout the United States. 2017 International Conference on Computational Science and Computational Intelligence (CSCI); 2017 14-16 Dec. 2017.

171. Martin KS, Monsen KA, Bowles KH. The Omaha system and meaningful use: applications for practice, education, and research. Comput Inform Nurs. 2011 Jan-Feb;29(1):52-8. PMID: 21099545. doi: 10.1097/NCN.0b013e3181f9ddc6.

172. Dluhopolskyi O, Dluhopolska T, Farion A, Karp I, Zhukovska A, Kryvokulska N. The Implementation of the Ehealth System and Anticorruption Reforms (Case of EU Countries for Ukraine). New York: Ieee; 2019. 346-9 p. ISBN: 978-1-7281-0449-2.

173. Pai MMM, Ganiga R, Pai RM, Sinha RK. Standard electronic health record (EHR) framework for Indian healthcare system. Health Services and Outcomes Research Methodology. 2021:24. PMID: WOS:000612289900001. doi: 10.1007/s10742-020-00238-0.

174. Zhao X, Innes KE, Bhattacharjee S, Dwibedi N, LeMasters TM, Sambamoorthi U. Facility and state-level factors associated with telemental health (TMH) adoption among mental health facilities in the United States. J Telemed Telecare. 2019 Sep 2:1357633x19868902. PMID: 31475879. doi: 10.1177/1357633x19868902.

175. Butane L, Paulins N, Latvia Univ A. E-health progress in Latvia. Jelgava: Latvia Univ Agriculture; 2013. 231-7 p.

176. Aanestad M, Grisot M, Hanseth O, Vassilakopoulou P. Information Infrastructures for eHealth. In: Aanestad M, Grisot M, Hanseth O, Vassilakopoulou P, editors. Information Infrastructures within European Health Care: Working with the Installed Base. Cham (CH): Springer

1. Copyright 2017, The Author(s). 2017. p. 11-23.

177. Essen A, Stern AD, Haase CB, Car J, Greaves F, Paparova D, et al. Health app policy: international comparison of nine countries' approaches. npj Digit Med. 2022 Mar;5(1):10. PMID: WOS:000770626200002. doi: 10.1038/s41746-022-00573-1.

178. Manocchia A. Telehealth: Enhancing Care through Technology. R I Med J (2013). 2020 Feb 3;103(1):18-20. PMID: 32013298.

179. Strachan H, Dallest K. An Electronic Portal to Support Using Information to Improve Healthcare. In: Saranto K, Brennan PF, Park HA, Tallberg M, Ensio A, editors. Connecting Health and Humans. Amsterdam: I O S Press; 2009. p. 663-7.

180. Gilman M, Stensland J. Telehealth and Medicare: payment policy, current use, and prospects for growth. Medicare Medicaid Res Rev. 2013;3(4). PMID: 24834368. doi: 10.5600/mmrr.003.04.a04.

181. Moore A, Parr G, Logan M, Neely H, Roesner D, Durer U. Developing a European internet and kiosk-based health information system. J Med Internet Res. 2001;3(1):8. PMID: WOS:000207624300005. doi: 10.2196/jmir.3.1.e6.

182. Kaufman JH, Eiron I, Deen G, Ford DA, Smith E, Knoop S, et al. From regional healthcare information organizations to a national healthcare information infrastructure. Perspect Health Inf Manag. 2005 Dec 6;2:10. PMID: 18066378.

183. Julien HM, Eberly LA, Adusumalli S. Telemedicine and the Forgotten America. Circulation. 2020 Jul 28;142(4):312-4. PMID: 32525712. doi: 10.1161/circulationaha.120.048535.

184. Tansel AU. Innovation through Patient Health Records. In: Eren E, editor. Second International Conference on Leadership, Technology and Innovation Management. Amsterdam: Elsevier Science Bv; 2013. p. 183-8.

185. Zachrison KS, Hayden EM, Schwamm LH, Espinola JA, Sullivan AF, Boggs KM, et al. Characterizing New England Emergency Departments by Telemedicine Use. West J Emerg Med. 2017 Oct;18(6):1055-60. PMID: 29085537. doi: 10.5811/westjem.2017.8.34880.

186. Milenkovic MJ, Radojicic Z, Milenkovic D, Vukmirovic D. Applying electronic documents in development of the healthcare information system in the Republic of Serbia. Comput Sci Inf Syst. 2009 Dec;6(2):111-26. PMID: WOS:000272897000005. doi: 10.2298/csis0902111J.

187. Mihalas GI, Farcas DD, Lungeanu D, Focsa M. Building eHealth National Strategies - The Romanian Experience. In: Adlassnig KP, Blobel B, Mantas J, Masic I, editors. Medical Informatics in a United and Healthy Europe. Amsterdam: Ios Press; 2009. p. 33-7.

188. Whiddett D, Hunter I, McDonald B, Norris T, Waldon J. Consent and widespread access to personal health information for the delivery of care: a large scale telephone survey of consumers' attitudes using vignettes in New Zealand. BMJ Open. 2016 Aug 23;6(8):e011640. PMID: 27554103. doi: 10.1136/bmjopen-2016-011640.

189. Gornall J. Does telemedicine deserve the green light? BMJ. 2012 Jul 10;345:e4622. PMID: 22782733. doi: 10.1136/bmj.e4622.

190. Kautsch M, Lichon M, Matuszak N. Development of publicly funded eHealth in Poland: Barriers and opportunities. Economics & Sociology. 2016;9(3):28-40. PMID: WOS:000387911700003. doi: 10.14254/2071-789x.2016/9-3/3.

191. Rensmann B, Smits M. Analyzing the Added Value of Electronic Intermediaries in the Dutch Health Care Sector. Hampe JF, Swatman MC, Gricar J, Pucihar A, Lenart G, editors. Kranj: Univ Maribor; 2008. 211-22 p. ISBN: 978-961-232-217-5.

192. Al-Fadhli AA, Othman M, Ramasamy A, Rashed A. Tele-Health In Yemen: an Overview. In: Jamaludin Z, ChePa N, Ishak WHW, Zaibon SB, editors. Proceedings of the 5th International Conference on Computing & Informatics. Sintok: Univ Utari Malaysia-Uum; 2015. p. 121-6.

193. Soar J, Seo Y. Health and aged care enabled by information technology. In: Weller NJ, Rattan SIS, editors. Healthy Aging and Longevity. Oxford: Blackwell Publishing; 2007. p. 154-61.

194. Ng HS, Sim ML, Tan CM, Wong CC. Wireless technologies for telemedicine. Bt Technology Journal. 2006 Apr;24(2):130-7. PMID: WOS:000238859200019. doi: 10.1007/s10550-006-0050-9.

195. Zeng K, Bernardo SN, Havins WE. The Use of Digital Tools to Mitigate the COVID-19 Pandemic: Comparative Retrospective Study of Six Countries. JMIR Public Health Surveill. 2020 Dec 23;6(4):e24598. PMID: 33302255. doi: 10.2196/24598.

196. Dolan SB, Alao ME, Mwansa FD, Lymo DC, Bulula N, Carnahan E, et al. Perceptions of factors influencing the introduction and adoption of electronic immunization registries in Tanzania and Zambia: a mixed methods study. Implement Sci Commun. 2020;1:38. PMID: 32885195. doi: 10.1186/s43058-020-00022-8.

197. Balch D. Developing a National Inventory of Telehealth Resources for Rapid and Effective Emergency Medical Care: a white paper developed by the American Telemedicine Association Emergency Preparedness and Response Special Interest Group. Telemed J E Health. 2008 Aug;14(6):606-10. PMID: 18729762. doi: 10.1089/tmj.2007.0127.

198. West DM, Miller EA. The digital divide in public e-health: barriers to accessibility and privacy in state health department websites. J Health Care Poor Underserved. 2006 Aug;17(3):652-67. PMID: 16960328. doi: 10.1353/hpu.2006.0115.

199. Angood PB. Telemedicine, the Internet, and world wide web: Overview, current status, and relevance to surgeons. World J Surg. 2001 Nov;25(11):1449-57. PMID: WOS:000172316800016. doi: 10.1007/s00268-001-0130-4.

200. Ramos V. New Wireless Solutions for Health and Welfare. New York: Ieee; 2009. 55-8 p. ISBN: 978-1-4244-4066-5.

201. Mykkänen J, Korhonen M, Porrasmaa J, Tuomainen T, Ensio A. A National Study of eHealth Standardization in Finland - Goals and Recommendations. In: Kuhn KA, Warren JR, Leong TY, editors. Medinfo 2007: Proceedings of the 12th World Congress on Health. Amsterdam: Ios Press; 2007. p. 469-+.

202. Skelly JR, O'Connor T. Guidelines for the use of the Attend Anywhere Platform for Telecommunications within the Pain Service. Ir Med J. 2021 Aug 19;114(7):403. PMID: 34520346.

203. Wen HC, Chang WP, Hsu MH, Ho CH, Chu CM. An Assessment of the Interoperability of Electronic Health Record Exchanges Among Hospitals and Clinics in Taiwan. JMIR Med Inform. 2019 Mar 28;7(1):e12630. PMID: 30920376. doi: 10.2196/12630.

204. Nakamura MM, Harper MB, Castro AV, Yu FB, Jr., Jha AK. Impact of the meaningful use incentive program on electronic health record adoption by US children's hospitals. J Am Med Inform Assoc. 2015 Mar;22(2):390-8. PMID: 25755126. doi: 10.1093/jamia/ocu045.

205. Bagayoko CO, Muller H, Geissbuhler A. Assessment of Internet-based tele-medicine in Africa (the RAFT project). Computerized Medical Imaging and Graphics. 2006 Sep-Oct;30(6-7):407-16. PMID: WOS:000242759900011. doi: 10.1016/j.compmedimag.2006.09.014.

206. Palappallil DS, Pinheiro C. Perceptions of Prescribers towards Electronic Prescription: A Pre-Implementation Evaluation. J Young Pharm. 2018 Jul-Sep;10(3):313-7. PMID: WOS:000438923800014. doi: 10.5530/jyp.2018.10.69.

207. Mejia S, Cardona O, Ospina JG, Gutierrez J, Castrillon J, Giraldo D, et al. Antioquia's telemedicine network: Informatic and telecomunication technologies to service of health. Proceedings of the 25th Annual International Conference of the Ieee Engineering in Medicine and Biology Society, Vols 1-4: A New Beginning for Human Health. New York: Ieee; 2003. p. 3649-52.

208. Blatt AJ. Geospatial Applications in Disease Surveillance: Solutions for the Future. International Journal of Applied Geospatial Research. 2013 Apr-Jun;4(2):1-8. PMID: WOS:000219203300001. doi: 10.4018/jagr.2013040101.

209. Pham QV, Nguyen DC, Thien HT, Hwang WJ, Pathirana PN. Artificial Intelligence (AI) and Big Data for Coronavirus (COVID-19) Pandemic: A Survey on the State-of-the-Arts. Ieee Access. 2020;8:130820-39. PMID: WOS:000552987800001. doi: 10.1109/access.2020.3009328.

210. Marrie RA, Leung S, Tyry T, Cutter GR, Fox R, Salter A. Use of eHealth and mHealth technology by persons with multiple sclerosis. Mult Scler Relat Disord. 2019 Jan;27:13-9. PMID: WOS:000455500500005. doi: 10.1016/j.msard.2018.09.036.

211. Kuehne M, Blinn N, Rosenkranz C, Nuettgens M. Web 2.0 in Healthcare: State-of-the-Art in the German Health Insurance Landscape. In: Moen A, Andersen SK, Aarts J, Hurlen P, editors. User Centred Networked Health Care. Amsterdam: Ios Press; 2011. p. 649-53.

212. Krupinski EA. Innovations and Possibilities in Connected Health. J Am Acad Audiol. 2015 Oct;26(9):761-7. PMID: WOS:000361861500003. doi: 10.3766/jaaa.14047.

213. Margolis A, Bessonart L, Barbiel A, Pazos P, Gil J, Machado H, et al. A countrywide clinical informatics project in Uruguay. In: Safran C, Reti S, Marin HF, editors. Medinfo 2010, Pts I and Ii. Amsterdam: Ios Press; 2010. p. 391-5.

214. Dzissah DA, Lee JS, Suzuki H, Nakamura M, Obi T. Privacy Enhanced Healthcare Information Sharing System for Home-Based Care Environments. Healthc Inform Res. 2019 Apr;25(2):106-14. PMID: WOS:000467845500007. doi: 10.4258/hir.2019.25.2.106.

215. Jiang XH, Xie H, Tang R, Du YM, Li T, Gao JS, et al. Characteristics of Online Health Care Services From China's Largest Online Medical Platform: Cross-sectional Survey Study. J Med Internet Res. 2021 Apr;23(4):14. PMID: WOS:000642262100007. doi: 10.2196/25817.

216. Palma FNS, editor. Interoperability Challenges and Critical Success Factors in the Deployment of Cross-border Digital Medical Prescriptions in Finland and Estonia. 2022 IEEE International Conference on Digital Health (ICDH); 2022 10-16 July 2022.

217. Rodriguez-Martinez P, Sanchez-Lorente MM, Peris-Piqueras C, Sanchis-Sanchez E, Boone ALD. Implementation Plan for Stroke Telemedicine in the Valencian Region. New York: Ieee; 2016. ISBN: 978-1-5090-2486-5.

218. Batsis JA, Pletcher SN, Stahl JE. Telemedicine and primary care obesity management in rural areas - innovative approach for older adults? BMC Geriatr. 2017 Jan;17:9. PMID: WOS:000392021300003. doi: 10.1186/s12877-016-0396-x.

219. Badalato GM, Kaag M, Lee R, Vora A, Burnett A, Workgrp AUAT. Role of Telemedicine in Urology: Contemporary Practice Patterns and Future Directions. Urol Pract. 2020 Mar;7(2):122-6. PMID: WOS:000525456700010. doi: 10.1097/upj.0000000000000094.

220. Garritano FG, Goldenberg D. Successful telemedicine programs in otolaryngology. Otolaryngol Clin North Am. 2011 Dec;44(6):1259-74, vii. PMID: 22032480. doi: 10.1016/j.otc.2011.08.003.

221. Alam MZ, Hoque MR, Hu W, Barua Z. Factors influencing the adoption of mHealth services in a developing country: A patient-centric study. International Journal of Information Management. 2020 Feb;50:128-43. PMID: WOS:000497989600009. doi: 10.1016/j.ijinfomgt.2019.04.016.

222. Bali RK, Naguib RNG. Towards gestalt telehealth: Considering social, ethical and cultural issues. Dunne S, editor. New York: Ieee; 2001. 1367-71 p. ISBN: 0-7803-6715-4.

223. Geier AS. [Digital health applications (DiGA) on the road to success-the perspective of the German Digital Healthcare Association]. Bundesgesundheitsblatt Gesundheitsforschung Gesundheitsschutz. 2021 Oct;64(10):1228-31. PMID: 34524478. doi: 10.1007/s00103-021-03419-5.

224. Yao HW, Suo JJ, Xing YB, Du MM, Bai YL, Liu BW, et al. The Minimum Data Set and Quality Indicators for National Healthcare-Associated Infection Surveillance in Mainland China: Towards Precision Management. Biomed Res Int. 2019 Jul;2019:7. PMID: WOS:000476729000001. doi: 10.1155/2019/2936264.

225. Hwabamungu B, Williams Q. m-Health adoption and sustainability prognosis from a care givers' and patients' perspective. Proceedings of the 2010 Annual Research Conference of the South African Institute of Computer Scientists and Information Technologists; Bela Bela, South Africa: Association for Computing Machinery; 2010. p. 123–31.

226. Mukherjee A, Daniel M, Kaur A, Devarapalli S, Kallakuri S, Essue B, et al. Operational challenges in the pre-intervention phase of a mental health trial in rural India: reflections from SMART Mental Health. Int J Ment Health Syst. 2022 Aug;16(1):15. PMID: WOS:000841232700001. doi: 10.1186/s13033-022-00549-4.

227. Pfeiffer KP. [Actual state and perspectives of e-health in Austria and international--an overview]. Wien Med Wochenschr. 2011 Jul;161(13-14):334-40. PMID: 21858631. doi: 10.1007/s10354-011-0008-5.

228. Peabody MR, Dai ML, Turner K, Peterson LE, Mainous AG. Prevalence and Factors Associated with Family Physicians Providing E-Visits. J Am Board Fam Med. 2019 Nov-Dec;32(6):868-75. PMID: WOS:000496237600015. doi: 10.3122/jabfm.2019.06.190081.

229. Moo S, Fletcher J. Northern Territory HealthConnect: Shared Electronic Health Record Service Implementation Experiences and Benefits Realised in Indigenous Health. In: Kuhn KA, Warren JR, Leong TY, editors. Medinfo 2007: Proceedings of the 12th World Congress on Health. Amsterdam: Ios Press; 2007. p. 297-+.

230. Rinaldi S, Gandhi A, Selviandro N, Ieee, editors. Usability Evaluation and Recommendation of User Interface Design for e-HAC Application by Using User-Centered Design Method. 24th International Conference on Advanced Communication Technology (ICACT) - Artificial Intelligence Technologies toward Cybersecurity; 2022 Feb 13-16; Electr Network. NEW YORK: Ieee; 2022.

231. Carlo L, Carpio V, Verdezoto N, Eslambolchilar P, Cruz E, Malo F, et al. Healthcare Infrastructures in Ecuador: Challenges, Reflections and Opportunities for Digital Health. Proceedings of the 2020 International Conference on Information and Communication Technologies and Development; Guayaquil, Ecuador: Association for Computing Machinery; 2020. p. Article 25.

232. Julien SP. Electronic Health Records. In: Magnuson JA, Fu PC, editors. Public Health Informatics and Information Systems, 2nd Edition. New York: Springer; 2014. p. 173-89.

233. Ambrosino N, Vagheggini G, Mazzoleni S, Vitacca M. Telemedicine in chronic obstructive pulmonary disease. Breathe (Sheff). 2016 Dec;12(4):350-6. PMID: 28210321. doi: 10.1183/20734735.014616.

234. Van Eaton EG, Devlin AB, Devine EB, Flum DR, Tarczy-Hornoch P. Achieving and sustaining automated health data linkages for learning systems: barriers and solutions. EGEMS (Wash DC). 2014;2(2):1069. PMID: 25848606. doi: 10.13063/2327-9214.1069.

235. Bernstein K, Tvede I, Petersen J, Bredegaard K. Can openEHR Archetypes Be Used in a National Context? The Danish Archetype Proof-of-Concept Project. In: Adlassnig KP, Blobel B, Mantas J, Masic I, editors. Medical Informatics in a United and Healthy Europe. Amsterdam: Ios Press; 2009. p. 147-51.

236. Ramos AC, Buceta BB, Da Silva AF, Lorenzo RB. eHealth in Spain: evolution, current status and future prospects. Saude E Sociedade. 2020;29(4):12. PMID: WOS:000610972300001. doi: 10.1590/s0104-12902020190886.

237. Dooley AB, de la Houssaye N, Baum N. Use of Telemedicine for Sexual Medicine Patients. Sexual Medicine Reviews. 2020 Oct;8(4):507-17. PMID: WOS:000576792100002. doi: 10.1016/j.sxmr.2020.06.001.

238. Otte-Trojel T, de Bont A, van de Klundert J, Rundall TG. Characteristics of patient portals developed in the context of health information exchanges: early policy effects of incentives in the meaningful use program in the United States. J Med Internet Res. 2014 Nov 21;16(11):e258. PMID: 25447837. doi: 10.2196/jmir.3698.

239. Fisher B, Fitton R, Poirier C, Stables D. Patient Record Access - The Time Has Come. In: Bos L, Roa L, Yogesan K, Oconnell B, Marsh A, Blobel B, editors. Medical and Care Compunetics 3. Amsterdam: Ios Press; 2006. p. 162-+.

240. Tsoulkas VN, Pantelous AA. The embedding of system's and control's terminology and conceptualization to model-based Tele-medicine-Assisted Home Support (TAHoS). Uksim 2009: Eleventh International Conference on Computer Modelling and Simulation. Los Alamitos: Ieee Computer Soc; 2009. p. 538-+.

241. Gjestsen MT, Wiig S, Testad I. What are the key contextual factors when preparing for successful implementation of assistive living technology in primary elderly care? A case study from Norway. Bmj Open. 2017 Sep;7(9):9. PMID: WOS:000412650700051. doi: 10.1136/bmjopen-2016-015455.

242. Nakajima I. Worldwide Trends in Universal Service Funds and Telemedicine. J Med Syst. 2010 Dec;34(6):1105-10. PMID: WOS:000283258000013. doi: 10.1007/s10916-009-9329-y.

243. Wahab RA, Kusumawardani QD, Wijaya FP, editors. The Potential Implementation of Telemedicine in Frontier, Outmost, and Underdeveloped Region of Indonesia. 2021 2nd International Conference on ICT for Rural Development (IC-ICTRuDev); 2021 27-28 Oct. 2021.

244. Alpay LL, Henkemans OB, Otten W, Rövekamp TA, Dumay AC. E-health applications and services for patient empowerment: directions for best practices in The Netherlands. Telemed J E Health. 2010 Sep;16(7):787-91. PMID: 20815745. doi: 10.1089/tmj.2009.0156.

245. Wamala D, Katamba A, Dworak O. Feasibility and diagnostic accuracy of Internet-based dynamic telepathology between Uganda and Germany. J Telemed Telecare. 2011;17(5):222-5. PMID: 21565844. doi: 10.1258/jtt.2010.100609.

246. Dillon E, Loermans J. Telehealth in Western Australia: the challenge of evaluation. J Telemed Telecare. 2003;9:S15-S9. PMID: WOS:000187627900007.

247. Zanaboni P, Knarvik U, Wootton R. Adoption of routine telemedicine in Norway: the current picture. Glob Health Action. 2014;7:22801. PMID: 24433942. doi: 10.3402/gha.v7.22801.

248. Cortez NG, Cohen IG, Kesselheim AS. FDA regulation of mobile health technologies. N Engl J Med. 2014 Jul 24;371(4):372-9. PMID: 25054722. doi: 10.1056/NEJMhle1403384.

249. Williams D, Lawrence J, Hong YR, Winn A. Tele-ICUs for COVID-19: A Look at National Prevalence and Characteristics of Hospitals Providing Teleintensive Care. J Rural Health. 2021 Jan;37(1):133-41. PMID: WOS:000575992700001. doi: 10.1111/jrh.12524.

250. Medford-Davis LN, Chang L, Rhodes KV. Health Information Exchange: What do patients want? Health Inform J. 2017 Dec;23(4):268-78. PMID: WOS:000415227500003. doi: 10.1177/1460458216647190.

251. Leao BF, Costa CG, Facchini LA, Bandarra EB, Goncalves SF, Junior NB, et al. The Brazilian Health Informatics and Information Policy: building the consensus. In: Fieschi M, Coiera E, Li YCJ, editors. Medinfo 2004: Proceedings of the 11th World Congress on Medical Informatics, Pt 1 and 2. Amsterdam: I O S Press; 2004. p. 1207-10.

252. Carnicero R, Rojas D, Elicegui I, Carnicero J. Proposal of a Learning Health System to Transform the National Health System of Spain. Processes. 2019 Sep;7(9):23. PMID: WOS:000489121800066. doi: 10.3390/pr7090613.

253. Frøen JF, Myhre SL, Frost MJ, Chou D, Mehl G, Say L, et al. eRegistries: Electronic registries for maternal and child health. BMC Pregnancy Childbirth. 2016 Jan 19;16:11. PMID: 26791790. doi: 10.1186/s12884-016-0801-7.

254. Mendelson DN, Salinsky EM. Health information systems and the role of state government. Health Affairs. 1997 May-Jun;16(3):106-19. PMID: WOS:A1997WW87600013. doi: 10.1377/hlthaff.16.3.106.

255. Cullen R. The Use of ICT in the Health Sector in Pacific Island Countries. Cullen R, Hassall G, editors. Cham: Springer International Publishing Ag; 2017. 305-35 p. ISBN: 978-3-319-50972-3; 978-3-319-50970-9.

256. Breitinger S, Gentry MT, Hilty DM. Key Opportunities for the COVID-19 Response to Create a Path to Sustainable Telemedicine Services. Mayo Clin Proc. 2020 Dec;95(12):2602-5. PMID: 33276833. doi: 10.1016/j.mayocp.2020.09.034.

257. Belghiti J, Oget-Gendre C, Berthon AF, Fagon JY. Supporting innovation in health care: A short experience in a dedicated unit of the French Ministry of Public Health. J Visc Surg. 2021 Jun;158(3s):S6-s11. PMID: 33716003. doi: 10.1016/j.jviscsurg.2021.01.009.

258. Saleem Y, Taylor MH, Khalifa N. Forensic telepsychiatry in the United Kingdom. Behav Sci Law. 2008;26(3):333-44. PMID: 18548516. doi: 10.1002/bsl.810.

259. Zachrison KS, Boggs KM, Hayden EM, Cash RE, Espinola JA, Samuels-Kalow ME, et al. Factors associated with emergency department adoption of telemedicine: 2014 to 2018. J Am Coll Emerg Physicians Open. 2020 Dec;1(6):1304-11. PMID: 33392537. doi: 10.1002/emp2.12233.

260. Wood CS, Thomas MR, Budd J, Mashamba-Thompson TP, Herbst K, Pillay D, et al. Taking connected mobile-health diagnostics of infectious diseases to the field. Nature. 2019 Feb;566(7745):467-74. PMID: 30814711. doi: 10.1038/s41586-019-0956-2.

261. Htat KK, Williams PAH, McCauley V. Security of ePrescriptions: data in transit comparison using existing and mobile device services. Proceedings of the Australasian Computer Science Week Multiconference; Geelong, Australia: Association for Computing Machinery; 2017. p. Article 56.

262. Flynn D, Gregory P, Makki H, Gabbay M. Expectations and experiences of eHealth in primary care: a qualitative practice-based investigation. Int J Med Inform. 2009 Sep;78(9):588-604. PMID: 19482542. doi: 10.1016/j.ijmedinf.2009.03.008.

263. Taylor L, Capling H, Portnoy JM. Administering a Telemedicine Program. Curr Allergy Asthma Rep. 2018 Nov;18(11):7. PMID: WOS:000444612500001. doi: 10.1007/s11882-018-0812-8.

264. Brennan DM, Holtz BE, Chumbler NR, Kobb R, Rabinowitz T. Visioning technology for the future of telehealth. Telemed J E Health. 2008 Nov;14(9):982-5. PMID: 19035812. doi: 10.1089/tmj.2008.0116.

265. Krasuska M, Williams R, Sheikh A, Franklin B, Hinder S, TheNguyen H, et al. Driving digital health transformation in hospitals: a formative qualitative evaluation of the English Global Digital Exemplar programme. BMJ Health Care Inform. 2021 Dec;28(1). PMID: 34921060. doi: 10.1136/bmjhci-2021-100429.

266. Hogan K, Macedo B, Macha V, Barman A, Jiang XQ. Contact Tracing Apps: Lessons Learned on Privacy, Autonomy, and the Need for Detailed and Thoughtful Implementation. JMIR Med Inf. 2021 Jul;9(7):20. PMID: WOS:000679941500019. doi: 10.2196/27449.

267. De Rosis S, Nuti S. Public strategies for improving eHealth integration and long-term sustainability in public health care systems: Findings from an Italian case study. Int J Health Plann Manage. 2018 Jan;33(1):e131-e52. PMID: 28791771. doi: 10.1002/hpm.2443.

268. Wells S, Rozenblum R, Park A, Dunn M, Bates DW. Personal health records for patients with chronic disease: a major opportunity. Appl Clin Inform. 2014;5(2):416-29. PMID: 25024758. doi: 10.4338/aci-2014-01-ra-0002.

269. Rozenblum R, Jang Y, Zimlichman E, Salzberg C, Tamblyn M, Buckeridge D, et al. A qualitative study of Canada's experience with the implementation of electronic health information technology. Can Med Assoc J. 2011 Mar;183(5):E281-E8. PMID: WOS:000288953900014. doi: 10.1503/cmaj.100856.

270. Perrone G, Zerbo S, Bilotta C, Malta G, Argo A. Telemedicine during Covid-19 pandemic: Advantage or critical issue? Med Leg J. 2020 Jul;88(2):76-7. PMID: 32490720. doi: 10.1177/0025817220926926.

271. Al Hemairy M, Amin S, Hijji M, Serhani M, Al Ahmad M. Integrated and Scalable Architecture for Providing Cost-Effective Remote Health Monitoring. In: Aljumeily D, Hussain A, Tawfik H, Hamdan H, Dawson T, Hind J, editors. 2016 9th International Conference on Developments in Esystems Engineering. New York: Ieee; 2016. p. 74-80.

272. Sao D, Gupta A, Gantz DA. Interoperable Electronic Health Care Record: A Case for Adoption of a National Standard to Stem the Ongoing Health Care Crisis. Journal of Legal Medicine. 2013 Jan;34(1):55-90. PMID: WOS:000317267200004. doi: 10.1080/01947648.2013.768153.

273. Strumann C, Möller B, Steinhäuser J. [Assessing Electronic Prescription: A Cross-sectional Study of Pharmacists in Germany]. Gesundheitswesen. 2022 Oct;84(10):961-7. PMID: 34161985. doi: 10.1055/a-1498-1816.

274. Devlin AM, McGee-Lennon M, O'Donnell CA, Bouamrane MM, Agbakoba R, O'Connor S, et al. Delivering digital health and well-being at scale: lessons learned during the implementation of the dallas program in the United Kingdom. Journal of the American Medical Informatics Association. 2016 Jan;23(1):48-59. PMID: WOS:000374179500008. doi: 10.1093/jamia/ocv097.

275. Jorgensen D, Hallenborg K, Demazeau Y. Assessment of Agent Architectures for Telehealth. In: Corchado JM, Bajo J, Kozlak J, Pawlewski P, Molina JM, Gaudou B, et al., editors. Highlights of Practical Applications of Heterogeneous Multi-Agent Systems: The Paams Collection. Berlin: Springer-Verlag Berlin; 2014. p. 79-88.

276. Voss H, Heimly V, Sjogren LH. The Baltic Health Network - Taking Secure, Internet-based Healthcare Networks to the Next Level. In: Engelbrecht R, Geissbuhler A, Lovis C, Mihalas G, editors. Connecting Medical Informatics and Bio-Informatics; Amsterdam: Ios Press; 2005. p. 421-6.

277. Brauns HJ, Loos W. [Telemedicine in Germany. Status, Barriers, Perspectives]. Bundesgesundheitsblatt Gesundheitsforschung Gesundheitsschutz. 2015 Oct;58(10):1068-73. PMID: 26324096. doi: 10.1007/s00103-015-2223-5.

278. Scandurra I, Hagglund M, Persson A, Ahlfeldt RM. Disturbing or Facilitating? - On the Usability of Swedish eHealth Systems 2013. In: Lovis C, Seroussi B, Hasman A, PapeHaugaard L, Saka O, Andersen SK, editors. E-Health - for Continuity of Care. Amsterdam: Ios Press; 2014. p. 221-5.

279. Lewis CE. My computer, my doctor: a constitutional call for federal regulation of cybermedicine. Am J Law Med. 2006;32(4):585-609. PMID: 17240732. doi: 10.1177/009885880603200403.

280. Hinman AR, Ross DA. Immunization Registries Can Be Building Blocks For National Health Information Systems. Health Affairs. 2010 Apr;29(4):676-82. PMID: WOS:000276360200016. doi: 10.1377/hlthaff.2007.0594.

281. Stroetmann KA. Analysis and Typology of Global eHealth Platforms - A Survey on Five Continents. In: Gillis G, Newsham D, Maeder AJ, editors. Global Telehealth 2015: Integrating Technology and Information for Better Healthcare. Amsterdam: Ios Press; 2015. p. 162-9.

282. Reams C, Powell M, Edwards R. State synergies and disease surveillance: creating an electronic health data communication model for cancer reporting and comparative effectiveness research in kentucky. EGEMS (Wash DC). 2014;2(2):1064. PMID: 25848604. doi: 10.13063/2327-9214.1064.

283. Abbasi-Feinberg F. Telemedicine Coding and Reimbursement - Current and Future Trends. Sleep Med Clin. 2020 Sep;15(3):417-29. PMID: WOS:000555699600009. doi: 10.1016/j.jsmc.2020.06.002.

284. Pettersen S, Uldal SB, Baardsgard A, Amundsen M, Myrvang R, Nordvag D, et al. The North Norwegian Health Net. J Telemed Telecare. 1999;5:34-6. PMID: WOS:000079101100014. doi: 10.1258/1357633991932469.

285. Bashshur RL, Doarn CR, Frenk JM, Kvedar JC, Shannon GW, Woolliscroft JO. Beyond the COVID Pandemic, Telemedicine, and Health Care. Telemed J E Health. 2020 Nov;26(11):1310-3. PMID: 32809913. doi: 10.1089/tmj.2020.0328.

286. Kijsanayotin B, Kasitipradith N, Pannarunothai S. eHealth in Thailand: the current status. Stud Health Technol Inform. 2010;160(Pt 1):376-80. PMID: 20841712.

287. Multak N. Primary Care Patient Management and Health Information Technology. Sarnikar S, Bennett D, Gaynor M, editors. Hersey: Igi Global; 2013. 113-21 p. ISBN: 978-1-4666-2702-4; 978-1-4666-2671-3.

288. Coppolino L, D'Antonio S, Romano L, Staffa M. KONFIDO Project: a secure infrastructure increasing interoperability on a systemic level among eHealth services across Europe. Wu Y, Min G, Georgalas N, AlDubi A, Jin X, Yang L, et al., editors. New York: Ieee; 2017. 342-7 p. ISBN: 978-1-5386-3066-2.

289. Were MC, Emenyonu N, Achieng M, Shen CY, Ssali J, Masaba JPM, et al. Evaluating a scalable model for implementing electronic health records in resource-limited settings. Journal of the American Medical Informatics Association. 2010 May;17(3):237-44. PMID: WOS:000277580700002. doi: 10.1136/jamia.2009.002303.

290. Alsharif A. Applying eHealth for Pandemic Management in Saudi Arabia in the Context of COVID-19: Survey Study and Framework Proposal. JMIR Med Inf. 2020 Nov;8(11):13. PMID: WOS:000595663400031. doi: 10.2196/19524.

291. Kaur A, Gupta AK, editors. E-Health Approaches for Developing Countries. 2019 5th International Conference on Signal Processing, Computing and Control (ISPCC); 2019 10-12 Oct. 2019.

292. Bhatta R, Aryal K, Ellingsen G. Opportunities and Challenges of a Rural-telemedicine Program in Nepal. J Nepal Health Res Counc. 2015 May-Aug;13(30):149-53. PMID: 26744201.

293. Calman N, Hauser D, Lurio J, Wu WY, Pichardo M. Strengthening public health and primary care collaboration through electronic health records. Am J Public Health. 2012 Nov;102(11):e13-8. PMID: 22994274. doi: 10.2105/ajph.2012.301000.

294. Tahan V, Almashhrawi A, Mutrux R, Ibdah JA. Show Me ECHO-Hepatitis C: A telemedicine mentoring program for patients with hepatitis C in underserved and rural areas in Missouri as a model in developing countries. Turk J Gastroenterol. 2015 Nov;26(6):447-9. PMID: 26510085. doi: 10.5152/tjg.2015.159000.

295. Da Silveira M, Guelfi N, Baldacchino JD, Plumer P, Seil M, Wienecke A. A survey of interoperability in e-health systems - The European approach. Azevedo L, Londral AR, editors. Setubal: Insticc-Inst Syst Technologies Information Control & Communication; 2008. 172-+ p. ISBN: 978-989-8111-16-6.

296. Al Jarullah A, El-Masri S. Proposal of an architecture for the national integration of Electronic Health Records: a semi-centralized approach. Stud Health Technol Inform. 2012;180:917-21. PMID: 22874326.

297. El-Mahalli AA, El-Khafif SH, Al-Qahtani MF. Successes and challenges in the implementation and application of telemedicine in the eastern province of Saudi Arabia. Perspect Health Inf Manag. 2012;9(Fall):1-27. PMID: 23209455.

298. Dogac A, Yuksel M, Ertürkmen GL, Kabak Y, Namli T, Yıldız MH, et al. Healthcare information technology infrastructures in Turkey. Yearb Med Inform. 2014 May 22;9(1):228-34. PMID: 24853036. doi: 10.15265/iy-2014-0001.

299. Zulriqar E, Junejo A. Suparco telemedicine pilot project. New York: Ieee; 2007. 655-9 p. ISBN: 978-1-4244-1056-9.

300. Fong A, Adams K, Samarth A, McQueen L, Trivedi M, Chappel T, et al. Assessment of Automating Safety Surveillance From Electronic Health Records: Analysis for the Quality and Safety Review System. J Patient Saf. 2021 Sep 1;17(6):e524-e8. PMID: 28671914. doi: 10.1097/pts.0000000000000402.

301. Dolezel D, Moczygemba J. Implementing EHRs: An Exploratory Study to Examine Current Practices in Migrating Physician Practice. Perspect Health Inf Manag. 2015;12(Winter):1e. PMID: 26807077.

302. Jaroslawski S, Saberwal G. In eHealth in India today, the nature of work, the challenges and the finances: an interview-based study. Bmc Medical Informatics and Decision Making. 2014 Jan;14:12. PMID: WOS:000331820700001. doi: 10.1186/1472-6947-14-1.

303. Ghane K. Healthcare Information Exchange System based on a Hybrid Central/Federated Model. 2014 36th Annual International Conference of the Ieee Engineering in Medicine and Biology Society. New York: Ieee; 2014. p. 1362-5.

304. Keesara S, Jonas A, Schulman K. Covid-19 and Health Care's Digital Revolution. N Engl J Med. 2020 Jun 4;382(23):e82. PMID: 32240581. doi: 10.1056/NEJMp2005835.

305. Gerber A, Topaz M. Promoting Meaningful Use of Health Information Technology in Israel: Ministry of Health Vision. In: Saranto K, Weaver CA, Chang P, editors. Nursing Informatics 2014: East Meets West Esmart+. Amsterdam: Ios Press; 2014. p. 108-15.

306. Mougiakakou SG, Kyriacou E, Perakis K, Papadopoulos H, Androulidakis A, Konnis G, et al. A feasibility study for the provision of electronic healthcare tools and services in areas of Greece, Cyprus and Italy. Biomed Eng Online. 2011 Jun;10:17. PMID: WOS:000292239800001. doi: 10.1186/1475-925x-10-49.

307. Dittrich F, Albrecht UV, von Jan U, Malinka C, Ansorg J, Jung J, et al. The Digital Healthcare Act - a Turning Point in the German Digitisation Strategy? Z Orthop Unfall. 2020 May 4. PMID: 32365397. doi: 10.1055/a-1141-4274.

308. Jahn K, Gartig-Daugs A, Nagel E. Electronic health records within integrated care in Germany. Telemed J E Health. 2005 Apr;11(2):146-50. PMID: WOS:000228957900021. doi: 10.1089/tmj.2005.11.146.

309. Fan J, Lin HY, Qin MW. [Practice and System Construction of Telemedicine for Coronavirus Disease 2019 Epidemic Prevention and Control]. Zhongguo Yi Xue Ke Xue Yuan Xue Bao. 2020 Aug 30;42(4):531-4. PMID: 32895106. doi: 10.3881/j.issn.1000-503X.12890.

310. Metzger MH, Durand T, Lallich S, Salamon R, Castets P. The use of regional platforms for managing electronic health records for the production of regional public health indicators in France. Bmc Medical Informatics and Decision Making. 2012 Apr;12:14. PMID: WOS:000305411300001. doi: 10.1186/1472-6947-12-28.

311. Xu XY, Cai YY, Wu SY, Guo JH, Yang L, Lan JL, et al. Assessment of Internet Hospitals in China During the COVID-19 Pandemic: National Cross-Sectional Data Analysis Study. J Med Internet Res. 2021 Jan;23(1):12. PMID: WOS:000609010900002. doi: 10.2196/21825.

312. Rodrigues J, Pedro L, Vardasca T, de la Torre-Diez I, Martins HMG. Mobile health platform for pressure ulcer monitoring with electronic health record integration. Health Inform J. 2013 Dec;19(4):300-11. PMID: WOS:000329918000004. doi: 10.1177/1460458212474909.

313. Nakamura MM, Ferris TG, DesRoches CM, Jha AK. Electronic health record adoption by children's hospitals in the United States. Arch Pediatr Adolesc Med. 2010 Dec;164(12):1145-51. PMID: 21135344. doi: 10.1001/archpediatrics.2010.234.

314. Yun EK, Park HA. Strategy development for the implementation of telenursing in Korea. Comput Inform Nurs. 2007 Sep-Oct;25(5):301-6. PMID: 17827994. doi: 10.1097/01.NCN.0000289167.38992.4b.

315. Khan MAH, Cruz VO, Azad AK. Bangladesh's digital health journey: reflections on a decade of quiet revolution. WHO South East Asia J Public Health. 2019 Sep;8(2):71-6. PMID: 31441440. doi: 10.4103/2224-3151.264849.

316. Heimann P, Lorenz N, Blum N, Schifferings C. [Experiences of digital health care applications (DIGA) manufacturers with the BfArM Fast-Track procedure]. Bundesgesundheitsblatt Gesundheitsforschung Gesundheitsschutz. 2021 Oct;64(10):1249-53. PMID: 34542649. doi: 10.1007/s00103-021-03422-w.

317. Neves J, Santos M, Machado J, Abelha A. Electronic Health Records - Organizational, Regional, National, or Worldwide? In: Long CA, Anninos P, Pham T, Anastassopoulos G, Mastorakis NE, editors. Proceedings of the 1st Wseas International Conference on Biomedical Electronics and Biomedical Informatics. Athens: World Scientific and Engineering Acad and Soc; 2008. p. 116-+.

318. Ding CY. Patient Privacy Protection in China in the Age of Electronic Health Records. Hong Kong Law Journal. 2013;43:245-78. PMID: WOS:000322091100010.

319. Hohman JA, Martinez KA, Anand A, Rood M, Martyn T, Rose S, et al. Use of Direct-to-Consumer Telemedicine to Access Mental Health Services. J Gen Intern Med. 2022 Aug;37(11):2759-67. PMID: WOS:000748306500001. doi: 10.1007/s11606-021-07326-y.

320. Keugoung B, Bello KOA, Millimouno TM, Sidibé S, Dossou JP, Delamou A, et al. Mobilizing health district management teams through digital tools: Lessons from the District.Team initiative in Benin and Guinea using an action research methodology. Learn Health Syst. 2021 Oct;5(4):e10244. PMID: 34667871. doi: 10.1002/lrh2.10244.

321. Schwarz U. [Health education through digital audio-visual media: strategies of the German Federal Centre for Health Education (BZgA)]. Bundesgesundheitsblatt Gesundheitsforschung Gesundheitsschutz. 2020 Jun;63(6):715-20. PMID: 32430510. doi: 10.1007/s00103-020-03145-4.

322. Karamagkioli KZ, Karamagioli E, Smedberg A. European E-Health Framework: Towards More "Patient-Friendly" Healthcare Services? Hersey: Igi Global; 2012. 262-78 p. ISBN: 978-1-60960-866-8.

323. Weiser SJ. Breaking down the federal and state barriers preventing the implementation of accurate, reliable and cost effective electronic health records. Ann Health Law. 2010;19(1 Spec No):205-11. PMID: 21495572.

324. Juhra C, Hernandez M, Ho K, Kushniruk A, Borycki E. The health policy guidance and practice of introducing technologies in health system in Europe. New York: Ieee; 2016. 35-6 p. ISBN: 978-1-5090-1000-4.

325. Visser JJW, Bloo JKC, Grobbe FA, Vollenbroek-Hutten MMR. Video Teleconsultation Service: Who Is Needed to Do What, to Get It Implemented in Daily Care? Telemed J E Health. 2010 May;16(4):439-45. PMID: WOS:000278107000044. doi: 10.1089/tmj.2009.0101.

326. Lauer W, Lobker W, Hofgen B. Digital health applications (DiGA): assessment of reimbursability by means of the "DiGA Fast Track" procedure at the Federal Institute for Drugs and Medical Devices (BfArM). Bundesgesundheitsblatt-Gesund. 2021 Oct;64(10):1232-40. PMID: WOS:000696425300004. doi: 10.1007/s00103-021-03409-7.

327. Mertz L. Electronic health records usher in a new era in health care: making the transition to health information technology. IEEE Pulse. 2012 Nov-Dec;3(6):43-50. PMID: 23247158. doi: 10.1109/mpul.2012.2216718.

328. Saeed SA. Telebehavioral health: clinical applications, benefits, technology needs, and setup. N C Med J. 2015 Jan-Feb;76(1):25-6. PMID: 25621475.

329. Andreassen OA. eHealth provides a novel opportunity to exploit the advantages of the Nordic countries in psychiatric genetic research, building on the public health care system, biobanks, and registries. American Journal of Medical Genetics Part B-Neuropsychiatric Genetics. 2018 Oct;177(7):625-9. PMID: WOS:000449631600004. doi: 10.1002/ajmg.b.32561.

330. Ellimoottil C, An L, Moyer M, Sossong S, Hollander JE. Challenges And Opportunities Faced By Large Health Systems Implementing Telehealth. Health Aff (Millwood). 2018 Dec;37(12):1955-9. PMID: 30633667. doi: 10.1377/hlthaff.2018.05099.

331. Brighi R, Virone MG. EHR and usability of health data to benefit patient and public health. Stud Health Technol Inform. 2014;205:965-9. PMID: 25160331.

332. Sjogren LH, Schwieler A, Karlsson L. Telemedicine in Sweden: National co-operation, development & evaluation. In: Patel VL, Rogers R, Haux R, editors. Medinfo 2001: Proceedings of the 10th World Congress on Medical Informatics, Pts 1 and 2. Amsterdam: I O S Press; 2001. p. 879-.

333. Pande A, Kimbahune S, Bondale N, Shinde R, Shanbhag S. Distributed Processing and Internet Technology to Solve Challenges of Primary Healthcare in India. In: Ramanujam R, editor. Distributed Computing and Internet Technology. Berlin: Springer-Verlag Berlin; 2012. p. 188-99.

334. Park HY, Kim JY, Koo HY, Han J, Jun JH, Lee W, et al. Evaluation of a Telehealth Counseling Program for Expatriates. Telemed e-Health. 2019 Aug;25(8):693-700. PMID: WOS:000444045000001. doi: 10.1089/tmj.2018.0082.

335. Paul S, Das Bhattacharya S, Sudar A, Patra D, Majumdar AK, Mukhopadhyay J, et al. A Web-Based Electronic Health Care System for the Treatment of Pediatric HIV. New York: Ieee; 2009. 175-+ p. ISBN: 978-1-4244-5013-8.

Publications without sufficient description of the intervention

1. Aanestad M, Grisot M, Hanseth O, Vassilakopoulou P. Information Infrastructures within European Health Care: Working with the Installed Base. Aanestad M, Grisot M, Hanseth O, Vassilakopoulou P, editors. Cham (CH): Springer; 2017.

2. Abbott-Garner P, Richardson J, Jones RB. The Impact of Superfast Broadband, Tailored Booklets for Households, and Discussions With General Practitioners on Personal Electronic Health Readiness: Cluster Factorial Quasi-Randomized Control Trial. J Med Internet Res. 2019 Mar 11;21(3):e11386. PMID: 30855234. doi: 10.2196/11386.

3. Abd Ghani MK, Bali RK, Naguib RN, Marshall IM. Electronic health records approaches and challenges: a comparison between Malaysia and four East Asian countries. Int J Electron Healthc. 2008;4(1):78-104. PMID: 18583297. doi: 10.1504/ijeh.2008.018922.

4. Abdolrasulnia M, Menachemi M, Shewchuk RM, Ginter PM, Duncan WJ, Brooks RG. Market effects on electronic health record adoption by physicians. Health Care Manage Rev. 2008 Jul-Sep;33(3):243-52. PMID: WOS:000257090700007. doi: 10.1097/01.HMR.0000324904.19272.c2.

5. Abid A, Cheikhrouhou S, Kallel S, Jmaiel M. NovidChain: Blockchain-based privacy-preserving platform for COVID-19 test/vaccine certificates. Softw-Pract Exp. 2022 Apr;52(4):841-67. PMID: WOS:000651548100001. doi: 10.1002/spe.2983.

6. Abidi SSR, Goh A, Yusoff Z. Telemedicine and medical informatics in the Multimedia Super Corridor: The Malaysian vision. In: Cesnik B, McCray AT, Scherrer JR, editors. Medinfo '98 - 9th World Congress on Medical Informatics, Pts 1 and 2. Amsterdam: I O S Press; 1998. p. 1282-6.

7. Abidi SSR, Yusoff Z. Telemedicine in the Malaysian Multimedia Super Corridor: Towards Personalised Lifetime Health Plans. In: Kokol P, Zupan B, Stare J, Premik M, Engelbrecht R, editors. Medical Informatics Europe '99. Amsterdam: I O S Press; 1999. p. 283-8.

8. Abraham S. Technological Trends in Health Care Electronic Health Record. Health Care Manager. 2010 Oct-Dec;29(4):318-23. PMID: WOS:000212531400004. doi: 10.1097/HCM.0b013e3181fa032c.

9. Abramson EL, Edwards A, Silver M, Kaushal R. Trending Health Information Technology Adoption Among New York Nursing Homes. Am J Manag Care. 2014 Nov;20:ESP53-+. PMID: WOS:000351003100008.

10. Abramson EL, Silver M, Kaushal R, Investigators H. Meaningful Use Status and Participation in Health Information Exchange Among New York State Hospitals: A Longitudinal Assessment. Jt Comm J Qual Patient Saf. 2014 Oct;40(10):452-+. PMID: WOS:000443983800003. doi: 10.1016/s1553-7250(14)40058-8.

11. Acharya A, Schroeder D, Schwei K, Chyou PH. Update on Electronic Dental Record and Clinical Computing Adoption Among Dental Practices in the United States. Clin Med Res. 2017 Dec;15(3-4):59-74. PMID: 29229631. doi: 10.3121/cmr.2017.1380.

12. Achelrod D, Schreyogg J, Stargardt T. Health-economic evaluation of home telemonitoring for COPD in Germany: evidence from a large population-based cohort. European Journal of Health Economics. 2017 Sep;18(7):869-82. PMID: WOS:000406690000006. doi: 10.1007/s10198-016-0834-x.

13. Adams JL, Myers TL, Waddell EM, Spear KL, Schneider RB. Telemedicine: a Valuable Tool in Neurodegenerative Diseases. Current Geriatrics Reports. 2020 Jun;9(2):72-81. PMID: WOS:000528382700007. doi: 10.1007/s13670-020-00311-z.

14. Adler-Milstein J, Kvedar J, Bates DW. Telehealth among US hospitals: several factors, including state reimbursement and licensure policies, influence adoption. Health Aff (Millwood). 2014 Feb;33(2):207-15. PMID: 24493762. doi: 10.1377/hlthaff.2013.1054.

15. Adler-Milstein J, Raphael K, Bonner A, Pelton L, Fulmer T. Hospital adoption of electronic health record functions to support age-friendly care: results from a national survey. J Am Med Inform Assoc. 2020 Aug 1;27(8):1206-13. PMID: 32772089. doi: 10.1093/jamia/ocaa129.

16. Adler-Milstein J, Sarma N, Woskie LR, Jha AK. A comparison of how four countries use health IT to support care for people with chronic conditions. Health Aff (Millwood). 2014 Sep;33(9):1559-66. PMID: 25201660. doi: 10.1377/hlthaff.2014.0424.

17. Ahmadi H, Nilashi M, Shahmoradi L, Ibrahim O. Hospital Information System adoption: Expert perspectives on an adoption framework for Malaysian public hospitals. Comput Human Behav. 2017 Feb;67:161-89. PMID: WOS:000390075200016. doi: 10.1016/j.chb.2016.10.023.

18. Akematsu Y, Tsuji M. Relation between telecare implementation and number of treatment days in a Japanese town. J Telemed Telecare. 2013 Jan;19(1):36-9. PMID: 23390215. doi: 10.1177/1357633x12474743.

19. Al Jarullah A, El-Masri S. A Novel System Architecture for the National Integration of Electronic Health Records: A Semi-Centralized Approach. J Med Syst. 2013 Aug;37(4):20. PMID: WOS:000323671800003. doi: 10.1007/s10916-013-9953-4.

20. Al Rajeh A, Steiner MC, Aldabayan Y, Aldhahir A, Pickett E, Quaderi S, et al. Use, utility and methods of telehealth for patients with COPD in England and Wales: a healthcare provider survey. BMJ Open Respir Res. 2019;6(1):e000345. PMID: 30956795. doi: 10.1136/bmjresp-2018-000345.

21. Alami H, Gagnon MP, Wootton R, Fortin JP, Zanaboni P. Exploring factors associated with the uneven utilization of telemedicine in Norway: a mixed methods study. BMC Med Inform Decis Mak. 2017 Dec 28;17(1):180. PMID: 29282048. doi: 10.1186/s12911-017-0576-4.

22. Alanezi F. Factors affecting the adoption of e-health system in the Kingdom of Saudi Arabia. Int Health. 2021 Sep;13(5):456-70. PMID: WOS:000696579000008. doi: 10.1093/inthealth/ihaa091.

23. Alfredsson J, Zary N. Use and usability of health related e-services among the senior citizens. Bio-Algorithms and Med-Systems. 2012 Mar;8(1):133-44. PMID: WOS:000453239000008. doi: 10.2478/bams-2012-0008.

24. Alharbi A, Alzuwaed J, Qasem H. Evaluation of e-health (Seha) application: a cross-sectional study in Saudi Arabia. BMC Med Inform Decis Mak. 2021 Mar 18;21(1):103. PMID: 33736622. doi: 10.1186/s12911-021-01437-6.

25. Ali A, Faisal A, Sorwar G, editors. Telemedicine framework for Bangladesh. TENCON 2010 - 2010 IEEE Region 10 Conference; 2010 21-24 Nov. 2010.

26. Alkmim MB, Figueira RM, Marcolino MS, Cardoso CS, de Abreu MP, Cunha LR, et al. Improving patient access to specialized health care: the Telehealth Network of Minas Gerais, Brazil. Bulletin of the World Health Organization. 2012 May;90(5):373-8. PMID: WOS:000303784900012. doi: 10.2471/blt.11.099408.

27. Almond H, Cummings E, Turner P. Avoiding Failure for Australia's Digital Health Record: The Findings from a Rural E-Health Participatory Research Project. In: Georgiou A, Schaper LK, Whetton S, editors. Digital Health Innovation for Consumers, Clinicians, Connectivity and Community. Amsterdam: Ios Press; 2016. p. 8-13.

28. Almond H, Cummings E, Turner P. An Approach for Enhancing Adoption, Use and Utility of Shared Digital Health Records in Rural Australian Communities. In: Randell R, Cornet R, McCowan C, Peek N, Scott PJ, editors. Informatics for Health: Connected Citizen-Led Wellness and Population Health. Amsterdam: Ios Press; 2017. p. 378-82.

29. Almond H, Cummings E, Turner P. Recommendations for Enhancing the Implementation and Utility of Shared Digital Health Records in Rural Australian Communities. In: Cummings E, Ryan A, Schaper LK, editors. Connecting the System to Enhance the Practitioner and Consumer Experience in Healthcare. Amsterdam: Ios Press; 2018. p. 15-20.

30. Alsaffar M, Yellowlees P, Odor A, Hogarth M. The State of Open Source Electronic Health Record Projects: A Software Anthropology Study. JMIR Med Inf. 2017 Jan-Mar;5(1):10. PMID: WOS:000395895000006. doi: 10.2196/medinform.5783.

31. Alsahafi YA, Gay V, Khwaji AA. Factors affecting the acceptance of integrated electronic personal health records in Saudi Arabia: The impact of e-health literacy. Health Inf Manag. 2020 Nov 28:1833358320964899. PMID: 33249857. doi: 10.1177/1833358320964899.

32. Alsaleh MM, Watzlaf VJM, DeAlmeida DR, Saptono A. Evaluation of a telehealth application (SEHHA) used during the Covid-19 pandemic in Saudi Arabia: Provider experience and satisfaction. Perspect Health Inf Manag. 2021 Fall;18(4):1b. PMID: 34975351.

33. Alsulame K, Khalifa M, Househ M. eHealth in Saudi Arabia: Current Trends, Challenges and Recommendations. In: Mantas J, Hasman A, Househ MS, editors. Enabling Health Informatics Applications. Amsterdam: Ios Press; 2015. p. 233-6.

34. Altmann S, Milsom L, Zillessen H, Blasone R, Gerdon F, Bach R, et al. Acceptability of App-Based Contact Tracing for COVID-19: Cross-Country Survey Study. JMIR Mhealth Uhealth. 2020 Aug 28;8(8):e19857. PMID: 32759102. doi: 10.2196/19857.

35. Alyoubi R, Kobeisy S, Elkady A, Bamusa M, Alotaibi S, Muthaffar O, et al. Implementation of Virtual Consultations for Epilepsy during the COVID-19 Pandemic among Neurologists in Saudi Arabia. Med Sci. 2020 Sep-Oct;24(105):3717-23. PMID: WOS:000604259000096.

36. Amirfar S, Anane S, Buck M, Cohen R, Di Lonardo S, Maa P, et al. Study of electronic prescribing rates and barriers identified among providers using electronic health records in New York City. Inform Prim Care. 2011;19(2):91-7. PMID: 22417819. doi: 10.14236/jhi.v19i2.800.

37. Ancker JS, Barron Y, Rockoff ML, Hauser D, Pichardo M, Szerencsy A, et al. Use of an Electronic Patient Portal Among Disadvantaged Populations. J Gen Intern Med. 2011 Oct;26(10):1117-23. PMID: WOS:000295329600011. doi: 10.1007/s11606-011-1749-y.

38. Ancker JS, Silver M, Kaushal R. Rapid Growth in Use of Personal Health Records in New York, 2012-2013. J Gen Intern Med. 2014 Jun;29(6):850-4. PMID: WOS:000338206600010. doi: 10.1007/s11606-014-2792-2.

39. Ancker JS, Singh MP, Thomas R, Edwards A, Snyder A, Kashyap A, et al. Predictors of success for electronic health record implementation in small physician practices. Appl Clin Inform. 2013;4(1):12-24. PMID: WOS:000317184500002. doi: 10.4338/aci-2012-09-ra-0033.

40. Anderson JG. Social, ethical and legal barriers to E-health. International Journal of Medical Informatics. 2007 May-Jun;76(5-6):480-3. PMID: WOS:000246320500025. doi: 10.1016/j.ijmedinf.2006.09.016.

41. Apolinario-Hagen J, Hennemann S, Kuck C, Wodner A, Geibel D, Riebschlager M, et al. Exploring User-Related Drivers of the Early Acceptance of Certified Digital Stress Prevention Programs in Germany. Health Services Insights. 2020 Mar;13:11. PMID: WOS:000524527000001. doi: 10.1177/1178632920911061.

42. Aries P, Welcker M, Callhoff J, Chehab G, Krusche M, Schneider M, et al. [Statement of the German Society for Rheumatology (DGRh) on the use of video consultations in rheumatology]. Z Rheumatol. 2020 Dec;79(10):1078-85. PMID: 33201305. doi: 10.1007/s00393-020-00932-x.

43. Armstrong AW, Kwong MW, Ledo L, Nesbitt TS, Shewry SL. Practice models and challenges in teledermatology: a study of collective experiences from teledermatologists. PLoS One. 2011;6(12):e28687. PMID: 22194887. doi: 10.1371/journal.pone.0028687.

44. Armstrong AW, Sanders C, Farbstein AD, Wu GZ, Lin SW, Liu FT, et al. Evaluation and Comparison of Store-and-Forward Teledermatology Applications. Telemed J E Health. 2010 May;16(4):424-38. PMID: WOS:000278107000043. doi: 10.1089/tmj.2009.0133.

45. Asadi F, Moghaddasi H, Rabiei R, Rahimi F, Mirshekarlou SJ. The Evaluation of SEPAS National Project Based on Electronic Health Record System (EHRS) Coordinates in Iran. Acta Inform Med. 2015 Dec;23(6):369-73. PMID: 26862248. doi: 10.5455/aim.2015.23.369-373.

46. Asangansi I, Braa K. The Emergence of Mobile-Supported National Health Information Systems in Developing Countries. In: Safran C, Reti S, Marin HF, editors. Medinfo 2010, Pts I and Ii. Amsterdam: Ios Press; 2010. p. 540-4.

47. Avdagovska M, Stafinski T, Ballermann M, Menon D, Olson K, Paul P. Tracing the Decisions That Shaped the Development of MyChart, an Electronic Patient Portal in Alberta, Canada: Historical Research Study. J Med Internet Res. 2020 May 26;22(5):e17505. PMID: 32452811. doi: 10.2196/17505.

48. Avdoshin SM, Pesotskaya EY. Mobile healthcare: Perspectives in Russia. Biznes Informatika-Business Informatics. 2016;37(3):38-44. PMID: WOS:000406496000004. doi: 10.17323/1998-0663.2016.3.38.44.

49. Bae J, Encinosa WE. National estimates of the impact of electronic health records on the workload of primary care physicians. BMC Health Serv Res. 2016 May;16:11. PMID: WOS:000376572000001. doi: 10.1186/s12913-016-1422-6.

50. Bae J, Hockenberry JM, Rask KJ, Becker ER. Evidence that electronic health records can promote physician counseling for healthy behaviors. Health Care Manage Rev. 2017 Jul-Sep;42(3):258-68. PMID: WOS:000402539600008. doi: 10.1097/hmr.0000000000000108.

51. Bagayoko CO, Tchuente J, Traore D, Lipenguet GM, Mbenga RO, Koumamba AP, et al. Implementation of a national electronic health information system in Gabon: a survey of healthcare providers' perceptions. Bmc Medical Informatics and Decision Making. 2020 Aug;20(1):9. PMID: WOS:000567133600001. doi: 10.1186/s12911-020-01213-y.

52. Bagot KL, Bladin CF, Vu M, Kim J, Hand PJ, Campbell B, et al. Exploring the benefits of a stroke telemedicine programme: An organisational and societal perspective. J Telemed Telecare. 2016 Dec;22(8):489-94. PMID: WOS:000387352400009. doi: 10.1177/1357633x16673695.

53. Bai VT, Murali V, Kim R, Srivatsa SK. Teleophthalmology-based rural eye care in India. Telemed J E Health. 2007 Jun;13(3):313-21. PMID: 17603834. doi: 10.1089/tmj.2006.0048.

54. Bangs I, Baldwin LP, Clarke M, Hands L, Jones RW, Maheffey W. A technology-assisted approach to integrating healthcare in the community. Telemed J E Health. 2003 Sum;9(2):215-21. PMID: WOS:000183766400010. doi: 10.1089/153056203766437552.

55. Bardsley M, Steventon A, Doll H. Impact of telehealth on general practice contacts: findings from the whole systems demonstrator cluster randomised trial. BMC Health Serv Res. 2013 Oct;13:9. PMID: WOS:000328116200001. doi: 10.1186/1472-6963-13-395.

56. Barnett S, Henderson J, Hodgkins A, Harrison C, Ghosh A, Dijkmans-Hadley B, et al. A valuable approach to the use of electronic medical data in primary care research: Panning for gold. Health Information Management Journal. 2017 May;46(2):51-7. PMID: WOS:000399486700001. doi: 10.1177/1833358316669888.

57. Bashingwa JJH, Mohan D, Chamberlain S, Arora S, Mendiratta J, Rahul S, et al. Assessing exposure to Kilkari: a big data analysis of a large maternal mobile messaging service across 13 states in India. BMJ Glob Health. 2021 Jul;6(Suppl 5). PMID: 34312148. doi: 10.1136/bmjgh-2021-005213.

58. Baskaya M, Yuksel M, Erturkmen GBL, Cunningham M, Cunningham P. Health4Afrika - Implementing HL7 FHIR Based Interoperability. Stud Health Technol Inform. 2019 Aug 21;264:20-4. PMID: 31437877. doi: 10.3233/shti190175.

59. Bates DW. Physicians and ambulatory electronic health records. Health Affairs. 2005 Sep-Oct;24(5):1180-9. PMID: WOS:000235033400014. doi: 10.1377/hlthaff.24.5.1180.

60. Beckers R. [Regional and nationwide developments in telemedicine. A contradiction?]. Bundesgesundheitsblatt Gesundheitsforschung Gesundheitsschutz. 2015 Oct;58(10):1074-8. PMID: 26275570. doi: 10.1007/s00103-015-2225-3.

61. Belden CM, Proeschold-Bell RJ. A Comparison of the Adoption of Electronic Health Records in North Carolina and South Carolina HIV Systems. South Med J. 2010 Nov;103(11):1115-8. PMID: WOS:000283716400009. doi: 10.1097/SMJ.0b013e3181f69add.

62. Ben-Assuli O. Electronic health records, adoption, quality of care, legal and privacy issues and their implementation in emergency departments. Health Policy. 2015 Mar;119(3):287-97. PMID: WOS:000352245700006. doi: 10.1016/j.healthpol.2014.11.014.

63. Bennion MR, Hardy G, Moore RK, Millings A. E-therapies in England for stress, anxiety or depression: what is being used in the NHS? A survey of mental health services. BMJ Open. 2017 Jan 23;7(1):e014844. PMID: 28115336. doi: 10.1136/bmjopen-2016-014844.

64. Bhatia JS, Sharma S. Telemedicine odyssey customised telemedicine solution for rural and remote areas in India. Stud Health Technol Inform. 2006;121:22-35. PMID: 17095800.

65. Biggs JS, Willcocks A, Burger M, Makeham MA. Digital health benefits evaluation frameworks: building the evidence to support Australia's National Digital Health Strategy. Med J Aust. 2019 Apr;210 Suppl 6:S9-s11. PMID: 30927475. doi: 10.5694/mja2.50034.

66. Bikava I, Kreituse I. Interest group impact on E-health implementation in Latvia. In: Berkis U, Vilka L, editors. 6th International Interdisciplinary Scientific Conference Society Health Welfare. Cedex A: E D P Sciences; 2018.

67. Bitaraf E, Jafarpour M, Jami V, Sarani Rad F. The Iranian Integrated Care Electronic Health Record. Stud Health Technol Inform. 2021 May 27;281:654-8. PMID: 34042657. doi: 10.3233/shti210252.

68. Bjering H, Ginige A, Maeder A, Bensoussan A, Zhu XS, Lattuca C. Electronic Medical Record Information System for Patient Consultations in Chinese Medicine. In: Hansen DP, Maeder AJ, Schaper LK, editors. Health Informatics: The Transformative Power of Innovation. Amsterdam: Ios Press; 2011. p. 10-5.

69. Boffin N, Bossuyt N, Vanthomme K, Van Casteren V. Readiness of the Belgian network of sentinel general practitioners to deliver electronic health record data for surveillance purposes: results of survey study. BMC Fam Pract. 2010 Jun 25;11:50. PMID: 20579350. doi: 10.1186/1471-2296-11-50.

70. Bordowitz R. Electronic health records: A primer. Labmedicine. 2008 May;39(5):301-6. PMID: WOS:000255176800009. doi: 10.1309/0r3v3k6xq9tufah6.

71. Bouamrane M-M, Mair F. An overview of electronic health systems development & integration in Scotland. Proceedings of the first international workshop on Managing interoperability and complexity in health systems; Glasgow, Scotland, UK: Association for Computing Machinery; 2011. p. 59–62.

72. Brown EM. The Ontario Telemedicine Network: a case report. Telemed J E Health. 2013 May;19(5):373-6. PMID: 23301768. doi: 10.1089/tmj.2012.0299.

73. Bujnowska-Fedak MM, Wysoczański Ł. Access to an Electronic Health Record: A Polish National Survey. Int J Environ Res Public Health. 2020 Aug 25;17(17). PMID: 32854345. doi: 10.3390/ijerph17176165.

74. Carnicero R, Rojas D, Elicegui I, Carnicero J. Proposal of a Learning Health System to Transform the National Health System of Spain. Processes. 2019 Sep;7(9):23. PMID: WOS:000489121800066. doi: 10.3390/pr7090613.

75. Carroll J, Butler-Henderson K. MyHealthRecord in Australian Primary Health Care: An Attitudinal Evaluation Study. J Med Syst. 2017 Sep 2;41(10):158. PMID: 28866846. doi: 10.1007/s10916-017-0807-3.

76. Casey MM, Sorensen TD, Elias W, Knudson A, Gregg W. Current practices and state regulations regarding telepharmacy in rural hospitals. Am J Health Syst Pharm. 2010 Jul 1;67(13):1085-92. PMID: 20554595. doi: 10.2146/ajhp090531.

77. Chamberlain S, Dutt P, Godfrey A, Mitra R, LeFevre AE, Scott K, et al. Ten lessons learnt: scaling and transitioning one of the largest mobile health communication programmes in the world to a national government. BMJ Glob Health. 2021 Jul;6(Suppl 5). PMID: 34312151. doi: 10.1136/bmjgh-2021-005341.

78. Chasco EE, Shafer C, Dillon DMB, Owens S, Ohl ME, Hoth AB. Bringing Iowa TelePrEP to Scale: A Qualitative Evaluation. Am J Prev Med. 2021 Nov;61(5):S108-S17. PMID: WOS:000709462100013. doi: 10.1016/j.amepre.2021.05.040.

79. Cheong HJ, Shin NY, Joeng YB, editors. Improving Korean Service Delivery System in Health Care: Focusing on National E-health System. 2009 International Conference on eHealth, Telemedicine, and Social Medicine; 2009 1-7 Feb. 2009.

80. Chouinard I, Scott RE. Informed consent for videoconsultations in Canada. J Telemed Telecare. 2009;15(4):171-4. PMID: 19471027. doi: 10.1258/jtt.2008.080905.

81. Cilliers L, Flowerday SV. Health information systems to improve health care: A telemedicine case study. South African Journal of Information Management. 2013;15(1):5. PMID: WOS:000217243200003. doi: 10.4102/sajim.v15i1.541.

82. Clarke M, Fursse J, Brown-Connolly NE, Sharma U, Jones R. Evaluation of the National Health Service (NHS) Direct Pilot Telehealth Program: Cost-Effectiveness Analysis. Telemed J E Health. 2018 Jan;24(1):67-76. PMID: 28723244. doi: 10.1089/tmj.2016.0280.

83. Clarke MA, Lyden ER, Ma J, King KM, Siahpush MM, Michaud T, et al. Sociodemographic Differences and Factors Affecting Patient Portal Utilization. J Racial Ethn Health Disparities. 2020 Aug 24. PMID: 32839896. doi: 10.1007/s40615-020-00846-z.

84. Cocoros NM, Fuller CC, Adimadhyam S, Ball R, Brown JS, Dal Pan GJ, et al. A COVID-19-ready public health surveillance system: The Food and Drug Administration's Sentinel System. Pharmacoepidemiol Drug Saf. 2021 Jul;30(7):827-37. PMID: 33797815. doi: 10.1002/pds.5240.

85. Colicchio TK, Cimino JJ, Del Fiol G. Unintended Consequences of Nationwide Electronic Health Record Adoption: Challenges and Opportunities in the Post-Meaningful Use Era. J Med Internet Res. 2019 Jun;21(6):9. PMID: WOS:000470667600001. doi: 10.2196/13313.

86. Collen MF, Hammond WE. Development of Medical Information Systems (MISs). In: Collen MF, Ball MJ, editors. History of Medical Informatics in the United States. New York: Springer; 2015. p. 123-206.

87. Cresswell K, Callaghan M, Mozaffar H, Sheikh A. NHS Scotland's Decision Support Platform: a formative qualitative evaluation. BMJ Health Care Inform. 2019 May;26(1). PMID: 31160318. doi: 10.1136/bmjhci-2019-100022.

88. Cresswell K, Sheikh A, Franklin BD, Krasuska M, The Nguyen H, Hinder S, et al. Interorganizational Knowledge Sharing to Establish Digital Health Learning Ecosystems: Qualitative Evaluation of a National Digital Health Transformation Program in England. J Med Internet Res. 2021 Aug 19;23(8):e23372. PMID: 34420927. doi: 10.2196/23372.

89. Cresswell KM, Robertson A, Sheikh A. Lessons learned from England's national electronic health record implementation: implications for the international community. Proceedings of the 2nd ACM SIGHIT International Health Informatics Symposium; Miami, Florida, USA: Association for Computing Machinery; 2012. p. 685–90.

90. Cresswell KM, Worth A, Sheikh A. Integration of a nationally procured electronic health record system into user work practices. BMC Med Inform Decis Mak. 2012 Mar 8;12:15. PMID: 22400978. doi: 10.1186/1472-6947-12-15.

91. Crudo F, Fernandez M, Fermepin MR, Entrocassi AC, Cardone KA, Markmann FS, et al. Impact of a public health intervention for active surveillance and mitigation of SARS-CoV-2 in a district from Buenos Aires province, Argentina: a descriptive epidemiological study. Bmj Open. 2021 Nov;11(11):9. PMID: WOS:000724352600030. doi: 10.1136/bmjopen-2021-053595.

92. de la Torre-Díez I, González S, López-Coronado M. EHR systems in the Spanish Public Health National System: the lack of interoperability between primary and specialty care. J Med Syst. 2013 Feb;37(1):9914. PMID: 23321962. doi: 10.1007/s10916-012-9914-3.

93. De Pietro C, Francetic I. E-health in Switzerland: The laborious adoption of the federal law on electronic health records (EHR) and health information exchange (HIE) networks. Health Policy. 2018 Feb;122(2):69-74. PMID: WOS:000425080300001. doi: 10.1016/j.healthpol.2017.11.005.

94. Dehelean S, Sauciuc DG, Miclea L, Hoka I, editors. EN13606 overview and compatibility with the Romanian healthcare system. 2008 IEEE International Conference on Automation, Quality and Testing, Robotics; 2008 22-25 May 2008.

95. Demeke HB, Pao LZ, Clark H, Romero L, Neri A, Shah R, et al. Telehealth Practice Among Health Centers During the COVID-19 Pandemic - United States, July 11-17, 2020. MMWR Morb Mortal Wkly Rep. 2020 Dec 18;69(50):1902-5. PMID: 33332297. doi: 10.15585/mmwr.mm6950a4.

96. deShazo RD, Parker SB. Lessons Learned from Mississippi's Telehealth Approach to Health Disparities. Am J Med. 2017 Apr;130(4):403-8. PMID: 27899245. doi: 10.1016/j.amjmed.2016.11.005.

97. DesRoches CM, Campbell EG, Rao SR, Donelan K, Ferris TG, Jha A, et al. Electronic health records in ambulatory care - A national survey of physicians. New England Journal of Medicine. 2008 Jul;359(1):50-60. PMID: WOS:000257246000007. doi: 10.1056/NEJMsa0802005.

98. DesRoches CM, Charles D, Furukawa MF, Joshi MS, Kralovec P, Mostashari F, et al. Adoption of electronic health records grows rapidly, but fewer than half of US hospitals had at least a basic system in 2012. Health Aff (Millwood). 2013 Aug;32(8):1478-85. PMID: 23840052. doi: 10.1377/hlthaff.2013.0308.

99. Deters FG, Meier T, Milek A, Horn AB. Self-Focused and Other-Focused Health Concerns as Predictors of the Uptake of Corona Contact Tracing Apps: Empirical Study. J Med Internet Res. 2021 Aug;23(8):15. PMID: WOS:000683837700005. doi: 10.2196/29268.

100. Dias RD, Marques ADH, Diniz PRB, da Silva TAB, Cofiel L, Mariani MMD, et al. Telemental health in Brazil: past, present and integration into primary care. Revista De Psiquiatria Clinica. 2015;42(2):41-4. PMID: WOS:000356011700003. doi: 10.1590/0101-60830000000046.

101. Dowie R, Mistry H, Rigby M, Young TA, Weatherburn G, Rowlinson G, et al. A paediatric telecardiology service for district hospitals in south-east England: an observational study. Arch Dis Child. 2009 Apr;94(4):273-7. PMID: 18786954. doi: 10.1136/adc.2008.138495.

102. Eberly LA, Kallan MJ, Julien HM, Haynes N, Khatana SAM, Nathan AS, et al. Patient Characteristics Associated With Telemedicine Access for Primary and Specialty Ambulatory Care During the COVID-19 Pandemic. JAMA Netw Open. 2020 Dec 1;3(12):e2031640. PMID: 33372974. doi: 10.1001/jamanetworkopen.2020.31640.

103. El-Mahalli AA. Adoption and Barriers to Adoption of Electronic Health Records by Nurses in Three Governmental Hospitals in Eastern Province, Saudi Arabia. Perspect Health Inf Manag. 2015;12(Fall):1f. PMID: 26604875.

104. El-Toukhy S, Méndez A, Collins S, Pérez-Stable EJ. Barriers to Patient Portal Access and Use: Evidence from the Health Information National Trends Survey. J Am Board Fam Med. 2020 Nov-Dec;33(6):953-68. PMID: 33219074. doi: 10.3122/jabfm.2020.06.190402.

105. Ellingsen G, Christensen B, Hertzum M. Persuasion Tactics in the Implementation of Large-Scale EHR Suites in Public Healthcare. Stud Health Technol Inform. 2021 Nov 8;286:33-7. PMID: 34755686. doi: 10.3233/shti210632.

106. Essen A, Gerrits R, Kuhlmann E. Patient accessible electronic health records: Connecting policy and provider action in the Netherlands. Health Policy Technol. 2017 Jun;6(2):134-41. PMID: WOS:000403988700003. doi: 10.1016/j.hlpt.2017.03.001.

107. Fareed N, Bazzoli GJ, Mick SSF, Harless DW. The influence of institutional pressures on hospital electronic health record presence. Soc Sci Med. 2015 May;133:28-35. PMID: WOS:000354579800005. doi: 10.1016/j.socscimed.2015.03.047.

108. Felix H, Dayama N, Morris ME, Pradhan R, Bradway C. Organizational Characteristics and the Adoption of Electronic Health Records Among Nursing Homes in One Southern State. J Appl Gerontol. 2020 Feb 21:733464820906685. PMID: 32081058. doi: 10.1177/0733464820906685.

109. Findikoglu M, Watson-Manheim MB. Linking macro-level goals to micro-level routines: EHR-enabled transformation of primary care services. Journal of Information Technology. 2016 Dec;31(4):382-400. PMID: WOS:000391900900005. doi: 10.1057/s41265-016-0023-5.

110. Findlay I, Morris T, Zhang RQ, McCowan C, Shield S, Forbes B, et al. Linking hospital patient records for suspected or established acute coronary syndrome in a complex secondary care system: a proof-of-concept e-registry in National Health Service Scotland. European Heart Journal-Quality of Care and Clinical Outcomes. 2018 Jul;4(3):155-67. PMID: WOS:000442854900005. doi: 10.1093/ehjqcco/qcy007.

111. Fonseca M, Karkaletsis K, Cruz IA, Berler A, Oliveira IC. OpenNCP: a novel framework to foster cross-border e-Health services. Stud Health Technol Inform. 2015;210:617-21. PMID: 25991222.

112. Fontaine P, Zink T, Boyle RG, Kralewski J. Health information exchange: participation by Minnesota primary care practices. Arch Intern Med. 2010 Apr 12;170(7):622-9. PMID: 20386006. doi: 10.1001/archinternmed.2010.54.

113. Fragidis LL, Chatzoglou PD. Development of Nationwide Electronic Health Record (NEHR): An international survey. Health Policy Technol. 2017 Jun;6(2):124-33. PMID: WOS:000403988700002. doi: 10.1016/j.hlpt.2017.04.004.

114. Fragidis LL, Chatzoglou PD. Implementation of a nationwide electronic health record (EHR): The international experience in 13 countries. Int J Health Care Qual Assur. 2018;31(2):116-30. PMID: WOS:000426687000004. doi: 10.1108/ijhcqa-09-2016-0136.

115. Fragidis LL, Chatzoglou PD, Aggelidis VP. Integrated Nationwide Electronic Health Records system: Semi-distributed architecture approach. Technol Health Care. 2016 Nov 14;24(6):827-42. PMID: 27392830. doi: 10.3233/thc-161231.

116. Friedman DJ. Assessing the potential of national strategies for electronic health records for population health monitoring and research. Vital Health Stat 2. 2006 Jan(143):1-83. PMID: 17552126.

117. Friedman RH. Medicaid information technology architecture: an overview. Health Care Financ Rev. 2006 Winter;28(2):1-9. PMID: 17427840.

118. Frielitz FS, Storm N, Hiort O, Katalinic A, von Sengbusch S. [The creation of a data protection policy: a guide to telemedicine healthcare projects]. Bundesgesundheitsblatt Gesundheitsforschung Gesundheitsschutz. 2019 Apr;62(4):479-85. PMID: 30874830. doi: 10.1007/s00103-019-02918-w.

119. Fuentes AF, Botero DF, Ramirez CT, editors. A Covid-19 Vaccination Tracking and Control Platform in Santiago de Cali. 4th Ibero-American Congress on Smart Cities (ICSC-CITIES); 2021 Nov 29-Dec 01; Cancun, MEXICO. CHAM: Springer International Publishing Ag; 2022.

120. Fuji KT, Gait KA, Siracuse MV, Christoffersen JS. Electronic health record adoption and use by Nebraska pharmacists. Perspect Health Inf Manag. 2011;8(Summer):1d. PMID: 21796266.

121. Fuji KT, Galt KA, Serocca AB. Personal health record use by patients as perceived by ambulatory care physicians in Nebraska and South Dakota: a cross-sectional study. Perspect Health Inf Manag. 2008;5:15. PMID: 18927602.

122. Gabriel MH, Jones EB, Samy L, King J. Progress And Challenges: Implementation And Use Of Health Information Technology Among Critical-Access Hospitals. Health Affairs. 2014 Jul;33(7):1262-70. PMID: WOS:000340469700022. doi: 10.1377/hlthaff.2014.0279.

123. Gagnon MP, Payne-Gagnon J, Breton E, Fortin JP, Khoury L, Dolovich L, et al. Adoption of Electronic Personal Health Records in Canada: Perceptions of Stakeholders. Int J Health Policy Manag. 2016 Jul;5(7):425-33. PMID: WOS:000379825200004. doi: 10.15171/ijhpm.2016.36.

124. Ganapathy K, Alagappan D, Rajakumar H, Dhanapal B, Subbu GR, Nukala L, et al. Tele-Emergency Services in the Himalayas. Telemed e-Health. 2019 May;25(5):380-90. PMID: WOS:000439662200001. doi: 10.1089/tmj.2018.0027.

125. Ganapathy K, Chawdhry V, Premanand S, Sarma A, Chandralekha J, Kumar KY, et al. Telemedicine in the Himalayas: Operational Challenges-A Preliminary Report. Telemed J E Health. 2016 Oct;22(10):821-35. PMID: 27135412. doi: 10.1089/tmj.2015.0249.

126. Garfield MJ, Watson RT. Four case studies in state-supported telemedicine initiatives. Telemed J E Health. 2003 Sum;9(2):197-205. PMID: WOS:000183766400008. doi: 10.1089/153056203766437534.

127. Garrety K, McLoughlin I, Wilson R, Zelle G, Martin M. National electronic health records and the digital disruption of moral orders. Soc Sci Med. 2014 Jan;101:70-7. PMID: WOS:000332906800009. doi: 10.1016/j.socscimed.2013.11.029.

128. Garrety K, McLoughlin I, Zelle G. Disruptive Innovation in Health Care: Business Models, Moral Orders and Electronic Records. Social Policy and Society. 2014 Oct;13(4):579-92. PMID: WOS:000212089900008. doi: 10.1017/s1474746413000560.

129. Ge FM, Qian H, Lei JB, Ni YQ, Li Q, Wang S, et al. Experiences and Challenges of Emerging Online Health Services Combating COVID-19 in China: Retrospective, Cross-Sectional Study of Internet Hospitals. JMIR Med Inf. 2022 Jun;10(6):19. PMID: WOS:000809331100003. doi: 10.2196/37042.

130. Geiger MF, Wilhelmy S, Schmidt M, Firsching R, Gross D, Clusmann H. Current Practice of Neurosurgical Teleconsultation in Germany. Journal of Neurological Surgery Part a-Central European Neurosurgery. 2020 Nov;81(06):521-8. PMID: WOS:000567831900007. doi: 10.1055/s-0040-1710505.

131. Gheorghiu B, Ratchford F. Scaling up the use of remote patient monitoring in Canada. Stud Health Technol Inform. 2015;209:23-6. PMID: 25980701.

132. Ginsburg OM, Chowdhury M, Wu W, Chowdhury M, Pal BC, Hasan R, et al. An mHealth Model to Increase Clinic Attendance for Breast Symptoms in Rural Bangladesh: Can Bridging the Digital Divide Help Close the Cancer Divide? Oncologist. 2014 Feb;19(2):177-85. PMID: WOS:000332077300010. doi: 10.1634/theoncologist.2013-0314.

133. Glassman P, Helgeson M, Kattlove J. Using telehealth technologies to improve oral health for vulnerable and underserved populations. J Calif Dent Assoc. 2012 Jul;40(7):579-85. PMID: 22916379.

134. Golbeck AL, Hansen D, Lee K, Noblitt V, Christner J, Pinsonneault J. Telemonitoring improves home health utilization outcomes in rural settings. J Telemed Telecare. 2011;17(5):273-8. PMID: 21824969. doi: 10.1258/jtt.2011.100807.

135. Gonzalez C. Integrated telemedicine system in Andalusia. Iecon-2002: Proceedings of the 2002 28th Annual Conference of the Ieee Industrial Electronics Society, Vols 1-4. New York: Ieee; 2002. p. 3398-402.

136. Gordon NP, Hornbrook MC. Differences in Access to and Preferences for Using Patient Portals and Other eHealth Technologies Based on Race, Ethnicity, and Age: A Database and Survey Study of Seniors in a Large Health Plan. J Med Internet Res. 2016 Mar;18(3):28. PMID: WOS:000380777800002. doi: 10.2196/jmir.5105.

137. Gowda GS, Kulkarni K, Bagewadi V, Shyam RPS, Manjunatha BR, Shashidhara HN, et al. A study on collaborative telepsychiatric consultations to outpatients of district hospitals of Karnataka, India. Asian J Psychiatr. 2018 Oct;37:161-6. PMID: WOS:000448471300031. doi: 10.1016/j.ajp.2018.09.003.

138. Greenberg AJ, Falisi AL, Rutten LJF, Chou WYS, Patel V, Moser RP, et al. Access to Electronic Personal Health Records Among Patients With Multiple Chronic Conditions: A Secondary Data Analysis. J Med Internet Res. 2017 Jun;19(6):13. PMID: WOS:000403145600009. doi: 10.2196/jmir.7417.

139. Greenberg AJ, Haney D, Blake KD, Moser RP, Hesse BW. Differences in Access to and Use of Electronic Personal Health Information Between Rural and Urban Residents in the United States. J Rural Health. 2018 Feb;34:S30-S8. PMID: WOS:000425117300004. doi: 10.1111/jrh.12228.

140. Greenhalgh T, Hinder S, Stramer K, Bratan T, Russell J. Adoption, non-adoption, and abandonment of a personal electronic health record: case study of HealthSpace. Bmj-British Medical Journal. 2010 Nov;341:11. PMID: WOS:000284585600010. doi: 10.1136/bmj.c5814.

141. Greenhalgh T, Shaw S, Wherton J, Vijayaraghavan S, Morris J, Bhattacharya S, et al. Real-World Implementation of Video Outpatient Consultations at Macro, Meso, and Micro Levels: Mixed-Method Study. J Med Internet Res. 2018 Apr 17;20(4):e150. PMID: 29625956. doi: 10.2196/jmir.9897.

142. Greis C, Maul LV, Hsu C, Djamei V, Schmid-Grendelmeier P, Navarini AA. Artificial intelligence to support telemedicine in Africa. Hautarzt. 2020 Sep;71(9):686-90. PMID: WOS:000556612400001. doi: 10.1007/s00105-020-04664-6.

143. Grigsby B, Brega AG, Bennett RE, Devore PA, Paulich MJ, Talkington SG, et al. The slow pace of interactive video telemedicine adoption: The perspective of telemedicine program administrators on physician participation. Telemed J E Health. 2007 Dec;13(6):645-56. PMID: WOS:000254067400022. doi: 10.1089/tmj.2007.0090.

144. Grinspan ZM, Banerjee S, Kaushal R, Kern LM. Physician Specialty and Variations in Adoption of Electronic Health Records. Appl Clin Inform. 2013;4(2):225-40. PMID: WOS:000323701800006. doi: 10.4338/aci-2013-02-ra-0015.

145. Gummadi S, Housri N, Zimmers TA, Koniaris LG. Electronic Medical Record: A Balancing Act of Patient Safety, Privacy and Health Care Delivery. Am J Med Sci. 2014 Sep;348(3):238-43. PMID: WOS:000342770200011. doi: 10.1097/maj.0000000000000287.

146. Gupta A, Agrawal R, Gupt A, Guleri R, Bajpayee D, Joshi N, et al. Systems E-approach for women at risk (SEWA)-A digital health solution for detection of high-risk pregnancies. J Fam Med Prim Care. 2021 Oct;10(10):3712-9. PMID: WOS:000751457500030. doi: 10.4103/jfmpc.jfmpc_466_21.

147. Gupta M, Chotard L, Ingporsson O, Bastos J, Borges I. Health@Home - An e-Service Model for Disease Prevention and Healthcare in the Home. In: Weerasinghe D, editor. Electronic Healthcare. New York: Springer; 2009. p. 17-+.

148. Haddad AE, Skelton-Macedo MC, Abdala V, Bavaresco C, Mengehel D, Abdala CG, et al. Formative Second Opinion: Qualifying Health Professionals for the Unified Health System Through the Brazilian Telehealth Program. Telemed J E Health. 2015 Feb;21(2):138-42. PMID: 25493611. doi: 10.1089/tmj.2014.0001.

149. Hailey D, Bulger T, Stayberg S, Urness D. The evolution of a successful telemedicine mental health service. J Telemed Telecare. 2002;8:24-6. PMID: WOS:000179744700011. doi: 10.1258/13576330260440754.

150. Hamamura FD, Withy K, Hughes K. Identifying Barriers in the Use of Electronic Health Records in Hawai'i. Hawaii J Med Public Health. 2017 Mar;76(3 Suppl 1):28-35. PMID: 28435756.

151. Hanssen B, Wangberg SC, Gammon D. Use of videoconferencing in Norwegian psychiatry. J Telemed Telecare. 2007;13(3):130-5. PMID: WOS:000246265300006. doi: 10.1258/135763307780677587.

152. Hayrinen K, Saranto K. The Core Data Elements of Electronic Health Record in Finland. In: Engelbrecht R, Geissbuhler A, Lovis C, Mihalas G, editors. Connecting Medical Informatics and Bio-Informatics. Amsterdam: I O S Press; 2005. p. 131-6.

153. Heaney D, Caldow J, McClusky C, King G, Webster K, Mair F, et al. The Introduction of a New Consulting Technology into the National Health Service (NHS) for Scotland. Telemed J E Health. 2009 Aug;15(6):546-51. PMID: WOS:000268744600009. doi: 10.1089/tmj.2009.0017.

154. Heimly V, Grimsmo A, Faxvaag A. Diffusion of Electronic Health Records and electronic communication in Norway. Appl Clin Inform. 2011;2(3):355-64. PMID: WOS:000208686700010. doi: 10.4338/aci-2011-01-ie-0008.

155. Heimly V, Grimsmo A, Henningsen TP, Faxvaag A. Diffusion and use of Electronic Health Record systems in Norway. Stud Health Technol Inform. 2010;160(Pt 1):381-5. PMID: 20841713.

156. Heisey-Grove D, Wall HK, Helwig A, Wright JS. Using electronic clinical quality measure reporting for public health surveillance. MMWR Morb Mortal Wkly Rep. 2015 May 1;64(16):439-42. PMID: 25928469.

157. Hendy J, Chrysanthaki T, Barlow J, Knapp M, Rogers A, Sanders C, et al. An organisational analysis of the implementation of telecare and telehealth: the whole systems demonstrator. BMC Health Serv Res. 2012 Nov;12:10. PMID: WOS:000312875500001. doi: 10.1186/1472-6963-12-403.

158. Holt B, Faraklas I, Theurer L, Cochran A, Saffle JR. Telemedicine use among burn centers in the United States: a survey. J Burn Care Res. 2012 Jan-Feb;33(1):157-62. PMID: 22105096. doi: 10.1097/BCR.0b013e31823d0b68.

159. Howley MJ, Chou EY, Hansen N, Dalrymple PW. The long-term financial impact of electronic health record implementation. Journal of the American Medical Informatics Association. 2015 Mar;22(2):443-52. PMID: WOS:000352771500022. doi: 10.1136/amiajnl-2014-002686.

160. Hsiao CJ, Hing E, Ashman J. Trends in electronic health record system use among office-based physicians: United States, 2007-2012. Natl Health Stat Report. 2014 May 20(75):1-18. PMID: 24844589.

161. Huang EW, Hung RS, Chiou SF, Liu FY, Liou DM. Design and development of a tele-healthcare information system based on web services and HL7 standards. Adv Exp Med Biol. 2011;696:599-606. PMID: 21431601. doi: 10.1007/978-1-4419-7046-6_61.

162. Huilgol YS, Miron-Shatz T, Joshi AU, Hollander JE. Hospital Telehealth Adoption Increased in 2014 and 2015 and Was Influenced by Population, Hospital, and Policy Characteristics. Telemed e-Health. 2020 Apr;26(4):455-61. PMID: WOS:000468780800001. doi: 10.1089/tmj.2019.0029.

163. Iasbech PAB, Lavarda RAB. Strategy and practices: A qualitative study of a Brazilian public healthcare system of telemedicine. International Journal of Public Sector Management. 2018;31(3):347-71. PMID: WOS:000428169400004. doi: 10.1108/ijpsm-12-2016-0207.

164. Ilin I, Iliashenko V, Iliashenko O. Information exchange model for remote consulting systems in the Russian Federation. International Science Conference Spbwosce-2018: Business Technologies for Sustainable Urban Development. Cedex A: E D P Sciences; 2019.

165. Jalal-Karim A, Balachandran W. The National Strategies for Electronic Health Record in three developed countries: General Status. New York: Ieee; 2008. 132-8 p. ISBN: 978-1-4244-2823-6.

166. Jeganathan VS, Hall HN, Sanders R. Electronic Referrals and Digital Imaging Systems in Ophthalmology: A Global Perspective. Asia Pac J Ophthalmol (Phila). 2017 Jan-Feb;6(1):3-7. PMID: 28161930. doi: 10.22608/apo.2016110.

167. Jensen C, McKerrow NH. The feasibility and ongoing use of electronic decision support to strengthen the implementation of IMCI in KwaZulu-Natal, South Africa. BMC Pediatr. 2022 Feb 7;22(1):80. PMID: 35130847. doi: 10.1186/s12887-022-03147-y.

168. Jones RW. Diabetes and Telehealth in China: Diagnoses, Treatment and Intervention. New York: Ieee; 2018. 148-53 p. ISBN: 978-1-5386-7743-8.

169. Joshi NK, Bhardwaj P, Suthar P, Jain YK, Joshi V, Singh K. Overview of e-Health initiatives in Rajasthan: An exploratory study. J Fam Med Prim Care. 2021 Mar;10(3):1369-76. PMID: WOS:000648447400050. doi: 10.4103/jfmpc.jfmpc_1989_20.

170. Kenoui M, Belgacem K, Chaffa G, Bouderbala FZ, Lakhneche R, Oudjoudi I, editors. First Steps Toward a Full-Web National Telemedicine Portal. 2020 2nd International Workshop on Human-Centric Smart Environments for Health and Well-being (IHSH); 2021 9-10 Feb. 2021.

171. Khaliq AA, Mwachofi AK, Hughes DR, Broyles RW, Wheeler D, Roswell RH. The current state of electronic health record (EHR) use in Oklahoma. J Okla State Med Assoc. 2013 Feb;106(2):53-6. PMID: 23620983.

172. Kierkegaard P. Mapping Telemedicine Efforts: Surveying Regional Initiatives in Denmark. Telemed e-Health. 2015 May;21(5):427-35. PMID: WOS:000354099600012. doi: 10.1089/tmj.2014.0123.

173. Kifle M, Mbarika VWA, Bradley RV. Global diffusion of the Internet X: The diffusion of telemedicine in Ethiopia: Potential benefits, present challenges, and potential factors. Communications of the Association for Information Systems. 2006;18:612-40. PMID: WOS:000414835200030.

174. Kim K, Seok H, Proctor RW. Comparison of national e-health implementation in the United States and South Korea. Human Factors and Ergonomics in Manufacturing & Service Industries. 2016 Dec;26(6):692-9. PMID: WOS:000385873700005. doi: 10.1002/hfm.20408.

175. Kim KK, Rudin RS, Wilson MD. Health Information Technology Adoption in California Community Health Centers. Am J Manag Care. 2015 Dec;21(12):E677-E83. PMID: WOS:000369388100008.

176. Klaib AF, Nuser MS. Evaluating EHR and Health Care in Jordan According to the international Health Metrics Network (HMN) Framework and Standards: A Case Study of Hakeem. Ieee Access. 2019;7:51457-65. PMID: WOS:000466704700001. doi: 10.1109/access.2019.2911684.

177. Knox M, Murphy EJ, Leslie T, Wick R, Tuot DS. e-Consult Implementation Success: Lessons From 5 County-Based Delivery Systems. Am J Manag Care. 2020 Jan;26(1):E21-+. PMID: WOS:000518165700005.

178. Kohler S, Keil T, Reinhold T, Muller-Riemenschneider F, Willich SN, Roll S. Usage of a German prevention and health promotion web portal and cost per pageview: A life-cycle assessment. Digit Health. 2019 Sep;5:15. PMID: WOS:000488751700001. doi: 10.1177/2055207619872090.

179. Kose I, Rayner J, Birinci S, Ulgu MM, Yilmaz I, Guner S, et al. Adoption rates of electronic health records in Turkish Hospitals and the relation with hospital sizes. BMC Health Serv Res. 2020 Oct;20(1):16. PMID: WOS:000586418600004. doi: 10.1186/s12913-020-05767-5.

180. Kristensen MBD, Høiberg L, Nøhr C. Updated Mapping of Telemedicine Projects in Denmark. Stud Health Technol Inform. 2019;257:223-8. PMID: 30741200.

181. Krupinski EA, Weinstein RS. Telemedicine in an academic center--the Arizona Telemedicine Program. Telemed J E Health. 2013 May;19(5):349-56. PMID: 23343258. doi: 10.1089/tmj.2012.0285.

182. Kruse GR, Hays H, Orav EJ, Palan M, Sequist TD. Meaningful Use of the Indian Health Service Electronic Health Record. Health Services Research. 2017 Aug;52(4):1349-63. PMID: WOS:000405927400007. doi: 10.1111/1475-6773.12531.

183. Latifi R, Merrell RC, Doarn CR, Hadeed GJ, Bekteshi F, Lecaj I, et al. "Initiate-Build-Operate-Transfer"-A Strategy for Establishing Sustainable Telemedicine Programs in Developing Countries: Initial Lessons from the Balkans. Telemed J E Health. 2009 Dec;15(10):956-69. PMID: WOS:000272995000044. doi: 10.1089/tmj.2009.0084.

184. Leavitt ER, Kessler S, Pun S, Gill T, Escobedo LA, Cockburn M, et al. Teledermatology as a tool to improve access to care for medically underserved populations: A retrospective descriptive study. J Am Acad Dermatol. 2016 Dec;75(6):1259-61. PMID: 27846951. doi: 10.1016/j.jaad.2016.07.043.

185. Lee L, Williams R, Sheikh A. How does joint procurement affect the design, customisation and usability of a hospital ePrescribing system? Health Inform J. 2016 Dec;22(4):828-38. PMID: WOS:000389055600004. doi: 10.1177/1460458215592915.

186. Leite CV, Almeida AM, editors. The Current Status of Online Resources from the Portuguese Health System for Childbirth Education. 2021 16th Iberian Conference on Information Systems and Technologies (CISTI); 2021 23-26 June 2021.

187. Lennon MR, Bouamrane MM, Devlin AM, O'Connor S, O'Donnell C, Chetty U, et al. Readiness for Delivering Digital Health at Scale: Lessons From a Longitudinal Qualitative Evaluation of a National Digital Health Innovation Program in the United Kingdom. J Med Internet Res. 2017 Feb;19(2):18. PMID: WOS:000493380200001. doi: 10.2196/jmir.6900.

188. Leu MG, O'Connor KG, Marshall R, Price DT, Klein JD. Pediatricians' use of health information technology: a national survey. Pediatrics. 2012 Dec;130(6):e1441-6. PMID: 23166335. doi: 10.1542/peds.2012-0396.

189. Lewkowicz D, Wohlbrandt AM, Bottinger E. Digital Therapeutic Care Apps With Decision-Support Interventions for People With Low Back Pain in Germany: Cost-Effectiveness Analysis. JMIR Mhealth Uhealth. 2022 Feb 7;10(2):e35042. PMID: 35129454. doi: 10.2196/35042.

190. Liang J, Li Y, Zhang Z, Shen D, Xu J, Zheng X, et al. Adoption of Electronic Health Records (EHRs) in China During the Past 10 Years: Consecutive Survey Data Analysis and Comparison of Sino-American Challenges and Experiences. J Med Internet Res. 2021 Feb 18;23(2):e24813. PMID: 33599615. doi: 10.2196/24813.

191. Liddy C, Deri Armstrong C, Drosinis P, Mito-Yobo F, Afkham A, Keely E. What are the Costs of Improving Access to Specialists through eConsultation? The Champlain BASE Experience. Stud Health Technol Inform. 2015;209:67-74. PMID: 25980707.

192. Liew CL, Harjadinata J. Patient Portal Service: An Exploration of Patients' Experience and Perception. Berntzen L, GersbeckSchierholz B, editors. Wilmington: Iaria Xps Press; 2017. 1-5 p. ISBN: 978-1-61208-537-1.

193. Lin JC, Humphries MD, Shutze WP, Aalami OO, Fischer UM, Hodgson KJ. Telemedicine platforms and their use in the coronavirus disease-19 era to deliver comprehensive vascular care. J Vasc Surg. 2021 Feb;73(2):392-8. PMID: WOS:000609890400006. doi: 10.1016/j.jvs.2020.06.051.

194. Lin JC, Kavousi Y, Sullivan B, Stevens C. Analysis of Outpatient Telemedicine Reimbursement in an Integrated Healthcare System. Ann Vasc Surg. 2020 May;65:100-6. PMID: 31678131. doi: https://doi.org/10.1016/j.avsg.2019.10.069.

195. Liu PR, Meng MQH, Liu PX, Tong FFL, Chen XJ. A telemedicine system for remote health and activity monitoring for the elderly. Telemed J E Health. 2006 Dec;12(6):622-31. PMID: WOS:000243752700018. doi: 10.1089/tmj.2006.12.622.

196. Liu VX, Haq N, Chan IC, Hoberman B. Inpatient Electronic Health Record Maintenance From 2010 to 2015. Am J Manag Care. 2019 Jan;25(1):18-21. PMID: WOS:000456388800007.

197. Lo YS, Yang CY, Chien HF, Chang SS, Lu CY, Chen RJ. Blockchain-Enabled iWellChain Framework Integration With the National Medical Referral System: Development and Usability Study. J Med Internet Res. 2019 Dec;21(12):13. PMID: WOS:000500983100001. doi: 10.2196/13563.

198. Lobban F, Appelbe D, Appleton V, Aref-Adib G, Barraclough J, Billsborough J, et al. Health Services and Delivery Research. An online supported self-management toolkit for relatives of people with psychosis or bipolar experiences: the IMPART multiple case study. Southampton (UK): NIHR Journals Library

Copyright © Queen’s Printer and Controller of HMSO 2020. This work was produced by Lobban et al. under the terms of a commissioning contract issued by the Secretary of State for Health and Social Care. This issue may be freely reproduced for the purposes of private research and study and extracts (or indeed, the full report) may be included in professional journals provided that suitable acknowledgement is made and the reproduction is not associated with any form of advertising. Applications for commercial reproduction should be addressed to: NIHR Journals Library, National Institute for Health Research, Evaluation, Trials and Studies Coordinating Centre, Alpha House, University of Southampton Science Park, Southampton SO16 7NS, UK.; 2020.

199. Lowery CL, Bronstein JM, Benton TL, Fletcher DA. Distributing Medical Expertise: The Evolution And Impact Of Telemedicine In Arkansas. Health Affairs. 2014 Feb;33(2):235-43. PMID: WOS:000331407800009. doi: 10.1377/hlthaff.2013.1001.

200. Lv Q, Jiang YT, Qi J, Zhang YL, Zhang X, Fang LK, et al. Using Mobile Apps for Health Management: A New Health Care Mode in China. Jmir Mhealth and Uhealth. 2019 Jun;7(6):8. PMID: WOS:000470735900001. doi: 10.2196/10299.

201. Mairesse GH, Braunschweig F, Klersy K, Cowie MR, Leyva F. Implementation and reimbursement of remote monitoring for cardiac implantable electronic devices in Europe: a survey from the health economics committee of the European Heart Rhythm Association. Europace. 2015 May;17(5):814-8. PMID: 25713012. doi: 10.1093/europace/euu390.

202. Mandl KD, Mandel JC, Murphy SN, Bernstam EV, Ramoni RL, Kreda DA, et al. The SMART Platform: early experience enabling substitutable applications for electronic health records. Journal of the American Medical Informatics Association. 2012 Jul;19(4):597-603. PMID: WOS:000306024100019. doi: 10.1136/amiajnl-2011-000622.

203. Marcin JP, Ellis J, Mawis R, Nagrampa E, Nesbitt TS, Dimand RJ. Using telemedicine to provide pediatric subspecialty care to children with special health care needs in an underserved rural community. Pediatrics. 2004 Jan;113(1):1-6. PMID: WOS:000188010600015. doi: 10.1542/peds.113.1.1.

204. Mares ML, Gustafson DH, Glass JE, Quanbeck A, McDowell H, McTavish F, et al. Implementing an mHealth system for substance use disorders in primary care: a mixed methods study of clinicians' initial expectations and first year experiences. Bmc Medical Informatics and Decision Making. 2016 Sep;16:12. PMID: WOS:000384170100001. doi: 10.1186/s12911-016-0365-5.

205. Martin-Khan M, Fatehi F, Kezilas M, Lucas K, Gray LC, Smith AC. Establishing a centralised telehealth service increases telehealth activity at a tertiary hospital. BMC Health Serv Res. 2015 Dec;15:13. PMID: WOS:000365781900001. doi: 10.1186/s12913-015-1180-x.

206. Martinez A, Villarroel V, Seoane J, del Pozo F. EHAS program: Rural telemedicine systems for primary healthcare in developing countries. Herkert JR, editor. New York: Ieee; 2002. 31-6 p. ISBN: 0-7803-7284-0.

207. Martiniuk A, Negin J, Hersch F, Dalipanda T, Jagilli R, Houasia P, et al. Telemedicine in the Solomon Islands: 2006 to 2009. J Telemed Telecare. 2011;17(5):251-6. PMID: 21628420. doi: 10.1258/jtt.2011.100920.

208. Mason P, Mayer R, Chien WW, Monestime JP. Overcoming Barriers to Implementing Electronic Health Records in Rural Primary Care Clinics. Qualitative Report. 2017 Nov;22(11):2943-55. PMID: WOS:000416607200007.

209. Mathai N, McGill T, Toohey D. Factors Influencing Consumer Adoption of Electronic Health Records. Journal of Computer Information Systems. 2020:11. PMID: WOS:000573970700001. doi: 10.1080/08874417.2020.1802788.

210. McCullough JM, Zimmerman FJ, Bell DS, Rodriguez HP. Local public health department adoption and use of electronic health records. J Public Health Manag Pract. 2015 Jan-Feb;21(1):E20-8. PMID: 25271385. doi: 10.1097/phh.0000000000000143.

211. McEachern A, Cholewa D. Digital Health Services and Digital Identity in Alberta. In: Lau F, BartleClar J, Bliss G, Borycki E, Courtney K, Kuo A, editors. Building Capacity for Health Informatics in the Future. Amsterdam: Ios Press; 2017. p. 222-227.

212. McHugh M, Shi YF, McClellan SR, Shortell SM, Fareed N, Harvey J, et al. Using multi-stakeholder alliances to accelerate the adoption of health information technology by physician practices. HealthCare. 2016 Jun;4(2):86-91. PMID: WOS:000379247000004. doi: 10.1016/j.hjdsi.2016.01.004.

213. Melchiorre MG, Papa R, Quattrini S, Lamura G, Barbabella F, Rijken M, et al. Integrated Care Programs for People with Multimorbidity in European Countries: eHealth Adoption in Health Systems. Biomed Res Int. 2020 Apr;2020:23. PMID: WOS:000529175700001. doi: 10.1155/2020/9025326.

214. Melvin CL, Saef SH, Pierce HO, Obeid JS, Carr CM. Health Information Exchange in the ED: What Do ED Clinicians Think? South Med J. 2016 Jul;109(7):419-26. PMID: 27364028. doi: 10.14423/smj.0000000000000466.

215. Menachemi N, Matthews MC, Ford EW, Brooks RG. The influence of payer mix on electronic health record adoption by physicians. Health Care Manage Rev. 2007 Apr-Jun;32(2):111-8. PMID: WOS:000246026200004. doi: 10.1097/01.HMR.0000267791.02062.3f.

216. Mendelson D, Wolf G. “My [Electronic] Health Record” – Cui Bono (For Whose Benefit)? J Law Med. 2016;24(2):283-96. PMID: 30137703.

217. Merrill JA, Deegan M, Wilson RV, Kaushal R, Fredericks K. A system dynamics evaluation model: implementation of health information exchange for public health reporting. J Am Med Inform Assoc. 2013 Jun;20(e1):e131-8. PMID: 23292910. doi: 10.1136/amiajnl-2012-001289.

218. Mierdel S, Owen K. Telehomecare Reduces ER Use and Hospitalizations at William Osler Health System. In: Gillis G, Newsham D, Maeder AJ, editors. Global Telehealth 2015: Integrating Technology and Information for Better Healthcare. Amsterdam: Ios Press; 2015. p. 102-8.

219. Miklin DJ, Vangara SS, Delamater AM, Goodman KW. Understanding of and Barriers to Electronic Health Record Patient Portal Access in a Culturally Diverse Pediatric Population. JMIR Med Inf. 2019 Apr-Jun;7(2):127-35. PMID: WOS:000473777800011. doi: 10.2196/11570.

220. Milberg JA. Development, use, and integration of a nationally-distributed HIV/AIDS electronic health information system. Journal of the American Medical Informatics Association. 2016 Nov;23(6):1190-4. PMID: WOS:000388006800022. doi: 10.1093/jamia/ocv212.

221. Miley ML, Demaerschalk BM, Olmstead NL, Kiernan TEJ, Corday DA, Chikani V, et al. The State of Emergency Stroke Resources and Care in Rural Arizona: A Platform for Telemedicine. Telemed J E Health. 2009 Sep;15(7):691-9. PMID: WOS:000269738800048. doi: 10.1089/tmj.2009.0018.

222. Ming LC, Untong N, Aliudin NA, Osili N, Kifli N, Tan CS, et al. Mobile Health Apps on COVID-19 Launched in the Early Days of the Pandemic: Content Analysis and Review. JMIR Mhealth Uhealth. 2020 Sep 16;8(9):e19796. PMID: 32609622. doi: 10.2196/19796.

223. Miranda ML, Ferranti J, Strauss B, Neelon B, Califf RM. Geographic Health Information Systems: A Platform To Support The 'Triple Aim'. Health Affairs. 2013 Sep;32(9):1608-15. PMID: WOS:000324681500014. doi: 10.1377/hlthaff.2012.1199.

224. Mooranian A, Emmerton L, Hattingh L. The introduction of the national e-health record into Australian community pharmacy practice: pharmacists' perceptions. Int J Pharm Pract. 2013 Dec;21(6):405-12. PMID: 23560554. doi: 10.1111/ijpp.12034.

225. Morishita M, Takahashi O, Yoshii S, Hayashi M, Kibune R, Nakamura T, et al. Effect of COVID-19 on dental telemedicine in Japan. J Dent Sci. 2022 Jan;17(1):42-8. PMID: 34457200. doi: 10.1016/j.jds.2021.07.028.

226. Muhammad I, Teoh SY, Wickramasinghe N. The Need for a Socio-Technical Analysis in E-Health: The Case of the PCEHR. International Journal of E-Health and Medical Communications. 2013;4(2):65-79. PMID: WOS:000438709900005. doi: 10.4018/jehmc.2013040105.

227. Muinga N, Magare S, Monda J, Kamau O, Houston S, Fraser H, et al. Implementing an Open Source Electronic Health Record System in Kenyan Health Care Facilities: Case Study. JMIR Med Inf. 2018 Apr-Jun;6(2):215-27. PMID: WOS:000438272800018. doi: 10.2196/medinform.8403.

228. Mwachofi AK, Khaliq AA, Carrillo ER, Winfree W. Technology versus humanism: how patients perceive the use of electronic health records in physicians' offices-a qualitative study. Health Commun. 2016 Mar;31(3):257-64. PMID: WOS:000364556000001. doi: 10.1080/10410236.2014.947467.

229. Namageyo-Funa A, Aketch M, Tabu C, MacNeil A, Bloland P. Assessment of select electronic health information systems that support immunization data capture - Kenya, 2017. BMC Health Serv Res. 2018 Aug;18:7. PMID: WOS:000441357800004. doi: 10.1186/s12913-018-3435-9.

230. Naverlo S, Carson DB, Edin-Liljegren A, Ekstedt M. Patient perceptions of a Virtual Health Room installation in rural Sweden. Rural Remote Health. 2016 Oct-Dec;16(4):8. PMID: WOS:000396697000007.

231. Ndlovu K, Mauco KL, Keetile M, Kadimo K, Senyatso RY, Ntebela D, et al. Acceptance of the District Health Information System Version 2 Platform for Malaria Case-Based Surveillance By Health Care Workers in Botswana: Web-Based Survey. JMIR Form Res. 2022 Mar 15;6(3):e32722. PMID: 35289760. doi: 10.2196/32722.

232. Nesbitt TS, Cole SL, Pellegrino L, Keast P. Rural outreach in home telehealth: Assessing challenges and reviewing successes. Telemed J E Health. 2006 Apr;12(2):107-13. PMID: WOS:000237647800020. doi: 10.1089/tmj.2006.12.107.

233. Nesbitt TS, Hilty DM, Kuenneth CA, Siefkin A. Development of a telemedicine program. West J Med. 2000 Sep;173(3):169-74. PMID: WOS:000089026400015. doi: 10.1136/ewjm.173.3.169-a.

234. Neuner J, Fedders M, Caravella M, Bradford L, Schapira M. Meaningful Use and the Patient Portal: Patient Enrollment, Use, and Satisfaction With Patient Portals at a Later-Adopting Center. Am J Med Qual. 2015 Mar-Apr;30(2):105-13. PMID: WOS:000350467800001. doi: 10.1177/1062860614523488.

235. Ng SW, Hwong WY, Husin M, Ab Rahman N, Nasir NH, Juval K, et al. Assessing the Availability of Teleconsultation and the Extent of Its Use in Malaysian Public Primary Care Clinics: Cross-sectional Study. JMIR Form Res. 2022 May 9;6(5):e34485. PMID: 35532973. doi: 10.2196/34485.

236. Nielssen O, Dear BF, Staples LG, Dear R, Ryan K, Purtell C, et al. Procedures for risk management and a review of crisis referrals from the MindSpot Clinic, a national service for the remote assessment and treatment of anxiety and depression. BMC Psychiatry. 2015 Dec 1;15:304. PMID: 26626712. doi: 10.1186/s12888-015-0676-6.

237. Nyangena J, Rajgopal R, Ombech EA, Oloo E, Luchetu H, Wambugu S, et al. Maturity assessment of Kenya's health information system interoperability readiness. BMJ Health Care Inform. 2021 Jun;28(1). PMID: 34210718. doi: 10.1136/bmjhci-2020-100241.

238. O'Gorman LD, Hogenbirk JC, Warry W. Clinical Telemedicine Utilization in Ontario over the Ontario Telemedicine Network. Telemed e-Health. 2016 Jun;22(6):473-9. PMID: WOS:000377383600003. doi: 10.1089/tmj.2015.0166.

239. O'Hara R, Jackson S. Integrating telehealth services into a remote allied health service: A pilot study. Aust J Rural Health. 2017 Feb;25(1):53-7. PMID: 25823551. doi: 10.1111/ajr.12189.

240. O'Malley AS, Draper K, Gourevitch R, Cross DA, Scholle SH. Electronic health records and support for primary care teamwork. Journal of the American Medical Informatics Association. 2015 Mar;22(2):426-34. PMID: WOS:000352771500020. doi: 10.1093/jamia/ocu029.

241. Paavola T, Makela K, Pyykko V, Perala S. A national Finnish e-health development project 'ProViisikko'. J Telemed Telecare. 2006;12:67-9. PMID: WOS:000243423100022. doi: 10.1258/135763306779379987.

242. Painter J, Turner J, Procter PM. Understanding and Accommodating Patient and Staff Choice When Implementing Video Consultations in Mental Health Services. Comput Inform Nurs. 2021 Jul 12;39(10):578-83. PMID: 34238832. doi: 10.1097/cin.0000000000000804.

243. Pangka KR, Chandrasena R, Wijeratne N, Mann M. Exploring the views of emergency department staff on the use of videoconferencing for mental health emergencies in southwestern Ontario. Stud Health Technol Inform. 2015;209:114-20. PMID: 25980713.

244. Papaioannou M, Neocleous A, Savva P, Miguel F, Panayides A, Antoniou Z, et al., editors. A Prototype of the National EHR system for Cyprus. 2021 43rd Annual International Conference of the IEEE Engineering in Medicine & Biology Society (EMBC); 2021 1-5 Nov. 2021.

245. Park SY, Chen YN, Rudkin S. Technological and Organizational Adaptation of EMR Implementation in an Emergency Department. Acm Transactions on Computer-Human Interaction. 2015 Mar;22(1):24. PMID: WOS:000351224400001. doi: 10.1145/2656213.

246. Pearce C, Bartlett J, McLeod A, Eustace P, Amos R, Shearer M. Effectiveness of local support for the adoption of a national programme--a descriptive study. Inform Prim Care. 2014;21(4):171-8. PMID: 25479347. doi: 10.14236/jhi.v21i4.70.

247. Pearce C, McLeod A, Supple J, Gardner K, Proposch A, Ferrigi J. Responding to COVID-19 with real-time general practice data in Australia. International Journal of Medical Informatics. 2022 Jan;157:6. PMID: WOS:000718292900004. doi: 10.1016/j.ijmedinf.2021.104624.

248. Pfeiffer KP, Auer CM. Challenges in the implementation of electronic health care records and patient cards in Austria. Bundesgesundheitsblatt-Gesund. 2009 Mar;52(3):324-9. PMID: WOS:000265498000009. doi: 10.1007/s00103-009-0791-y.

249. Pharow P, Blobel B, Hildebrand C. How Can the German Electronic Health Card Support Patient's Role in Care Management. In: Bos L, Blobel B, Marsh A, Carroll D, editors. Medical and Care Compunetics 5. Amsterdam: I O S Press; 2008. p. 386-401.

250. Popova I, Asrafi S. Adoption of e-health in private and public hospitals: A case study of Bangladesh. Macedo M, Gauzente C, Nunes MB, Peng GC, editors. Lisboa: Iadis-Int Assoc Development Information Society; 2015. 27-34 p. ISBN: 978-989-8533-42-5.

251. Rajamani S, Kayser A, Ruprecht A, Cassman J, Polzer M, Homan T, et al. Electronic Case Reporting (eCR) of COVID-19 to Public Health: Implementation Perspectives from the Minnesota Department of Health. J Am Med Inform Assoc. 2022 Jul 29. PMID: 35904765. doi: 10.1093/jamia/ocac133.

252. Razavi H, Copeland SP, Turner AW. Increasing the impact of teleophthalmology in Australia: Analysis of structural and economic drivers in a state service. Aust J Rural Health. 2017 Feb;25(1):45-52. PMID: WOS:000394998500008. doi: 10.1111/ajr.12277.

253. Reed M, Huang J, Brand R, Graetz I, Neugebauer R, Fireman B, et al. Implementation of an outpatient electronic health record and emergency department visits, hospitalizations, and office visits among patients with diabetes. JAMA. 2013 Sep 11;310(10):1060-5. PMID: 24026601. doi: 10.1001/jama.2013.276733.

254. Reitz R, Common K, Fifield P, Stiasny E. Collaboration in the presence of an electronic health record. Fam Syst Health. 2012 Mar;30(1):72-80. PMID: 22429079. doi: 10.1037/a0027016.

255. Reynolds E, Martel LD, Bah MO, Bah M, Bah MB, Boubacar B, et al. Implementation of DHIS2 for Disease Surveillance in Guinea: 2015-2020. Front Public Health. 2021;9:761196. PMID: 35127614. doi: 10.3389/fpubh.2021.761196.

256. Richardson JE, Abramson EL, Pfoh ER, Kaushal R. How communities are leveraging the health information technology workforce to implement electronic health records. AMIA Annu Symp Proc. 2011;2011:1186-95. PMID: 22195179.

257. Rickwood D, Webb M, Kennedy V, Telford N. Who Are the Young People Choosing Web-based Mental Health Support? Findings From the Implementation of Australia's National Web-based Youth Mental Health Service, eheadspace. JMIR Ment Health. 2016 Jul-Sep;3(3):11. PMID: WOS:000414980700011. doi: 10.2196/mental.5988.

258. Rodrigues DLG, Belber GS, Borysow IDC, Maeyama MA, Pinho A. Description of e-Health Initiatives to Reduce Chronic Non-Communicable Disease Burden on Brazilian Health System. Int J Environ Res Public Health. 2021 Sep 28;18(19). PMID: 34639518. doi: 10.3390/ijerph181910218.

259. Ronis SD, McConnochie KM, Wang HY, Wood NE. Urban Telemedicine Enables Equity in Access to Acute Illness Care. Telemed e-Health. 2017 Feb;23(2):105-12. PMID: WOS:000394355200006. doi: 10.1089/tmj.2016.0098.

260. Rosati S, Zema M, Castagneri C, Marchetti F, Balestra G. Modelling and Analysis of Four Telemedicine Italian Experiences. 2017 39th Annual International Conference of the Ieee Engineering in Medicine and Biology Society. New York: Ieee; 2017. p. 2634-7.

261. Ross J, Stevenson F, Dack C, Pal K, May C, Michie S, et al. Developing an implementation strategy for a digital health intervention: an example in routine healthcare. BMC Health Serv Res. 2018 Oct;18:13. PMID: WOS:000450985900009. doi: 10.1186/s12913-018-3615-7.

262. Ross JAD, Barron E, McGough B, Valabhji J, Daff K, Irwin J, et al. Uptake and impact of the English National Health Service digital diabetes prevention programme: observational study. BMJ Open Diabetes Res Care. 2022 May;10(3). PMID: 35504697. doi: 10.1136/bmjdrc-2021-002736.

263. Salleh MIM, Abdullah R, Zakaria N. Evaluating the effects of electronic health records system adoption on the performance of Malaysian health care providers. BMC Med Inform Decis Mak. 2021 Feb 25;21(1):75. PMID: 33632216. doi: 10.1186/s12911-021-01447-4.

264. Scandurra I, Jansson A, Forsberg-Fransson ML, Alander T. Is 'patient's online access to health records' a good reform? - Opinions from Swedish healthcare professionals differ. In: CruzCunha MM, Varajao J, Rijo R, Martinho R, Schubert P, Boonstra A, et al., editors. Conference on Enterprise Information Systems/International Conference on Project Management/Conference on Health and Social Care Information Systems and Technologies, Centeris/Projman / Hcist 2015. Amsterdam: Elsevier Science Bv; 2015. p. 964-8.

265. Scott BK, Miller GT, Fonda SJ, Yeaw RE, Gaudaen JC, Pavliscsak HH, et al. Advanced Digital Health Technologies for COVID-19 and Future Emergencies. Telemed e-Health. 2020 Oct;26(10):1226-33. PMID: WOS:000536272300001. doi: 10.1089/tmj.2020.0140.

266. Secor AM, Mtenga H, Richard J, Bulula N, Ferriss E, Rathod M, et al. Added Value of Electronic Immunization Registries in Low- and Middle-Income Countries: Observational Case Study in Tanzania. JMIR Public Health Surveill. 2022 Jan;8(1):24. PMID: WOS:000749005400001. doi: 10.2196/32455.

267. Sequist TD, Cullen T, Hays H, Taualii MM, Simon SR, Bates DW. Implementation and use of an electronic health record within the Indian health service. Journal of the American Medical Informatics Association. 2007 Mar-Apr;14(2):191-7. PMID: WOS:000245562600007. doi: 10.1197/jamia.M2234.

268. Séroussi B, Bouaud J. Update on the DMP, the French Nationally Shared Medical Record: Did We Make It? Stud Health Technol Inform. 2020 Jun 16;270:698-702. PMID: 32570473. doi: 10.3233/shti200250.

269. Setiawan AW, Handayani A, Setiawan AD, Saptawati GAP, Suksmono AB, Mengko TR. A Review of the OpenEHR Implementation in Indonesian National Health Information System: Integrated Health Post. In: Dossel O, Schlegel WC, editors. World Congress on Medical Physics and Biomedical Engineering, Vol 25, Pt 5. New York: Springer; 2009. p. 234-6.

270. Sezgin E, Alasehir O, Yildirim SO. Work in Progress toward Adoption of an e-Health Application by Healthcare Personnel: A Model Validation. In: Varajao J, Cunha M, BjornAndersen N, Turner R, Wijesekera D, Martinho R, et al., editors. Centeris 2014 - Conference on Enterprise Information Systems / Projman 2014 - International Conference on Project Management / Hcist 2014 - International Conference on Health and Social Care Information Systems and Technologies. Amsterdam: Elsevier Science Bv; 2014. p. 1327-33.

271. Shah GH, Leider JP, Castrucci BC, Williams KS, Luo HB. Characteristics of Local Health Departments Associated with Implementation of Electronic Health Records and Other Informatics Systems. Public Health Rep. 2016 Mar-Apr;131(2):272-82. PMID: WOS:000372392900010. doi: 10.1177/003335491613100211.

272. Sharma N, O'Hare K, O'Connor KG, Nehal U, Okumura MJ. Care Coordination and Comprehensive Electronic Health Records are Associated With Increased Transition Planning Activities. Acad Pediatr. 2018 Jan-Feb;18(1):111-8. PMID: WOS:000419810600016.

273. Sheikh A, Cornford T, Barber N, Avery A, Takian A, Lichtner V, et al. Implementation and adoption of nationwide electronic health records in secondary care in England: final qualitative results from prospective national evaluation in "early adopter" hospitals. Bmj-British Medical Journal. 2011 Oct;343:14. PMID: WOS:000297245400002. doi: 10.1136/bmj.d6054.

274. Shi YF, Amill-Rosario A, Rudin RS, Fischer SH, Shekelle P, Scanlon D, et al. Health Information Technology for Ambulatory Care in Health Systems. Am J Manag Care. 2020 Jan;26(1):32-+. PMID: WOS:000518165700011.

275. Showell CM. Citizens, patients and policy: a challenge for Australia's national electronic health record. Health Information Management Journal. 2011;40(2):39-43. PMID: WOS:000292621500006. doi: 10.1177/183335831104000206.

276. Shur N, Atabaki SM, Kisling MS, Tabarani A, Williams C, Fraser JL, et al. Rapid deployment of a telemedicine care model for genetics and metabolism during COVID-19. Am J Med Genet A. 2021 Jan;185(1):68-72. PMID: 33051968. doi: 10.1002/ajmg.a.61911.

277. Silva GS, Farrell S, Shandra E, Viswanathan A, Schwamm LH. The status of telestroke in the United States: a survey of currently active stroke telemedicine programs. Stroke. 2012 Aug;43(8):2078-85. PMID: 22700532. doi: 10.1161/strokeaha.111.645861.

278. Silva RSD, Schmtiz CAA, Harzheim E, Molina-Bastos CG, Oliveira EB, Roman R, et al. The Role of Telehealth in the Covid-19 Pandemic: A Brazilian Experience. Cien Saude Colet. 2021 Jun;26(6):2149-57. PMID: 34231727. doi: 10.1590/1413-81232021266.39662020.

279. Sittig DF, Singh H. Legal, Ethical, and Financial Dilemmas in Electronic Health Record Adoption and Use. Pediatrics. 2011 Apr;127(4):E1042-E7. PMID: WOS:000289074800025. doi: 10.1542/peds.2010-2184.

280. Skrceska I, Velinov G, Kon-Popovska M, Neskovska M, Pevac M, Stoimenov L, et al. Analysis of the Preconditions for Implementation of Nationwide EHR Systems. In: Damasevicius R, Mikasyte V, editors. Information and Software Technologies. Berlin: Springer-Verlag Berlin; 2017. p. 177-89.

281. Slight SP, Quinn C, Avery AJ, Bates DW, Sheikh A. A qualitative study identifying the cost categories associated with electronic health record implementation in the UK. J Am Med Inform Assoc. 2014 Oct;21(e2):e226-31. PMID: 24523391. doi: 10.1136/amiajnl-2013-002404.

282. Slotwiner D, Wilkoff B. Cost efficiency and reimbursement of remote monitoring: a US perspective. Europace. 2013 Jun;15:54-8. PMID: WOS:000320120500013. doi: 10.1093/europace/eut109.

283. Snoswell CL, Caffery LJ, Haydon HM, Thomas EE, Smith AC. Telehealth uptake in general practice as a result of the coronavirus (COVID-19) pandemic. Aust Health Rev. 2020;44(5):737-40. PMID: WOS:000563094700001. doi: 10.1071/ah20183.

284. Sørensen T, Dyb K, Rygh E, Salvesen R, Thomassen L. A qualitative description of telemedicine for acute stroke care in Norway: technology is not the issue. BMC Health Serv Res. 2014 Dec 19;14:643. PMID: 25523241. doi: 10.1186/s12913-014-0643-9.

285. Souliotis K, Mantzana V, Papageorgiou M. Transforming Public Servants' Health Care Organization in Greece through the Implementation of an Electronic Referral Project. Value Health Reg Issues. 2013 Sep-Oct;2(2):312-8. PMID: 29702883. doi: 10.1016/j.vhri.2013.06.003.

286. Speedie SM, Park YT, Du J, Theera-Ampornpunt N, Bershow BA, Gensinger RA, Jr., et al. The impact of electronic health records on people with diabetes in three different emergency departments. J Am Med Inform Assoc. 2014 Feb;21(e1):e71-7. PMID: 23842938. doi: 10.1136/amiajnl-2013-001804.

287. Spil TAM, Katsma CP, Stegwee RA, Albers EF, Freriks A, Ligt E. Value, Participation and Quality of Electronic Health Records in the Netherlands. 43rd Hawaii International Conference on Systems Sciences Vols 1-5. Los Alamitos: Ieee Computer Soc; 2010. p. 2443-52.

288. Srivastava S, Agarwal N, Pane M, Abraham A. A Secured Model for Indian E-Health System. 2013 9th International Conference on Information Assurance and Security. New York: Ieee; 2013. p. 96-+.

289. Ssembatya R, Kayem A, Marsden G. On the challenge of adopting standard EHR systems in developing countries. Proceedings of the 3rd ACM Symposium on Computing for Development; Bangalore, India: Association for Computing Machinery; 2013. p. Article 23.

290. Stroetmann KA. Scoping Global Good eHealth Platforms: Implications for Sub-Saharan Africa. New York: Ieee; 2014. ISBN: 978-1-905824-44-1.

291. Sun C, Sun L, Xi S, Zhang H, Wang H, Feng Y, et al. Mobile Phone-Based Telemedicine Practice in Older Chinese Patients with Type 2 Diabetes Mellitus: Randomized Controlled Trial. JMIR Mhealth Uhealth. 2019 Jan 4;7(1):e10664. PMID: 30609983. doi: 10.2196/10664.

292. Swartz A, LeFevre AE, Perera S, Kinney MV, George AS. Multiple pathways to scaling up and sustainability: an exploration of digital health solutions in South Africa. Global Health. 2021 Jul;17(1):13. PMID: WOS:000670257200001. doi: 10.1186/s12992-021-00716-1.

293. Tavakoli N, Jahanbakhsh M, Mokhtari H, Tadayon HR. Opportunities of Electronic Health Record Implementation in Isfahan. In: Karahoca A, Kanbul S, editors. World Conference on Information Technology. Amsterdam: Elsevier Science Bv; 2011.

294. Thakkar M, Davis DC. Risks, barriers, and benefits of EHR systems: a comparative study based on size of hospital. Perspect Health Inf Manag. 2006 Aug 14;3:5. PMID: 18066363.

295. Thorne T, Smith M, Dever G. The Current Status of Telehealth and Distance Learning in Palau. Hawaii J Health Soc Welf. 2022 Apr;81(4):87-93. PMID: 35415614.

296. Tubaishat A, Al-Rawajfah OM. The Use of Electronic Medical Records in Jordanian Hospitals A Nationwide Survey. CIN-Comput Inform Nurs. 2017 Oct;35(10):538-45. PMID: WOS:000412432900007. doi: 10.1097/cin.0000000000000343.

297. Turakhia MP, Kaiser DW. Transforming the care of atrial fibrillation with mobile health. J Interv Card Electrophysiol. 2016 Oct;47(1):45-50. PMID: WOS:000387110400007. doi: 10.1007/s10840-016-0136-3.

298. Ummer O, Scott K, Mohan D, Chakraborty A, LeFevre AE. Connecting the dots: Kerala's use of digital technology during the COVID-19 response. BMJ Glob Health. 2021 Jul;6(Suppl 5). PMID: 34312152. doi: 10.1136/bmjgh-2021-005355.

299. Vest JR, Kern LM, Silver MD, Kaushal R, Investigators H. The potential for community-based health information exchange systems to reduce hospital readmissions. Journal of the American Medical Informatics Association. 2015 Mar;22(2):435-42. PMID: WOS:000352771500021. doi: 10.1136/amiajnl-2014-002760.

300. Visser M, Kotze M, van Rensburg MJ. An mHealth HIV prevention programme for youth: lessons learned from the iloveLife.mobi programme in South Africa. Aids Care-Psychological and Socio-Medical Aspects of Aids/Hiv. 2020 May;32:S148-S54. PMID: WOS:000524224300001. doi: 10.1080/09540121.2020.1742866.

301. Visweswaran S, McLay B, Cappella N, Morris M, Milnes JT, Reis SE, et al. An atomic approach to the design and implementation of a research data warehouse. J Am Med Inform Assoc. 2022 Mar 15;29(4):601-8. PMID: 34613409. doi: 10.1093/jamia/ocab204.

302. Wang CS, Liu CW, Wang TW. The Implementation of Remotely and Real-timely Monitoring System from Web End for Long-term Health Care Institute. In: Luo Q, Zeng D, editors. Information Technology for Manufacturing Systems Ii, Pts 1-3. Durnten-Zurich: Trans Tech Publications Ltd; 2011. p. 2499-+.

303. Ward MM, Ullrich F, MacKinney AC, Bell AL, Shipp S, Mueller KJ. Tele-emergency utilization: In what clinical situations is tele-emergency activated? J Telemed Telecare. 2016 Jan;22(1):25-31. PMID: 26026189. doi: 10.1177/1357633x15586319.

304. Weinerman BH, Barnett J, Loyola M, den Duyf J, Robertson S, Ashworth V, et al. Telehealth--a change in a practice model in oncology. Telemed J E Health. 2012 Jun;18(5):391-3. PMID: 22489930. doi: 10.1089/tmj.2011.0183.

305. Weiss JB, Grant A, Marelli A, Khairy P, Maurais T, Rehel S, et al. Assessment of Electronic Health Information System Use and Need in US Adult Congenital Heart Disease Centers. Congenit Heart Dis. 2011 Mar-Apr;6(2):134-8. PMID: WOS:000289425500007. doi: 10.1111/j.1747-0803.2011.00498.x.

306. Welch WP, Bazarko D, Ritten K, Burgess Y, Harmon R, Sandy LG. Electronic health records in four community physician practices: impact on quality and cost of care. J Am Med Inform Assoc. 2007 May-Jun;14(3):320-8. PMID: 17329734. doi: 10.1197/jamia.M2125.

307. Whitacre BE. Rural EMR adoption rates overtake those in urban areas. Journal of the American Medical Informatics Association. 2015 Mar;22(2):399-408. PMID: WOS:000352771500017. doi: 10.1093/jamia/ocu035.

308. Whittaker SL, Adkins S, Phillips R, Jones J, Horsley MA, Kelley G. Success factors in the long-term sustainability of a telediabetes programme. J Telemed Telecare. 2004;10(2):84-8. PMID: WOS:000220737700004. doi: 10.1258/135763304773391512.

309. Whitten P, Buis L. Private payer reimbursement for telemedicine services in the United States. Telemed J E Health. 2007 Feb;13(1):15-23. PMID: WOS:000245031000017. doi: 10.1089/tmj.2006.0028.

310. Widmer RJ, Allison TG, Lennon R, Lopez-Jimenez F, Lerman LO, Lerman A. Digital health intervention during cardiac rehabilitation: A randomized controlled trial. Am Heart J. 2017 Jun;188:65-72. PMID: WOS:000403197800008. doi: 10.1016/j.ahj.2017.02.016.

311. Wilkes MS, Marcin JP, Ritter LA, Pruitt S. Organizational and teamwork factors of tele-intensive care units. Am J Crit Care. 2016 Sep;25(5):431-9. PMID: WOS:000389409600010. doi: 10.4037/ajcc2016357.

312. Wilson A, Moretto N, Langbecker D, Snoswell CL. Use of Reimbursed Psychology Videoconference Services in Australia: An Investigation Using Administrative Data. Value Health Reg Issues. 2020 May;21:69-73. PMID: 31655466. doi: 10.1016/j.vhri.2019.07.007.

313. Winkie MJ, Nambudiri VE. A tale of two applications: lessons learned from national LMIC COVID applications. Journal of the American Medical Informatics Association.6. PMID: WOS:000847392800001. doi: 10.1093/jamia/ocac146.

314. Wittie M, Ngo-Metzger Q, Lebrun-Harris L, Shi LY, Nair S. Enabling Quality: Electronic Health Record Adoption and Meaningful Use Readiness in Federally Funded Health Centers. J Healthc Qual. 2016 Jan-Feb;38(1):42-51. PMID: WOS:000372788700007.

315. Wylie MC, Baier RR, Gardner RL. Perceptions of Electronic Health Record Implementation: A Statewide Survey of Physicians in Rhode Island. Am J Med. 2014 Oct;127(10):7. PMID: WOS:000343249200037. doi: 10.1016/j.amjmed.2014.06.011.

316. Xia ZN, Gao WJ, Wei XJ, Peng YC, Ran HJ, Wu H, et al. Perceived Value of Electronic Medical Records in Community Health Services: A National Cross-Sectional Survey of Primary Care Workers in Mainland China. International Journal of Environmental Research and Public Health. 2020 Nov;17(22):19. PMID: WOS:000594131300001. doi: 10.3390/ijerph17228510.

317. Xie X, Zhou WM, Lin LY, Fan S, Lin F, Wang L, et al. Internet Hospitals in China: Cross-Sectional Survey. J Med Internet Res. 2017 Jul;19(7):8. PMID: WOS:000404930100005. doi: 10.2196/jmir.7854.

318. Xierali IM, Hsiao CJ, Puffer JC, Green LA, Rinaldo JC, Bazemore AW, et al. The rise of electronic health record adoption among family physicians. Ann Fam Med. 2013 Jan-Feb;11(1):14-9. PMID: 23319501. doi: 10.1370/afm.1461.

319. Xierali IM, Phillips RL, Jr., Green LA, Bazemore AW, Puffer JC. Factors influencing family physician adoption of electronic health records (EHRs). J Am Board Fam Med. 2013 Jul-Aug;26(4):388-93. PMID: 23833153. doi: 10.3122/jabfm.2013.04.120351.

320. Yang JY, Wu YW, Chuang W, Lin TC, Chang SW, Cheng SH, et al. An Integrated Community-Based Blood Pressure Telemonitoring Program - A Population-Based Observational Study. Acta Cardiol Sin. 2022 Sep;38(5):612-22. PMID: 36176366. doi: 10.6515/acs.202209_38(5).20220330a.

321. Yim KM, Florek AG, Oh DH, McKoy K, Armstrong AW. Teledermatology in the United States: An Update in a Dynamic Era. Telemed J E Health. 2018 Sep;24(9):691-7. PMID: 29356616. doi: 10.1089/tmj.2017.0253.

322. Yousef CC, Thomas A, Alenazi AO, Elgadi S, Abu Esba LC, AlAzmi A, et al. Adoption of a Personal Health Record in the Digital Age: Cross-Sectional Study. J Med Internet Res. 2020 Oct 28;22(10):e22913. PMID: 32998854. doi: 10.2196/22913.

323. Yu J, Mink PJ, Huckfeldt PJ, Gildemeister S, Abraham JM. Population-Level Estimates Of Telemedicine Service Provision Using An All-Payer Claims Database. Health Aff (Millwood). 2018 Dec;37(12):1931-9. PMID: 30633676. doi: 10.1377/hlthaff.2018.05116.

324. Zakus D, Moussa M, Ezechiel M, Yimbesalu JP, Orkar P, Damecour C, et al. Clinical evaluation of the use of an mhealth intervention on quality of care provided by Community Health Workers in southwest Niger. J Glob Health. 2019 Jun;9(1):010812. PMID: 31263555. doi: 10.7189/jogh.09.010812.

325. Zaman TU, Raheem TMA, Alharbi GM, Shodri MF, Kutbi AH, Alotaibi SM, et al. E-health and its Transformation of Healthcare Delivery System in Makkah, Saudi Arabia. International Journal of Medical Research & Health Sciences. 2018;7(5):76-82. PMID: WOS:000436123400001.

326. Zanaboni P, Wootton R. Adoption of routine telemedicine in Norwegian hospitals: progress over 5 years. BMC Health Serv Res. 2016 Sep 20;16:496. PMID: 27644324. doi: 10.1186/s12913-016-1743-5.

327. Zapata BC, Hernández Niñirola A, Fernández-Alemán JL, Toval A. Assessing the privacy policies in mobile personal health records. Annu Int Conf IEEE Eng Med Biol Soc. 2014;2014:4956-9. PMID: 25571104. doi: 10.1109/embc.2014.6944736.

328. Zelmer J, Ronchi E, Hypponen H, Lupianez-Villanueva F, Codagnone C, Nohr C, et al. International health IT benchmarking: learning from cross-country comparisons. Journal of the American Medical Informatics Association. 2017 Mar;24(2):371-9. PMID: WOS:000397029100019.

329. Zhang D, Wang G, Zhu W, Thapa JR, Switzer JA, Hess DC, et al. Expansion Of Telestroke Services Improves Quality Of Care Provided In Super Rural Areas. Health Aff (Millwood). 2018 Dec;37(12):2005-13. PMID: 30633675. doi: 10.1377/hlthaff.2018.05089.

330. Zhou YY, Leith WM, Li H, Tom JO. Personal health record use for children and health care utilization: propensity score-matched cohort analysis. J Am Med Inform Assoc. 2015 Jul;22(4):748-54. PMID: 25656517. doi: 10.1093/jamia/ocu018.

Publications with a wrong study design or publication type

1. Afridi MI. Tele-mental and behavioural health: implications in glocal context - a hope to meet the needs of the underserved. J Pak Med Assoc. 2013 Jun;63(6):668-9. PMID: 23901661.

2. Asch DA. The hidden economics of telemedicine. Ann Intern Med. 2015 Nov 17;163(10):801-2. PMID: 26343261. doi: 10.7326/m15-1416.

3. Bashshur R. Telemedicine and state-based licensure in the United States, revisited. Telemed J E Health. 2008 May;14(4):310-1. PMID: 18570558. doi: 10.1089/tmj.2008.9977.

4. Doarn CR, Merrell RC. A national strategy for telemedicine and e-health. Telemed J E Health. 2007 Jun;13(3):243-4. PMID: 17603825. doi: 10.1089/tmj.2007.9982.

5. Fazzi R, Ashe T, Doak L. Telehealth and technology in home care: part I. Insights from the Philips National Study on the Future of Technology and Telehealth in Home Care. Caring. 2007 Oct;26(10):40-2, 4-7. PMID: 17988003.

6. Gerkin DG. Telemedicine and e-health. Tenn Med. 2008 Jul;101(7):6-7, 10. PMID: 18649619.

7. Goozner M. National action needed to advance telemedicine. Mod Healthc. 2015 Jan 19;45(3):26. PMID: 25671904.

8. Gordon CR. The mobile technology era: HITECH and expectations for the future. Plast Reconstr Surg. 2013 Aug;132(2):319e. PMID: 23897367. doi: 10.1097/PRS.0b013e3182958eaf.

9. Halpern NA. From telemedicine to a critical care database: a new resource for national benchmarking. Chest. 2011 Nov;140(5):1111-3. PMID: 22045874. doi: 10.1378/chest.11-2004.

10. Merrell RC, Doarn CR. Tales of telemedicine: I met my doctor on television. Telemed J E Health. 2013 Aug;19(8):571-2. PMID: 23806083. doi: 10.1089/tmj.2013.9990.

11. Merrell RC, Doarn CR. Tales of telemedicine--telepsychiatry at work. Telemed J E Health. 2013 Apr;19(4):233-4. PMID: 23540275. doi: 10.1089/tmj.2013.9995.

12. Reddy KV. Using teledentistry for providing the specialist access to rural Indians. Indian J Dent Res. 2011 Mar-Apr;22(2):189. PMID: 21891882. doi: 10.4103/0970-9290.84275.

13. van der Heijden J. Teledermatology integrated in the Dutch national healthcare system. J Eur Acad Dermatol Venereol. 2010 May;24(5):615-6. PMID: WOS:000276491100022. doi: 10.1111/j.1468-3083.2010.03595.x.

14. Debnath D. Activity analysis of telemedicine in the UK. Postgrad Med J. 2004 Jun;80(944):335-8. PMID: WOS:000221943500009. doi: 10.1136/pgmj.2003.015453.

15. Militello C, La Iacona S, Serbanati LD, Ricci FL, Gilardi MC. A Virtual Health Record-based EHR System. 2015 E-Health and Bioengineering Conference. New York: Ieee; 2015.

16. Alanee S, Dynda D, LeVault K, Mueller G, Sadowski D, Wilber A, et al. Delivering kidney cancer care in rural Central and Southern Illinois: a telemedicine approach. Eur J Cancer Care (Engl). 2014 Nov;23(6):739-44. PMID: 25286964. doi: 10.1111/ecc.12248.

17. Baumgart DC. Digital advantage in the COVID-19 response: perspective from Canada's largest integrated digitalized healthcare system. npj Digit Med. 2020 Aug;3(1):4. PMID: WOS:000615150300001. doi: 10.1038/s41746-020-00326-y.

18. Jahangirian M, Taylor SJE. Profiling e-health projects in Africa: trends and funding patterns. Information Development. 2015 Jun;31(3):199-218. PMID: WOS:000353466900002. doi: 10.1177/0266666913511478.

19. Panait L, Doarn CR, Saftoiu A, Popovici C, Valeanu V, Merrell RC. A review of telemedicine in Romania. J Telemed Telecare. 2004;10(1):1-5. PMID: WOS:000189174400001. doi: 10.1258/135763304322764103.

20. Wagner S. Blood Pressure Self-Measurement. Adv Exp Med Biol. 2017;956:97-107. PMID: 27757934. doi: 10.1007/5584_2016_151.

21. Berger E. Telemedicine: has its time come? Ann Emerg Med. 2010 Nov;56(5):A15-7. PMID: 21064235. doi: 10.1016/j.annemergmed.2010.09.008.

22. Bain C, Alvandi AO. Digital Healthcare Across Oceania. CACM. 2020 Apr;63(4):64-7. PMID: WOS:000582585200022. doi: 10.1145/3378420.

23. Williams ML. Support for the clinician in providing a regional telehealth service. J Telemed Telecare. 2007;13(6):271-3. PMID: 17785021. doi: 10.1258/135763307781644942.

24. Afarikumah E. Electronic health in Ghana: current status and future prospects. Online J Public Health Inform. 2014;5(3):230. PMID: 24678382. doi: 10.5210/ojphi.v5i3.4943.

25. Merrell RC, Doarn CR. Telemedicine for vulnerable populations. Telemed J E Health. 2014 Oct;20(10):885-6. PMID: 25244668. doi: 10.1089/tmj.2014.9977.

26. Merchant R, Szefler SJ, Bender BG, Tuffli M, Barrett MA, Gondalia R, et al. Impact of a digital health intervention on asthma resource utilization. World Allergy Organization Journal. 2018 Dec;11:4. PMID: WOS:000451868800001. doi: 10.1186/s40413-018-0209-0.

27. Butler SM. After COVID-19: Thinking Differently About Running the Health Care System. JAMA. 2020 Jun 23;323(24):2450-1. PMID: 32573658. doi: 10.1001/jama.2020.8484.

28. Potter LA. 21st-century learning and health-care in the home - creating a national telecommunication network. Government Publications Review. 1993 Nov-Dec;20(6):732-3. PMID: WOS:A1993MQ00100018. doi: 10.1016/0277-9390(93)90093-5.

29. Giese KK. Coronavirus Disease 2019's Shake-up of Telehealth Policy: Application of Kingdon's Multiple Streams Framework. JNP-J Nurse Pract. 2020 Nov-Dec;16(10):768-70. PMID: WOS:000592038200021. doi: 10.1016/j.nurpra.2020.08.015.

30. Sensmeier J, Halley EC. Connecting Humans and Health through Health Information Exchange. In: Saranto K, Brennan PF, Park HA, Tallberg M, Ensio A, editors. Connecting Health and Humans. Amsterdam: Ios Press; 2009. p. 756-+.

31. Merz R. [MFA magazine "info practice team". Telemedicine establishes itself in rural areas]. MMW Fortschr Med. 2014 Jun 12;156(11):14. PMID: 25022077. doi: 10.1007/s15006-014-3143-2.

32. Varroud-Vial M. Improving diabetes management with electronic medical records. Diabetes Metab. 2011 Dec;37:S48-S52. PMID: WOS:000298834700003. doi: 10.1016/s1262-3636(11)70965-x.

33. Mader JK. Personal Experiences With Coronavirus Disease 2019 and Diabetes: The Time for Telemedicine is Now. J Diabetes Sci Technol. 2020 Jul;14(4):752-3. PMID: 32443942. doi: 10.1177/1932296820930289.

34. Makeham M. Role of digital technology in delivering 'healthy futures' and 'healthy cities'. Intern Med J. 2020 Nov;50(11):1408-9. PMID: WOS:000590567700018. doi: 10.1111/imj.15062.

35. Cook R. Introducing: telehealth and telecare. Br J Community Nurs. 2007 Jul;12(7):307. PMID: 17876934. doi: 10.12968/bjcn.2007.12.7.23823.

36. Demaerschalk BM. Telestrokologists: treating stroke patients here, there, and everywhere with telemedicine. Semin Neurol. 2010 Nov;30(5):477-91. PMID: 21207340. doi: 10.1055/s-0030-1268869.

37. Antohi R, Ogescu C, Bistriceanu D, Stefan L, Dumitru S. Electronic Health Records (EHR) in PROMED platform in accordance with the Romanian legislative framework. In: Bos L, Blobel B, Marsh A, Carroll D, editors. Medical and Care Compunetics 5. Amsterdam: Ios Press; 2008. p. 34-+.

38. Morrison Z, Robertson A, Cresswell K, Crowe S, Sheikh A. Understanding Contrasting Approaches to Nationwide Implementations of Electronic Health Record Systems: England, the USA and Australia. J Healthc Eng. 2011 Mar;2(1):25-41. PMID: WOS:000311668900002. doi: 10.1260/2040-2295.2.1.25.

39. McCartney M. Show us the evidence for telehealth. BMJ. 2012 Jan 18;344:e469. PMID: 22257981. doi: 10.1136/bmj.e469.

40. Robertson A, Cornford T, Barber N, Avery T, Sheikh A, T NHSCRSE. The NHS IT project: more than just a bad dream. Lancet. 2012 Jan;379(9810):29-30. PMID: WOS:000298913000032. doi: 10.1016/s0140-6736(12)60023-3.

41. Dodoo JE, Al-Samarraie H, Alsswey A. The development of telemedicine programs in Sub-Saharan Africa: Progress and associated challenges. Health and Technology. 2022 Jan;12(1):33-46. PMID: WOS:000722503200001. doi: 10.1007/s12553-021-00626-7.

42. Achey M, Aldred JL, Aljehani N, Bloem BR, Biglan KM, Chan P, et al. The Past, Present, and Future of Telemedicine for Parkinson's Disease. Mov Disord. 2014 Jun;29(7):871-83. PMID: WOS:000337670600011. doi: 10.1002/mds.25903.

43. Young AJ. New technologies and general practice. Br J Gen Pract. 2016 Dec;66(653):601-2. PMID: 27884890. doi: 10.3399/bjgp16X688021.

44. Shull JG. Digital Health and the State of Interoperable Electronic Health Records. JMIR Med Inf. 2019 Oct-Dec;7(4):259-66. PMID: WOS:000510198100019. doi: 10.2196/12712.

45. Matcham F, Hotopf M, Galloway J. Mobile apps, wearables and the future of technology in rheumatic disease care. Rheumatology (Oxford). 2019 Jul 1;58(7):1126-7. PMID: 30535022. doi: 10.1093/rheumatology/key391.

46. Müschenich M, Wamprecht L. [Health 4.0 - how are we doing tomorrow?]. Bundesgesundheitsblatt Gesundheitsforschung Gesundheitsschutz. 2018 Mar;61(3):334-9. PMID: 29411045. doi: 10.1007/s00103-018-2702-6.

47. Alverson DC, Edison K, Flournoy L, Korte B, Magruder C, Miller C. Telehealth tools for public health, emergency, or disaster preparedness and response: a summary report. Telemed J E Health. 2010 Jan-Feb;16(1):112-4. PMID: 20043703. doi: 10.1089/tmj.2009.0149.

48. Kesavadev J, Krishnan G, Mohan V. Digital health and diabetes: experience from India. Ther Adv Endocrinol Metab. 2021 Nov;12:13. PMID: WOS:000720867100001. doi: 10.1177/20420188211054676.

49. Lauer W, Sudhop T, Broich K. [E-health and medical devices : Federal Health Gazette Special Issue]. Bundesgesundheitsblatt Gesundheitsforschung Gesundheitsschutz. 2018 Mar;61(3):249-51. PMID: 29468403. doi: 10.1007/s00103-018-2714-2.

50. Tenderich A. Virtual Nation: Telemedicine's Breakout Moment. J Diabetes Sci Technol. 2020 Jul;14(4):799-800. PMID: 32429751. doi: 10.1177/1932296820929359.

51. May L. The National E-Health Transition Authority (NEHTA). Health Inf Manag. 2005;34(1):19-20. PMID: 18239225. doi: 10.1177/183335830503400106.

52. Robertson A, Cresswell K, Takian A, Petrakaki D, Crowe S, Cornford T, et al. Implementation and adoption of nationwide electronic health records in secondary care in England: qualitative analysis of interim results from a prospective national evaluation. Bmj-British Medical Journal. 2010 Sep;341:12. PMID: WOS:000281615700003. doi: 10.1136/bmj.c4564.

53. Emmanouilidou M, Burke M. A thematic review and a policy-analysis agenda of Electronic Health Records in the Greek National Health System. Health Policy. 2013 Jan;109(1):31-7. PMID: WOS:000314146100005. doi: 10.1016/j.healthpol.2012.09.010.

54. Donaldson I. Delivering the digital future. Br J Nurs. 2018 Oct 18;27(19):1136. PMID: 30346816. doi: 10.12968/bjon.2018.27.19.1136.

55. Gudi N, Lakiang T, Pattanshetty S, Sarbadhikari SN, John O. Challenges and prospects in india's digital health journey. Indian J Public Health. 2021 Apr-Jun;65(2):209-12. PMID: 34135195. doi: 10.4103/ijph.IJPH_1446_20.

56. Akbik F, Hirsch JA, Chandra RV, Frei D, Patel AB, Rabinov JD, et al. Telestroke-the promise and the challenge. Part one: growth and current practice. J Neurointerv Surg. 2017 Apr;9(4):357-60. PMID: 26984868. doi: 10.1136/neurintsurg-2016-012291.

57. Patel PS, Jiang B, Marcelli M, Mediwala SN, Vasudevan MM. Electronic Consultation: An Effective Alternative to In-Person Clinical Care for Patients With Diabetes Mellitus. J Diabetes Sci Technol. 2019 Jan;13(1):152-3. PMID: 30348023. doi: 10.1177/1932296818807472.

58. Webster PC. The pocketbook impact of electronic health records: Part 1. Fee-for-service billing is compatible with EHRs: Agreed. CMAJ. 2010 May 18;182(8):752-3. PMID: 20371650. doi: 10.1503/cmaj.109-3225.

59. Marcin JP. Telemedicine in the pediatric intensive care unit. Pediatr Clin North Am. 2013 Jun;60(3):581-92. PMID: 23639656. doi: 10.1016/j.pcl.2013.02.002.

60. Cilliers L, Wright G. Electronic Health Records in the Cloud: Improving Primary Health Care Delivery in South Africa. Stud Health Technol Inform. 2017;245:35-9. PMID: 29295047.

61. Russell A, Hellawell G. The future of electronic health records. Br J Hosp Med (Lond). 2013 Nov;74(11):604-5. PMID: 24220519. doi: 10.12968/hmed.2013.74.11.604.

62. Armstrong S. Finally, the NHS goes digital. Or does it? BMJ. 2015 Jul 13;351:h3726. PMID: 26169657. doi: 10.1136/bmj.h3726.

63. Pruitt S. The Office for the Advancement of Telehealth. Telemed J E Health. 2013 May;19(5):346-8. PMID: 23343256. doi: 10.1089/tmj.2012.0283.

64. Rayburn WF. The Role of Telemedicine in Improving Women's Health Care. Obstet Gynecol Clin North Am. 2020 Jun;47(2):xiii-xiv. PMID: 32451024. doi: 10.1016/j.ogc.2020.03.002.

65. Webster PC. The pocketbook impact of electronic health records: Part 2. Fee-for-service billing is compatible with EHRs: Disagreed. CMAJ. 2010 May 18;182(8):753-4. PMID: 20385742. doi: 10.1503/cmaj.109-3226.

66. Alsaleh S, editor. Toward Improving Mobile Health Services in the Kingdom of Saudi Arabia Based on the Saudi 2030 Vision. 2021 3rd International Conference on Electrical, Control and Instrumentation Engineering (ICECIE); 2021 27-27 Nov. 2021.

67. Miller A, Rhee E, Gettman M, Spitz A. The Current State of Telemedicine in Urology. Med Clin North Am. 2018 Mar;102(2):387-98. PMID: 29406066. doi: 10.1016/j.mcna.2017.10.014.

68. Oh JY, Park YT, Jo EC, Kim SM. Current Status and Progress of Telemedicine in Korea and Other Countries. Healthc Inform Res. 2015 Oct;21(4):239-43. PMID: WOS:000219441300005. doi: 10.4258/hir.2015.21.4.239.

69. Mosnaim GS, Stempel H, Van Sickle D, Stempel DA. The Adoption and Implementation of Digital Health Care in the Post-COVID-19 Era. J Allergy Clin Immunol Pract. 2020 Sep;8(8):2484-6. PMID: 32585407. doi: 10.1016/j.jaip.2020.06.006.

70. Carter A. Remote telemonitoring technology will expand, but progress depends on more research and federal support. Home Healthc Nurse. 2011 Jun;29(6):389-90. PMID: 21633231. doi: 10.1097/NHH.0b013e31821b71b9.

71. McKenzie R, Kanhutu KN. Telehealth quality check: Is it time for national standards? Aust J Gen Pract. 2021 Oct;50(10):778-81. PMID: 34590087. doi: 10.31128/ajgp-05-21-5967.

72. Khatri V, Peterson CB, Kyriazokos S, Prasad NR. A Review of Telemedicine Services in Finland. In: Dremstrup K, Rees S, Jensen O, editors. 15th Nordic-Baltic Conference on Biomedical Engineering and Medical Physics. New York: Springer; 2011. p. 1-8.

73. Mishra SK, Kapoor L, Singh IP. Telemedicine in India: Current Scenario and the Future. Telemed J E Health. 2009 Aug;15(6):568-75. PMID: WOS:000268744600013. doi: 10.1089/tmj.2009.0059.

74. Spence D. Bad medicine: The future is video consulting. Br J Gen Pract. 2018 Sep;68(674):437. PMID: 30166394. doi: 10.3399/bjgp18X698777.

75. Chidambaram S, Erridge S, Kinross J, Purkayastha S. Observational study of UK mobile health apps for COVID-19. Lancet Digit Health. 2020 Aug;2(8):e388-e90. PMID: 32835196. doi: 10.1016/s2589-7500(20)30144-8.

76. Ricci FL. The Italian national telemedicine programme. J Telemed Telecare. 2002;8(2):72-80. PMID: WOS:000175070300002. doi: 10.1258/1357633021937514.

77. Rockoff M. An Overview of Some Technological/Health-Care System Implications of Seven Exploratory Broad-Band Communication Experiments. IEEE Transactions on Communications. 1975;23(1):20-30. doi: 10.1109/TCOM.1975.1092657.

78. Chellaiyan VG, Nirupama AY, Taneja N. Telemedicine in India: Where do we stand? J Family Med Prim Care. 2019 Jun;8(6):1872-6. PMID: 31334148. doi: 10.4103/jfmpc.jfmpc_264_19.

79. Ahmad FS, Tsang T. Diabetes prevention, health information technology, and meaningful use: challenges and opportunities. Am J Prev Med. 2013 Apr;44(4 Suppl 4):S357-63. PMID: 23498299. doi: 10.1016/j.amepre.2012.12.020.

80. Wolf L, Harvell J, Jha AK. Hospitals ineligible for federal meaningful-use incentives have dismally low rates of adoption of electronic health records. Health Aff (Millwood). 2012 Mar;31(3):505-13. PMID: 22392661. doi: 10.1377/hlthaff.2011.0351.

81. Wong ZW, Cross HL. Telehealth in cancer care during the COVID-19 pandemic. Med J Aust. 2020 Sep;213(5):237-.e1. PMID: 32803783. doi: 10.5694/mja2.50740.

82. Moore MA, Coffman M, Jetty A, Petterson S, Bazemore A. Only 15% of FPs Report Using Telehealth; Training and Lack of Reimbursement Are Top Barriers. Am Fam Physician. 2016 Jan 15;93(2):101. PMID: 26926405.

83. Horvath L. Toward smart health care: Building a National Health Information Infrastructure (EESZT) in Hungary. New York: Ieee; 2017. 11- p. ISBN: 978-1-5386-4636-6.

84. Maon SN, Edirippulige S. An Overview of the National Telehealth Initiative in Malaysia. In: Smith AC, Maeder AJ, editors. Global Telehealth. Amsterdam: Ios Press; 2010. p. 95-103.

85. Alqifari S, Saleh SM, Habboush O, Ibrahim AA. Characteristics of Electronic Health Services in Saudi Arabia During the COVID-19 Pandemic. Cureus. 2022 Aug;14(8):e28441. PMID: 36176858. doi: 10.7759/cureus.28441.

86. Desborough J, Dykgraaf SH, de Toca L, Davis S, Roberts L, Kelaher C, et al. Australia's nationalCOVID-19 primary care response. Med J Aust. 2020 Aug;213(3):104-+. PMID: WOS:000547548700001. doi: 10.5694/mja2.50693.

87. AOA Health Information Technology and Telemedicine Committee. Common questions on electronic health records incentives. Optometry. 2010 Jun;81(6):327-9. PMID: 20723533. doi: 10.1016/j.optm.2010.04.086.

88. Aminpour F, Sadoughi F, Ahmadi M. Towards the Application of Open Source Software in Developing National Electronic Health Record- Narrative Review Article. Iran J Public Health. 2013 Dec;42(12):1333-9. PMID: WOS:000328843100001.

89. Bassi A, Arfin S, John O, Jha V. An overview of mobile applications (apps) to support the coronavirus disease 2019 response in India. Indian J Med Res. 2020 May;151(5):468-73. PMID: WOS:000549899900012. doi: 10.4103/ijmr.IJMR_1200_20.

90. Daschle T, Dorsey ER. The return of the house call. Ann Intern Med. 2015 Apr 21;162(8):587-8. PMID: 25894028. doi: 10.7326/m14-2769.

91. Stevanovic R, Pristas I, Uhernik AI, Stanic A. Development and deployment of a health information system in transitional countries - (Croatian experience). In: Bos L, Marsh A, editors. Medical and Care Compunetics 2. Amsterdam: I O S Press; 2005. p. 82-7.

92. Khoja S, Scott R, Husyin N, Durrani H, Arif M, Faqiri F, et al. Impact of simple conventional and Telehealth solutions on improving mental health in Afghanistan. J Telemed Telecare. 2016 Dec;22(8):495-8. PMID: WOS:000387352400010. doi: 10.1177/1357633x16674631.

93. Slomski A. Telehealth Success Spurs a Call for Greater Post-COVID-19 License Portability. JAMA. 2020 Sep 15;324(11):1021-2. PMID: 32930757. doi: 10.1001/jama.2020.9142.

94. Rohatgi R, Ross MJ, Majoni SW. Telenephrology: current perspectives and future directions. Kidney Int. 2017 Dec;92(6):1328-33. PMID: WOS:000415760400012. doi: 10.1016/j.kint.2017.06.032.

95. Doarn CR, Pruitt S, Jacobs J, Harris Y, Bott DM, Riley W, et al. Original Research Federal Efforts to Define and Advance Telehealth-A Work in Progress. Telemed e-Health. 2014 May;20(5):409-18. PMID: WOS:000335393400004. doi: 10.1089/tmj.2013.0336.

96. Shachar C, Engel J, Elwyn G. Implications for Telehealth in a Postpandemic Future: Regulatory and Privacy Issues. JAMA. 2020 Jun 16;323(23):2375-6. PMID: 32421170. doi: 10.1001/jama.2020.7943.

97. Nelson R. Electronic health records: useful tools or high-tech headache? Am J Nurs. 2007 Mar;107(3):25-6. PMID: 17314547. doi: 10.1097/00000446-200703000-00015.

98. Palkhivala A. Canada develops models of teleoncology. J Natl Cancer Inst. 2011 Nov 2;103(21):1566-8. PMID: 22010180. doi: 10.1093/jnci/djr449.

99. Gray CS, Mercer S, Palen T, McKinstry B, Hendry A. eHealth Advances in Support of People with Complex Care Needs: Case Examples from Canada, Scotland and the US. Healthc Q. 2016;19(2):29-37. PMID: 27700971. doi: 10.12927/hcq.2016.24696.

100. Switzer JA, Demaerschalk BM. Overcoming challenges to sustain a telestroke network. J Stroke Cerebrovasc Dis. 2012 Oct;21(7):535-40. PMID: 22819542. doi: 10.1016/j.jstrokecerebrovasdis.2012.06.014.

101. Shankar R, Newman C, McLean B, Anderson T, Obe JH. Can technology help reduce risk of harm in patients with epilepsy? Br J Gen Pract. 2015 Sep;65(638):448-9. PMID: 26324472. doi: 10.3399/bjgp15X686413.

102. Mamlin BW, Tierney WM. The Promise of Information and Communication Technology in Healthcare: Extracting Value From the Chaos. Am J Med Sci. 2016 Jan;351(1):59-68. PMID: WOS:000382663000010. doi: 10.1016/j.amjms.2015.10.015.

103. Barton AJ. eHealth national priorities. Clin Nurse Spec. 2015 Mar-Apr;29(2):66-7. PMID: 25654701. doi: 10.1097/nur.0000000000000100.

104. Miles N. Giving clinicians and patients the remote control. Br J Community Nurs. 2019 Aug 2;24(8):368-9. PMID: 31369317. doi: 10.12968/bjcn.2019.24.8.368.

105. Beaney P, Odulaja A, Hadley A, Prince C, Obe RC. GP Online: turning expectations into reality with the new NHS app. Br J Gen Pract. 2019 Apr;69(681):172-3. PMID: 30745356. doi: 10.3399/bjgp19X701333.

106. Black A, Sahama T, Gajanayake R. eHealth-as-a-Service (eHaaS): a data-driven decision making approach in Australian context. Stud Health Technol Inform. 2014;205:915-9. PMID: 25160321.

107. Barros LP, Carvalho AC, Barros Junior Ede M. Questioning the cost-benefit ratio of the telecardiology service in the state of Minas Gerais. Arq Bras Cardiol. 2012 Sep;99(3):865; author reply -6. PMID: 23011194. doi: 10.1590/s0066-782x2012001200013.

108. Kaufman N, Khurana I. Using Digital Health Technology to Prevent and Treat Diabetes. Diabetes Technol Ther. 2016 Feb;18 Suppl 1(Suppl 1):S56-68. PMID: 26836430. doi: 10.1089/dia.2016.2506.

109. Cross RK, Kane S. Integration of Telemedicine Into Clinical Gastroenterology and Hepatology Practice. Clin Gastroenterol Hepatol. 2017 Feb;15(2):175-81. PMID: WOS:000397246400009. doi: 10.1016/j.cgh.2016.09.011.

110. Rosemberg A, Schmid A, Plaut O. MonDossierMedical. ch - The Personal Health Record for Every Geneva Citizen. In: Sermeus W, Procter PM, Weber P, editors. Nursing Informatics 2016: Ehealth for All: Every Level Collaboration - from Project to Realization. Amsterdam: Ios Press; 2016. p. 700-2.

111. Mijares MT. Telemedicine in Ecuador: Failure or a learning experience? Kurokawa K, Nakajima I, Ishibashi Y, editors. New York: Ieee; 2004. 41-3 p. ISBN: 0-7803-8453-9.

112. Neufeld JD, Doarn CR, Aly R. State Policies Influence Medicare Telemedicine Utilization. Telemed J E Health. 2016 Jan;22(1):70-4. PMID: 26218148. doi: 10.1089/tmj.2015.0044.

113. Rodolfo I. Improving the senior wellbeing through telehealth support and integrated care. SIGACCESS Access Comput. 2016 (114):49–52. doi: 10.1145/2904092.2904102.

114. Santos R, Correia ME, Antunes L. Securing a health information system with a government issued digital identification card. Sanson LD, Fliegel K, editors. New York: Ieee; 2008. 135-+ p. ISBN: 978-1-4244-1816-9.

115. Traynor K. Telepharmacy services bring new patient care opportunities. Am J Health Syst Pharm. 2013 Apr 1;70(7):565-6. PMID: 23515504. doi: 10.2146/news130025.

116. Shah SM, Khan RA. Secondary Use of Electronic Health Record: Opportunities and Challenges. Ieee Access. 2020;8:136947-65. PMID: WOS:000558143200001. doi: 10.1109/access.2020.3011099.

117. Webster PC. Electronic health records a "strong priority" for US government. CMAJ. 2010 May 18;182(8):E315-6. PMID: 20351129. doi: 10.1503/cmaj.109-3218.

118. Kraschnewski JL, Gabbay RA. Role of health information technologies in the Patient-centered Medical Home. J Diabetes Sci Technol. 2013 Sep 1;7(5):1376-85. PMID: 24124967. doi: 10.1177/193229681300700530.

119. Sawal SH, Khan HM, Qayum M, Pervaiz N. Prospects of e-Health in federally administered tribal areas of Pakistan. J Coll Physicians Surg Pak. 2015 Mar;25(3):229-30. PMID: 25772971.

120. Huang F, Blaschke S, Lucas H. Beyond pilotitis: taking digital health interventions to the national level in China and Uganda. Global Health. 2017 Jul;13:11. PMID: WOS:000406696500001. doi: 10.1186/s12992-017-0275-z.

121. Hassounah M, Raheel H, Alhefzi M. Digital Response During the COVID-19 Pandemic in Saudi Arabia. J Med Internet Res. 2020 Sep;22(9):14. PMID: WOS:000568754600015. doi: 10.2196/19338.

122. Glasper A. Expanding the use of digital technology across the NHS. Br J Nurs. 2016 Oct 27;25(19):1078-9. PMID: 27792434. doi: 10.12968/bjon.2016.25.19.1078.

123. Rubin CB, Kovarik CL. The nuts and bolts of teledermatology: Preventing fragmented care. J Am Acad Dermatol. 2015 Nov;73(5):886-8. PMID: 26475549. doi: 10.1016/j.jaad.2015.06.046.

124. Mittal S, Movsowitz C, Varma N. The modern EP practice: EHR and remote monitoring. Cardiol Clin. 2014 May;32(2):239-52. PMID: 24793800. doi: 10.1016/j.ccl.2014.01.001.

125. Guttmann-Bauman I, Kono J, Lin AL, Ramsey KL, Boston BA. Use of Telehealth Videoconferencing in Pediatric Type 1 Diabetes in Oregon. Telemed J E Health. 2018 Jan;24(1):86-8. PMID: 28654350. doi: 10.1089/tmj.2017.0072.

126. Greiner AL. Telemedicine Applications in Obstetrics and Gynecology. Clin Obstet Gynecol. 2017 Dec;60(4):853-66. PMID: 28990981. doi: 10.1097/grf.0000000000000328.

127. Kassari P, Papaioannou P, Billiris A, Karanikas H, Eleftheriou S, Thireos E, et al. E-Health: A National Registry and Therapeutic Algorithm for the Prevention and Management of Overweight and Obesity in Childhood and Adolescence in Greece. Horm Res Paediatr. 2016;86:304-. PMID: WOS:000384166801108.

128. Prathiba V, Rema M. Teleophthalmology: a model for eye care delivery in rural and underserved areas of India. Int J Family Med. 2011;2011:683267. PMID: 22295192. doi: 10.1155/2011/683267.

129. Edison K. Telehealth and Outreach in the Post COVID-19 World: The MU Show-Me ECHO Program as a National Model. Mo Med. 2020 May-Jun;117(3):214-5. PMID: 32636552.

130. Simko A, Han SH, Aldana PR. Telemedicine: Providing Access to Care in Pediatric Neurosurgery to Underserved Communities. World Neurosurg. 2020 Jun;138:556-7. PMID: 32544993. doi: 10.1016/j.wneu.2020.03.181.

131. Ackerman MJ, Filart R, Burgess LP, Lee I, Poropatich RK. Developing Next-Generation Telehealth Tools and Technologies: Patients, Systems, and Data Perspectives. Telemed J E Health. 2010 Jan-Feb;16(1):93-5. PMID: WOS:000274181600015. doi: 10.1089/tmj.2009.0153.

132. Jha AK. Meaningful use of electronic health records: the road ahead. JAMA. 2010 Oct 20;304(15):1709-10. PMID: 20959581. doi: 10.1001/jama.2010.1497.

133. Alverson DC, Shannon S, Sullivan E, Prill A, Effertz G, Helitzer D, et al. Telehealth in the trenches: Reporting back from the frontlines in rural America. Telemed J E Health. 2004;10:S95-S109. PMID: WOS:000225457500014.

134. Dorsey ER, Topol EJ. State of Telehealth. N Engl J Med. 2016 Jul 14;375(2):154-61. PMID: 27410924. doi: 10.1056/NEJMra1601705.

135. Armstrong MJ, Booth C. Electronic health records in ambulatory care. N Engl J Med. 2008 Oct 23;359(17):1848; author reply 9. PMID: 18946074. doi: 10.1056/NEJMc081586.

136. Tchao ET, Acquah I, Kotey SD, Aggor CS, Kponyo JJ. On Telemedicine Implementations in Ghana. Int J Adv Comput Sci Appl. 2019 Mar;10(3):193-201. PMID: WOS:000464695700026.

137. Alkhaldi B, Sahama T, Huxley C, Gajanayake R. Barriers to implementing eHealth: a multi-dimensional perspective. Stud Health Technol Inform. 2014;205:875-9. PMID: 25160313.

138. Pluut B, Zuurmond A. Changing perspectives on informatics? A Comparison of Three National Electronic Health Records. Azevedo L, Londral AR, editors. Setubal: Insticc-Inst Syst Technologies Information Control & Communication; 2009. 416-+ p. ISBN: 978-989-8111-63-0.

139. Robertson A, Bates DW, Sheikh A. The rise and fall of England's National Programme for IT. J R Soc Med. 2011 Nov;104(11):434-5. PMID: WOS:000297453000002. doi: 10.1258/jrsm.2011.11k039.

140. Andrade AQ, Roughead EE. Consumer-directed technologies to improve medication management and safety. Med J Aust. 2019 Apr;210 Suppl 6:S24-s7. PMID: 30927471. doi: 10.5694/mja2.50029.

141. Kc A, Sunny AK, Poudel RP, Basnet O. A Review of eHealth Initiatives: Implications for Improving Health Service Delivery in Nepal. J Nepal Health Res Counc. 2019 Nov 13;17(3):269-77. PMID: 31735916. doi: 10.33314/jnhrc.v17i3.1787.

142. Woods D. eHealth: is it the panacea? Br J Community Nurs. 2013 May;18(5):213. PMID: 23752319. doi: 10.12968/bjcn.2013.18.5.213.

143. Tie M. Teleradiology in Australia: at the crossroads of electronic health. J Am Coll Radiol. 2011 Jan;8(1):71-3. PMID: 21211770. doi: 10.1016/j.jacr.2010.06.017.

144. Shoenbill K, Fost N, Tachinardi U, Mendonca EA. Genetic data and electronic health records: a discussion of ethical, logistical and technological considerations. Journal of the American Medical Informatics Association. 2014 Jan;21(1):171-80. PMID: WOS:000336716000025. doi: 10.1136/amiajnl-2013-001694.

145. Merrell RC, Doarn CR. Affordable Care Act and telemedicine. Telemed J E Health. 2015 Apr;21(4):243-4. PMID: 25831220. doi: 10.1089/tmj.2015.9995.

146. Merrell RC, Doarn CR. Telemedicine in Controversy-Again. Telemed J E Health. 2019 Aug;25(8):661-2. PMID: 31373893. doi: 10.1089/tmj.2019.29027.crd.

147. Borycki EM, Newsham D, Bates DW. eHealth in North America. Yearb Med Inform. 2013;8:103-6. PMID: 23974555.

148. Beger C, Haller H, Limbourg FP. [Telemonitoring and eHealth for arterial hypertension : Status quo and perspectives]. Internist (Berl). 2021 Mar;62(3):263-8. PMID: 33580308. doi: 10.1007/s00108-021-00966-6.

149. Persaud N. A national electronic health record for primary care. Can Med Assoc J. 2019 Jan;191(2):E28-E9. PMID: WOS:000456375000001. doi: 10.1503/cmaj.181647.

150. Wood D. The move to accountable care organizations includes telemedicine. Telemed J E Health. 2011 May;17(4):237-40. PMID: 21612522. doi: 10.1089/tmj.2011.9985.

151. Wangler J, Jansky M. [The national health portal: development opportunities and potential uses with special consideration of the general practitioner's perspective]. Bundesgesundheitsblatt Gesundheitsforschung Gesundheitsschutz. 2021 Feb 13. PMID: 33580814. doi: 10.1007/s00103-021-03288-y.

152. Xie B. Effects of an eHealth Literacy Intervention for Older Adults. J Med Internet Res. 2011 Oct-Dec;13(4):19. PMID: WOS:000299313300035. doi: 10.2196/jmir.1880.

153. McMullen PC, Howie WO, Philipsen N, Bryant VC, Setlow PD, Calhoun M, et al. Electronic Medical Records and Electronic Health Records: Overview for Nurse Practitioners. JNP-J Nurse Pract. 2014 Oct;10(9):56-61. PMID: WOS:000344219600008. doi: 10.1016/j.nurpra.2014.07.013.

154. Win AZ. Telepharmacy: Time to pick up the line. Res Social Adm Pharm. 2017 Jul-Aug;13(4):882-3. PMID: 26141501. doi: 10.1016/j.sapharm.2015.06.002.

155. Jaen CR. Successful Health Information Technology Implementation Requires Practice and Health Care System Transformation. Ann Fam Med. 2011 Sep-Oct;9(5):388-9. PMID: WOS:000295704100002. doi: 10.1370/afm.1307.

156. Kappal R, Mehndiratta A, Anandaraj P, Tsanas A. Current Impact, Future Prospects and Implications of Mobile Healthcare in India. Central Asian Journal of Global Health. 2014;3(1):15. PMID: WOS:000420804400002. doi: 10.5195/cajgh.2014.116.

157. Tragomalou A, Moschonis G, Manios Y, Kassari P, Ioakimidis I, Diou C, et al. Novel e-Health Applications for the Management of Cardiometabolic Risk Factors in Children and Adolescents in Greece. Nutrients. 2020 May 12;12(5). PMID: 32408523. doi: 10.3390/nu12051380.

158. Istepanian RSH. Telemedicine in the United Kingdom: current status and future prospects. IEEE Trans Inf Technol Biomed. 1999;3(2):158-9. doi: 10.1109/4233.767091.

159. Doarn CR. Development of Telemedicine and NASA's Contribution. Aerosp Med Hum Perform. 2015 May;86(5):504-5. PMID: 25945679. doi: 10.3357/amhp.4324.2015.

160. Stroetmann KA, Artmann J, Stroetmann V. Developing national eHealth infrastructures--results and lessons from Europe. AMIA Annu Symp Proc. 2011;2011:1347-54. PMID: 22195196.

161. Sudmeyer M, Wojtecki L, Schnitzler A. Teleneurology in Parkinson's disease. Diabetologe. 2012 Jun;8(4):275-9. PMID: WOS:000305683700007. doi: 10.1007/s11428-011-0837-2.

162. Ye Q, Zhou J, Wu H. Using Information Technology to Manage the COVID-19 Pandemic: Development of a Technical Framework Based on Practical Experience in China. JMIR Med Inf. 2020 Jun;8(6):9. PMID: WOS:000546388900025. doi: 10.2196/19515.

163. Oberer B. eGovernment in Healthcare: Smart Open Services for European Patients. In: Lee G, editor. Social Science and Health. Newark: Information Engineering Research Inst, USA; 2013. p. 233-8.

164. Wise J. UK government signals its support for telemedicine. BMJ. 2011 Nov 30;343:d7792. PMID: 22131213. doi: 10.1136/bmj.d7792.

165. Müller A, Cau A, Semakula M, Lodokiyiia P, Abdullahi O, Bullock M, et al. Digital mHealth and Virtual Care Use in Pandemics: A Rapid Landscape Review of Interventions Used Internationally During COVID-19 in 4 Countries. JMIR Form Res. 2021 Oct 8. PMID: 34932498. doi: 10.2196/26041.

166. Graves JM, Mackelprang JL, Amiri S, Abshire DA. Barriers to Telemedicine Implementation in Southwest Tribal Communities During COVID-19. J Rural Health. 2021 Jan;37(1):239-41. PMID: 32506685. doi: 10.1111/jrh.12479.

167. Tachinardi U. Internet and healthcare in Brazil: the role of the Working Group for Healthcare (GT Saude). Computers in Biology and Medicine. 1998 Sep;28(5):519-29. PMID: WOS:000077407500006. doi: 10.1016/s0010-4825(98)00031-6.

168. Ting X, Minjie W, Hu L, Yiwen Y, Hua H, Tao T, et al. Application of Telemedicine System With 4G and High-Resolution Video in Diagnosis and Treatment of Wounds Between Wound Healing Department and Community Health Care Center in China. Int J Low Extrem Wounds. 2011 Sep;10(3):167-8. PMID: WOS:000297302700008. doi: 10.1177/1534734611420493.

169. Dorsey ER, Topol EJ. Telemedicine 2020 and the next decade. Lancet. 2020 Mar 14;395(10227):859. PMID: 32171399. doi: 10.1016/s0140-6736(20)30424-4.

170. Akbik F, Hirsch JA, Chandra RV, Frei D, Patel AB, Rabinov JD, et al. Telestroke: the promise and the challenge. Part two: expansion and horizons. J Neurointerv Surg. 2017 Apr;9(4):361-+. PMID: WOS:000403146900012. doi: 10.1136/neurintsurg-2016-012291.

171. Yao J, Tan CS, Chen C, Tan J, Lim N, Müller-Riemenschneider F. Bright spots, physical activity investments that work: National Steps Challenge, Singapore: a nationwide mHealth physical activity programme. Br J Sports Med. 2020 Sep;54(17):1047-8. PMID: 31857340. doi: 10.1136/bjsports-2019-101662.

172. Kleinpell RM. Redefining the role of telehealth for cardiovascular disease management. Heart Lung. 2012 May;41(3):213-4. PMID: 22284857. doi: 10.1016/j.hrtlng.2012.01.003.

173. Litvin CB. In the dark--the case for electronic health records. N Engl J Med. 2007 Jun 14;356(24):2454-5. PMID: 17568027. doi: 10.1056/NEJMp068283.

174. Gray CS, Wodchis WP, Upshur R, Cott C, McKinstry B, Mercer S, et al. Supporting Goal-Oriented Primary Health Care for Seniors with Complex Care Needs Using Mobile Technology: Evaluation and Implementation of the Health System Performance Research Network, Bridgepoint Electronic Patient Reported Outcome Tool. JMIR RES Protoc. 2016 Apr-Jun;5(2):16. PMID: WOS:000381213600027. doi: 10.2196/resprot.5756.

175. Wang Z, Gu H. A review of telemedicine in China. J Telemed Telecare. 2009;15(1):23-7. PMID: 19139216. doi: 10.1258/jtt.2008.080508.

176. Al-Shorbaji N. E-health in the Eastern Mediterranean Region: a decade of challenges and achievements. East Mediterr Health J. 2008;14 Suppl:S157-73. PMID: 19205616.

177. Switzer JA, Levine SR, Hess DC. Telestroke 10 years later--'telestroke 2.0'. Cerebrovasc Dis. 2009;28(4):323-30. PMID: 19628933. doi: 10.1159/000229550.

178. Roland M. General practice by smartphone. BMJ. 2019 Jul 24;366:l4713. PMID: 31340952. doi: 10.1136/bmj.l4713.

179. Campanella N, Novelli S, Morosini P, Sampaolo G. Use of the citizen's electronic health record by doctors and patients in a mature application throughout a district of the national health system of Italy. Eur J Intern Med. 2017 Jan;37:e47-e9. PMID: 27769570. doi: 10.1016/j.ejim.2016.10.005.

180. Lau J, Knudsen J, Jackson H, Wallach AB, Bouton M, Natsui S, et al. Staying Connected In The COVID-19 Pandemic: Telehealth At The Largest Safety-Net System In The United States. Health Affairs. 2020 Aug;39(8):1437-42. PMID: WOS:000557072300023. doi: 10.1377/hlthaff.2020.00903.

181. Shah RU, Matheny ME. Data and Information in the Sea of Electronic Health Records. Circ Cardiovasc Qual Outcomes. 2018 Dec;11(12):e005247. PMID: 30562078. doi: 10.1161/circoutcomes.118.005247.

182. Pattillo RE. Federal Communications Commission provides funding for telehealth and telemedicine. Nurse Educ. 2013 Jul-Aug;38(4):168. PMID: 23778048. doi: 10.1097/NNE.0b013e318296db7a.

183. Wiweko B, Zesario A, Agung PG. Overview The Development of Tele Health and Mobile Health Application in Indonesia. 2016 International Conference on Advanced Computer Science and Information Systems. New York: Ieee; 2016. p. 9-13.

184. Laur A. Fear of e-Health records implementation? Med Leg J. 2015 Mar;83(1):34-9. PMID: 25027492. doi: 10.1177/0025817214540396.

185. Mihova P, Vinarova J, Pendzhurov I. One Telemedical Solution in Bulgaria. In: Combi C, Shahar Y, AbuHanna A, editors. Artificial Intelligence in Medicine, Proceedings. Berlin: Springer-Verlag Berlin; 2009. p. 196-200.

186. dos Santos AD, dos Santos SF, de Melo MDB, Silva EMD, Reis GA, de Souza C. Telehealth in Primary Healthcare: An Analysis of Belo Horizonte's Experience. Telemed e-Health. 2011 Jan-Feb;17(1):25-9. PMID: WOS:000292194800006. doi: 10.1089/tmj.2010.0099.

187. Woodall T, Ramage M, LaBruyere JT, McLean W, Tak CR. Telemedicine Services During COVID-19: Considerations for Medically Underserved Populations. J Rural Health. 2021 Jan;37(1):231-4. PMID: 32613657. doi: 10.1111/jrh.12466.

188. Chanda KL, Shaw JG. The development of telehealth as a strategy to improve health care services in Zambia. Health Info Libr J. 2010 Jun;27(2):133-9. PMID: 20565554. doi: 10.1111/j.1471-1842.2010.00876.x.

189. Vimarlund V, Le Rouge C. Barriers and Opportunities to the Widespread Adoption of Telemedicine: A Bi-Country Evaluation. In: Lehmann CU, Ammenwerth E, Nohr C, editors. Medinfo 2013: Proceedings of the 14th World Congress on Medical and Health Informatics, Pts 1 and 2. Amsterdam: Ios Press; 2013. p. 933-.

190. Wirth A. Thing Two and Thing One. Biomed Instrum Technol. 2018 Jan;52(1):67-9. PMID: 29350988. doi: 10.2345/0899-8205-52.1.67.

191. McLean S, Sheikh A. Does telehealthcare offer a patient-centred way forward for the community-based management of long-term respiratory disease? Prim Care Respir J. 2009 Sep;18(3):125-6. PMID: 19159046. doi: 10.3132/pcrj.2009.00006.

192. English JS, Eedy DJ. Has teledermatology in the U.K. finally failed? Br J Dermatol. 2007 Mar;156(3):411. PMID: 17300226. doi: 10.1111/j.1365-2133.2007.07750.x.

193. Basch P, Smith JRL. CMS Payment Policy, E&M Guideline Reform, and the Prospect of Electronic Health Record Optimization. Appl Clin Inform. 2018 Oct;9(4):914-8. PMID: 30586672. doi: 10.1055/s-0038-1676337.

194. Stefanacci RG, Guerin S, Van Aken T. The Electronic Health Record Foundation for Pharmaceutical Management. Popul Health Manag. 2018 Jun;21(3):170-1. PMID: 28829919. doi: 10.1089/pop.2017.0103.

195. Mattei P. Digital governance in tax-funded European healthcare systems: from the Back office to patient empowerment. Isr J Health Policy Res. 2020 Jan;9(1):5. PMID: WOS:000513654000001. doi: 10.1186/s13584-020-0361-1.

196. Parker SG, Hawley MS. Telecare for an ageing population? Age Ageing. 2013 Jul;42(4):424-5. PMID: 23775029. doi: 10.1093/ageing/aft056.

197. Trono D. Switzerland and the Digital Health Revolution. Chimia. 2016;70(12):851-2. PMID: WOS:000391165000005. doi: 10.2533/chimia.2016.851.

198. Lorentz MM. Telenursing and home healthcare. the many facets of technology. Home Healthc Nurse. 2008 Apr;26(4):237-43. PMID: 18408517. doi: 10.1097/01.Nhh.0000316702.22633.30.

199. Brennan J, McElligott A, Power N. National Health Models and the Adoption of E-Health and E-Prescribing in Primary Care - New Evidence from Europe. J Innov Health Inform. 2015 Nov 25;22(4):399-408. PMID: 26855274. doi: 10.14236/jhi.v22i4.97.

200. Magrabi F, Habli I, Sujan M, Wong D, Thimbleby H, Baker M, et al. Why is it so difficult to govern mobile apps in healthcare? BMJ Health Care Inform. 2019 Nov;26(1). PMID: 31744843. doi: 10.1136/bmjhci-2019-100006.

201. Malik AZ, editor. Telemedicine Country Report-Pakistan. 2007 9th International Conference on e-Health Networking, Application and Services; 2007 19-22 June 2007.

202. Khoja S, Durrani H, Ali Faheem Z. National representative body for e-health in Pakistan. J Telemed Telecare. 2009;15(6):321-2. PMID: 19720771. doi: 10.1258/jtt.2009.006001.

203. Mubaraki AA, Alrabie AD, Sibyani AK, Aljuaid RS, Bajaber AS, Mubaraki MA. Advantages and disadvantages of telemedicine during the COVID-19 pandemic era among physicians in Taif, Saudi Arabia. Saudi Med J. 2021 Jan;42(1):110-5. PMID: 33399180. doi: 10.15537/smj.2021.1.25610.

204. Doarn CR, Fitzgerald S, Rodas E, Harnett B, Prabe-Egge A, Merrell RC. Telemedicine to integrate intermittent surgical services into primary care. Telemed J E Health. 2002 Spr;8(1):131-7. PMID: WOS:000175637900012. doi: 10.1089/15305620252933473.

205. Graves S. Confidentiality, electronic health records, and the clinician. Perspect Biol Med. 2013 Winter;56(1):105-25. PMID: 23748530. doi: 10.1353/pbm.2013.0003.

206. Klar R, Pelikan E. Telemedicine in Germany. Kramme R, Hoffmann KP, Pozos RS, editors. Berlin: Springer-Verlag Berlin; 2011. 1119-27 p. ISBN: 978-3-540-74658-4; 978-3-540-74657-7.

207. Krupinski E, Nypaver M, Poropatich R, Ellis D, Safwat R, Sapci H. Clinical applications in telemedicine/telehealth. Telemed J E Health. 2002 Spr;8(1):13-34. PMID: WOS:000175637900003. doi: 10.1089/15305620252933374.

208. Sodhi M. Telehealth Policies Impacting Federally Qualified Health Centers in Face of COVID-19. J Rural Health. 2021 Jan;37(1):158-60. PMID: 32277779. doi: 10.1111/jrh.12445.

209. Chellaiyan VG, Nirupama AY, Taneja N. Telemedicine: new technology, new promises? Indian Journal of Community Health. 2019 Oct-Dec;31(4):437-41. PMID: WOS:000511333600004.

210. Patterson V, Wootton R. A web-based telemedicine system for low-resource settings 13 years on: insights from referrers and specialists. Glob Health Action. 2013 Sep 23;6:21465. PMID: 24063752. doi: 10.3402/gha.v6i0.21465.

211. Angaran DM. Telemedicine and telepharmacy: Current status and future implications. Am J Health Syst Pharm. 1999 Jul;56(14):1405-26. PMID: WOS:000081610500010. doi: 10.1093/ajhp/56.14.1405.

212. Neves AL, Villanueva T. French boost European telemedicine. CMAJ. 2011 Apr 19;183(7):E387-8. PMID: 21444621. doi: 10.1503/cmaj.109-3804.

213. Orlova AO, Dunnagan M, Finitzo T, Higgins M, Watkins T, Tien A, et al. Electronic health record - public health (EHR-PH) system prototype for interoperability in 21st century healthcare systems. AMIA Annu Symp Proc. 2005;2005:575-9. PMID: 16779105.

214. Sturgiss E, Desborough J, Dykgraaf SH, Matenge S, Dut G, Davis S, et al. Digital health to support primary care provision during a global pandemic. Aust Health Rev. 2022;46(3):269-72. PMID: WOS:000770555900001. doi: 10.1071/ah21263.

215. Bahadori M, Teymourzadeh E, Mousavi SM. eHealth solutions and nonurgent visits in emergency departments. Technol Health Care. 2018;26(3):571-2. PMID: 29843275. doi: 10.3233/thc-181290.

216. Doyle-Lindrud S. The Evolution of the Electronic Health Record. Clin J Oncol Nurs. 2015 Apr;19(2):153-4. PMID: WOS:000352449700011. doi: 10.1188/15.Cjon.153-154.

217. Lopes M, de Oliveira GMM, Amaral A, Pereira FSD. Window to the Future or Door to Chaos? Arq Bras Cardiol. 2019 Apr;112(4):461-5. PMID: WOS:000464635100019. doi: 10.5935/abc.20190056.

218. Gilbert GL, Degeling C, Johnson J. Communicable Disease Surveillance Ethics in the Age of Big Data and New Technology. Asian Bioethics Review. 2019 Jun;11(2):173-87. PMID: WOS:000538518200007. doi: 10.1007/s41649-019-00087-1.

Publications with an unfitting intervention setting

1. Wang S, Lee SB, Pardue C, Ramsingh D, Waller J, Gross H, et al. Remote evaluation of acute ischemic stroke - Reliability of National Institutes of Health Stroke Scale via telestroke. Stroke. 2003 Oct;34(10):E188-E91. PMID: WOS:000185679100016. doi: 10.1161/01.Str.0000091847.82140.9d.

2. Yildiz F, Oksuzoglu B. Teleoncology or telemedicine for oncology patients during the COVID-19 pandemic: the new normal for breast cancer survivors? Future Oncol. 2020 Oct;16(28):2191-5. PMID: 32857603. doi: 10.2217/fon-2020-0714.

3. Spindler M, Jacobs D, Yuan K, Tropea T, Teng CW, Perrone C, et al. A Department Approach to Teleneurology. Telemed J E Health. 2020 Dec 18. PMID: 33337290. doi: 10.1089/tmj.2020.0323.

4. Park YT, Han D. Current Status of Electronic Medical Record Systems in Hospitals and Clinics in Korea. Healthc Inform Res. 2017 Jul;23(3):189-98. PMID: WOS:000417088300008. doi: 10.4258/hir.2017.23.3.189.

5. Yenikomshian HA, Lerew TL, Tam M, Mandell SP, Honari SE, Pham TN. Evaluation of Burn Rounds Using Telemedicine: Perspectives from Patients, Families, and Burn Center Staff. Telemed J E Health. 2019 Jan;25(1):25-30. PMID: 29733269. doi: 10.1089/tmj.2017.0320.

6. Tierney WM, Achieng M, Baker E, Bell A, Biondich P, Braitstein P, et al. Experience Implementing Electronic Health Records in Three East African Countries. In: Safran C, Reti S, Marin HF, editors. Medinfo 2010, Pts I and Ii. Amsterdam: Ios Press; 2010. p. 371-5.

7. Grabowski DC, O'Malley AJ. Use of telemedicine can reduce hospitalizations of nursing home residents and generate savings for medicare. Health Aff (Millwood). 2014 Feb;33(2):244-50. PMID: 24493767. doi: 10.1377/hlthaff.2013.0922.

8. Szalewska D, Niedoszytko P, Gierat-Haponiuk K. The impact of professional status on the effects of and adherence to the outpatient followed by home-based telemonitored cardiac rehabilitation in patientes referred by a social insurance institution. International Journal of Occupational Medicine and Environmental Health. 2015;28(4):761-70. PMID: WOS:000358993900010. doi: 10.13075/ijomeh.1896.00494.

9. Ezenwa C, Brooks L. Understanding the introduction and use of a mobile device-supported health information system in Nigeria. Electron J Inf Syst Dev Ctries. 2014 Mar;62(1):20. PMID: WOS:000447039300008.

10. Westra I, Niessen FB. Implementing Real-Time Video Consultation in Plastic Surgery. Aesthetic Plast Surg. 2015 Oct;39(5):783-90. PMID: WOS:000360861800020. doi: 10.1007/s00266-015-0526-4.

11. Waddington P, Downs B. The Sandwell Telecare Project. Journal of Integrated Care. 2005;13(3):40-8. PMID: WOS:000217683200008. doi: 10.1108/14769018200500025.

12. Haozous E, Doorenbos AZ, Demiris G, Eaton LH, Towle C, Kundu A, et al. Role of telehealth/videoconferencing in managing cancer pain in rural American Indian communities. Psychooncology. 2012 Feb;21(2):219-23. PMID: WOS:000299415200014. doi: 10.1002/pon.1887.

13. Scalvini S, Rivadossi F, Comini L, Muiesan ML, Glisenti F. Telemedicine: the role of specialist second opinion for GPs in the care of hypertensive patients. Blood Press. 2011 Jun;20(3):158-65. PMID: 21241165. doi: 10.3109/08037051.2010.542646.

14. Feldman SS, Schooley BL, Bhavsar GP. Health information exchange implementation: lessons learned and critical success factors from a case study. JMIR Med Inform. 2014 Aug 15;2(2):e19. PMID: 25599991. doi: 10.2196/medinform.3455.

15. Emon TA, Prodhan UK, Rahman MZ, Jahan I. Improving Security of the Telemedicine System for the Rural People of Bangladesh. Int J Adv Comput Sci Appl. 2018 Jan;9(1):381-90. PMID: WOS:000426979200052.

16. Joshi AU, Lewiss RE. Telehealth in the time of COVID-19. Emerg Med J. 2020 Oct;37(10):637-8. PMID: 32753392. doi: 10.1136/emermed-2020-209846.

17. Abraham C, Nishihara E, Akiyama M. Transforming healthcare with information technology in Japan: A review of policy, people, and progress. International Journal of Medical Informatics. 2011 Mar;80(3):157-70. PMID: WOS:000287231900002. doi: 10.1016/j.ijmedinf.2011.01.002.

18. Smrke D, Cerkvenik G, Piskur-Kosmac D, Stankovski V. The Hip Status: A telemedical application. In: Kokol P, Zupan B, Stare J, Premik M, Engelbrecht R, editors. Medical Informatics Europe '99. Amsterdam: I O S Press; 1999. p. 241-4.

19. Wadali JS, Sood SP, Kaushish R, Syed-Abdul S, Khosla PK, Bhatia M. Evaluation of Free, Open-source, Web-based DICOM Viewers for the Indian National Telemedicine Service (eSanjeevani). J Digit Imaging. 2020 Dec;33(6):1499-513. PMID: WOS:000548094800001. doi: 10.1007/s10278-020-00368-4.

20. Zanaboni P, Scalvini S, Bernocchi P, Borghi G, Tridico C, Masella C. Teleconsultation service to improve healthcare in rural areas: acceptance, organizational impact and appropriateness. BMC Health Serv Res. 2009 Dec;9:9. PMID: WOS:000273573200002. doi: 10.1186/1472-6963-9-238.

21. Martinez-Fernandez A, Lobos-Medina I, Diaz-Molina CA, Chen-Cruz MF, Prieto-Egido I. TulaSalud: An m-health system for maternal and infant mortality reduction in Guatemala. J Telemed Telecare. 2015 Jul;21(5):283-91. PMID: WOS:000357495500005. doi: 10.1177/1357633x15575830.

22. Wiegman R, Manson SM, Hansen AL, Huggins A, Trullinger L. The Native Telehealth Outreach and Technical Assistance Program: A community-based approach to the development of multimedia-focused health care information. Am Indian Alsk Native Ment Health Res. 2007;14(2):49-66. PMID: WOS:000253286000004.

23. Yang L, Brown-Johnson CG, Miller-Kuhlmann R, Kling SMR, Saliba-Gustafsson EA, Shaw JG, et al. Accelerated launch of video visits in ambulatory neurology during COVID-19: Key lessons from the Stanford experience. Neurology. 2020 Aug 18;95(7):305-11. PMID: 32611634. doi: 10.1212/wnl.0000000000010015.

24. Chau S, Oldman S, Smith SR, Lin CA, Ali S, Duffy VB. Online Behavioral Screener with Tailored Obesity Prevention Messages: Application to a Pediatric Clinical Setting. Nutrients. 2021 Jan;13:223. doi: https://doi.org/10.3390/nu13010223.

25. Ybarra ML, Prescott T, Mustanski B, Parsons J, Bull SS. Feasibility, Acceptability, and Process Indicators for Guy2Guy, an mHealth HIV Prevention Program for Sexual Minority Adolescent Boys. J Adolesc Health. 2019 Sep;65(3):417-22. PMID: WOS:000482179300019. doi: 10.1016/j.jadohealth.2019.04.025.

26. Almonacid C, Blanco-Aparicio M, Domínguez-Ortega J, Giner J, Molina J, Plaza V. Teleconsultation in the follow-up of the asthma patient. Lessons after COVID-19. Arch Bronconeumol. 2021 Jan;57 Suppl 1:13-4. PMID: 33229047. doi: 10.1016/j.arbres.2020.10.005.

27. Vossebeld DM, Puik ECN, Jaspers JEN, Schuurmans MJ. Development process of a mobile electronic medical record for nurses: a single case study. Bmc Medical Informatics and Decision Making. 2019 Jan;19:12. PMID: WOS:000455584800001. doi: 10.1186/s12911-018-0726-3.

28. Abramson EL, Pfoh ER, Barron Y, Quaresimo J, Kaushal R. The Effects of Electronic Prescribing by Community-Based Providers on Ambulatory Medication Safety. Jt Comm J Qual Patient Saf. 2013 Dec;39(12):545-52. PMID: WOS:000443972000002. doi: 10.1016/s1553-7250(13)39070-9.

29. Safran C, Pompilio-Weitzner G, Emery KD, Hampers L. A Medicaid eHealth program: an analysis of benefits to users and nonusers. AMIA Annu Symp Proc. 2005;2005:659-63. PMID: 16779122.

30. Abramson EL, McGinnis S, Edwards A, Maniccia DM, Moore J, Kaushal R, et al. Electronic health record adoption and health information exchange among hospitals in New York State. J Eval Clin Pract. 2012 Dec;18(6):1156-62. PMID: WOS:000310487300007. doi: 10.1111/j.1365-2753.2011.01755.x.

31. Suselj M, Zuffada R. Netc@rds for e-EHIC - a Step Towards the Introduction of the European Health Insurance Card. In: Cunningham P, Cunningham M, editors. Innovation and the Knowledge Economy: Issues, Applications, Case Studies, Pts 1 & 2. Amsterdam: Ios Press; 2005. p. 445-51.

32. Ye Q, Deng ZH, Chen YY, Liao JZ, Li G, Lu YB. How Resource Scarcity and Accessibility Affect Patients' Usage of Mobile Health in China: Resource Competition Perspective. Jmir Mhealth and Uhealth. 2019 Aug;7(8):14. PMID: WOS:000482775000001. doi: 10.2196/13491.

33. Zive DM, Cook J, Yang C, Sibell D, Tolle SW, Lieberman M. Implementation of a Novel Electronic Health Record-Embedded Physician Orders for Life-Sustaining Treatment System. J Med Syst. 2016 Nov;40(11):245. PMID: 27696173. doi: 10.1007/s10916-016-0605-3.

34. Seibert PS, Whitmore TA, Patterson C, Parker PD, Otto C, Basom J, et al. Telemedicine Facilitates CHF Home Health Care for Those with Systolic Dysfunction. International Journal of Telemedicine and Applications. 2008;2008:7. PMID: WOS:000214704100008. doi: 10.1155/2008/235031.

35. Mrak G, Paladino J, Dzubur A, Desnica A. Telemedicine in neurosurgery: teleradiology connections in the Republic of Croatia. J Telemed Telecare. 2009;15(3):142-4. PMID: 19364899. doi: 10.1258/jtt.2009.003012.

36. Traub SJ, Butler R, Chang YH, Lipinski C. Emergency department physician telemedical triage. Telemed J E Health. 2013 Nov;19(11):841-5. PMID: 24093916. doi: 10.1089/tmj.2013.0026.

37. Khatun F, Ahmed NU, Rahman H, Roy SS, Chowdhury SA, Chowdhury R, et al. The Promise of Teleconsultation in the Era of Pandemic: A Case from Bangladesh. Telemed e-Health.5. PMID: WOS:000831626500001. doi: 10.1089/tmj.2021.0529.

38. Correia R, Kon F, Kon R. Borboleta: a mobile telehealth system for primary homecare. Proceedings of the 2008 ACM symposium on Applied computing; Fortaleza, Ceara, Brazil: Association for Computing Machinery; 2008. p. 1343–7.

39. Gleason N, Prasad PA, Ackerman S, Ho C, Monacelli J, Wang M, et al. Adoption and impact of an eConsult system in a fee-for-service setting. Healthc (Amst). 2017 Mar;5(1-2):40-5. PMID: 27469441. doi: 10.1016/j.hjdsi.2016.05.005.

40. Steinman L, van Pelt M, Hen H, Chhorvann C, Lan CS, Te V, et al. Can mHealth and eHealth improve management of diabetes and hypertension in a hard-to-reach population? -lessons learned from a process evaluation of digital health to support a peer educator model in Cambodia using the RE-AIM framework. Mhealth. 2020;6:40. PMID: 33437836. doi: 10.21037/mhealth-19-249.

41. van der Voort PH, de Metz J, Wester JP, van Stijn I, Feijen HM, Balzereit A, et al. Telemedicine in a Dutch intensive care unit: A descriptive study of the first results. J Telemed Telecare. 2016 Apr;22(3):141-7. PMID: 26141722. doi: 10.1177/1357633x15590751.

42. Aguirre RR, Suarez O, Fuentes M, Sanchez-Gonzalez MA. Electronic Health Record Implementation: A Review of Resources and Tools. Cureus. 2019 Sep 13;11(9):e5649. PMID: 31700751. doi: 10.7759/cureus.5649.

43. Lux A. Cost-benefit analysis of a new health insurance card and electronic prescription in Germany. J Telemed Telecare. 2002;8:54-5. PMID: WOS:000177549700025. doi: 10.1258/135763302320302028.

44. Jusril H, Ariawan I, Damayanti R, Lazuardi L, Musa M, Wulandari SM, et al. Digital health for real-time monitoring of a national immunisation campaign in Indonesia: a large-scale effectiveness evaluation. BMJ Open. 2020 Dec 10;10(12):e038282. PMID: 33303436. doi: 10.1136/bmjopen-2020-038282.

45. Weinstein RS, Lopez AM, Joseph BA, Erps KA, Holcomb M, Barker GP, et al. Telemedicine, Telehealth, and Mobile Health Applications That Work: Opportunities and Barriers. Am J Med. 2014 Mar;127(3):183-7. PMID: WOS:000331725200018. doi: 10.1016/j.amjmed.2013.09.032.

46. Salvador CH, Ruiz-Sanchez A, González de Mingo MA, Carmona Rodríguez M, Carrasco MP, Sagredo PG, et al. Evaluation of a telemedicine-based service for the follow-up and monitoring of patients treated with oral anticoagulant therapy. IEEE Trans Inf Technol Biomed. 2008 Nov;12(6):696-706. PMID: 19000948. doi: 10.1109/titb.2008.910750.

47. Sharma R, Fleischut P, Barchi D. Telemedicine and its transformation of emergency care: a case study of one of the largest US integrated healthcare delivery systems. Int J Emerg Med. 2017 Jul;10:4. PMID: WOS:000405430300001. doi: 10.1186/s12245-017-0146-7.

48. Greenhalgh T, Vijayaraghavan S, Wherton J, Shaw S, Byrne E, Campbell-Richards D, et al. Virtual online consultations: advantages and limitations (VOCAL) study. BMJ Open. 2016 Jan 29;6(1):e009388. PMID: 26826147. doi: 10.1136/bmjopen-2015-009388.

49. Ybarra ML, Prescott TL, Phillips GL, Bull SS, Parsons JT, Mustanski B. Pilot RCT Results of an mHealth HIV Prevention Program for Sexual Minority Male Adolescents. Pediatrics. 2017 Jul;140(1):10. PMID: WOS:000404482500010. doi: 10.1542/peds.2016-2999.

50. Abramson EL, Malhotra S, Fischer K, Edwards A, Pfoh ER, Osorio SN, et al. Transitioning between electronic health records: effects on ambulatory prescribing safety. J Gen Intern Med. 2011 Aug;26(8):868-74. PMID: 21499828. doi: 10.1007/s11606-011-1703-z.

51. Sheet D. Toward a Comprehensive Cure: Digital information and communication technology is helping to meet health care challenges in India. IEEE Pulse. 2016 Nov-Dec;7(6):34-7. PMID: 27875116. doi: 10.1109/mpul.2016.2607140.

52. Tambo T, Hoffmann-Petersen N, Bejder K, Saha P. Architecting for Connected Healthcare: A Case of Telehomecare and Hypertension. Hersey: Igi Global; 2012. 306-25 p. ISBN: 978-1-4666-1825-1; 978-1-4666-1824-4.

53. Al-Qirim N. Realizing telemedicine advantages at the national level: cases from the United Arab Emirates. Telemed J E Health. 2007 Oct;13(5):545-55. PMID: 17999616. doi: 10.1089/tmj.2006.0087.

54. Schwarze JC, Tessmann S, Sassenberg C, Muller M, Prokosch HU, Uckert F. A modular electronic health record as an answer to communication problems in health care. Wirtschaftsinformatik. 2005;47(3):187-95. PMID: WOS:000230390300004. doi: 10.1007/bf03254899.

55. Sepper R, Ross P, Tiik M. Nationwide Health Data Management System: A Novel Approach for Integrating Biomarker Measurements with Comprehensive Health Records in Large Populations Studies. J Proteome Res. 2011 Jan;10(1):97-100. PMID: WOS:000285812000011. doi: 10.1021/pr1007784.

56. Bradley M, Black P, Noble S, Thompson R, Lamey PJ. Application of teledentistry in oral medicine in a community dental service, N. Ireland. Br Dent J. 2010 Oct 23;209(8):399-404. PMID: 20966999. doi: 10.1038/sj.bdj.2010.928.

57. Tresenriter M, Holdaway J, Killeen J, Chan T, Dameff C. The Implementation of an Emergency Medicine Telehealth System During a Pandemic. J Emerg Med. 2021 Apr;60(4):548-53. PMID: 33423835. doi: 10.1016/j.jemermed.2020.11.026.

58. Pires ETC, Cheng C, de Castro Silva SLF, Gois SR, Sabbadini FS, Gonçalves AA. The Implementation of a Mobile APP for Cancer Care Management at the Brazilian National Cancer Institute. Stud Health Technol Inform. 2022 Jan 14;289:353-6. PMID: 35062165. doi: 10.3233/shti210932.

59. Wrenn K, Catschegn S, Cruz M, Gleason N, Gonzales R. Analysis of an electronic consultation program at an academic medical centre: Primary care provider questions, specialist responses, and primary care provider actions. J Telemed Telecare. 2017 Feb;23(2):217-24. PMID: 26940797. doi: 10.1177/1357633x16633553.

60. Piette JD, Valverde H, Marinec N, Jantz R, Kamis K, de la Vega CL, et al. Establishing an independent mobile health program for chronic disease self-management support in Bolivia. Front Public Health. 2014;2:10. PMID: WOS:000498911400091. doi: 10.3389/fpubh.2014.00095.

61. Sasangohar F, Bradshaw MR, Carlson MM, Flack JN, Fowler JC, Freeland D, et al. Adapting an Outpatient Psychiatric Clinic to Telehealth During the COVID-19 Pandemic: A Practice Perspective. J Med Internet Res. 2020 Oct;22(10):9. PMID: WOS:000582525800004. doi: 10.2196/22523.

62. Tabbara M, Hodel T, Muller U, Briner G, Zimmermann H, Exadaktylos AK. DoWe Need New Personalized Emergency Telehealth Solutions? A Survey of 100 Emergency Department Patients and a First Report of the Swiss Limmex EmergencyWristwatch: An Original Study. International Journal of Telemedicine and Applications. 2012;2012:5. PMID: WOS:000214708100018. doi: 10.1155/2012/736264.

63. Ryan MS, Shih SC, Winther CH, Wang JJ. Does it Get Easier to Use an EHR? Report from an Urban Regional Extension Center. J Gen Intern Med. 2014 Oct;29(10):1341-8. PMID: WOS:000342453900009. doi: 10.1007/s11606-014-2891-0.

64. Abramson EL, Patel V, Malhotra S, Pfoh ER, Nena Osorio S, Cheriff A, et al. Physician experiences transitioning between an older versus newer electronic health record for electronic prescribing. Int J Med Inform. 2012 Aug;81(8):539-48. PMID: 22465355. doi: 10.1016/j.ijmedinf.2012.02.010.

65. Van Dillen C, Silvestri S, Haney M, Ralls G, Zuver C, Freeman D, et al. Evaluation of an off-the-shelf mobile telemedicine model in emergency department wound assessment and management. J Telemed Telecare. 2013 Feb;19(2):84-8. PMID: 23470449. doi: 10.1177/1357633x13476231.

66. Bonacina S, Masseroli M. A web application for managing data of cardiovascular risk patients. New York: Ieee; 2006. 4307-+ p. ISBN: 978-1-4244-0032-4.

67. Ybarra M, Goodenow C, Rosario M, Saewyc E, Prescott T. An mHealth Intervention for Pregnancy Prevention for LGB Teens: An RCT. Pediatrics. 2021 Feb 10. PMID: 33568491. doi: 10.1542/peds.2020-013607.

68. Fazal N, Webb A, Bangoura J, El Nasharty M. Telehealth: improving maternity services by modern technology. BMJ Open Qual. 2020 Nov;9(4). PMID: 33148603. doi: 10.1136/bmjoq-2019-000895.

69. Finkelstein J, O'Connor G, Friedman RH. Development and implementation of the Home Asthma Telemonitoring (HAT) system to facilitate asthma self-care. In: Patel VL, Rogers R, Haux R, editors. Medinfo 2001: Proceedings of the 10th World Congress on Medical Informatics, Pts 1 and 2. Amsterdam: Ios Press; 2001. p. 810-4.

70. Yen YF, Tsai YF, Su VY, Chan SY, Yu WR, Ho H, et al. Use and Cost-Effectiveness of a Telehealth Service at a Centralized COVID-19 Quarantine Center in Taiwan: Cohort Study. J Med Internet Res. 2020 Dec 11;22(12):e22703. PMID: 33259324. doi: 10.2196/22703.

71. Miscione G. Telemedicine in the Upper Amazon: Interplay with local health care practices. Mis Quarterly. 2007 Jun;31(2):403-25. PMID: WOS:000246728300010.

72. Singh T, Ngoh CLY, Wong WK, Khan BA. Impact of Telemedicine on Hospitalisation and Mortality Rates in Community-Based Haemodialysis Centres in Singapore During the COVID-19 Pandemic. Annals Academy of Medicine Singapore. 2020 Oct;49(10):756-63. PMID: WOS:000591608700006. doi: 10.47102/annals-acadmedsg.2020369.

73. Muqri H, Shrivastava A, Muhtadi R, Chuck RS, Mian UK. The Cost-Effectiveness of a Telemedicine Screening Program for Diabetic Retinopathy in New York City. Clinical Ophthalmology. 2022;16:1505-12. PMID: WOS:000800466600001. doi: 10.2147/opth.S357766.

74. Taghavi K, Banerjee D, Mandal R, Kallner HK, Thorsell M, Friis T, et al. Colposcopy telemedicine: live versus static swede score and accuracy in detecting CIN2+, a cross-sectional pilot study. BMC Womens Health. 2018 Jun 11;18(1):89. PMID: 29890991. doi: 10.1186/s12905-018-0569-1.

75. Woods K, Kutlar A, Grigsby RK, Adams L, Stachura ME. Primary-care delivery for sickle cell patients in rural Georgia using telemedicine. Telemed J. 1998 Win;4(4):353-61. PMID: WOS:000078257400011. doi: 10.1089/tmj.1.1998.4.353.

76. Wang GY, Liu XH, Xie K, Chen N, Chen T. DeepTriager: A Neural Attention Model for Emergency Triage with Electronic Health Records. In: Yoo IH, Bi JB, Hu X, editors. 2019 Ieee International Conference on Bioinformatics and Biomedicine; New York: Ieee; 2019. p. 978-82.

77. Acheampong F, Vimarlund V, Informat Resources Management A. Innovating Healthcare through Remote Monitoring: Effects and Business Model. Hersey: Igi Global; 2018. 247-68 p. ISBN: 978-1-5225-3927-8; 978-1-5225-3926-1.

78. Ratliff CR, Shifflett R, Howell A, Kennedy C. Telehealth for Wound Management During the COVID-19 Pandemic Case Studies. Journal of Wound Ostomy and Continence Nursing. 2020 Sep-Oct;47(5):445-9. PMID: WOS:000579196000006. doi: 10.1097/won.0000000000000692.

79. Sangare M, Tanner L, Voss S, Laureys F, Hollow D, Toure M. A national teleradiology programme in Mali: implementation and results. J Telemed Telecare. 2015 Apr;21(3):131-8. PMID: WOS:000354530300002. doi: 10.1177/1357633x15569966.

80. Tse MMY, Choi KCY, Leung RSW. E-health for older people: The use of technology in health promotion. CyberPsychol Behav. 2008 Aug;11(4):475-9. PMID: WOS:000258946800013. doi: 10.1089/cpb.2007.0151.

81. Boydell N, Reynolds-Wright JJ, Cameron ST, Harden J. Women's experiences of a telemedicine abortion service (up to 12 weeks) implemented during the coronavirus (COVID-19) pandemic: a qualitative evaluation. BJOG. 2021 Oct;128(11):1752-61. PMID: 34138505. doi: 10.1111/1471-0528.16813.

82. Weber JL, Blanc D, Dittmar A, Comet B, Corroy C, Noury N, et al. VTAM - A new "biocloth" for ambulatory telemonitoring. Itab 2003: 4th International Ieee Embs Special Topic Conference on Information Technology Applications in Biomedicine, Conference Proceedings: New Solutions for New Challenges. New York: Ieee; 2003. p. 299-301.

83. Tragomalou A, Moschonis G, Kassari P, Papageorgiou I, Genitsaridi SM, Karampatsou S, et al. A National e-Health Program for the Prevention and Management of Overweight and Obesity in Childhood and Adolescence in Greece. Nutrients. 2020 Sep 18;12(9). PMID: 32961973. doi: 10.3390/nu12092858.

Publications where the intervention does not fit the definition of digital public health

1. Stalker HJ, Wilson R, McCune H, Gonzalez J, Moffett M, Zori RT. Telegenetic medicine: improved access to services in an underserved area. J Telemed Telecare. 2006;12(4):182-5. PMID: 16774698. doi: 10.1258/135763306777488762.

2. Marinescu R, Nedelcu A. Smartphone Application for Heart Rate Monitoring. 2017 Ieee International Conference on E-Health and Bioengineering Conference. New York: Ieee; 2017. p. 141-4.

3. Giansanti D, Morelli S, Maccioni G, Lanzetta M, Macellari V. Health technology assessment of a homecare device for telemonitoring and telerehabilitation for patients after hand transplantation. Telemed J E Health. 2008 Jan-Feb;14(1):69-75. PMID: 18328027. doi: 10.1089/tmj.2007.0023.

4. Morrison JG. Telehealth application in occupational health. Stud Health Technol Inform. 2015;209:109-13. PMID: 25980712.

5. Sampa MB, Hoque MR, Islam R, Nishikitani M, Nakashima N, Yokota F, et al. Redesigning Portable Health Clinic Platform as a Remote Healthcare System to Tackle COVID-19 Pandemic Situation in Unreached Communities. Int J Environ Res Public Health. 2020 Jun 30;17(13). PMID: 32629963. doi: 10.3390/ijerph17134709.

6. Nyatuka DR, Harpe Rdl. Evaluating mHealth Interventions in an Underserved Context Using Service Design Strategy: A Case of Kenya. Proceedings of the third International Conference on Medical and Health Informatics 2019; Xiamen, China: Association for Computing Machinery; 2019. p. 153–60.

7. Gregoski MJ, Newton J, Ling CG, Blaylock K, Smith SA, Paguntalan J, et al. Effective weight-loss using an e-health delivered physical activity and dietary intervention: A federal credit union pilot study. Work. 2016 Apr 6;54(1):127-34. PMID: 27061690. doi: 10.3233/wor-162282.

8. Pearl PL, Sable C, Evans S, Knight J, Cunningham P, Lotrecchiano GR, et al. International telemedicine consultations for neurodevelopmental disabilities. Telemed J E Health. 2014 Jun;20(6):559-62. PMID: 24660879. doi: 10.1089/tmj.2013.0275.

9. Quantin C, Cohen O, Riandey B, Allaert FA. The French proposal for a health identification number. In: Hasman A, Haux R, VanderLei J, DeClercq E, France FHR, editors. Ubiquity: Technologies for Better Health in Aging Societies. Amsterdam: Ios Press; 2006. p. 201-+.

10. Simonaitis L, Dixon BE, Belsito A, Miller T, Overhage JM. Building a production-ready infrastructure to enhance medication management: early lessons from the nationwide health information network. AMIA Annu Symp Proc. 2009 Nov 14;2009:609-13. PMID: 20351927.

11. Moya M, Valdez J, Yonas H, Alverson DC. The impact of a telehealth web-based solution on neurosurgery triage and consultation. Telemed J E Health. 2010 Nov;16(9):945-9. PMID: 21034238. doi: 10.1089/tmj.2010.0044.

12. Bodemeyer J, Mariss G, Gursky S. Teleradiology - a blessing or a curse. Radiologe. 2002 Feb;42(2):71-81. PMID: WOS:000174788400003. doi: 10.1007/s00117-001-0694-x.

13. Plinkert PK, Plinkert B, Fuchs M, Zenner HP. Telemedicine applications in ENT - The Tubingen and Leipzig videoconference example. HNO. 2000 Oct;48(10):728-34. PMID: WOS:000090012200002. doi: 10.1007/s001060050649.

14. Saha S, Kotwani P, Pandya A, Patel C, Shah K, Saxena D, et al. Addressing comprehensive primary healthcare in Gujarat through mHealth intervention: Early implementation experience with TeCHO+ programme. J Family Med Prim Care. 2020 Jan;9(1):340-6. PMID: 32110616. doi: 10.4103/jfmpc.jfmpc_835_19.

15. Miyoshi NSB, De Azevedo-Marques JM, Alves D, De Azevedo-Marques PM. An eHealth Platform for the Support of a Brazilian Regional Network of Mental Health Care (eHealth-Interop): Development of an Interoperability Platform for Mental Care Integration. JMIR Ment Health. 2018 Dec;5(4):17. PMID: WOS:000453611300001. doi: 10.2196/10129.

16. Agbakoba R, McGee-Lennon M, Bouamrane MM, Watson N, Mair FS. Implementation factors affecting the large-scale deployment of digital health and well-being technologies: A qualitative study of the initial phases of the 'Living-It-Up' programme. Health Informatics J. 2016 Dec;22(4):867-77. PMID: 26276795. doi: 10.1177/1460458215594651.

17. Zulman DM, Damschroder LJ, Smith RG, Resnick PJ, Sen A, Krupka EL, et al. Implementation and evaluation of an incentivized Internet-mediated walking program for obese adults. Transl Behav Med. 2013 Dec;3(4):357-69. PMID: 24294324. doi: 10.1007/s13142-013-0211-6.

18. Quantin C, Allaert FA, Avillach P, Riandey B, Fieschi M, Fassa M, et al. Proposal of a French Health Identification Number Interoperable at the European Level. In: Kuhn KA, Warren JR, Leong TY, editors. Medinfo 2007: Proceedings of the 12th World Congress on Health. Amsterdam: Ios Press; 2007. p. 503-+.

19. Munsch N, Martin A, Gruarin S, Nateqi J, Abdarahmane I, Weingartner-Ortner R, et al. Diagnostic Accuracy of Web-Based COVID-19 Symptom Checkers: Comparison Study. J Med Internet Res. 2020 Oct 6;22(10):e21299. PMID: 33001828. doi: 10.2196/21299.

20. Breuer L, Schwab S. [Telemedicine in stroke care]. Med Klin Intensivmed Notfmed. 2017 Nov;112(8):687-94. PMID: 28913684. doi: 10.1007/s00063-017-0344-3.

21. Geisler F, Kunz A, Winter B, Rozanski M, Waldschmidt C, Weber JE, et al. Telemedicine in Prehospital Acute Stroke Care. J Am Heart Assoc. 2019 Mar 19;8(6):e011729. PMID: 30879372. doi: 10.1161/jaha.118.011729.

22. Lai L, Wittbold KA, Dadabhoy FZ, Sato R, Landman AB, Schwamm LH, et al. Digital triage: Novel strategies for population health management in response to the COVID-19 pandemic. HealthCare. 2020 Dec;8(4):7. PMID: WOS:000591980800003. doi: 10.1016/j.hjdsi.2020.100493.

23. Agbakoba R, McGee-Lennon M, Bouamrane MM, Watson N, Mair F. Implementing a National Scottish Digital Health & Wellbeing Service at Scale: A Qualitative Study of Stakeholders' Views. Stud Health Technol Inform. 2015;216:487-91. PMID: 26262098.

24. Zolfo M, Bateganya MH, Adetifa IM, Colebunders R, Lynen L. A telemedicine service for HIV/AIDS physicians working in developing countries. J Telemed Telecare. 2011;17(2):65-70. PMID: 21078680. doi: 10.1258/jtt.2010.100308.

25. Chen J, Jiao Y, Lu C, Zhou J, Zhang Z, Zhou C. A nationwide telepathology consultation and quality control program in China: implementation and result analysis. Diagn Pathol. 2014;9 Suppl 1(Suppl 1):S2. PMID: 25565398. doi: 10.1186/1746-1596-9-s1-s2.

26. Santos MR, Bax MP, Kalra D. Dealing with the Archetypes Development Process for a Regional EHR System. Appl Clin Inform. 2012;3(3):258-75. PMID: WOS:000317183900001. doi: 10.4338/aci-2011-12-ra-0074.

27. Shiferaw F, Zolfo M. The role of information communication technology (ICT) towards universal health coverage: the first steps of a telemedicine project in Ethiopia. Global Health Action. 2012;5:1-8. PMID: WOS:000302796900001. doi: 10.3402/gha.v5i0.15638.

28. Singh T, Ngoh CLY, Wong WK, Khan BA. Impact of Telemedicine on Hospitalisation and Mortality Rates in Community-Based Haemodialysis Centres in Singapore During the COVID-19 Pandemic. Annals Academy of Medicine Singapore. 2020 Oct;49(10):756-63. PMID: WOS:000591608700006. doi: 10.47102/annals-acadmedsg.2020369.

29. Millan-Cayetano JF, Herrera-Ibarra R, Rivas-Ruiz F, Garcia-Serrato P, Garcia-Montero P, Blazquez-Sanchez N, et al. Impact of a Community Intervention for Early Skin Cancer Diagniosis Implementing Teledermatology. Acta Dermatovenerologica Croatica. 2020;28(2):75-9. PMID: WOS:000603399300004.

30. Resneck JS, Abrouk M, Steuer M, Tam A, Yen A, Lee I, et al. Choice, Transparency, Coordination, and Quality Among Direct-to-Consumer Telemedicine Websites and Apps Treating Skin Disease. JAMA Dermatol. 2016 Jul;152(7):768-75. PMID: WOS:000379590100006. doi: 10.1001/jamadermatol.2016.1774.

31. Ratzan SC, Weinberger MB, Apfel F, Kocharian G. The Digital Health Scorecard: A New Health Literacy Metric for NCD Prevention and Care. Global Heart. 2013 Jun;8(2):171-9. PMID: WOS:000218857000011. doi: 10.1016/j.gheart.2013.05.006.

32. Nohra C, Wong MC, Turner P, Almond H, Parv L, Gilstad H, et al. Citizens' Access to Their Digital Health Data in Eleven Countries - A Comparative Study. In: Hoerbst A, Hackl WO, DeKeizer N, Prokosch HU, HercigonjaSzekeres M, DeLusignan S, editors. Exploring Complexity in Health: An Interdisciplinary Systems Approach. Amsterdam: Ios Press; 2016. p. 685-9.

Publications where the intervention is not the main object of the publication

1. Barbieri JS, Kovarik CL. Inpatient and Tertiary Consultations in Teledermatology. Curr Dermatol Rep. 2016 Jun;5(2):83-9. PMID: WOS:000386358100003. doi: 10.1007/s13671-016-0135-8.

2. Li D, Chao JQ, Kong J, Cao G, Lv MR, Zhang M. The efficiency analysis and spatial implications of health information technology: A regional exploratory study in China. Health Inform J. 2020 Sep;26(3):1700-13. PMID: WOS:000500665700001. doi: 10.1177/1460458219889794.

3. Gray GA, Stamm BH, Toevs S, Reischl U, Yarrington D. Study of participating and nonparticipating states' telemedicine Medicaid reimbursement status: Its impact on Idaho's policymaking process. Telemed J E Health. 2006 Dec;12(6):681-90. PMID: 17250490. doi: 10.1089/tmj.2006.12.681.

4. Sutherland JJ, Morrison RD, McNaughton CD, Daly TM, Milne SB, Daniels JS, et al. Assessment of Patient Medication Adherence, Medical Record Accuracy, and Medication Blood Concentrations for Prescription and Over-the-Counter Medications. JAMA Netw Open. 2018 Nov 2;1(7):e184196. PMID: 30646345. doi: 10.1001/jamanetworkopen.2018.4196.

5. Maria MS, Silvia AN, Beatriz DG, Andrew D, Guillermo PF. Health care in rural areas: proposal of a new telemedicine program assisted from the reference health centers, for a sustainable digitization and its contribution to the carbon footprint reduction. Heliyon. 2022 Jul;8(7):e09812. PMID: 35800252. doi: 10.1016/j.heliyon.2022.e09812.

6. Wadali JS, Sood SP, Kaushish R, Syed-Abdul S, Khosla PK, Bhatia M. Evaluation of Free, Open-source, Web-based DICOM Viewers for the Indian National Telemedicine Service (eSanjeevani). J Digit Imaging. 2020 Dec;33(6):1499-513. PMID: WOS:000548094800001. doi: 10.1007/s10278-020-00368-4.

7. Paul DL, McDaniel RR, Jr. Facilitating telemedicine project sustainability in medically underserved areas: a healthcare provider participant perspective. BMC Health Serv Res. 2016 Apr 26;16:148. PMID: 27112268. doi: 10.1186/s12913-016-1401-y.

8. Patel SA, Vashist K, Jarhyan P, Sharma H, Gupta P, Jindal D, et al. A model for national assessment of barriers for implementing digital technology interventions to improve hypertension management in the public health care system in India. BMC Health Serv Res. 2021 Oct;21(1):11. PMID: WOS:000707587000001. doi: 10.1186/s12913-021-06999-9.

9. Lie DYC, Lie T, Lie P, Nguyen T. Stop the Spread of COVID-19 with Digital Health: A Real-World Case Study. 2020 3rd International Conference on Digital Medicine and Image Processing; Kyoto, Japan: Association for Computing Machinery; 2020. p. 26–32.

10. Min J, Gurka KK, Kalesan B, Bian J, Prosperi M. Injury Burden in the United States: Accurate, Reliable, and Timely Surveillance Using Electronic Health Care Data. Am J Public Health. 2019 Dec;109(12):1702-6. PMID: WOS:000500737800028. doi: 10.2105/ajph.2019.305306.

11. Wright HR, Diamond JP. Service innovation in glaucoma management: using a Web-based electronic patient record to facilitate virtual specialist supervision of a shared care glaucoma programme. Br J Ophthalmol. 2015 Mar;99(3):313-7. PMID: 25336582. doi: 10.1136/bjophthalmol-2014-305588.

12. Chrapkowska C, Galanis I, Kark M, Lepp T, Lindstrand A, Roth A, et al. Validation of the new Swedish vaccination register - Accuracy and completeness of register data. Vaccine. 2020 May;38(25):4104-10. PMID: WOS:000531834000009. doi: 10.1016/j.vaccine.2020.04.020.

13. Kopetsch T, John S. The Electronic Health Information System (eGIS) of the National Association of Statutory Health Insurance Physicians (KBV). A basis for small-scale analyses of health-care provision. Bundesgesundheitsblatt-Gesund. 2014 Feb;57(2):207-14. PMID: WOS:000330729200008. doi: 10.1007/s00103-013-1891-2.

14. Shea CM, Reiter KL, Weaver MA, McIntyre M, Mose J, Thornhill J, et al. Stage 1 of the meaningful use incentive program for electronic health records: a study of readiness for change in ambulatory practice settings in one integrated delivery system. Bmc Medical Informatics and Decision Making. 2014 Dec;14:7. PMID: WOS:000347198300001. doi: 10.1186/s12911-014-0119-1.

15. De Luca G, Brattstrom M, Morreale P. Designing a Secure e-Health Network System. New York: Ieee; 2016. 99-103 p. ISBN: 978-1-4673-9519-9.

16. Leitsalu L, Alavere H, Tammesoo ML, Leego E, Metspalu A. Linking a Population Biobank with National Health Registries-The Estonian Experience. Journal of Personalized Medicine. 2015 Jun;5(2):96-106. PMID: WOS:000457944500005. doi: 10.3390/jpm5020096.

17. Gregor-Haack J, Busse T, Hagenmeyer EG. [The new approval process for the reimbursement of digital health applications (DiGA) from the perspective of the German statutory health insurance]. Bundesgesundheitsblatt Gesundheitsforschung Gesundheitsschutz. 2021 Oct;64(10):1220-7. PMID: 34459939. doi: 10.1007/s00103-021-03401-1.

18. Sousa J, Barata J, van Woerden HC, Kee F. COVID-19 Symptoms app analysis to foresee healthcare impacts: Evidence from Northern Ireland. Appl Soft Comput. 2022 Feb;116:10. PMID: WOS:000790114500009. doi: 10.1016/j.asoc.2021.108324.

19. Kushniruk A, Kaipio J, Nieminen M, Nohr C, Borycki E. Comparing Approaches to Measuring the Adoption and Usability of Electronic Health Records: Lessons Learned from Canada, Denmark and Finland. In: Lehmann CU, Ammenwerth E, Nohr C, editors. Medinfo 2013: Proceedings of the 14th World Congress on Medical and Health Informatics, Pts 1 and 2. Amsterdam: Ios Press; 2013. p. 367-71.

20. Madhavan S, Bastarache L, Brown JS, Butte AJ, Dorr DA, Embi PJ, et al. Use of electronic health records to support a public health response to the COVID-19 pandemic in the United States: a perspective from 15 academic medical centers. J Am Med Inform Assoc. 2021 Feb 15;28(2):393-401. PMID: 33260207. doi: 10.1093/jamia/ocaa287.

21. Williamson T, Green ME, Birtwhistle R, Khan S, Garies S, Wong ST, et al. Validating the 8 CPCSSN Case Definitions for Chronic Disease Surveillance in a Primary Care Database of Electronic Health Records. Ann Fam Med. 2014 Jul-Aug;12(4):367-72. PMID: WOS:000339276300014. doi: 10.1370/afm.1644.

22. Villumsen S, Nøhr C, Faxvaag A. Translating eHealth Visions from Strategy to Practice - A Benefit Management Approach. Stud Health Technol Inform. 2018;247:885-9. PMID: 29678088.

23. Latifi R, Dasho E, Lecaj I, Latifi K, Bekteshi F, Hadeed M, et al. Beyond "Initiate-Build-Operate-Transfer" strategy for creating sustainable telemedicine programs: lesson from the first decade. Telemed J E Health. 2012 Jun;18(5):388-90. PMID: 22524525. doi: 10.1089/tmj.2011.0263.

24. Stroetmann KA, Jones T, Dobrev A, Stroetmann VN. An evaluation of the economic impact of ten European e-health applications. J Telemed Telecare. 2007;13:S62-S4. PMID: WOS:000248602300026.

25. Zeng XM, Forrestal EJ, Cellucci LW, Kennedy MH, Smith D. Using Electronic Health Records and Data Warehouse Collaboratively in Community Health Centers. Journal of Cases on Information Technology. 2013 Oct-Dec;15(4):45-62. PMID: WOS:000212921300004. doi: 10.4018/jcit.2013100104.

26. Rogers J. Coding telehealth services during COVID-19. Nurse Pract. 2021 Feb 1;46(2):10-2. PMID: 33399335. doi: 10.1097/01.NPR.0000731584.40074.eb.

27. Pappas Y, Vseteckova J, Mastellos N, Greenfield G, Randhawa G. Diagnosis and Decision-Making in Telemedicine. J Patient Experience. 2019 Dec;6(4):296-304. PMID: WOS:000513887800007. doi: 10.1177/2374373518803617.

28. Nichols GA, Desai J, Elston Lafata J, Lawrence JM, O'Connor PJ, Pathak RD, et al. Construction of a multisite DataLink using electronic health records for the identification, surveillance, prevention, and management of diabetes mellitus: the SUPREME-DM project. Prev Chronic Dis. 2012;9:E110. PMID: 22677160. doi: 10.5888/pcd9.110311.

29. Winkelman TNA, Margolis KL, Waring S, Bodurtha PJ, Khazanchi R, Gildemeister S, et al. Minnesota Electronic Health Record Consortium COVID-19 Project: Informing Pandemic Response Through Statewide Collaboration Using Observational Data. Public Health Rep. 2022 Mar-Apr;137(2):263-71. PMID: 35060411. doi: 10.1177/00333549211061317.

30. Hägglund M, Scott Duncan T, Kai-Larsen K, Hedlin G, Krakau I. IntegrIT - Towards Utilizing the Swedish National Health Information Exchange Platform for Clinical Research. Stud Health Technol Inform. 2017;235:146-50. PMID: 28423772.

Publications published in another language than English, German, or Chinese

1. Alasheev AM, Hubert GJ, Santo GC, Vanhooren GT, Zvan B, Campos ST, et al. [Recommendations on telestroke in Europe]. Zh Nevrol Psikhiatr Im S S Korsakova. 2020;120(3. Vyp. 2):33-41. PMID: 32307428. doi: 10.17116/jnevro202012003233.

2. Alfaro M, Bonis J, Bravo R, Fluiters E, Minué S. [New technologies in primary care: people, machines, records, and networks. SESPAS Report 2012]. Gac Sanit. 2012 Mar;26 Suppl 1:107-12. PMID: 22336322. doi: 10.1016/j.gaceta.2011.12.005.

3. Amato S, Di Giovanni C, Politi M, De Salazar V. [The telemedicine as tool of management on chronic disease: the experience of ASL ROMA 3 (Italian Local Authority). Impact, outcomes and results]. Ig Sanita Pubbl. 2019 May-Jun;75(3):231-44. PMID: 31645064.

4. Bill G, Crisci CD, Canet T. [The Telehealth Network of the Americas and its role in primary health care]. Rev Panam Salud Publica. 2014 May-Jun;35(5-6):442-5. PMID: 25211574.

5. Cerda-Calafat I, Continente-Gonzalo M, Garcia-Lopez C, Guanyabens-Calvet J. Personal Health Folder. Med Clin (Barc). 2010 Feb;134:63-6. PMID: WOS:000277354300012. doi: 10.1016/s0025-7753(10)70012-9.

6. Consorti F. Health record in the Age of e-Health. Clin Ter. 2011 Nov-Dec;162(6):585-9. PMID: WOS:000299587600014.

7. Kern J, Erceg M, Poljicanin T. [Efficacy of public health surveillance systems]. Acta Med Croatica. 2010 Dec;64(5):415-23. PMID: 21692266.

8. Kiefer B. [Health politics, 2012]. Rev Med Suisse. 2012 Jan 18;8(324):144. PMID: 23185829.

9. Lopez DM. A reference Model for the Specification and Analysis of Information Systems for Public Health Surveillance. Sistemas & Telematica. 2007;5(10):29-46. PMID: WOS:000215564200002.

10. Mansilla E, Mazzon E, Carcamo D, Jurado F, Lara L, Arevalo M, et al. Telestroke in Chile: 1 year experience at 7 hospitals. Revista Medica De Chile. 2019 Sep;147(9):1109-15. PMID: WOS:000504650600003.

11. Mantero A, Posteraro A, Giordano G, Tonti G, Pinciroli F. The problem of the correct implementation of Information and Communication Technology in medicine in our country: document of the "e-cardio" Area of the National Association of Cardiologists and Hospital Doctors (ANMCO). G Ital Cardiol. 2013 Dec;14(12):867-74. PMID: WOS:000444319600009. doi: 10.1714/1371.15243.

12. Marco Cuenca G, Salvador Olivan JA. Knowledge representation in electronic interoperable health records: the case of the Digital Health Record of the Spanish National Health System. Scire-Representacion Y Organizacion Del Conocimiento. 2017 Jan-Jun;23(1):25-38. PMID: WOS:000406218700002.

13. Marimon-Sunol S, Rovira-Barbera M, Acedo-Anta M, Nozal-Baldajos MA, Guanyabens-Calvet J. Shared Electronic Health Record in Catalonia, Spain. Med Clin (Barc). 2010 Feb;134:45-8. PMID: WOS:000277354300009. doi: 10.1016/s0025-7753(10)70009-9.

14. Marti M, Balladelli PP, Gherardi A. [Implementation of the eHealth Strategy and Plan of Action in Argentina, 2011-2013]. Rev Panam Salud Publica. 2014 May-Jun;35(5-6):432-6. PMID: 25211572.

15. Merlière Y. [The shared medical record, a digital health record for all]. Soins. 2020 Jan-Feb;65(842):29-32. PMID: 32245555. doi: 10.1016/j.soin.2020.01.005.

16. Morozova LF. [Use of geographic information systems in public health]. Med Parazitol (Mosk). 2014 Jul-Sep(3):46-51. PMID: 25286553.

17. Perrier-Bonnet S. [A telemedicine consultation in the framework of a wound and wound healing network]. Rev Infirm. 2016 Aug-Sep;223:35-7. PMID: 27633698. doi: 10.1016/j.revinf.2016.06.010.

18. Cruz-Melendez C, Lopez ODV. Public policies oriented to e-health in Mexico during the Covid-19 crisis. Revista De Salud Publica-Cordoba. 2020 Dec:51-64. PMID: WOS:000607738600005. doi: 10.31052/1853.1180.v0.v0.31329.

19. Correia A, Azevedo V, Lapão LV. [Implementation of Telemedicine in Cape Verde: Influencing Factors]. Acta Med Port. 2017 Apr 28;30(4):255-62. PMID: 28555550. doi: 10.20344/amp.7578.

20. Tortajada-Goitia B, Morillo-Verdugo R, Margusino-Framinan L, Marcos JA, Fernandez-Llamazares CM. Survey on the situation of telepharmacy as applied to the outpatient care in hospital pharmacy departments in Spain during the COVID-19 pandemic. Farmacia Hospitalaria. 2020 Jul-Aug;44(4):135-40. PMID: WOS:000549399300004. doi: 10.7399/fh.11527.

21. Oliviera DG, Frias PG, Vanderlei LC, Vidal SA, Novaes Mde A, Souza WV. [Analysis of the implementation of the TeleHealth Program in Pernambuco State, Brazil: a case study]. Cad Saude Publica. 2015 Nov;31(11):2379-89. PMID: 26840817. doi: 10.1590/0102-311x00125914.

22. Costa JFR, Portela MC. [Views of health system administrators, professionals, and users concerning the electronic health record and facilitators and obstacles to its implementation]. Cad Saude Publica. 2018 Feb 5;34(1):e00187916. PMID: 29412325. doi: 10.1590/0102-311x00187916.

23. Zsuzsa G, Sandor B, Noemi SM, Orsolya N. Possibilities of telemedicine regarding the COVID-19 pandemic in light of the international and Hungarian experiences and recommendations. Orv Hetil. 2020 Jun;161(24):983-92. PMID: WOS:000537792800001. doi: 10.1556/650.2020.31873.

24. Polton D. [Health data]. Med Sci (Paris). 2018 May;34(5):449-55. PMID: 29900849. doi: 10.1051/medsci/20183405018.

25. Mesa M, Perez HI. The medical act in the era of telemedicine. Revista Medica De Chile. 2020 Jun;148(6):852-7. PMID: WOS:000576482400020.

26. Ramos AC, Buceta BB, Da Silva AF, Lorenzo RB. eHealth in Spain: evolution, current status and future prospects. Saude E Sociedade. 2020;29(4):12. PMID: WOS:000610972300001. doi: 10.1590/s0104-12902020190886.

27. Caetano R, Silva AB, Guedes A, de Paiva CCN, Ribeiro GD, Santos DL, et al. Challenges and opportunities for telehealth during the COVID-19 pandemic: ideas on spaces and initiatives in the Brazilian context. Cad Saude Publica. 2020;36(5):16. PMID: WOS:000538429700001. doi: 10.1590/0102-311x00088920.

28. Lee YM. A debate about telemedicine in South Korea. Journal of the Korean Medical Association. 2016 Nov;59(11):828-31. PMID: WOS:000391058800001. doi: 10.5124/jkma.2016.59.11.828.

29. Ryu S, Lee JG, Kim KH. Current State of u-Health and Its Developmental Strategies in Korea. Journal of the Korean Medical Association. 2009 Dec;52(12):1141-7. PMID: WOS:000273151700003. doi: 10.5124/jkma.2009.52.12.1141.
