# Supplementary material for: Mapping Digital Public Health Interventions Among Existing Digital Technologies and Internet-Based Interventions to Maintain and Improve Population Health in Practice: Scoping Review
Source: J Med Internet Res. 2024 Jul 17;26:e53927. doi: 10.2196/53927 (PMC11292160; doi:10.2196/53927)
Supplement: Multimedia Appendix 4 [file jmir_v26i1e53927_app4.pdf]

Maaß L, Angoumis K, Freye M, Pan CC. Mapping Digital Public Health Interventions Among Existing Digital Technologies and Internet-Based Interventions to Maintain and Improve Population Health in Practice: Scoping Review. Journal of Medical Internet Research 2024; 26: e53927. DOI: 10.2196/53927.

Multimedia Appendix 4: Heat Maps for selected intervention types

Telemedicine (n=49)

	Research	Financing	Preparedness for public health emergencies	Human resources	Governance	Health protection	Health information systems	Disease prevention	Health promotion	Social participation and health communication	Health care
**Calculate**	0	0	0	0	0	0	1	0	0	0	0
**Preventative behavior change**	0	0	0	0	0	0	0	0	1	0	0
**Active monitoring**	0	0	0	0	0	0	0	0	0	1	3
**simple monitoring**	0	0	0	1	0	0	0	0	0	0	0
**Self-manage**	0	1	0	0	0	0	0	0	2	0	2
**System services**	0	0	0	0	0	0	0	0	0	0	0
**Inform**	0	0	1	2	1	0	0	1	0	0	3
**Communicate**	0	1	0	4	0	1	0	0	2	0	20
**Diagnose**	0	0	2	3	2	1	1	1	0	1	11
**Treat**	0	0	0	3	2	0	0	0	1	0	3

Maaß L, Angoumis K, Freye M, Pan CC. Mapping Digital Public Health Interventions Among Existing Digital Technologies and Internet-Based Interventions to Maintain and Improve Population Health in Practice: Scoping Review. Journal of Medical Internet Research 2024; 26: e53927. DOI: 10.2196/53927.

Health or medical apps (n=28)

	Research	Financing	Preparedness for public health emergencies	Human resources	Governance	Health protection	Health information systems	Disease prevention	Health promotion	Social participation and health communication	Health care
**Calculate**	0	0	0	0	0	0	0	0	0	0	0
**Preventative behavior change**	1	0	0	0	0	1	0	3	1	0	0
**Active monitoring**	1	1	1	1	0	0	1	0	1	1	1
**simple monitoring**	0	0	1	0	1	1	1	0	0	0	1
**Self-manage**	0	0	0	1	0	1	1	0	0	2	1
**System services**	0	0	0	0	0	0	0	0	0	0	0
**Inform**	0	0	1	3	1	1	0	4	1	3	3
**Communicate**	0	0	0	0	0	1	0	1	1	2	1
**Diagnose**	0	0	0	0	0	0	0	0	1	1	2
**Treat**	0	0	1	0	0	0	0	1	1	0	1

Maaß L, Angoumis K, Freye M, Pan CC. Mapping Digital Public Health Interventions Among Existing Digital Technologies and Internet-Based Interventions to Maintain and Improve Population Health in Practice: Scoping Review. Journal of Medical Internet Research 2024; 26: e53927. DOI: 10.2196/53927.

Electronic health record (n=23)

	Research	Financing	Preparedness for public health emergencies	Human resources	Governance	Health protection	Health information systems	Disease prevention	Health promotion	Social participation and health communication	Health care
**Calculate**	0	0	0	0	0	0	0	0	0	0	0
**Preventative behavior change**	0	0	0	0	0	0	0	0	0	0	0
**Active monitoring**	0	0	0	0	0	0	0	0	0	0	0
**simple monitoring**	0	0	0	0	0	0	0	0	0	0	0
**Self-manage**	1	0	0	0	0	0	2	0	0	1	0
**System services**	0	0	0	0	0	0	0	0	0	0	0
**Inform**	0	1	1	0	1	1	2	0	0	0	6
**Communicate**	0	0	0	0	0	0	2	0	0	0	0
**Diagnose**	1	1	0	0	2	1	2	0	0	0	2
**Treat**	0	1	1	0	1	1	1	0	0	1	3
