# Supplementary material for: Mapping Digital Public Health Interventions Among Existing Digital Technologies and Internet-Based Interventions to Maintain and Improve Population Health in Practice: Scoping Review
Source: J Med Internet Res. 2024 Jul 17;26:e53927. doi: 10.2196/53927 (PMC11292160; doi:10.2196/53927)
Supplement: Multimedia Appendix 5 [file jmir_v26i1e53927_app5.pdf]

Maaß L, Angoumis K, Freye M, Pan CC. Mapping Digital Public Health Interventions Among Existing Digital Technologies and Internet-Based Interventions to Maintain and Improve Population Health in Practice: Scoping Review. Journal of Medical Internet Research 2024; 26: e53927. DOI: 10.2196/53927.

Multimedia Appendix 6: PRISMA Extension for Scoping Reviews (PRISMA-ScR) Checklist for this Manuscript

Section	Item	PRISMA-ScR checklist item	Statement in Manuscript
**Title**			
Title	1	Identify the report as a scoping review.	Mapping digital public health interventions in practice: **A scoping review** of existing digital technologies and Internet-based interventions to maintain and improve population health
**Abstract**			
Structured summary	2	Provide a structured summary including, as applicable: background, objectives, eligibility criteria, sources of evidence, charting methods, results and conclusions that relate to the review question(s) and objective(s).	See abstract
**Introduction**			
Rationale	3	Describe the rationale for the review in the context of what is already known. Explain why the review question(s)/objective(s) lend themselves to a scoping review approach.	"Still, to our knowledge, no study has yet attempted to holistically map the heterogeneous landscape of digital public health interventions (DiPHI). […] More importantly, a multidisciplinary and globally shared understanding of central terms for digitalizing health and digital public health (DiPH) is still missing. […] Eventually, developing, implementing, integrating, and evaluating needs-based digital public health interventions requires a clear and shared understanding of the specific characteristics of digital health technologies for public health purposes"
Objectives	4	Provide an explicit statement of the question(s) and objective(s) being addressed with reference to their key elements (e.g., population or participants, concepts and context), or other relevant key elements used to conceptualize the review question(s) and/or objective(s)).	"This scoping review primarily aims to outline a comprehensive range of real-world proposed to implemented DiPHI, including different levels of prevention, healthcare, and public health research initiatives. […] The second objective is to map the landscape of existing DiPHI, shedding light on their self-reported digital health functions (based on the evidence standards framework for digital health technologies (ESF) from the National Institute for Health and Care Excellence (NICE) and the addressed ESPHF as defined by the WHO."
**Methods**			
Protocol and registration	5	Indicate if a review protocol exists, if and where it can be accessed (e.g., web address), and, if available, provide registration information including registration number.	The protocol was registered at PROSPERO (ID CRD42021265562) and published (available under DOI: 10.2196/33404)

Section	Item	PRISMA-ScR checklist item	Statement in Manuscript
**Methods**			
Eligibility criteria	6	Specify the characteristics of the sources of evidence (e.g., years considered, language, publication status) used as criteria for eligibility, and provide a rationale.	- The inclusion criteria follow the PIS (Participants, Intervention, and Study design) format, additionally to the general requirements of full-text availability (Accessibility) and publication language.<br>- Language: Published in English, Chinese, or German<br>- Years considered: No restriction<br><br>PIS Components:<br>- P: The study focuses on the community level or above (regional or national) of the general population.<br>- I: The paper describes a concrete DiPHI, the DiPHI is paid or reimbursed by the government or health insurance, the DiPHI uses the Internet and/or Bluetooth to provide its core function or service.<br>- S: All original peer-reviewed studies, reports, books, book chapters, or peer-reviewed conference papers that have a description of a DiPHI as their primary intervention component
Information sources	7	Describe all information sources (e.g., databases with dates of coverage, contact with authors to identify additional sources) in the search, as well as the date the most recent search was executed.	Initial search on February 19, 2021, in CENTRAL (Cochrane Central Register of Controlled Trials), PubMed, and Web of Science. […] Additional search on December 1, 2021, in Association for Computing Machinery (ACM) Full-Text Collection and IEEE (Institute of Electrical and Electronics Engineers) Xplore. […] Updated search (for all 5 databases) on October 5, 2022. […] Same search string for all databases, no search limits, no alerts, no manual searches in journals. Reference list search for topic-wise potentially fitting reviews (no additionally included references) […] Missing data for included publications was requested from authors via mail.”
Search	8	Present the full electronic search strategy for at least one database, including any limits used, such that it could be repeated.	Multimedia Appendix 1
Selection of sources of evidence	9	State the process for selecting sources of evidence (i.e., screening, eligibility) included in the scoping review.	“Three authors (LM, MF, KA) independently screened the identified references for suitability, however KA did not participate in the title- and abstract screening in 2021, whereas MF did not take part in the updated search in 2022. After each screening phase, CCP was presented with the screening choices and decided for inclusion or exclusion in case of different selections. References published in another language than English, Chinese, or German were excluded during full-text screening. Chinese references were screened only by CCP. “<br>- Excluded Full-texts are presented in Multimedia Appendix 2 with an exclusion reason<br>- Flow chart of the search and screening process is presented in Figure 2.

Section	Item	PRISMA-ScR checklist item	Statement in Manuscript
**Methods**			
Data charting process	10	Describe the methods of charting data from the included sources of evidence (e.g., piloted forms; forms that have been tested by the team before their use, whether data charting was done independently, in duplicate) and any processes for obtaining and confirming data from investigators.	"Two authors (LM and KA) independently extracted data in a Microsoft Excel 2019 sheet. Discrepancies were resolved through discussion between the two authors and CCP. […] The extraction for most data followed a pre-defined coding table which was added if necessary (Figure 3) […] The extraction sheet was piloted with the first 10 included publications. […] Missing data for included publications was requested from authors via mail."
Data items	11	List and define all variables for which data were sought and any assumptions and simplifications made.	Figure 3
Critical appraisal of individual sources of evidence	12	***If done,*** provide a rationale for conducting a critical appraisal of included sources of evidence; describe the methods used and how this information was used in any data synthesis (if appropriate).	Not conducted
Summary measures	13	*Not applicable for scoping reviews.*	-
Synthesis of results	14	Describe the methods of handling and summarizing the data that were charted.	"For the qualitative analysis, we grouped the identified references by intervention types based on our study protocol and summarized the extracted data (see Figure 3) for each intervention type. Additional intervention types were added in an inductive procedure where needed."
Risk of bias across studies	15	*Not applicable for scoping reviews.*	-
Additional analyses	16	*Not applicable for scoping reviews.*	-
**Results**			
Selection of sources of evidence	17	Give numbers of sources of evidence screened, assessed for eligibility, and included in the review, with reasons for exclusions at each stage, ideally using a flow diagram.	Figure 2 (flow diagram) & Multimedia Appendix 2 (for list of excluded full-texts)
Characteristics of sources of evidence	18	For each source of evidence, present characteristics for which data were charted and provide the citations.	Multimedia Appendix 3
Critical appraisal within sources of evidence	19	***If done***, present data on critical appraisal of included sources of evidence (see item 12).	Not conducted
Results of individual sources of evidence	20	For each included source of evidence, present the relevant data that were charted that relate to the review question(s) and objective(s).	Multimedia Appendix 3

Section	Item	PRISMA-ScR checklist item	Statement in Manuscript
**Results**			
Synthesis of results	21	Summarize and/or present the charting results as they relate to the review question(s) and objective(s).	Figure 4, 5, 6, 7.
Risk of bias across studies	22	*Not applicable for scoping reviews.*	-
Additional analyses	23	*Not applicable for scoping reviews.*	-
**Discussion**			
Summary of evidence	24	Summarize the main results (including an overview of concepts, themes, and types of evidence available), explain how they relate to the review question(s) and objectives, and consider the relevance to key groups.	Section *Discussion of results*
Limitations	25	Discuss the limitations of the scoping review process.	Section *Strengths and limitations*
Conclusions	26	Provide a general interpretation of the results with respect to the review question(s) and objective(s), as well as potential implications and/or next steps.	"This scoping review has shown that digital public health interventions are distinctly diverse not only in their use cases between individual intervention groups, but also within such groups. Therefore, it is crucial to use precise terminology when planning, implementing, or evaluating DiPHIs. For instance, instead of phrasing an intervention as an electronic health record, one should ask for an intervention with a specific set of technological functions, non-technical characteristics, determined use cases (from a digital health and public health perspective), and defined user groups."
**Funding**			
Funding	27	Describe sources of funding for the included sources of evidence, as well as sources of funding for the scoping review. Describe the role of the funders of the scoping review.	No funding received for this scoping review
